# Trends in adolescent pertussis burden: a systematic analysis from 1990 to 2021 in global, regional, and national within the global burden of disease study 2021, with forecasts for 2046

**DOI:** 10.3389/fped.2025.1601110

**Published:** 2025-09-24

**Authors:** Huihui Zhu, Fangfang Lv, Shunhang Wen, Ming Xu, Hailin Zhang

**Affiliations:** ^1^ Department of Child Health Care, The Second Affiliated Hospital & Yuying Children’s Hospital, Wenzhou Medical University, Wenzhou, China; ^2^ Department of Pediatric Pulmonology, The Second Affiliated Hospital & Yuying Children’s Hospital, Wenzhou Medical University, Wenzhou, China

**Keywords:** pertussis, global burden of disease, socio-demographic index, adolescent, estimated annual percentage change

## Abstract

**Background:**

Pertussis, a highly contagious respiratory disease caused by Bordetella pertussis, traditionally associated with children, is now increasingly transmitted by adolescents (ages 10–19) and adults. Using data from the Global Burden of Diseases, Injuries, and Risk Factors Study 2021 (GBD 2021), we estimated the disease burden of pertussis among adolescents.

**Methods:**

We analyzed adolescent pertussis data from GBD 2021, assessing prevalence, incidence, mortality, and Disability-Adjusted Life Year (DALY) rate across global, regional, and national levels. Temporal trends were quantified with the Estimated Annual Percentage Change (EAPC), and decomposition and prediction models were employed for further insights.

**Results:**

From 1990 to 2021, adolescent pertussis prevalence, incidence, mortality, and DALY rate declined markedly worldwide. Declines were most pronounced in High and High-middle Socio-Demographic Index (SDI) regions, whereas Low SDI regions maintained the highest burden in 2021. Across the 21 GBD regions, the prevalence, incidence, mortality, and DALY rates have all declined. However, in Central Sub-Saharan Africa, the number of prevalence cases, incidence cases, deaths, and DALYs have increased, while these values have significantly decreased among adolescents in the other 20 GBD regions. Nationally and territorially, the study found substantial variability in the burden of pertussis among adolescents. Decomposition analysis indicated that epidemiological changes primarily drove the decline in most regions, though population growth contributed to increases in some areas. Projection models suggest that the global adolescent pertussis burden will reach its peak in 2022, followed by a gradual decline through 2046.

**Conclusions:**

Despite an overall global decline, pertussis remains a public health concern among adolescents due to their role in disease transmission. Strengthening epidemiological understanding in this age group is essential for effective prevention and control strategies.

## Background

Despite widespread vaccination, pertussis has re-emerged as a global public health challenge (1), characterized by a critical shift in transmission dynamics from children to adolescents and adults acting as the primary reservoir for infants (2–5). The World Health Organization (WHO) has explicitly acknowledged the central role of adolescents in this transmission chain (6); however, prior iterations of the Global Burden of Disease (GBD) studies have not systematically quantified the adolescent-specific burden of pertussis, creating a significant void in the evidence base (7). This is particularly concerning given that protective immunity following primary childhood vaccination wanes rapidly; within 5 to 10 years, a substantial majority of adolescents no longer maintain anti-pertussis toxin (PT) IgG antibody levels above the protective threshold, leaving them susceptible to infection and capable of transmitting the bacterium (8, 9). Infections in this age group consequently fuel a dual burden: they directly increase the risk of infant hospitalization and mortality, and impose a measurable socioeconomic burden through significant school absenteeism—averaging 5.5 days lost per adolescent case—and substantial work absenteeism among their parents (10). Analyses from Canadian models further indicate that these indirect costs substantially surpass the direct medical expenditures associated with treatment (11). Collectively, this lack of age-specific burden data represents a critical evidence gap, hindering the development of targeted booster immunization strategies and effective public health interventions.

To address this evidence gap, we analysed GBD 2021 data on prevalence, incidence, deaths and DALYs due to pertussis in adolescents. We used the EAPC, along with decomposition and prediction models, to analyze global, regional, and national trends in whooping cough from 1990 to 2021. This research provides data and recommendations for global pertussis prevention, aiming to reduce the disease burden and improve adolescent health.

## Methods

### Data source

The data were derived from the GBD study, which spans the period from 1980 to 2021 and includes 204 countries and territories across 21 geographic regions (12). The GBD study comprehensively assesses 369 diseases and injuries and 88 risk factors. The Socio-demographic Index (SDI) ranges from 0 to 1, with a higher score denoting a higher level of socioeconomic development. Based on this metric, regions are categorized into five groups: Low (<0.45), Low-middle (≥ 0.45 to <0.61), Middle (≥0.61 to <0.69), High-middle (≥0.69 to <0.80), and High (≥0.80) (13). All data and methodological details are publicly accessible through the GBD website and the Global Health Data Exchange (GHDx) query tools. The study adheres to the Guidelines for Accurate and Transparent Health Estimates Reporting (GATHER), ensuring transparency, reproducibility, and reliability of the estimates.

### EAPC model analysis

The EAPC model evaluates changes in disease rates over time by calculating the average annual percentage change. It's useful for identifying trends, comparing changes across periods or populations, and is commonly used in epidemiology to guide public health interventions (14).

### Decomposition model analysis

We used Das Gupta's decomposition method to break down changes in pertussis incidence, prevalence, deaths, and DALYs into three factors: population aging, population growth, and epidemiological changes, quantifying each factor's impact on overall trends (15).

### Nordpred model analysis

We applied the Nordpred model, which is grounded in the Age-Period-Cohort (APC) framework, to forecast the adolescent pertussis burden based on its specific suitability for surveillance data structured in 5-year age groups and 5-year calendar intervals—matching exactly the format of GBD 2021 data (16). This approach allowed us to explicitly model age, period, and cohort effects, thereby capturing key demographic and immunization-related trends that are often neglected by simpler time-series or machine learning models. Additionally, the model's integration of smoothing techniques helped reduce random noise, while the inclusion of more recent data improved medium- and long-term prediction accuracy. All statistics were done in R (v 4.3.3).

## Results

### Global level

**Figure 1:**
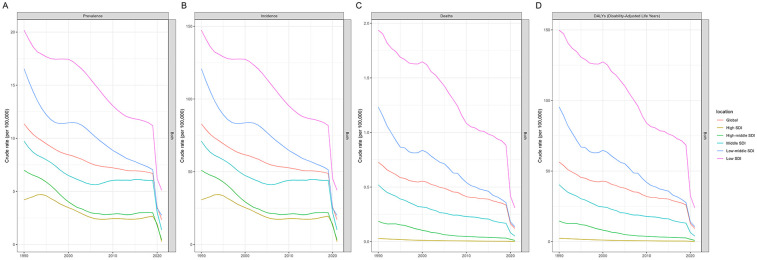
Global and SDI region trajectories of adolescent pertussis prevalence **(A)**, incidence **(B)**, mortality **(C)**, and DALYs rates **(D)**, 1990 to 2021.

From 1990 to 2021, the global burden of pertussis among adolescents showed a consistent and substantial decline in prevalence, incidence, deaths, and DALYs (see Figure 1, Table 1 and Supplementary Tables S1–S3). The total number of prevalent cases decreased by 74.87%, while incidence cases, deaths, and DALYs also fell markedly over this period. By 2021, the global prevalence rate had dropped to 2.33 per 100,000 people, down from 11.36 in 1990, and the incidence rate fell from 82.94 to 17.04 per 100,000. Mortality and DALYs similarly exhibited significant reductions. The EAPC in global adolescent pertussis burden from 1990 to 2021 were −2.44 for prevalence and incidence, −3.25 for mortality, and −3.24 for DALYs, all indicating significant declines (see Supplementary Results for detailed data).

**Table 1:** Global and regional prevalence of adolescent pertussis from 1990 to 2021.

Location	1990		2021		EAPC_95% CI
	Number (95% UI)	Rate (95% UI)	Number (95% UI)	Rate (95% UI)	
Global	119875.55 (91458.13 to 152637.55)	11.36 (8.67 to 14.47)	30,127.90 (20980.14 to 43205.85)	2.33 (1.63 to 3.35)	−2.44 (−3.13 to −1.75)
High SDI	5338.16 (4128.3 to 6782.47)	4.20 (3.25 to 5.34)	332.83 (202.6 to 558.75)	0.28 (0.17 to 0.46)	−3.70 (−4.98 to −2.4)
High-middle SDI	13007.26 (10069.25 to 16537.23)	6.99 (5.41 to 8.88)	670.68 (449.2 to 949.68)	0.44 (0.3 to 0.63)	−4.26 (−5.35 to −3.15)
Middle SDI	36030.31 (27720.19 to 45758.11)	9.72 (7.48 to 12.35)	5173.69 (3290.42 to 7977.18)	1.38 (0.88 to 2.12)	−2.40 (−3.34 to −1.45)
Low-middle SDI	42604.60 (32279.43 to 54190.37)	16.55 (12.54 to 21.05)	10392.82 (6320.83 to 16255.47)	2.75 (1.67 to 4.3)	−3.25 (−3.94 to −2.56)
Low SDI	22812.56 (17247.98 to 28924.42)	20.18 (15.26 to 25.59)	13550.26 (9734.77 to 18,856.16)	5.11 (3.67 to 7.11)	−2.69 (−3.25 to −2.13)
Andean Latin America	986.91 (757.16 to 1252.41)	11.30 (8.67 to 14.35)	366.08 (120.1 to 789.35)	3.20 (1.05 to 6.91)	−2.80 (−3.52 to −2.07)
Australasia	132.57 (102.46 to 168.91)	4.14 (3.2 to 5.27)	0.99 (0.25 to 2.26)	0.03 (0.01 to 0.06)	−5.19 (−8.47 to −1.79)
Caribbean	740.20 (568.26 to 940.97)	10.21 (7.83 to 12.97)	3.11 (0.9 to 14.57)	0.04 (0.01 to 0.19)	−4.18 (−7.3 to −0.95)
Central Asia	1268.11 (971.33 to 1617.4)	9.18 (7.03 to 11.7)	75.09 (41.82 to 129.6)	0.49 (0.28 to 0.85)	−4.44 (−5.75 to −3.11)
Central Europe	686.42 (530.86 to 881.59)	3.38 (2.62 to 4.35)	5.37 (1.68 to 11.94)	0.04 (0.01 to 0.1)	−2.94 (−5.48 to −0.34)
Central Latin America	4822.11 (3680.0 to 6142.32)	12.55 (9.57 to 15.98)	417.03 (240.57 to 661.15)	0.95 (0.55 to 1.51)	−2.45 (−3.72 to −1.17)
Central Sub-Saharan Africa	2424.90 (1833.0 to 3075.25)	19.49 (14.73 to 24.71)	3688.68 (1875.7 to 6463.09)	11.32 (5.76 to 19.84)	−1.46 (−1.83 to −1.09)
East Asia	14393.60 (11137.89 to 18269.8)	6.09 (4.71 to 7.73)	704.75 (370.83 to 1212.74)	0.42 (0.22 to 0.73)	−5.19 (−6.27 to −4.1)
Eastern Europe	4024.88 (3086.13 to 5123.16)	12.46 (9.56 to 15.86)	74.97 (36.27 to 133.62)	0.32 (0.16 to 0.58)	−4.37 (−6.18 to −2.53)
Eastern Sub-Saharan Africa	7507.34 (5695.22 to 9559.77)	16.53 (12.54 to 21.05)	4799.15 (3124.72 to 6976.05)	4.60 (3.0 to 6.69)	−2.69 (−3.33 to −2.05)
High-income Asia Pacific	1837.44 (1424.81 to 2325.37)	6.51 (5.05 to 8.24)	0.35 (0.05 to 1.36)	0.0021 (0.00,033 to 0.0083)	−9.98 (−13.56 to −6.25)
High-income North America	629.86 (480.16 to 819.48)	1.59 (1.21 to 2.07)	222.02 (102.98 to 408.57)	0.47 (0.22 to 0.87)	−0.65 (−2.01 to 0.73)
North Africa and Middle East	8188.97 (6291.33 to 10,377.5)	10.49 (8.06 to 13.3)	1629.66 (812.53 to 2983.54)	1.45 (0.72 to 2.66)	−2.26 (−3.15 to −1.35)
Oceania	222.86 (169.15 to 283.67)	15.09 (11.45 to 19.21)	18.14 (8.73 to 31.91)	0.65 (0.31 to 1.15)	−2.09 (−3.95 to −0.2)
South Asia	41203.62 (31183.51 to 52383.44)	17.47 (13.22 to 22.21)	10627.12 (4699.18 to 19383.87)	3.00 (1.33 to 5.47)	−3.58 (−4.37 to −2.77)
Southeast Asia	13728.17 (10475.15 to 17492.29)	13.23 (10.09 to 16.85)	582.70 (352.72 to 923.45)	0.51 (0.31 to 0.81)	−2.81 (−4.48 to −1.1)
Southern Latin America	670.42 (519.26 to 846.91)	7.26 (5.62 to 9.17)	30.62 (12.79 to 59.69)	0.30 (0.13 to 0.59)	−4.46 (−6.18 to −2.7)
Southern Sub-Saharan Africa	1077.58 (833.57 to 1368.03)	8.91 (6.89 to 11.31)	252.22 (101.04 to 507.82)	1.67 (0.67 to 3.37)	−1.32 (−3.0 to 0.4)
Tropical Latin America	3991.90 (3055.04 to 5081.01)	11.92 (9.12 to 15.17)	505.60 (145.23 to 1131.96)	1.55 (0.44 to 3.46)	−4.81 (−6.88 to −2.7)
Western Europe	2894.03 (2239.98 to 3670.71)	5.58 (4.32 to 7.08)	49.83 (29.78 to 78.68)	0.10 (0.06 to 0.17)	−5.93 (−7.5 to −4.34)
Western Sub-Saharan Africa	8443.67 (6387.0 to 10724.99)	19.54 (14.78 to 24.82)	6074.41 (3852.44 to 8881.82)	5.19 (3.29 to 7.59)	−2.12 (−2.77 to −1.46)

### SDI regional level

Between 1990 and 2021, adolescent pertussis burden decreased across all five SDI regions, with significant disparities in the magnitude of reduction observed (see Figure 1, Table 1, and Supplementary Tables S1–S3). The High-middle SDI region demonstrated the most substantial improvements in prevalent and incident cases, with the number of prevalent cases declining by 94.84% and incident cases decreasing by 94.26%, both with an EAPC of −4.26. For mortality and DALYs, the High SDI region showed the most pronounced reductions, with deaths decreasing by 97.05% (EAPC: −7.83) and DALYs by 96.77% (EAPC: −7.03). In contrast, the Low SDI region exhibited the most modest improvements across all indicators: prevalent cases decreased by 40.60% (EAPC: −2.69), incident cases by 34.81% (EAPC: −2.69), deaths by 62.53% (EAPC: −3.65), and DALYs by 62.43% (EAPC: −3.65). Consequently, the Low SDI region maintained the highest disease burden rates in 2021 (see Supplementary Results for detailed data).

### GBD regional level

Between 1990 and 2021, adolescent pertussis rates (prevalence, incidence, mortality, and DALYs) declined across all 21 GBD regions. However, in terms of absolute numbers, Central Sub-Saharan Africa experienced an increase in prevalent cases, incident cases, deaths, and DALYs. In contrast, the other 20 GBD regions showed a substantial decrease in the absolute burden across all these indicators (see Table 1, Supplementary Tables S1–S3). In 2021, Central Sub-Saharan Africa not only had the highest prevalence and incidence rates (11.32 and 82.66 per 100,000, respectively) but also recorded the highest mortality and DALY rates (0.72 deaths and 55.61 DALYs per 100,000). South Asia reported the highest absolute disease burden, with over 10,600 prevalent cases and 500 deaths in 2021. Conversely, High-income Asia Pacific and Australasia demonstrated the most successful control of adolescent pertussis, both achieving near-elimination status with the lowest absolute numbers and rates globally (see Supplementary Results for detailed data).

From 1990 to 2021, the High-income Asia Pacific region demonstrated the most rapid decline in adolescent pertussis burden, with EAPC values of −9.98 for prevalence, −9.98 for incidence, −11.56 for mortality, and −11.31 for DALYs. In contrast, High-income North America showed the slowest progress, with EAPCs of −0.65 for prevalence, −0.65 for incidence, −0.07 for mortality, and −0.57 for DALYs. A strong inverse correlation was observed between regional SDI and disease burden, with higher SDI regions generally experiencing faster declines (see Supplementary Figure S1). Notably, Western, Eastern, and Central Sub-Saharan Africa regions, along with Andean Latin America and South Asia, consistently exhibited burden rates above the global average (see Supplementary Results for detailed data).

### Countries level

**Figure 2:**
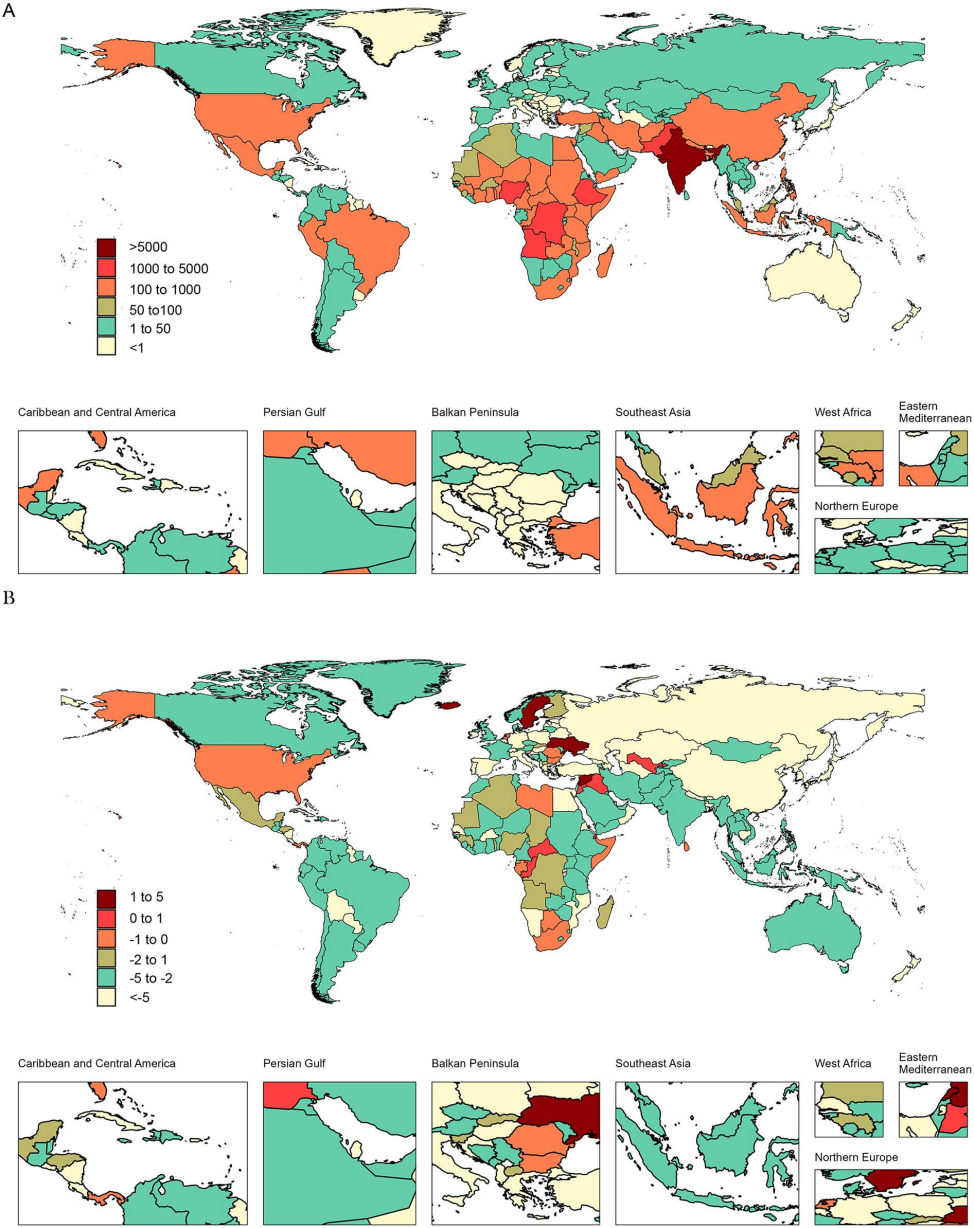
Global maps of adolescent pertussis prevalence cases in 2021 **(A)** EAPC for adolescent Pertussis prevalence **(B)**, 1990 to 2021.

The burden of adolescent pertussis exhibited substantial heterogeneity at the national level from 1990 to 2021. In 2021, India reported the highest absolute numbers across all four indicators—prevalence, incidence, deaths, and DALYs-representing the greatest overall disease burden globally. Meanwhile, Angola and Kiribati showed the highest prevalence and mortality rates, respectively. Several countries and territories, including Niue, Bermuda, and the Republic of Korea, achieved near-elimination status with the lowest reported burden worldwide (see Figure 2, Supplementary Figure S2, Supplementary Tables S4–S7, and Supplementary Results for detailed data).

Between 1990 and 2021, adolescent pertussis burden decreased in most countries, though several notable exceptions emerged. Nicaragua demonstrated the most substantial declines across all indicators, with EAPCs of −16.72 for prevalence and incidence, −18.74 for mortality, and −18.72 for DALYs. Conversely, Iceland showed significant increases in prevalence and incidence (EAPC: 5.38), while Spain experienced the sharpest rise in mortality (EAPC: 15.54). Ukraine recorded the greatest increase in DALYs (EAPC: 3.3) during this period (see Figure 2, Supplementary Figure S3, Supplementary Tables S8–S11, and Supplementary Results for detailed data).

### Decomposition analysis of global, SDI regions and 21 GBD regions adolescent pertussis trends, 1990 to 2021

**Figure 3:**
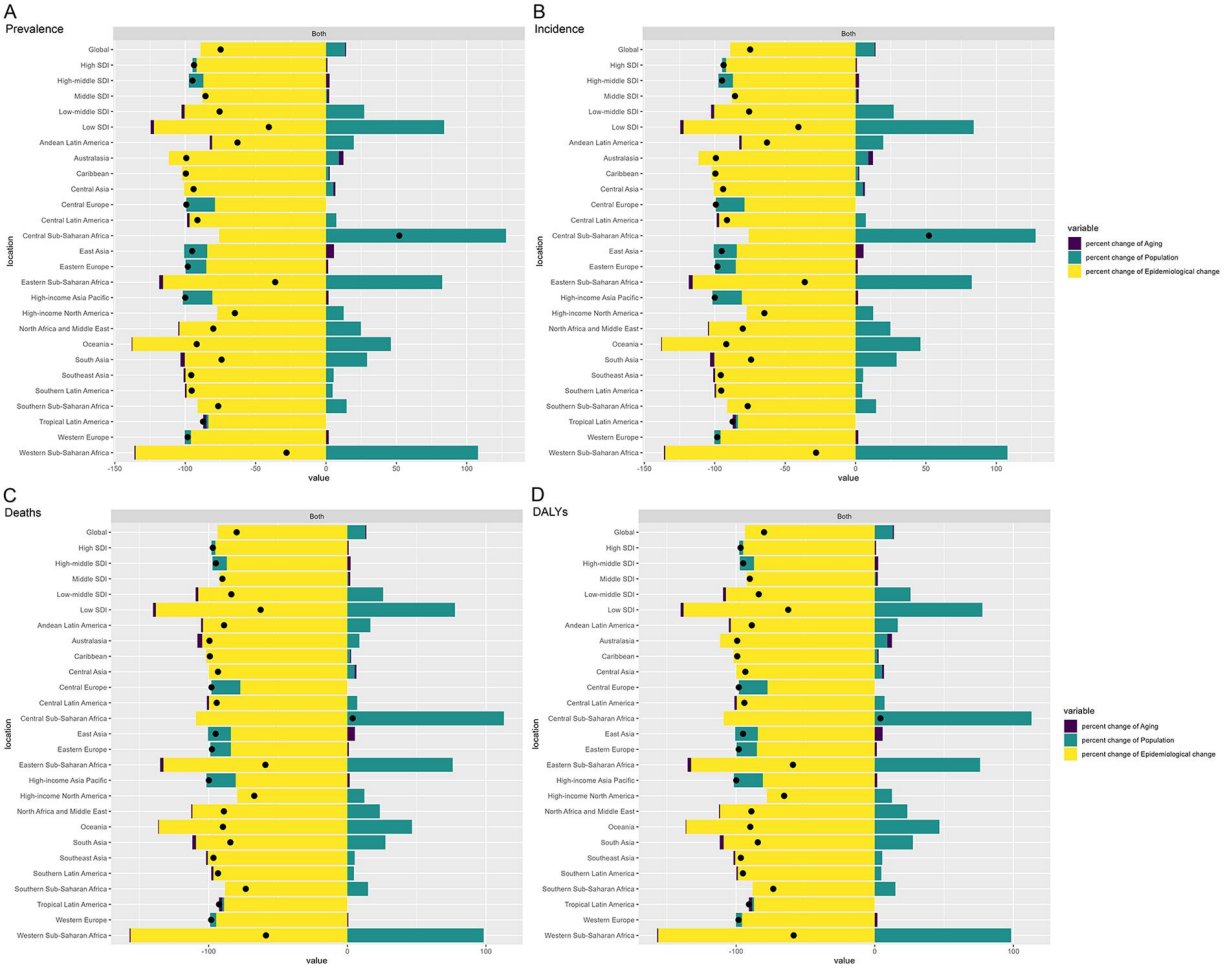
Decomposition analysis of the burden of adolescent pertussis prevalence **(A)**, incidence **(B)**, deaths **(C)**, and DALYs **(D)**, 1990 to 2021.

We used decomposition methods to analyze the contributions of population aging, population growth, and epidemiological changes to the variation in prevalence, incidence, deaths, and DALYs of adolescent pertussis globally, across five SDI regions, and 21 GBD regions (Figure 3, Supplementary Tables S12–S15). Globally, the overall burden of adolescent pertussis has declined, mainly due to epidemiological changes, while population growth has contributed to an increased burden. Among SDI regions, epidemiological changes were the main driver of burden reduction. The Low SDI region saw the slowest reduction, while the High-middle SDI region experienced the fastest decline in prevalence and incidence, and the High SDI region saw the fastest decline in deaths and DALYs. Among GBD regions, Central Sub-Saharan Africa was the only region to see an increase, driven by population growth, while other regions, especially high-income Asia Pacific, saw declines mainly due to epidemiological changes.

### Future burden of pertussis among adolescents worldwide

**Figure 4:**
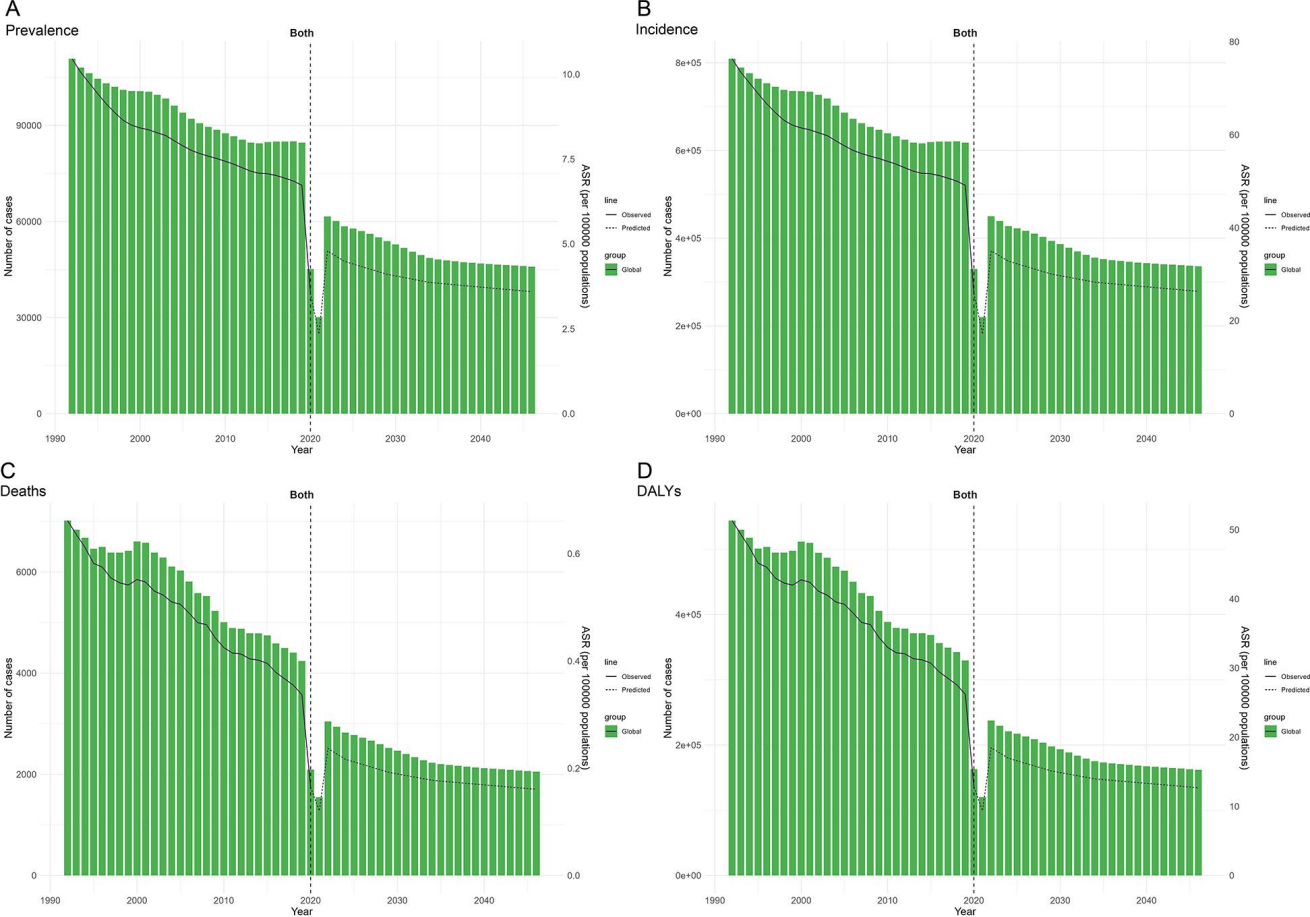
Global adolescent pertussis forecasts: prevalence **(A)**, incidence **(B)**, deaths **(C)**, and DALYs **(D)** from 1992 to 2046.

We predicted future trends in the global burden of adolescent pertussis (Figure 4, Supplementary Tables S16–S19). The burden is expected to increase until 2022, followed by a gradual decline from 2023 onward. In 2022, the prevalence rate was 4.79 per 100,000 people (61,612 cases), an increase of 51.1% from 2021. The incidence rate was 35.02 per 100,000 people (449,961 cases), up 51.12%, the mortality rate was 0.24 per 100,000 (3,045 deaths), up 49.2%, and the DALYs rate was 18.48 years per 100,000 (237,412 DALYs), up 49.4%. Despite the expected decline over the next 20 years, the burden will remain higher than in 2021. By 2046, the predicted prevalence rate is 3.59 per 100,000 people (45,936 cases), incidence rate is 26.31 per 100,000 (336,210 cases), mortality rate is 0.16 per 100,000 (2,053 deaths), and DALYs rate is 12.70 years per 100,000 (162,069 DALYs), all showing increases compared to 2021.

## Discussion

Our study leverages the latest GBD 2021 data to provide contemporary epidemiological insights into adolescent pertussis. Over the past three decades, there has been a general decline in the burden of this disease globally, as well as in five SDI regions and 21 GBD regions, highlighting the success of improved prevention and treatment strategies. However, although the disease burden of pertussis among adolescents in Central Sub-Saharan Africa is declining, the prevalence, incidence, deaths, and DALYs are rising. Our decomposition analysis reveals that population growth is the primary factor driving the increase in Central Sub-Saharan Africa, whereas epidemiological changes are responsible for reductions in other regions. Significant disparities exist among countries. For instance, India reports the highest numbers in prevalence, incidence, deaths, and DALYs, largely due to its vast population. Factors such as access to healthcare, environmental conditions, and cultural influences also play crucial roles (6). Our analysis found a negative correlation between the burden of adolescent pertussis and SDI, with higher SDI regions typically reporting lower rates of prevalence, incidence, mortality, and DALYs due to socioeconomic advancement and stronger healthcare systems. Lower SDI regions should focus on reducing health disparities by improving education, healthcare, and public health awareness. Despite the overall decline in burden, emerging data suggest it may be underestimated. Over the past two decades, there has been a shift in pertussis cases toward older children and adults (17, 18). This aligns with broader multinational analyses pointing to waning immunity and improved diagnosis in older age groups as key drivers of this global phenomenon. For instance, studies in China noted increasing positivity rates among adolescents (19). Similarly, the Netherlands observed a 60% increase in incidence among those aged 10–19 (20), and South Korea reported an incidence of 9.2 per 100,000 in adolescents aged 10–14 in 2018 (21). EU surveillance data further indicate that individuals aged 15 and above accounted for 62% of all pertussis cases (22). This widespread shift in epidemiology is further supported by a large Asian study, in which 4.8% of adolescents showed serological evidence of recent pertussis infection, confirming significant undetected transmission across the region (23). Similarly, Latin America mirrors this trend. Mexico now reports the highest case rates in 10–14-year-olds; however, only Argentina, Chile, Uruguay, and Venezuela include an adolescent Tdap booster in their national schedules. This has resulted in substantial immunity gaps and ongoing silent transmission (24). Collectively, these findings highlight the urgent need for adaptive public health strategies.

The clinical manifestations of pertussis in adolescents are often mild or atypical, such as a chronic cough, making diagnosis challenging and leading to potential underestimation in this population (25). Factors like atypical symptoms, low awareness, and healthcare limitations further complicate the epidemiology and burden of adolescent pertussis, contributing to underdiagnosis and underreporting (26). Predictive analysis shows a significant increase in adolescent pertussis followed by a gradual decline, with a turning point in 2022. This decrease may be partly attributable to global COVID-19 control measures—such as mask-wearing and social distancing—which likely reduced the transmission of respiratory pathogens. However, this remains a speculative interpretation, and our model cannot definitively establish causation; the observed turning point may also be influenced by other factors, such as immunity gaps or reporting disruptions from the pandemic itself. Thus, these predictions must be interpreted with caution due to the inherent limitations of modeling and warrant further investigation.

This study acknowledges limitations of the GBD 2021 study, such as reliance on incomplete or unreliable health data, especially in low- and middle-income countries. Variability in data quality and assumptions in statistical models affect accuracy. Changes in risk factor definitions and delays in data updates complicate comparisons. The GBD also oversimplifies cultural, socioeconomic factors, and health interventions, requiring cautious interpretation.

In summary, despite a significant global decline from 1990 to 2021, the burden of adolescent pertussis remains substantial and is likely underreported. The shifting epidemiology, coupled with significant regional disparities driven by population growth and socioeconomic factors, calls for enhanced age-specific surveillance, vaccination strategies that address waning immunity, and targeted international cooperation to mitigate the burden in this key age group.

## Data Availability

The original contributions presented in the study are included in the article/Supplementary Material, further inquiries can be directed to the corresponding author.
