# Supplementary material for: Trends in adolescent pertussis burden: a systematic analysis from 1990 to 2021 in global, regional, and national within the global burden of disease study 2021, with forecasts for 2046
Source: Front Pediatr. 2025 Sep 24;13:1601110. doi: 10.3389/fped.2025.1601110 (PMC12504105; doi:10.3389/fped.2025.1601110)
Supplement: Supplementary file 4 [file Datasheet1.docx]

**Results**

**Global level**

From 1990 to 2021, the global burden of pertussis among adolescents has shown a consistent decline in prevalence, incidence, mortality, and DALYs rates, indicating a reduction in the global burden of adolescent pertussis (Fig.1, Table 1, and S. Table 1-3). In 2021, the estimated the number of prevalence cases worldwide was 30,127.9 (95% UI: 20,980.14 to 43,205.85), a decrease of 74.87% compared to 119,875.55 cases in 1990 (95% UI: 91,458.13 to 152,637.55). The prevalence rate decreased from 11.36 cases per 100,000 people in 1990 to 2.33 cases per 100,000 people in 2021. Similarly, the incidence cases decreased by 74.87%, from 875,091.54 cases in 1990 (95% UI: 667,644.34 to 1,114,254.13) to 219,933.64 cases in 2021 (95% UI: 153,155.0 to 315,402.68). The incidence rate fell from 82.94 cases per 100,000 people in 1990 (95% UI: 63.28 to 105.61) to 17.04 cases per 100,000 people in 2021 (95% UI: 11.87 to 24.44). The number of deaths in 2021 was 1,547.49 (95% UI: 621.96 to 3,239.11), a decrease of 79.86% compared to 7,683.74 deaths in 1990 (95% UI: 3,216.6 to 17,549.96). The mortality rate decreased from 0.73 deaths per 100,000 people in 1990 (95% UI: 0.3 to 1.66) to 0.12 deaths per 100,000 people in 2021 (95% UI: 0.05 to 0.25). The DALYs fell from 595,387.26 in 1990 (95% UI: 251,519.07 to 1,352,699.32) to 120,133.62 in 2021 (95% UI: 49,073.35 to 249,877.15). From 1990 to 2021, the EAPC values for global adolescent pertussis were -2.44 (95% CI: -3.13 to -1.75) for prevalence, -2.44 (95% CI: -3.13 to -1.75) for incidence, -3.25 (95% CI: -3.99 to -2.5) for mortality, and -3.24 (95% CI: -3.98 to -2.49) for DALYs.

**SDI regional level**

From 1990 to 2021, the prevalence, incidence, mortality, and DALY rates of pertussis among adolescents decreased across all five SDI regions. Regions with High SDI and High-middle SDI experienced a greater relative reduction, but the Low SDI region had the highest prevalence, incidence, mortality, and DALY rates in 2021 (Fig.1, Table 1, and S. Table 1-3). In 1990, the prevalence cases in the High-middle SDI region was 13,007.26 (95% UI: 10,069.25 to 16,537.23), which decreased to 670.68 (95% UI: 449.2 to 949.68) by 2021, showing the highest reduction of 94.84%. The prevalence rate decreased from 6.99 per 100,000 people (95% UI: 5.41 to 8.88) in 1990 to 0.44 per 100,000 people (95% UI: 0.3 to 0.63) in 2021, with an EAPC of -4.26 (95% CI: -5.35 to -3.15). Other regions showed similar downward trends, with the Low SDI region exhibiting the lowest reduction. The prevalence cases in the Low SDI region decreased from 22,812.56 (95% UI: 17,247.98 to 28,924.42) in 1990 to 13,550.26 (95% UI: 9,734.77 to 18,856.16) in 2021, representing a 40.60% decrease. The prevalence rate decreased from 20.18 per 100,000 people (95% UI: 15.26 to 25.59) in 1990 to 5.11 per 100,000 people (95% UI: 3.67 to 7.11) in 2021, with an EAPC of -2.69 (95% CI: -3.25 to -2.13). Similarly, the incidence cases in the High-middle SDI region decreased from 120,721.78 (95% UI: 73,505.55 to 120,721.78) in 1990 to 6932.67 (95% UI: 3,279.15 to 6,932.67) in 2021, showing the highest reduction of 94.26%. The incidence rate decreased from 50.99 per 100,000 people (95% UI: 39.48 to 64.83) to 2.17 per 100,000 people (95% UI: 2.17 to 4.59), with an EAPC of -4.26 (95% CI: -5.35 to -3.15). The number of incidences in the Low SDI region decreased from 211,148.28 (95% UI: 125,910.22 to 211,148.28) in 1990 to 137,650.00 (95% UI: 71,063.85 to 137,650.0) in 2021, showing the lowest reduction of 34.81%. The incidence rate decreased from 147.32 (95% UI: 111.39 to 186.79) in 1990 to 26.8 (95% UI: 26.8 to 51.92) in 2021, with an EAPC of -2.69 (95% CI: -3.25 to -2.13). Mortality rate decreased significantly across all SDI regions. In 2021, the number of deaths in the High SDI region was only 1.1 (95% UI: 0.27 to 2.9), representing the highest reduction of 97.05%, with an EAPC of -7.83 (95% CI: -8.52 to -7.13). In contrast, the Low SDI region had the lowest reduction of 62.53% and the highest number of deaths in 2021 among the five SDI regions at 820.45 (95% UI: 316.68 to 1,714.48), with an EAPC of -3.65 (95% CI: -4.39 to -2.91). Similarly, DALYs decreased significantly across all SDI regions. The High SDI region experienced the highest reduction of 96.77%, with an EAPC of -7.03(95% CI: -7.83 to -6.22). While the Low SDI region had the lowest reduction of 62.43% and the highest DALYs among the five SDI regions in 2021, at 24.00 years (95% UI: 9.35 to 49.88), with an EAPC of -3.65 (95% CI: -4.38 to -2.9).

**GBD regional level**

From 1990 to 2021, the prevalence, incidence, mortality, and DALY rates have all declined across the 21 GBD regions. However, in the Central Sub-Saharan Africa region, the prevalence cases, incidence cases, deaths, and DALYs had all increased. In contrast, in the other 20 GBD regions, there has been a significant decrease in the prevalence cases, incidence cases, deaths, and DALYs among adolescents (see Table 1, S. Table 1-3). In 2021, Central Sub-Saharan Africa had the highest prevalence and incidence rates, with a prevalence rate of 11.32 per 100,000 (95% UI: 5.76 to 19.84) and an incidence rate of 82.66 per 100,000 (95% UI: 42.04 to 144.84). South Asia had the highest prevalence, incidence, deaths, and DALYs, with 10,627.12 prevalence cases (95% UI: 4,699.18 to 19,383.87), 77,578.00 incidence cases (95% UI: 34,304.03 to 141,502.23), 514.31 deaths (95% UI: 90.99 to 1,480.07), and 39,875.50 DALYs (95% UI: 7,375.13 to 114,284.81). The High-income Asia Pacific region had the lowest prevalence and incidence rates, with a prevalence rate of 0.0021 per 100,000 (95% UI: 0.00033 to 0.0083), 0.35 prevalence cases (95% UI: 0.05 to 1.36), an incidence rate of 0.015 per 100,000 (95% UI: 0.0024 to 0.06), and 2.54 incidence cases (95% UI: 0.39 to 9.96). Central Sub-Saharan Africa had the highest mortality and DALY rates, with a mortality rate of 0.72 per 100,000 (95% UI: 0.14 to 2.18) and a DALY rate of 55.61 years per 100,000 (95% UI: 10.82 to 168.47). Australasia had the lowest deaths, mortality, DALYs, and DALY rates, with 0.0000045 deaths (95% UI: 0.00000016 to 0.000031), a mortality rate of 0.00000012 per 100,000 (95% UI: 0.0000000041 to 0.00000081), 0.05 DALYs (95% UI: 0.01 to 0.12), and a DALY rate of 0.0013 years per 100,000 (95% UI: 0.00031 to 0.0032).

From 1990 to 2021, the High-income Asia Pacific region experienced the fastest decline in prevalence, incidence, mortality, and DALY rates of adolescent pertussis, with EAPC values for prevalence of -9.98 (95% CI: -13.56 to -6.25), incidence of -9.98 (95% CI: -13.56 to -6.25), mortality of -11.56 (95% CI: -13.35 to -9.73), and DALYs of -11.31 (95% CI: -13.2 to -9.39). The High-income North America region saw the slowest decline, with EAPC values for prevalence of -0.65 (95% CI: -2.01 to 0.73), incidence of -0.65 (95% CI: -2.01 to 0.73), mortality of -0.07 (95% CI: -1.61 to 1.48), and DALYs of -0.57 (95% CI: -1.89 to 0.76). As the SDI increased, the prevalence, incidence, mortality, and DALY rates decreased (Fig. 2). The Western, Eastern, and Central Sub-Saharan Africa regions, Andean Latin America, and South Asia had higher prevalence and incidence rates than the global level, while the other regions were below. Western, Eastern, and Central Sub-Saharan Africa, and South Asia had higher mortality and DALY rates than the global level, while the other regions were below (see S. Fig. 1，Table 1, and S. Table 1-3).

**Countries level**

From 1990 to 2021, the burden of adolescent pertussis varied significantly across countries. In 2021, India had the highest prevalence (8,669.62 cases, 95% UI: 3,255.64 to 17,254.14), incidence (63,288.20 cases, 95% UI: 23,766.18 to 125,955.22), deaths (420.50, 95% UI: 35.03 to 1,356.9), and DALYs (32,593.3, 95% UI: 3,142.11 to 104,374.42). Niue had the lowest prevalence (0.00016 cases, 95% UI: 0.00008 to 0.00029) and incidence (0.0012 cases, 95% UI: 0.00059 to 0.00215). Angola had the highest prevalence (12.85 per 100,000, 95% UI: 6.5 to 22.6) and incidence rates (93.77 per 100,000, 95% UI: 47.47 to 164.99), while Korea had the lowest rates (prevalence: 0.00051 per 100,000, 95% UI: 0.00038 to 0.00066; incidence: 0.0037 per 100,000, 95% UI: 0.0028 to 0.0048). Kiribati had the highest mortality (1.64 per 100,000, 95% UI: 0.15 to 5.03) and DALYs rates (125.68 per 100,000, 95% UI: 11.49 to 385.16), while Bermuda had the lowest with nearly zero deaths (0.000000000012, 95% UI: 0 to 0) and DALYs (0.000011 years, 95% UI: 0.0000027 to 0.000049). Latvia had the lowest DALYs rate (0.000078, 95% UI: 0.0000042 to 0.00041) (Fig. 2, S. Fig. 2, S. Table 4-7).

From 1990 to 2021, most countries saw a significant decrease in burden, though some saw increases. Iceland had the fastest rise in prevalence and incidence (EAPC: 5.38, 95% CI: 4.48 to 6.29), while Nicaragua had the fastest decline across all metrics (prevalence and incidence EAPC: -16.72, 95% CI: -18.85 to -14.53; mortality EAPC: -18.74, 95% CI: -20.81 to -16.62; DALYs EAPC: -18.72, 95% CI: -20.79 to -16.6). Spain had the fastest rise in mortality (EAPC: 15.54, 95% CI: 11.21 to 20.05), and Ukraine saw the highest increase in DALYs (EAPC: 3.3, 95% CI: 0.04 to 6.67) ( Fig. 2, S. Fig. 3, S.S. Table 8-11).

**Decomposition analysis of Global,SDI regions and 21 GBD regions Adolescent Pertussis Trends, 1990 to 2021**

We used decomposition methods to analyze the contributions of population aging, population growth, and epidemiological changes to the variation in prevalence, incidence, deaths, and DALYs of adolescent pertussis globally, across five SDI regions, and 21 GBD regions (Fig. 3, S. Table 12-15). Globally, the overall burden of adolescent pertussis has declined, mainly due to epidemiological changes, while population growth has contributed to an increased burden. Among SDI regions, epidemiological changes were the main driver of burden reduction. The Low SDI region saw the slowest reduction, while the High-middle SDI region experienced the fastest decline in prevalence and incidence, and the High SDI region saw the fastest decline in deaths and DALYs. Among GBD regions, Central Sub-Saharan Africa was the only region to see an increase, driven by population growth, while other regions, especially high-income Asia Pacific, saw declines mainly due to epidemiological changes.

**Future burden of Pertussis Among Adolescents Worldwide.**

We predicted future trends in the global burden of adolescent pertussis (Fig. 4, S. Table 16-19). The burden is expected to increase until 2022, followed by a gradual decline from 2023 onward. In 2022, the prevalence rate was 4.79 per 100,000 people (61,612 cases), an increase of 51.1% from 2021. The incidence rate was 35.02 per 100,000 people (449,961 cases), up 51.12%, the mortality rate was 0.24 per 100,000 (3,045 deaths), up 49.2%, and the DALYs rate was 18.48 years per 100,000 (237,412 DALYs), up 49.4%. Despite the expected decline over the next 20 years, the burden will remain higher than in 2021. By 2046, the predicted prevalence rate is 3.59 per 100,000 people (45,936 cases), incidence rate is 26.31 per 100,000 (336,210 cases), mortality rate is 0.16 per 100,000 (2,053 deaths), and DALYs rate is 12.70 years per 100,000 (162,069 DALYs), all showing increases compared to 2021.
