# Supplementary material for: Trends in adolescent pertussis burden: a systematic analysis from 1990 to 2021 in global, regional, and national within the global burden of disease study 2021, with forecasts for 2046
Source: Front Pediatr. 2025 Sep 24;13:1601110. doi: 10.3389/fped.2025.1601110 (PMC12504105; doi:10.3389/fped.2025.1601110)
Supplement: Supplementary file 5 [file Table1.docx]

Sup_Table 1 Global and Regional Incidence of Adolescent Pertussis from 1990 to 2021

| Location | 1990 |  | 2021 |  | EAPC_95%CI |
| --- | --- | --- | --- | --- | --- |
|  | Number(95%UI) | Rate(95%UI) | Number(95%UI) | Rate(95%UI) |  |
| Global | 875091.54 | 82.94 | 219933.64 | 17.04 | -2.44 |
|  | (667644.34 to 1114254.13) | (63.28 to 105.61) | (153155.0 to 315402.68) | (11.87 to 24.44) | (-3.13 to -1.75) |
| High SDI | 49512.05 | 30.69 | 4078.90 | 1.23 | -3.70 |
|  | (30136.62 to 49512.05) | (23.73 to 38.99) | (1478.98 to 4078.9) | (1.23 to 3.39) | (-4.98 to -2.4) |
| High-middle SDI | 120721.78 | 50.99 | 6932.67 | 2.17 | -4.26 |
|  | (73505.55 to 120721.78) | (39.48 to 64.83) | (3279.15 to 6932.67) | (2.17 to 4.59) | (-5.35 to -3.15) |
| Middle SDI | 334034.22 | 70.99 | 58233.38 | 6.40 | -2.40 |
|  | (202357.35 to 334034.22) | (54.61 to 90.15) | (24020.04 to 58233.38) | (6.4 to 15.51) | (-3.34 to -1.45) |
| Low-middle SDI | 395589.72 | 120.81 | 118664.95 | 12.21 | -3.25 |
|  | (235639.82 to 395589.72) | (91.53 to 153.67) | (46142.04 to 118664.95) | (12.21 to 31.4) | (-3.94 to -2.56) |
| Low SDI | 211148.28 | 147.32 | 137650.00 | 26.80 | -2.69 |
|  | (125910.22 to 211148.28) | (111.39 to 186.79) | (71063.85 to 137650.0) | (26.8 to 51.92) | (-3.25 to -2.13) |
| Andean Latin America | 7204.48 | 82.52 | 2672.40 | 23.39 | -2.80 |
|  | (5527.3 to 9142.56) | (63.31 to 104.72) | (876.71 to 5762.23) | (7.67 to 50.43) | (-3.52 to -2.07) |
| Australasia | 967.75 | 30.21 | 7.24 | 0.19 | -5.19 |
|  | (747.98 to 1233.05) | (23.35 to 38.49) | (1.79 to 16.5) | (0.05 to 0.44) | (-8.47 to -1.79) |
| Caribbean | 5403.44 | 74.50 | 22.69 | 0.30 | -4.18 |
|  | (4148.28 to 6869.05) | (57.19 to 94.71) | (6.57 to 106.33) | (0.09 to 1.41) | (-7.3 to -0.95) |
| Central Asia | 9257.23 | 66.98 | 548.17 | 3.61 | -4.44 |
|  | (7090.72 to 11806.99) | (51.3 to 85.43) | (305.29 to 946.07) | (2.01 to 6.22) | (-5.75 to -3.11) |
| Central Europe | 5010.84 | 24.70 | 39.19 | 0.32 | -2.94 |
|  | (3875.27 to 6435.59) | (19.1 to 31.72) | (12.23 to 87.19) | (0.1 to 0.72) | (-5.48 to -0.34) |
| Central Latin America | 35201.38 | 91.59 | 3044.30 | 6.97 | -2.45 |
|  | (26864.03 to 44838.97) | (69.9 to 116.66) | (1756.17 to 4826.43) | (4.02 to 11.05) | (-3.72 to -1.17) |
| Central Sub-Saharan Africa | 17701.78 | 142.25 | 26927.36 | 82.66 | -1.46 |
|  | (13380.92 to 22449.29) | (107.53 to 180.4) | (13692.62 to 47180.56) | (42.04 to 144.84) | (-1.83 to -1.09) |
| East Asia | 105073.25 | 44.46 | 5144.68 | 3.09 | -5.19 |
|  | (81306.63 to 133369.54) | (34.4 to 56.43) | (2707.06 to 8853.0) | (1.63 to 5.32) | (-6.27 to -4.1) |
| Eastern Europe | 29381.61 | 90.98 | 547.26 | 2.36 | -4.37 |
|  | (22528.76 to 37399.03) | (69.76 to 115.81) | (264.78 to 975.43) | (1.14 to 4.21) | (-6.18 to -2.53) |
| Eastern Sub-Saharan Africa | 54803.59 | 120.69 | 35033.82 | 33.58 | -2.69 |
|  | (41575.14 to 69786.33) | (91.56 to 153.68) | (22810.47 to 50925.16) | (21.86 to 48.81) | (-3.33 to -2.05) |
| High-income Asia Pacific | 13413.29 | 47.53 | 2.54 | 0.015 | -9.98 |
|  | (10401.13 to 16975.18) | (36.86 to 60.15) | (0.39 to 9.96) | (0.0024 to 0.06) | (-13.56 to -6.25) |
| High-income North America | 4597.99 | 11.63 | 1620.74 | 3.43 | -0.65 |
|  | (3505.2 to 5982.21) | (8.86 to 15.13) | (751.75 to 2982.55) | (1.59 to 6.32) | (-2.01 to 0.73) |
| North Africa and Middle East | 59779.46 | 76.59 | 11896.50 | 10.61 | -2.26 |
|  | (45926.69 to 75755.74) | (58.84 to 97.06) | (5931.47 to 21779.86) | (5.29 to 19.42) | (-3.15 to -1.35) |
| Oceania | 1626.87 | 110.15 | 132.44 | 4.76 | -2.09 |
|  | (1234.8 to 2070.79) | (83.61 to 140.21) | (63.71 to 232.95) | (2.29 to 8.37) | (-3.95 to -0.2) |
| South Asia | 300786.41 | 127.52 | 77578.00 | 21.88 | -3.58 |
|  | (227639.59 to 382399.13) | (96.51 to 162.12) | (34304.03 to 141502.23) | (9.67 to 39.91) | (-4.37 to -2.77) |
| Southeast Asia | 100215.66 | 96.54 | 4253.72 | 3.71 | -2.81 |
|  | (76468.59 to 127693.7) | (73.67 to 123.02) | (2574.85 to 6741.19) | (2.24 to 5.88) | (-4.48 to -1.1) |
| Southern Latin America | 4894.07 | 53.01 | 223.53 | 2.22 | -4.46 |
|  | (3790.57 to 6182.42) | (41.06 to 66.97) | (93.33 to 435.76) | (0.93 to 4.33) | (-6.18 to -2.7) |
| Southern Sub-Saharan Africa | 7866.33 | 65.03 | 1841.19 | 12.21 | -1.32 |
|  | (6085.08 to 9986.64) | (50.3 to 82.55) | (737.59 to 3707.1) | (4.89 to 24.58) | (-3.0 to 0.4) |
| Tropical Latin America | 29140.89 | 87.01 | 3690.89 | 11.30 | -4.81 |
|  | (22301.78 to 37091.4) | (66.59 to 110.75) | (1060.21 to 8263.33) | (3.25 to 25.29) | (-6.88 to -2.7) |
| Western Europe | 21126.42 | 40.72 | 363.76 | 0.77 | -5.93 |
|  | (16351.83 to 26796.18) | (31.52 to 51.65) | (217.42 to 574.35) | (0.46 to 1.21) | (-7.5 to -4.34) |
| Western Sub-Saharan Africa | 61638.78 | 142.66 | 44343.23 | 37.88 | -2.12 |
|  | (46625.08 to 78292.4) | (107.91 to 181.2) | (28122.78 to 64837.28) | (24.02 to 55.38) | (-2.77 to -1.46) |

Sup_Table 2 Global and Regional Deaths of Adolescent Pertussis from 1990 to 2021

| Location | 1990 |  | 2021 |  | EAPC_95%CI |
| --- | --- | --- | --- | --- | --- |
|  | Number(95%UI) | Rate(95%UI) | Number(95%UI) | Rate(95%UI) |  |
| Global | 7683.74 | 0.73 | 1547.49 | 0.12 | -3.25 |
|  | (3216.6 to 17549.96) | (0.3 to 1.66) | (621.96 to 3239.11) | (0.05 to 0.25) | (-3.99 to -2.5) |
| High SDI | 37.25 | 0.03 | 1.10 | 0.00091 | -7.83 |
|  | (10.21 to 86.61) | (0.01 to 0.07) | (0.27 to 2.9) | (0.00022 to 0.0024) | (-8.52 to -7.13) |
| High-middle SDI | 347.04 | 0.19 | 17.86 | 0.01 | -6.91 |
|  | (109.4 to 872.02) | (0.06 to 0.47) | (6.01 to 40.18) | (0.004 to 0.027) | (-7.43 to -6.38) |
| Middle SDI | 1928.98 | 0.52 | 189.88 | 0.05 | -4.39 |
|  | (707.82 to 4722.5) | (0.19 to 1.27) | (69.03 to 431.55) | (0.02 to 0.11) | (-5.2 to -3.58) |
| Low-middle SDI | 3175.49 | 1.23 | 517.73 | 0.14 | -4.47 |
|  | (1124.65 to 8023.78) | (0.44 to 3.12) | (160.08 to 1303.79) | (0.04 to 0.34) | (-5.2 to -3.73) |
| Low SDI | 2189.85 | 1.94 | 820.45 | 0.31 | -3.65 |
|  | (921.81 to 4680.85) | (0.82 to 4.14) | (316.68 to 1714.48) | (0.12 to 0.65) | (-4.39 to -2.91) |
| Andean Latin America | 57.40 | 0.66 | 6.35 | 0.06 | -7.05 |
|  | (13.6 to 148.22) | (0.16 to 1.7) | (0.58 to 24.71) | (0.01 to 0.22) | (-8.2 to -5.89) |
| Australasia | 0.00083 | 0.000026 | 0.0000045 | 0.00000012 | -3.85 |
|  | (0.00024 to 0.0017) | (0.000008 to 0.000056) | (0.00000016 to 0.000031) | (0.0000000041 to 0.00000081) | (-7.18 to -0.4) |
| Caribbean | 50.88 | 0.70 | 0.41 | 0.01 | -4.10 |
|  | (9.11 to 138.53) | (0.13 to 1.91) | (0.01 to 1.76) | (0.000087 to 0.02) | (-6.78 to -1.34) |
| Central Asia | 21.72 | 0.16 | 1.45 | 0.01 | -5.70 |
|  | (5.72 to 57.84) | (0.04 to 0.42) | (0.31 to 4.03) | (0.002 to 0.027) | (-6.82 to -4.57) |
| Central Europe | 7.21 | 0.04 | 0.14 | 0.00119 | -5.12 |
|  | (2.66 to 14.66) | (0.01 to 0.07) | (0.03 to 0.41) | (0.00023 to 0.00335) | (-6.54 to -3.68) |
| Central Latin America | 33.92 | 0.09 | 1.95 | 0.0045 | -5.93 |
|  | (15.23 to 68.32) | (0.04 to 0.18) | (0.45 to 6.58) | (0.001 to 0.0151) | (-7.01 to -4.84) |
| Central Sub-Saharan Africa | 224.99 | 1.81 | 233.58 | 0.72 | -2.06 |
|  | (44.92 to 584.65) | (0.36 to 4.7) | (44.75 to 711.59) | (0.14 to 2.18) | (-2.42 to -1.7) |
| East Asia | 862.38 | 0.36 | 43.09 | 0.026 | -9.16 |
|  | (97.49 to 2596.53) | (0.04 to 1.1) | (4.28 to 119.79) | (0.0026 to 0.072) | (-9.75 to -8.56) |
| Eastern Europe | 0.11 | 0.00034 | 0.0026 | 0.000011 | -8.21 |
|  | (0.06 to 0.15) | (0.00018 to 0.00048) | (0.00026 to 0.0072) | (0.0000011 to 0.000031) | (-9.35 to -7.05) |
| Eastern Sub-Saharan Africa | 805.81 | 1.77 | 329.73 | 0.32 | -3.84 |
|  | (272.74 to 1884.92) | (0.6 to 4.15) | (117.5 to 727.57) | (0.11 to 0.7) | (-4.65 to -3.04) |
| High-income Asia Pacific | 21.80 | 0.08 | 0.02 | 0.00013 | -11.56 |
|  | (2.45 to 70.29) | (0.01 to 0.25) | (0.00017 to 0.98) | (0.000001 to 0.00059) | (-13.35 to -9.73) |
| High-income North America | 0.10 | 0.00024 | 0.03 | 0.00007 | -0.07 |
|  | (0.06 to 0.15) | (0.00014 to 0.00037) | (0.01 to 0.06) | (0.00003 to 0.00013) | (-1.61 to 1.48) |
| North Africa and Middle East | 527.54 | 0.68 | 57.62 | 0.05 | -3.86 |
|  | (219.72 to 1087.48) | (0.28 to 1.39) | (17.17 to 133.45) | (0.02 to 0.12) | (-4.87 to -2.83) |
| Oceania | 16.39 | 1.11 | 1.68 | 0.06 | -1.97 |
|  | (3.87 to 46.27) | (0.26 to 3.13) | (0.57 to 3.77) | (0.02 to 0.14) | (-3.8 to -0.1) |
| South Asia | 3297.25 | 1.40 | 514.31 | 0.15 | -5.06 |
|  | (753.09 to 9696.96) | (0.32 to 4.11) | (90.99 to 1480.07) | (0.03 to 0.42) | (-5.9 to -4.22) |
| Southeast Asia | 944.22 | 0.91 | 32.19 | 0.03 | -4.38 |
|  | (295.06 to 2235.89) | (0.28 to 2.15) | (6.43 to 96.42) | (0.01 to 0.08) | (-5.87 to -2.87) |
| Southern Latin America | 0.06 | 0.00063 | 0.0039 | 0.000039 | -2.37 |
|  | (0.02 to 0.11) | (0.00025 to 0.00116) | (0.00093 to 0.01) | (0.000009 to 0.000103) | (-5.17 to 0.52) |
| Southern Sub-Saharan Africa | 58.76 | 0.49 | 15.70 | 0.10 | -1.39 |
|  | (12.91 to 154.82) | (0.11 to 1.28) | (2.34 to 46.79) | (0.02 to 0.31) | (-3.0 to 0.24) |
| Tropical Latin America | 5.27 | 0.02 | 0.39 | 0.00121 | -5.26 |
|  | (0.77 to 15.69) | (0.0 to 0.05) | (0.04 to 1.29) | (0.00013 to 0.00396) | (-6.35 to -4.16) |
| Western Europe | 0.21 | 0.00040 | 0.0037 | 0.000008 | -4.44 |
|  | (0.1 to 0.41) | (0.00018 to 0.00079) | (0.00099 to 0.0078) | (0.0000021 to 0.000016) | (-6.25 to -2.59) |
| Western Sub-Saharan Africa | 747.74 | 1.73 | 308.84 | 0.26 | -3.17 |
|  | (298.23 to 1592.02) | (0.69 to 3.68) | (76.08 to 808.64) | (0.06 to 0.69) | (-4.01 to -2.32) |

Sup_Table 3 Global and Regional DALYs of Adolescent Pertussis from 1990 to 2021

| Location | 1990 |  | 2021 |  | EAPC_95%CI |
| --- | --- | --- | --- | --- | --- |
|  | Number(95%UI) | Rate(95%UI) | Number(95%UI) | Rate(95%UI) |  |
| Global | 595387.26 | 56.43 | 120133.62 | 9.31 | -3.24 |
|  | (251519.07 to 1352699.32) | (23.84 to 128.81) | (49073.35 to 249877.15) | (3.8 to 19.36) | (-3.98 to -2.49) |
| High SDI | 3117.55 | 2.46 | 100.76 | 0.08 | -7.03 |
|  | (1057.89 to 6895.73) | (0.83 to 5.43) | (34.28 to 246.54) | (0.03 to 0.21) | (-7.83 to -6.22) |
| High-middle SDI | 27194.60 | 14.60 | 1399.83 | 0.93 | -6.81 |
|  | (9066.36 to 67376.87) | (4.87 to 36.18) | (490.47 to 3099.78) | (0.33 to 2.05) | (-7.36 to -6.27) |
| Middle SDI | 149494.85 | 40.35 | 14783.27 | 3.94 | -4.36 |
|  | (55841.29 to 363965.73) | (15.07 to 98.23) | (5474.94 to 33298.84) | (1.46 to 8.87) | (-5.17 to -3.55) |
| Low-middle SDI | 245814.94 | 95.49 | 40182.45 | 10.63 | -4.45 |
|  | (87945.68 to 618254.17) | (34.16 to 240.16) | (12654.05 to 100404.43) | (3.35 to 26.57) | (-5.19 to -3.72) |
| Low SDI | 169366.79 | 149.83 | 63630.10 | 24.00 | -3.65 |
|  | (71608.03 to 360897.35) | (63.35 to 319.27) | (24783.72 to 132237.52) | (9.35 to 49.88) | (-4.38 to -2.9) |
| Andean Latin America | 4456.30 | 51.04 | 504.38 | 4.41 | -6.96 |
|  | (1105.28 to 11433.5) | (12.66 to 130.96) | (54.27 to 1932.55) | (0.47 to 16.91) | (-8.09 to -5.82) |
| Australasia | 6.74 | 0.21 | 0.05 | 0.0013 | -5.17 |
|  | (3.91 to 10.72) | (0.12 to 0.33) | (0.01 to 0.12) | (0.00031 to 0.0032) | (-8.45 to -1.78) |
| Caribbean | 3943.55 | 54.37 | 31.69 | 0.42 | -4.10 |
|  | (733.06 to 10674.08) | (10.11 to 147.17) | (0.56 to 135.67) | (0.01 to 1.8) | (-6.79 to -1.34) |
| Central Asia | 1727.28 | 12.50 | 115.32 | 0.76 | -5.65 |
|  | (497.9 to 4523.53) | (3.6 to 32.73) | (26.8 to 315.21) | (0.18 to 2.07) | (-6.77 to -4.52) |
| Central Europe | 586.61 | 2.89 | 11.34 | 0.09 | -4.91 |
|  | (231.66 to 1164.64) | (1.14 to 5.74) | (2.43 to 31.6) | (0.02 to 0.26) | (-6.4 to -3.4) |
| Central Latin America | 2840.68 | 7.39 | 169.38 | 0.39 | -5.38 |
|  | (1376.4 to 5549.46) | (3.58 to 14.44) | (52.03 to 521.31) | (0.12 to 1.19) | (-6.47 to -4.28) |
| Central Sub-Saharan Africa | 17394.07 | 139.78 | 18113.63 | 55.61 | -2.05 |
|  | (3556.53 to 45005.3) | (28.58 to 361.66) | (3526.05 to 54878.5) | (10.82 to 168.47) | (-2.41 to -1.69) |
| East Asia | 66600.96 | 28.18 | 3331.42 | 2.00 | -9.06 |
|  | (8177.43 to 199223.73) | (3.46 to 84.29) | (362.58 to 9229.91) | (0.22 to 5.54) | (-9.65 to -8.47) |
| Eastern Europe | 209.25 | 0.65 | 3.98 | 0.017 | -4.57 |
|  | (124.37 to 336.56) | (0.39 to 1.04) | (1.73 to 7.73) | (0.01 to 0.03) | (-6.3 to -2.81) |
| Eastern Sub-Saharan Africa | 62266.39 | 137.12 | 25516.04 | 24.46 | -3.84 |
|  | (21245.35 to 145222.98) | (46.79 to 319.8) | (9207.38 to 56048.75) | (8.83 to 53.73) | (-4.64 to -3.03) |
| High-income Asia Pacific | 1758.22 | 6.23 | 1.73 | 0.0105 | -11.31 |
|  | (278.11 to 5470.09) | (0.99 to 19.38) | (0.03 to 7.57) | (0.00015 to 0.0458) | (-13.2 to -9.39) |
| High-income North America | 39.31 | 0.10 | 13.62 | 0.029 | -0.57 |
|  | (24.38 to 61.35) | (0.06 to 0.16) | (6.1 to 27.64) | (0.01 to 0.06) | (-1.89 to 0.76) |
| North Africa and Middle East | 40913.11 | 52.42 | 4510.25 | 4.02 | -3.83 |
|  | (17173.11 to 83825.02) | (22.0 to 107.4) | (1361.32 to 10411.31) | (1.21 to 9.28) | (-4.85 to -2.81) |
| Oceania | 1269.13 | 85.93 | 129.56 | 4.65 | -1.97 |
|  | (305.44 to 3564.93) | (20.68 to 241.38) | (44.75 to 289.58) | (1.61 to 10.4) | (-3.8 to -0.1) |
| South Asia | 255032.10 | 108.12 | 39875.50 | 11.25 | -5.05 |
|  | (59767.77 to 745780.24) | (25.34 to 316.18) | (7375.13 to 114284.81) | (2.08 to 32.23) | (-5.89 to -4.21) |
| Southeast Asia | 73073.90 | 70.40 | 2480.07 | 2.16 | -4.37 |
|  | (23187.94 to 172111.73) | (22.34 to 165.81) | (510.44 to 7386.96) | (0.45 to 6.44) | (-5.86 to -2.85) |
| Southern Latin America | 37.97 | 0.41 | 1.86 | 0.018 | -4.03 |
|  | (17.06 to 69.14) | (0.18 to 0.75) | (0.74 to 4.04) | (0.01 to 0.04) | (-5.86 to -2.17) |
| Southern Sub-Saharan Africa | 4554.00 | 37.65 | 1221.01 | 8.10 | -1.38 |
|  | (1033.05 to 11919.71) | (8.54 to 98.53) | (189.03 to 3625.28) | (1.25 to 24.04) | (-2.98 to 0.25) |
| Tropical Latin America | 603.37 | 1.80 | 55.84 | 0.17 | -4.98 |
|  | (221.43 to 1404.79) | (0.66 to 4.19) | (16.48 to 131.91) | (0.05 to 0.4) | (-6.08 to -3.86) |
| Western Europe | 160.07 | 0.31 | 2.80 | 0.0059 | -5.77 |
|  | (99.6 to 251.97) | (0.19 to 0.49) | (1.51 to 4.91) | (0.0032 to 0.0103) | (-7.34 to -4.17) |
| Western Sub-Saharan Africa | 57914.25 | 134.04 | 24044.15 | 20.54 | -3.15 |
|  | (23263.41 to 122823.86) | (53.84 to 284.27) | (6055.4 to 62573.45) | (5.17 to 53.45) | (-3.99 to -2.31) |

Sup_Table 4 Prevalence of Adolescent Pertussis in 204 Countries and Territories in 2021

| measure | location | sex | age | cause | metric | year | val | upper | lower |
| --- | --- | --- | --- | --- | --- | --- | --- | --- | --- |
| Prevalence | Timor-Leste | Both | 10 to 19 | Pertussis | Number | 2021 | 1.644513301 | 2.861281525 | 0.795591281 |
| Prevalence | Timor-Leste | Both | 10 to 19 | Pertussis | Rate | 2021 | 0.496980656 | 0.864694477 | 0.240431911 |
| Prevalence | Thailand | Both | 10 to 19 | Pertussis | Number | 2021 | 3.73080776 | 10.51908123 | 0.605605382 |
| Prevalence | Thailand | Both | 10 to 19 | Pertussis | Rate | 2021 | 0.049336192 | 0.139104302 | 0.008008524 |
| Prevalence | Philippines | Both | 10 to 19 | Pertussis | Number | 2021 | 243.2912002 | 457.600061 | 103.8076538 |
| Prevalence | Philippines | Both | 10 to 19 | Pertussis | Rate | 2021 | 1.098636435 | 2.066396563 | 0.468766937 |
| Prevalence | Sri Lanka | Both | 10 to 19 | Pertussis | Number | 2021 | 1.262653129 | 3.851297539 | 0.266138636 |
| Prevalence | Sri Lanka | Both | 10 to 19 | Pertussis | Rate | 2021 | 0.034880168 | 0.106390191 | 0.007351948 |
| Prevalence | Serbia | Both | 10 to 19 | Pertussis | Number | 2021 | 0.825124483 | 1.760484287 | 0.303693428 |
| Prevalence | Serbia | Both | 10 to 19 | Pertussis | Rate | 2021 | 0.076147179 | 0.1624675 | 0.028026556 |
| Prevalence | Romania | Both | 10 to 19 | Pertussis | Number | 2021 | 0.918722306 | 3.151556071 | 0.123630074 |
| Prevalence | Romania | Both | 10 to 19 | Pertussis | Rate | 2021 | 0.043358655 | 0.148736163 | 0.005834662 |
| Prevalence | Azerbaijan | Both | 10 to 19 | Pertussis | Number | 2021 | 6.349072771 | 15.51552638 | 1.386373151 |
| Prevalence | Azerbaijan | Both | 10 to 19 | Pertussis | Rate | 2021 | 0.426916469 | 1.043275763 | 0.093220782 |
| Prevalence | Viet Nam | Both | 10 to 19 | Pertussis | Number | 2021 | 49.47083253 | 114.30627 | 13.76393364 |
| Prevalence | Viet Nam | Both | 10 to 19 | Pertussis | Rate | 2021 | 0.335373292 | 0.774906507 | 0.093308632 |
| Prevalence | Myanmar | Both | 10 to 19 | Pertussis | Number | 2021 | 43.21639444 | 75.62416729 | 20.9554337 |
| Prevalence | Myanmar | Both | 10 to 19 | Pertussis | Rate | 2021 | 0.417635412 | 0.730818262 | 0.202509518 |
| Prevalence | Maldives | Both | 10 to 19 | Pertussis | Number | 2021 | 0.160966439 | 0.282587919 | 0.078922373 |
| Prevalence | Maldives | Both | 10 to 19 | Pertussis | Rate | 2021 | 0.252761563 | 0.443740722 | 0.123929823 |
| Prevalence | Lao People's Democratic Republic | Both | 10 to 19 | Pertussis | Number | 2021 | 22.50503193 | 43.68688728 | 9.507650064 |
| Prevalence | Lao People's Democratic Republic | Both | 10 to 19 | Pertussis | Rate | 2021 | 1.603673407 | 3.113059318 | 0.677500286 |
| Prevalence | Vanuatu | Both | 10 to 19 | Pertussis | Number | 2021 | 0.161256979 | 0.283075069 | 0.079072992 |
| Prevalence | Vanuatu | Both | 10 to 19 | Pertussis | Rate | 2021 | 0.246157663 | 0.432112136 | 0.120704376 |
| Prevalence | Taiwan (Province of China) | Both | 10 to 19 | Pertussis | Number | 2021 | 5.776820809 | 9.89627268 | 3.020758956 |
| Prevalence | Taiwan (Province of China) | Both | 10 to 19 | Pertussis | Rate | 2021 | 0.277737364 | 0.475791924 | 0.145231721 |
| Prevalence | China | Both | 10 to 19 | Pertussis | Number | 2021 | 696.7611078 | 1198.899669 | 366.7614898 |
| Prevalence | China | Both | 10 to 19 | Pertussis | Rate | 2021 | 0.433133767 | 0.745282599 | 0.227993187 |
| Prevalence | Samoa | Both | 10 to 19 | Pertussis | Number | 2021 | 0.26753564 | 0.467420451 | 0.12882902 |
| Prevalence | Samoa | Both | 10 to 19 | Pertussis | Rate | 2021 | 0.595174434 | 1.03984913 | 0.286600091 |
| Prevalence | Micronesia (Federated States of) | Both | 10 to 19 | Pertussis | Number | 2021 | 0.081780174 | 0.14266964 | 0.039658688 |
| Prevalence | Micronesia (Federated States of) | Both | 10 to 19 | Pertussis | Rate | 2021 | 0.376036875 | 0.656015307 | 0.18235629 |
| Prevalence | Tonga | Both | 10 to 19 | Pertussis | Number | 2021 | 0.002583597 | 0.004747291 | 0.001179947 |
| Prevalence | Tonga | Both | 10 to 19 | Pertussis | Rate | 2021 | 0.011635788 | 0.02138045 | 0.005314145 |
| Prevalence | Cambodia | Both | 10 to 19 | Pertussis | Number | 2021 | 1.155018246 | 2.485751298 | 0.370785142 |
| Prevalence | Cambodia | Both | 10 to 19 | Pertussis | Rate | 2021 | 0.036478978 | 0.078507563 | 0.011710519 |
| Prevalence | Georgia | Both | 10 to 19 | Pertussis | Number | 2021 | 0.333587039 | 1.122414295 | 0.069536648 |
| Prevalence | Georgia | Both | 10 to 19 | Pertussis | Rate | 2021 | 0.078556003 | 0.264315968 | 0.0163751 |
| Prevalence | Malaysia | Both | 10 to 19 | Pertussis | Number | 2021 | 68.97951985 | 131.1505231 | 31.01239189 |
| Prevalence | Malaysia | Both | 10 to 19 | Pertussis | Rate | 2021 | 1.316800977 | 2.503629154 | 0.592018443 |
| Prevalence | Hungary | Both | 10 to 19 | Pertussis | Number | 2021 | 0.082261241 | 0.17659982 | 0.029216838 |
| Prevalence | Hungary | Both | 10 to 19 | Pertussis | Rate | 2021 | 0.00854505 | 0.018344658 | 0.003034957 |
| Prevalence | Croatia | Both | 10 to 19 | Pertussis | Number | 2021 | 0.180620457 | 0.383558427 | 0.066353729 |
| Prevalence | Croatia | Both | 10 to 19 | Pertussis | Rate | 2021 | 0.042422357 | 0.090086432 | 0.015584511 |
| Prevalence | Indonesia | Both | 10 to 19 | Pertussis | Number | 2021 | 146.2866658 | 301.6170446 | 53.82536586 |
| Prevalence | Indonesia | Both | 10 to 19 | Pertussis | Rate | 2021 | 0.319887067 | 0.659550147 | 0.117700669 |
| Prevalence | Democratic People's Republic of Korea | Both | 10 to 19 | Pertussis | Number | 2021 | 2.213375944 | 3.976340293 | 1.117864215 |
| Prevalence | Democratic People's Republic of Korea | Both | 10 to 19 | Pertussis | Rate | 2021 | 0.063003227 | 0.113185594 | 0.031819743 |
| Prevalence | North Macedonia | Both | 10 to 19 | Pertussis | Number | 2021 | 0.164126907 | 0.349785365 | 0.060487632 |
| Prevalence | North Macedonia | Both | 10 to 19 | Pertussis | Rate | 2021 | 0.070485335 | 0.150217531 | 0.025976795 |
| Prevalence | Uruguay | Both | 10 to 19 | Pertussis | Number | 2021 | 0.908880203 | 1.621334023 | 0.416096876 |
| Prevalence | Uruguay | Both | 10 to 19 | Pertussis | Rate | 2021 | 0.192927658 | 0.344159963 | 0.088324727 |
| Prevalence | Papua New Guinea | Both | 10 to 19 | Pertussis | Number | 2021 | 16.10479732 | 28.31563616 | 7.719312663 |
| Prevalence | Papua New Guinea | Both | 10 to 19 | Pertussis | Rate | 2021 | 0.767066976 | 1.348665801 | 0.367668695 |
| Prevalence | Czechia | Both | 10 to 19 | Pertussis | Number | 2021 | 0.298382428 | 0.637841388 | 0.107516541 |
| Prevalence | Czechia | Both | 10 to 19 | Pertussis | Rate | 2021 | 0.027214368 | 0.058175176 | 0.00980619 |
| Prevalence | Solomon Islands | Both | 10 to 19 | Pertussis | Number | 2021 | 0.344783752 | 0.605075905 | 0.169018375 |
| Prevalence | Solomon Islands | Both | 10 to 19 | Pertussis | Rate | 2021 | 0.23060543 | 0.404699434 | 0.11304638 |
| Prevalence | Ireland | Both | 10 to 19 | Pertussis | Number | 2021 | 1.416817915 | 4.317203275 | 0.281272996 |
| Prevalence | Ireland | Both | 10 to 19 | Pertussis | Rate | 2021 | 0.207352282 | 0.631825684 | 0.041164497 |
| Prevalence | Marshall Islands | Both | 10 to 19 | Pertussis | Number | 2021 | 0.037829816 | 0.065975238 | 0.018400003 |
| Prevalence | Marshall Islands | Both | 10 to 19 | Pertussis | Rate | 2021 | 0.327497714 | 0.571156351 | 0.159291255 |
| Prevalence | Belarus | Both | 10 to 19 | Pertussis | Number | 2021 | 5.498874903 | 13.33647864 | 1.308483355 |
| Prevalence | Belarus | Both | 10 to 19 | Pertussis | Rate | 2021 | 0.56224445 | 1.363617327 | 0.133788733 |
| Prevalence | Slovenia | Both | 10 to 19 | Pertussis | Number | 2021 | 0.080492149 | 0.247841714 | 0.017103732 |
| Prevalence | Slovenia | Both | 10 to 19 | Pertussis | Rate | 2021 | 0.04023363 | 0.123882539 | 0.008549222 |
| Prevalence | Latvia | Both | 10 to 19 | Pertussis | Number | 2021 | 0.002783559 | 0.015769728 | 0.000119503 |
| Prevalence | Latvia | Both | 10 to 19 | Pertussis | Rate | 2021 | 0.00146406 | 0.008294353 | 6.29E-05 |
| Prevalence | Republic of Moldova | Both | 10 to 19 | Pertussis | Number | 2021 | 1.564739427 | 3.284431939 | 0.514310693 |
| Prevalence | Republic of Moldova | Both | 10 to 19 | Pertussis | Rate | 2021 | 0.433219837 | 0.909340586 | 0.142394056 |
| Prevalence | Brunei Darussalam | Both | 10 to 19 | Pertussis | Number | 2021 | 0.001128991 | 0.005143664 | 0.000169341 |
| Prevalence | Brunei Darussalam | Both | 10 to 19 | Pertussis | Rate | 2021 | 0.001704559 | 0.007765939 | 0.000255672 |
| Prevalence | Haiti | Both | 10 to 19 | Pertussis | Number | 2021 | 1.629936284 | 7.58921015 | 0.470048376 |
| Prevalence | Haiti | Both | 10 to 19 | Pertussis | Rate | 2021 | 0.062853156 | 0.292653039 | 0.018125876 |
| Prevalence | Kiribati | Both | 10 to 19 | Pertussis | Number | 2021 | 0.160797546 | 0.280942206 | 0.077428922 |
| Prevalence | Kiribati | Both | 10 to 19 | Pertussis | Rate | 2021 | 0.646791787 | 1.130061473 | 0.31144997 |
| Prevalence | Republic of Korea | Both | 10 to 19 | Pertussis | Number | 2021 | 0.023495814 | 0.030730227 | 0.017669544 |
| Prevalence | Republic of Korea | Both | 10 to 19 | Pertussis | Rate | 2021 | 0.000507705 | 0.000664029 | 0.000381809 |
| Prevalence | Kyrgyzstan | Both | 10 to 19 | Pertussis | Number | 2021 | 15.65385954 | 35.74385241 | 4.555454988 |
| Prevalence | Kyrgyzstan | Both | 10 to 19 | Pertussis | Rate | 2021 | 1.269383535 | 2.898496541 | 0.369405356 |
| Prevalence | Fiji | Both | 10 to 19 | Pertussis | Number | 2021 | 0.127766768 | 0.227730125 | 0.062263624 |
| Prevalence | Fiji | Both | 10 to 19 | Pertussis | Rate | 2021 | 0.076773758 | 0.136840728 | 0.037413582 |
| Prevalence | Germany | Both | 10 to 19 | Pertussis | Number | 2021 | 1.807180515 | 4.304086383 | 0.592813045 |
| Prevalence | Germany | Both | 10 to 19 | Pertussis | Rate | 2021 | 0.022979564 | 0.054729468 | 0.007538032 |
| Prevalence | Uzbekistan | Both | 10 to 19 | Pertussis | Number | 2021 | 20.08331061 | 36.18315651 | 9.278165585 |
| Prevalence | Uzbekistan | Both | 10 to 19 | Pertussis | Rate | 2021 | 0.371955829 | 0.670135329 | 0.171837594 |
| Prevalence | Finland | Both | 10 to 19 | Pertussis | Number | 2021 | 1.300906808 | 2.977080826 | 0.375639827 |
| Prevalence | Finland | Both | 10 to 19 | Pertussis | Rate | 2021 | 0.21179831 | 0.484693202 | 0.061157248 |
| Prevalence | Israel | Both | 10 to 19 | Pertussis | Number | 2021 | 1.322429115 | 2.307276964 | 0.629022765 |
| Prevalence | Israel | Both | 10 to 19 | Pertussis | Rate | 2021 | 0.084636466 | 0.147667474 | 0.040257934 |
| Prevalence | Bulgaria | Both | 10 to 19 | Pertussis | Number | 2021 | 0.348834971 | 0.741586856 | 0.128354466 |
| Prevalence | Bulgaria | Both | 10 to 19 | Pertussis | Rate | 2021 | 0.052331165 | 0.111250613 | 0.019255348 |
| Prevalence | Iceland | Both | 10 to 19 | Pertussis | Number | 2021 | 1.436316815 | 2.606124709 | 0.681454055 |
| Prevalence | Iceland | Both | 10 to 19 | Pertussis | Rate | 2021 | 3.180549843 | 5.770947919 | 1.508997573 |
| Prevalence | Kazakhstan | Both | 10 to 19 | Pertussis | Number | 2021 | 13.51135388 | 26.63555178 | 5.225292823 |
| Prevalence | Kazakhstan | Both | 10 to 19 | Pertussis | Rate | 2021 | 0.459019997 | 0.904887179 | 0.177518398 |
| Prevalence | Tajikistan | Both | 10 to 19 | Pertussis | Number | 2021 | 9.451060037 | 16.98564465 | 4.369096047 |
| Prevalence | Tajikistan | Both | 10 to 19 | Pertussis | Rate | 2021 | 0.496288495 | 0.891940162 | 0.229427397 |
| Prevalence | Poland | Both | 10 to 19 | Pertussis | Number | 2021 | 1.69641264 | 6.226292865 | 0.175071049 |
| Prevalence | Poland | Both | 10 to 19 | Pertussis | Rate | 2021 | 0.043797796 | 0.160749749 | 0.004519965 |
| Prevalence | Luxembourg | Both | 10 to 19 | Pertussis | Number | 2021 | 0.021472598 | 0.037723851 | 0.010166998 |
| Prevalence | Luxembourg | Both | 10 to 19 | Pertussis | Rate | 2021 | 0.03163201 | 0.055572282 | 0.014977349 |
| Prevalence | Turkmenistan | Both | 10 to 19 | Pertussis | Number | 2021 | 0.403283684 | 0.743870429 | 0.177335673 |
| Prevalence | Turkmenistan | Both | 10 to 19 | Pertussis | Rate | 2021 | 0.044518945 | 0.082116703 | 0.019576287 |
| Prevalence | Armenia | Both | 10 to 19 | Pertussis | Number | 2021 | 8.218606425 | 18.16047527 | 2.448385934 |
| Prevalence | Armenia | Both | 10 to 19 | Pertussis | Rate | 2021 | 2.222680434 | 4.911408452 | 0.662153561 |
| Prevalence | Slovakia | Both | 10 to 19 | Pertussis | Number | 2021 | 0.257736672 | 0.599064155 | 0.075319866 |
| Prevalence | Slovakia | Both | 10 to 19 | Pertussis | Rate | 2021 | 0.046424726 | 0.107906217 | 0.013566964 |
| Prevalence | Lithuania | Both | 10 to 19 | Pertussis | Number | 2021 | 4.648395331 | 8.923972185 | 2.021683612 |
| Prevalence | Lithuania | Both | 10 to 19 | Pertussis | Rate | 2021 | 1.771999142 | 3.401877407 | 0.770679207 |
| Prevalence | Estonia | Both | 10 to 19 | Pertussis | Number | 2021 | 0.113391987 | 0.306733757 | 0.025381261 |
| Prevalence | Estonia | Both | 10 to 19 | Pertussis | Rate | 2021 | 0.08082695 | 0.218642911 | 0.018092019 |
| Prevalence | Japan | Both | 10 to 19 | Pertussis | Number | 2021 | 0.235456895 | 1.29353098 | 0.00917877 |
| Prevalence | Japan | Both | 10 to 19 | Pertussis | Rate | 2021 | 0.002068727 | 0.01136498 | 8.06E-05 |
| Prevalence | Argentina | Both | 10 to 19 | Pertussis | Number | 2021 | 27.13670441 | 54.65367634 | 10.27522288 |
| Prevalence | Argentina | Both | 10 to 19 | Pertussis | Rate | 2021 | 0.383561816 | 0.772498495 | 0.145234406 |
| Prevalence | Bosnia and Herzegovina | Both | 10 to 19 | Pertussis | Number | 2021 | 0.368917943 | 0.789195075 | 0.135672322 |
| Prevalence | Bosnia and Herzegovina | Both | 10 to 19 | Pertussis | Rate | 2021 | 0.10612188 | 0.227017597 | 0.039027112 |
| Prevalence | Ukraine | Both | 10 to 19 | Pertussis | Number | 2021 | 20.07435384 | 33.37040915 | 10.26696016 |
| Prevalence | Ukraine | Both | 10 to 19 | Pertussis | Rate | 2021 | 0.438286057 | 0.728580613 | 0.224159917 |
| Prevalence | Mongolia | Both | 10 to 19 | Pertussis | Number | 2021 | 1.088007334 | 4.124882091 | 0.068791668 |
| Prevalence | Mongolia | Both | 10 to 19 | Pertussis | Rate | 2021 | 0.20497256 | 0.777097376 | 0.012959843 |
| Prevalence | Albania | Both | 10 to 19 | Pertussis | Number | 2021 | 0.009573735 | 0.020879776 | 0.003325375 |
| Prevalence | Albania | Both | 10 to 19 | Pertussis | Rate | 2021 | 0.002939289 | 0.006410423 | 0.001020943 |
| Prevalence | United States of America | Both | 10 to 19 | Pertussis | Number | 2021 | 208.4451287 | 396.2087578 | 90.69174578 |
| Prevalence | United States of America | Both | 10 to 19 | Pertussis | Rate | 2021 | 0.485283056 | 0.922417319 | 0.211140302 |
| Prevalence | Honduras | Both | 10 to 19 | Pertussis | Number | 2021 | 38.19445098 | 86.90000237 | 11.13539863 |
| Prevalence | Honduras | Both | 10 to 19 | Pertussis | Rate | 2021 | 1.767781828 | 4.022056636 | 0.515387834 |
| Prevalence | Cyprus | Both | 10 to 19 | Pertussis | Number | 2021 | 0.152163711 | 0.263172396 | 0.07239392 |
| Prevalence | Cyprus | Both | 10 to 19 | Pertussis | Rate | 2021 | 0.11107116 | 0.19210141 | 0.05284359 |
| Prevalence | Canada | Both | 10 to 19 | Pertussis | Number | 2021 | 13.55198112 | 33.39118391 | 3.076442103 |
| Prevalence | Canada | Both | 10 to 19 | Pertussis | Rate | 2021 | 0.3204145 | 0.789480106 | 0.072737458 |
| Prevalence | Montenegro | Both | 10 to 19 | Pertussis | Number | 2021 | 0.058476448 | 0.124625649 | 0.021535996 |
| Prevalence | Montenegro | Both | 10 to 19 | Pertussis | Rate | 2021 | 0.075832068 | 0.161614138 | 0.027927809 |
| Prevalence | France | Both | 10 to 19 | Pertussis | Number | 2021 | 11.82199668 | 25.33979997 | 4.087557482 |
| Prevalence | France | Both | 10 to 19 | Pertussis | Rate | 2021 | 0.142428577 | 0.305287825 | 0.049245911 |
| Prevalence | Norway | Both | 10 to 19 | Pertussis | Number | 2021 | 0.410997656 | 1.358976139 | 0.070889458 |
| Prevalence | Norway | Both | 10 to 19 | Pertussis | Rate | 2021 | 0.063555336 | 0.210147633 | 0.010962114 |
| Prevalence | Spain | Both | 10 to 19 | Pertussis | Number | 2021 | 6.237905184 | 16.65046033 | 1.523316371 |
| Prevalence | Spain | Both | 10 to 19 | Pertussis | Rate | 2021 | 0.130490918 | 0.348311459 | 0.031866299 |
| Prevalence | Antigua and Barbuda | Both | 10 to 19 | Pertussis | Number | 2021 | 0.001840093 | 0.00838653 | 0.000534383 |
| Prevalence | Antigua and Barbuda | Both | 10 to 19 | Pertussis | Rate | 2021 | 0.014613006 | 0.066601198 | 0.004243776 |
| Prevalence | Bahamas | Both | 10 to 19 | Pertussis | Number | 2021 | 0.014588976 | 0.066573531 | 0.004239532 |
| Prevalence | Bahamas | Both | 10 to 19 | Pertussis | Rate | 2021 | 0.021430826 | 0.097794784 | 0.006227762 |
| Prevalence | Malta | Both | 10 to 19 | Pertussis | Number | 2021 | 0.034796081 | 0.060577262 | 0.016575608 |
| Prevalence | Malta | Both | 10 to 19 | Pertussis | Rate | 2021 | 0.085903029 | 0.149550472 | 0.040921131 |
| Prevalence | Italy | Both | 10 to 19 | Pertussis | Number | 2021 | 0.83094976 | 1.494852958 | 0.389416105 |
| Prevalence | Italy | Both | 10 to 19 | Pertussis | Rate | 2021 | 0.014496354 | 0.026078492 | 0.006793568 |
| Prevalence | Singapore | Both | 10 to 19 | Pertussis | Number | 2021 | 0.087471283 | 0.306772114 | 0.009102621 |
| Prevalence | Singapore | Both | 10 to 19 | Pertussis | Rate | 2021 | 0.018875042 | 0.066197 | 0.001964214 |
| Prevalence | Colombia | Both | 10 to 19 | Pertussis | Number | 2021 | 32.78030177 | 84.00658383 | 7.303372417 |
| Prevalence | Colombia | Both | 10 to 19 | Pertussis | Rate | 2021 | 0.430459397 | 1.103144922 | 0.095905319 |
| Prevalence | Switzerland | Both | 10 to 19 | Pertussis | Number | 2021 | 0.856356062 | 1.484699811 | 0.408376224 |
| Prevalence | Switzerland | Both | 10 to 19 | Pertussis | Rate | 2021 | 0.098468252 | 0.170718469 | 0.046957212 |
| Prevalence | Sweden | Both | 10 to 19 | Pertussis | Number | 2021 | 3.843873649 | 9.215697939 | 1.043398739 |
| Prevalence | Sweden | Both | 10 to 19 | Pertussis | Rate | 2021 | 0.315346822 | 0.756044897 | 0.085599191 |
| Prevalence | Nicaragua | Both | 10 to 19 | Pertussis | Number | 2021 | 0.287556255 | 0.614392443 | 0.09106936 |
| Prevalence | Nicaragua | Both | 10 to 19 | Pertussis | Rate | 2021 | 0.022602458 | 0.048292393 | 0.007158222 |
| Prevalence | Chile | Both | 10 to 19 | Pertussis | Number | 2021 | 2.573881013 | 5.948029025 | 0.674616635 |
| Prevalence | Chile | Both | 10 to 19 | Pertussis | Rate | 2021 | 0.101882289 | 0.235441657 | 0.026703444 |
| Prevalence | Panama | Both | 10 to 19 | Pertussis | Number | 2021 | 17.61249552 | 42.95674573 | 3.789541318 |
| Prevalence | Panama | Both | 10 to 19 | Pertussis | Rate | 2021 | 2.39708991 | 5.846484482 | 0.515762871 |
| Prevalence | Russian Federation | Both | 10 to 19 | Pertussis | Number | 2021 | 43.06471623 | 95.58947502 | 10.81777278 |
| Prevalence | Russian Federation | Both | 10 to 19 | Pertussis | Rate | 2021 | 0.258768714 | 0.57438125 | 0.065002197 |
| Prevalence | Andorra | Both | 10 to 19 | Pertussis | Number | 2021 | 0.003203881 | 0.005586531 | 0.00151623 |
| Prevalence | Andorra | Both | 10 to 19 | Pertussis | Rate | 2021 | 0.037777631 | 0.065871954 | 0.017878184 |
| Prevalence | Australia | Both | 10 to 19 | Pertussis | Number | 2021 | 0.823920965 | 1.873181843 | 0.203862634 |
| Prevalence | Australia | Both | 10 to 19 | Pertussis | Rate | 2021 | 0.026476022 | 0.060193158 | 0.006550958 |
| Prevalence | Cuba | Both | 10 to 19 | Pertussis | Number | 2021 | 0.148973146 | 0.678625935 | 0.042982988 |
| Prevalence | Cuba | Both | 10 to 19 | Pertussis | Rate | 2021 | 0.012016904 | 0.054741296 | 0.003467219 |
| Prevalence | New Zealand | Both | 10 to 19 | Pertussis | Number | 2021 | 0.167476185 | 0.386985015 | 0.041498849 |
| Prevalence | New Zealand | Both | 10 to 19 | Pertussis | Rate | 2021 | 0.02542445 | 0.058747942 | 0.006299913 |
| Prevalence | Belize | Both | 10 to 19 | Pertussis | Number | 2021 | 0.027701333 | 0.126743889 | 0.008013274 |
| Prevalence | Belize | Both | 10 to 19 | Pertussis | Rate | 2021 | 0.030806039 | 0.140949072 | 0.008911385 |
| Prevalence | Dominican Republic | Both | 10 to 19 | Pertussis | Number | 2021 | 0.863861385 | 3.982060471 | 0.250555111 |
| Prevalence | Dominican Republic | Both | 10 to 19 | Pertussis | Rate | 2021 | 0.045885629 | 0.211514662 | 0.013308708 |
| Prevalence | Iran (Islamic Republic of) | Both | 10 to 19 | Pertussis | Number | 2021 | 159.8027889 | 300.3493248 | 77.9283843 |
| Prevalence | Iran (Islamic Republic of) | Both | 10 to 19 | Pertussis | Rate | 2021 | 1.299477556 | 2.442367928 | 0.633694737 |
| Prevalence | Belgium | Both | 10 to 19 | Pertussis | Number | 2021 | 1.677162825 | 3.568701655 | 0.570218728 |
| Prevalence | Belgium | Both | 10 to 19 | Pertussis | Rate | 2021 | 0.128633281 | 0.273708549 | 0.04373404 |
| Prevalence | Saint Lucia | Both | 10 to 19 | Pertussis | Number | 2021 | 0.005122887 | 0.02337874 | 0.001487888 |
| Prevalence | Saint Lucia | Both | 10 to 19 | Pertussis | Rate | 2021 | 0.022106812 | 0.100886363 | 0.006420689 |
| Prevalence | Saint Vincent and the Grenadines | Both | 10 to 19 | Pertussis | Number | 2021 | 0.001508127 | 0.006815034 | 0.000433914 |
| Prevalence | Saint Vincent and the Grenadines | Both | 10 to 19 | Pertussis | Rate | 2021 | 0.008489495 | 0.038362946 | 0.002442573 |
| Prevalence | Greece | Both | 10 to 19 | Pertussis | Number | 2021 | 0.006210805 | 0.019534072 | 0.001043226 |
| Prevalence | Greece | Both | 10 to 19 | Pertussis | Rate | 2021 | 0.000600361 | 0.00188824 | 0.000100842 |
| Prevalence | Denmark | Both | 10 to 19 | Pertussis | Number | 2021 | 0.258676477 | 0.742638942 | 0.043444437 |
| Prevalence | Denmark | Both | 10 to 19 | Pertussis | Rate | 2021 | 0.0382056 | 0.109685142 | 0.006416589 |
| Prevalence | Austria | Both | 10 to 19 | Pertussis | Number | 2021 | 1.858260989 | 3.174654937 | 0.895235698 |
| Prevalence | Austria | Both | 10 to 19 | Pertussis | Rate | 2021 | 0.209209278 | 0.35741334 | 0.100788649 |
| Prevalence | Barbados | Both | 10 to 19 | Pertussis | Number | 2021 | 0.006947277 | 0.031671862 | 0.002019662 |
| Prevalence | Barbados | Both | 10 to 19 | Pertussis | Rate | 2021 | 0.018566979 | 0.084644784 | 0.005397657 |
| Prevalence | Jamaica | Both | 10 to 19 | Pertussis | Number | 2021 | 0.054831846 | 0.249822689 | 0.015842642 |
| Prevalence | Jamaica | Both | 10 to 19 | Pertussis | Rate | 2021 | 0.012175241 | 0.05547235 | 0.003517809 |
| Prevalence | Dominica | Both | 10 to 19 | Pertussis | Number | 2021 | 0.001347638 | 0.006139493 | 0.000389273 |
| Prevalence | Dominica | Both | 10 to 19 | Pertussis | Rate | 2021 | 0.011988913 | 0.054618387 | 0.003463062 |
| Prevalence | Suriname | Both | 10 to 19 | Pertussis | Number | 2021 | 0.04177334 | 0.192253458 | 0.012118512 |
| Prevalence | Suriname | Both | 10 to 19 | Pertussis | Rate | 2021 | 0.043240651 | 0.199006467 | 0.012544181 |
| Prevalence | Grenada | Both | 10 to 19 | Pertussis | Number | 2021 | 0.003717948 | 0.016964861 | 0.001081497 |
| Prevalence | Grenada | Both | 10 to 19 | Pertussis | Rate | 2021 | 0.023160727 | 0.105681546 | 0.006737119 |
| Prevalence | Palestine | Both | 10 to 19 | Pertussis | Number | 2021 | 2.818937971 | 5.43983305 | 1.22371464 |
| Prevalence | Palestine | Both | 10 to 19 | Pertussis | Rate | 2021 | 0.244391764 | 0.471613923 | 0.10609165 |
| Prevalence | Costa Rica | Both | 10 to 19 | Pertussis | Number | 2021 | 0.218651193 | 0.464504537 | 0.067802342 |
| Prevalence | Costa Rica | Both | 10 to 19 | Pertussis | Rate | 2021 | 0.030763529 | 0.065354314 | 0.009539574 |
| Prevalence | Netherlands | Both | 10 to 19 | Pertussis | Number | 2021 | 2.630318732 | 7.883174958 | 0.384702792 |
| Prevalence | Netherlands | Both | 10 to 19 | Pertussis | Rate | 2021 | 0.135207209 | 0.405221646 | 0.019775014 |
| Prevalence | Portugal | Both | 10 to 19 | Pertussis | Number | 2021 | 0.520190385 | 1.739650585 | 0.060094297 |
| Prevalence | Portugal | Both | 10 to 19 | Pertussis | Rate | 2021 | 0.049075315 | 0.16412049 | 0.00566936 |
| Prevalence | Bangladesh | Both | 10 to 19 | Pertussis | Number | 2021 | 386.9359426 | 702.4296727 | 183.2797023 |
| Prevalence | Bangladesh | Both | 10 to 19 | Pertussis | Rate | 2021 | 1.244012695 | 2.258336159 | 0.589250703 |
| Prevalence | United Kingdom | Both | 10 to 19 | Pertussis | Number | 2021 | 11.32920651 | 19.35101595 | 5.410758265 |
| Prevalence | United Kingdom | Both | 10 to 19 | Pertussis | Rate | 2021 | 0.141891244 | 0.242359403 | 0.067766372 |
| Prevalence | Mexico | Both | 10 to 19 | Pertussis | Number | 2021 | 236.038199 | 441.1172004 | 110.5584952 |
| Prevalence | Mexico | Both | 10 to 19 | Pertussis | Rate | 2021 | 1.055706694 | 1.972944986 | 0.494484977 |
| Prevalence | El Salvador | Both | 10 to 19 | Pertussis | Number | 2021 | 1.407749137 | 3.48696417 | 0.288199743 |
| Prevalence | El Salvador | Both | 10 to 19 | Pertussis | Rate | 2021 | 0.122291493 | 0.302913383 | 0.025035978 |
| Prevalence | Guatemala | Both | 10 to 19 | Pertussis | Number | 2021 | 41.81372298 | 89.55616546 | 15.19342977 |
| Prevalence | Guatemala | Both | 10 to 19 | Pertussis | Rate | 2021 | 1.257170862 | 2.692594529 | 0.456805466 |
| Prevalence | Pakistan | Both | 10 to 19 | Pertussis | Number | 2021 | 1437.218115 | 2185.986744 | 871.2077642 |
| Prevalence | Pakistan | Both | 10 to 19 | Pertussis | Rate | 2021 | 2.842897269 | 4.324003211 | 1.723297353 |
| Prevalence | Venezuela (Bolivarian Republic of) | Both | 10 to 19 | Pertussis | Number | 2021 | 48.6738542 | 80.87968584 | 25.57230931 |
| Prevalence | Venezuela (Bolivarian Republic of) | Both | 10 to 19 | Pertussis | Rate | 2021 | 1.117741833 | 1.857313538 | 0.587240118 |
| Prevalence | Bahrain | Both | 10 to 19 | Pertussis | Number | 2021 | 1.487817 | 2.840278087 | 0.652575492 |
| Prevalence | Bahrain | Both | 10 to 19 | Pertussis | Rate | 2021 | 0.718849607 | 1.37230102 | 0.315296596 |
| Prevalence | Bolivia (Plurinational State of) | Both | 10 to 19 | Pertussis | Number | 2021 | 2.754302538 | 7.091058619 | 0.599143742 |
| Prevalence | Bolivia (Plurinational State of) | Both | 10 to 19 | Pertussis | Rate | 2021 | 0.126553103 | 0.325815867 | 0.027529111 |
| Prevalence | Guyana | Both | 10 to 19 | Pertussis | Number | 2021 | 0.027526854 | 0.125492382 | 0.008003882 |
| Prevalence | Guyana | Both | 10 to 19 | Pertussis | Rate | 2021 | 0.02041412 | 0.093066083 | 0.005935738 |
| Prevalence | Morocco | Both | 10 to 19 | Pertussis | Number | 2021 | 49.95691887 | 95.37112157 | 21.91209318 |
| Prevalence | Morocco | Both | 10 to 19 | Pertussis | Rate | 2021 | 0.78416538 | 1.497024506 | 0.343950453 |
| Prevalence | Comoros | Both | 10 to 19 | Pertussis | Number | 2021 | 5.291858754 | 7.60184318 | 3.408081419 |
| Prevalence | Comoros | Both | 10 to 19 | Pertussis | Rate | 2021 | 3.479049453 | 4.997712445 | 2.24058962 |
| Prevalence | Tunisia | Both | 10 to 19 | Pertussis | Number | 2021 | 10.65857301 | 20.36049154 | 4.688262853 |
| Prevalence | Tunisia | Both | 10 to 19 | Pertussis | Rate | 2021 | 0.616156769 | 1.177010719 | 0.271021729 |
| Prevalence | Iraq | Both | 10 to 19 | Pertussis | Number | 2021 | 204.9715627 | 384.8349666 | 92.71357137 |
| Prevalence | Iraq | Both | 10 to 19 | Pertussis | Rate | 2021 | 2.356303014 | 4.423968768 | 1.065812568 |
| Prevalence | Trinidad and Tobago | Both | 10 to 19 | Pertussis | Number | 2021 | 0.079991053 | 0.368079559 | 0.023206617 |
| Prevalence | Trinidad and Tobago | Both | 10 to 19 | Pertussis | Rate | 2021 | 0.042431026 | 0.195246751 | 0.012309884 |
| Prevalence | Ecuador | Both | 10 to 19 | Pertussis | Number | 2021 | 44.40790169 | 122.1349011 | 8.522086685 |
| Prevalence | Ecuador | Both | 10 to 19 | Pertussis | Rate | 2021 | 1.370380738 | 3.768953487 | 0.262982555 |
| Prevalence | Libya | Both | 10 to 19 | Pertussis | Number | 2021 | 18.15501839 | 34.13391428 | 8.206646342 |
| Prevalence | Libya | Both | 10 to 19 | Pertussis | Rate | 2021 | 1.559943211 | 2.932906302 | 0.705143998 |
| Prevalence | Saudi Arabia | Both | 10 to 19 | Pertussis | Number | 2021 | 37.66998827 | 71.83893707 | 16.51076169 |
| Prevalence | Saudi Arabia | Both | 10 to 19 | Pertussis | Rate | 2021 | 0.739195982 | 1.40969127 | 0.323989713 |
| Prevalence | Oman | Both | 10 to 19 | Pertussis | Number | 2021 | 1.450828815 | 2.944630259 | 0.511636885 |
| Prevalence | Oman | Both | 10 to 19 | Pertussis | Rate | 2021 | 0.233503602 | 0.473923433 | 0.082345384 |
| Prevalence | Peru | Both | 10 to 19 | Pertussis | Number | 2021 | 318.9196598 | 743.6254393 | 84.79383294 |
| Prevalence | Peru | Both | 10 to 19 | Pertussis | Rate | 2021 | 5.307855932 | 12.37633547 | 1.411243977 |
| Prevalence | Turkey | Both | 10 to 19 | Pertussis | Number | 2021 | 100.2210436 | 190.9737055 | 43.88709674 |
| Prevalence | Turkey | Both | 10 to 19 | Pertussis | Rate | 2021 | 0.792224906 | 1.509604374 | 0.346917671 |
| Prevalence | Algeria | Both | 10 to 19 | Pertussis | Number | 2021 | 81.43501939 | 154.7952799 | 36.17506585 |
| Prevalence | Algeria | Both | 10 to 19 | Pertussis | Rate | 2021 | 1.147258767 | 2.180760111 | 0.509635311 |
| Prevalence | Brazil | Both | 10 to 19 | Pertussis | Number | 2021 | 499.0014745 | 1127.430213 | 139.9032046 |
| Prevalence | Brazil | Both | 10 to 19 | Pertussis | Rate | 2021 | 1.592835241 | 3.598808154 | 0.446577347 |
| Prevalence | Bhutan | Both | 10 to 19 | Pertussis | Number | 2021 | 0.139019428 | 0.257768288 | 0.058363136 |
| Prevalence | Bhutan | Both | 10 to 19 | Pertussis | Rate | 2021 | 0.104875236 | 0.194458504 | 0.044028721 |
| Prevalence | Lebanon | Both | 10 to 19 | Pertussis | Number | 2021 | 13.60170825 | 25.66804607 | 6.142321775 |
| Prevalence | Lebanon | Both | 10 to 19 | Pertussis | Rate | 2021 | 1.663644703 | 3.139496017 | 0.751276303 |
| Prevalence | Paraguay | Both | 10 to 19 | Pertussis | Number | 2021 | 6.600326507 | 14.88132075 | 1.755385858 |
| Prevalence | Paraguay | Both | 10 to 19 | Pertussis | Rate | 2021 | 0.491209453 | 1.107497549 | 0.130639314 |
| Prevalence | India | Both | 10 to 19 | Pertussis | Number | 2021 | 8669.615965 | 17254.14028 | 3255.641343 |
| Prevalence | India | Both | 10 to 19 | Pertussis | Rate | 2021 | 3.25354929 | 6.475165232 | 1.221783021 |
| Prevalence | Egypt | Both | 10 to 19 | Pertussis | Number | 2021 | 148.731565 | 336.1244218 | 47.7842147 |
| Prevalence | Egypt | Both | 10 to 19 | Pertussis | Rate | 2021 | 0.715335427 | 1.616615186 | 0.229821703 |
| Prevalence | Kuwait | Both | 10 to 19 | Pertussis | Number | 2021 | 3.806491956 | 7.272714467 | 1.67055078 |
| Prevalence | Kuwait | Both | 10 to 19 | Pertussis | Rate | 2021 | 0.709002005 | 1.354624992 | 0.3111589 |
| Prevalence | Democratic Republic of the Congo | Both | 10 to 19 | Pertussis | Number | 2021 | 2357.725039 | 4122.583351 | 1197.045097 |
| Prevalence | Democratic Republic of the Congo | Both | 10 to 19 | Pertussis | Rate | 2021 | 10.97324211 | 19.18718446 | 5.571245778 |
| Prevalence | Congo | Both | 10 to 19 | Pertussis | Number | 2021 | 105.5906962 | 183.3309988 | 53.90073495 |
| Prevalence | Congo | Both | 10 to 19 | Pertussis | Rate | 2021 | 8.828118425 | 15.32774976 | 4.506477259 |
| Prevalence | Jordan | Both | 10 to 19 | Pertussis | Number | 2021 | 32.88497226 | 77.21297308 | 9.108857561 |
| Prevalence | Jordan | Both | 10 to 19 | Pertussis | Rate | 2021 | 1.259455312 | 2.957165004 | 0.348858407 |
| Prevalence | Tuvalu | Both | 10 to 19 | Pertussis | Number | 2021 | 0.004125505 | 0.007212358 | 0.002019456 |
| Prevalence | Tuvalu | Both | 10 to 19 | Pertussis | Rate | 2021 | 0.172769453 | 0.302041809 | 0.08457152 |
| Prevalence | Central African Republic | Both | 10 to 19 | Pertussis | Number | 2021 | 156.1153015 | 274.0495069 | 79.21546345 |
| Prevalence | Central African Republic | Both | 10 to 19 | Pertussis | Rate | 2021 | 11.99029594 | 21.04812699 | 6.084072738 |
| Prevalence | Malawi | Both | 10 to 19 | Pertussis | Number | 2021 | 94.12371342 | 136.1040766 | 59.71940074 |
| Prevalence | Malawi | Both | 10 to 19 | Pertussis | Rate | 2021 | 1.818985019 | 2.630275277 | 1.154105499 |
| Prevalence | United Arab Emirates | Both | 10 to 19 | Pertussis | Number | 2021 | 5.674544687 | 10.83291846 | 2.48894017 |
| Prevalence | United Arab Emirates | Both | 10 to 19 | Pertussis | Rate | 2021 | 0.735840914 | 1.40474788 | 0.32275083 |
| Prevalence | Yemen | Both | 10 to 19 | Pertussis | Number | 2021 | 230.9295651 | 435.5853452 | 104.8593177 |
| Prevalence | Yemen | Both | 10 to 19 | Pertussis | Rate | 2021 | 2.846761288 | 5.3696351 | 1.292642829 |
| Prevalence | Ethiopia | Both | 10 to 19 | Pertussis | Number | 2021 | 1882.445604 | 2747.324498 | 1204.028686 |
| Prevalence | Ethiopia | Both | 10 to 19 | Pertussis | Rate | 2021 | 7.048462488 | 10.28683837 | 4.508258305 |
| Prevalence | Seychelles | Both | 10 to 19 | Pertussis | Number | 2021 | 0.012325083 | 0.021970102 | 0.006015066 |
| Prevalence | Seychelles | Both | 10 to 19 | Pertussis | Rate | 2021 | 0.083078091 | 0.148091024 | 0.040544976 |
| Prevalence | Mozambique | Both | 10 to 19 | Pertussis | Number | 2021 | 106.9799843 | 154.3162009 | 67.92089766 |
| Prevalence | Mozambique | Both | 10 to 19 | Pertussis | Rate | 2021 | 1.392112146 | 2.008090196 | 0.883842966 |
| Prevalence | Syrian Arab Republic | Both | 10 to 19 | Pertussis | Number | 2021 | 80.84205577 | 152.4301013 | 36.69608847 |
| Prevalence | Syrian Arab Republic | Both | 10 to 19 | Pertussis | Rate | 2021 | 2.420254379 | 4.563461638 | 1.098609727 |
| Prevalence | Equatorial Guinea | Both | 10 to 19 | Pertussis | Number | 2021 | 38.64634834 | 67.58705613 | 19.6199209 |
| Prevalence | Equatorial Guinea | Both | 10 to 19 | Pertussis | Rate | 2021 | 10.06804891 | 17.60760889 | 5.111332162 |
| Prevalence | Mauritius | Both | 10 to 19 | Pertussis | Number | 2021 | 0.172795846 | 0.305141087 | 0.084357403 |
| Prevalence | Mauritius | Both | 10 to 19 | Pertussis | Rate | 2021 | 0.104190375 | 0.183990326 | 0.050864819 |
| Prevalence | Angola | Both | 10 to 19 | Pertussis | Number | 2021 | 1003.314474 | 1765.38315 | 507.9662667 |
| Prevalence | Angola | Both | 10 to 19 | Pertussis | Rate | 2021 | 12.84513802 | 22.60167754 | 6.503341647 |
| Prevalence | Qatar | Both | 10 to 19 | Pertussis | Number | 2021 | 0.20530963 | 0.434606743 | 0.068770381 |
| Prevalence | Qatar | Both | 10 to 19 | Pertussis | Rate | 2021 | 0.08365179 | 0.17707709 | 0.028019949 |
| Prevalence | Botswana | Both | 10 to 19 | Pertussis | Number | 2021 | 5.945994795 | 12.04505947 | 2.366717876 |
| Prevalence | Botswana | Both | 10 to 19 | Pertussis | Rate | 2021 | 1.327626776 | 2.689431126 | 0.528442781 |
| Prevalence | Nepal | Both | 10 to 19 | Pertussis | Number | 2021 | 133.2138192 | 257.2877351 | 53.09512709 |
| Prevalence | Nepal | Both | 10 to 19 | Pertussis | Rate | 2021 | 2.109389506 | 4.074052165 | 0.840740883 |
| Prevalence | Zimbabwe | Both | 10 to 19 | Pertussis | Number | 2021 | 29.8286918 | 60.22348322 | 11.73207 |
| Prevalence | Zimbabwe | Both | 10 to 19 | Pertussis | Rate | 2021 | 0.817319861 | 1.650151111 | 0.321464108 |
| Prevalence | Namibia | Both | 10 to 19 | Pertussis | Number | 2021 | 4.237422761 | 8.550078504 | 1.668547641 |
| Prevalence | Namibia | Both | 10 to 19 | Pertussis | Rate | 2021 | 0.820062201 | 1.654684131 | 0.322911574 |
| Prevalence | Afghanistan | Both | 10 to 19 | Pertussis | Number | 2021 | 210.3705253 | 396.8596202 | 95.53560488 |
| Prevalence | Afghanistan | Both | 10 to 19 | Pertussis | Rate | 2021 | 2.770120371 | 5.225774462 | 1.257995268 |
| Prevalence | Gabon | Both | 10 to 19 | Pertussis | Number | 2021 | 27.28705659 | 47.27188734 | 13.9829524 |
| Prevalence | Gabon | Both | 10 to 19 | Pertussis | Rate | 2021 | 6.903747489 | 11.95999914 | 3.537749562 |
| Prevalence | Senegal | Both | 10 to 19 | Pertussis | Number | 2021 | 78.16892059 | 134.0150449 | 40.628867 |
| Prevalence | Senegal | Both | 10 to 19 | Pertussis | Rate | 2021 | 2.093351006 | 3.588901149 | 1.088034464 |
| Prevalence | Lesotho | Both | 10 to 19 | Pertussis | Number | 2021 | 4.437880909 | 8.984819853 | 1.76295885 |
| Prevalence | Lesotho | Both | 10 to 19 | Pertussis | Rate | 2021 | 1.051063011 | 2.127955211 | 0.417537305 |
| Prevalence | Eritrea | Both | 10 to 19 | Pertussis | Number | 2021 | 31.77552258 | 45.85221587 | 20.26474574 |
| Prevalence | Eritrea | Both | 10 to 19 | Pertussis | Rate | 2021 | 2.186637802 | 3.155327761 | 1.394521804 |
| Prevalence | Madagascar | Both | 10 to 19 | Pertussis | Number | 2021 | 328.2048487 | 640.5968477 | 121.5734339 |
| Prevalence | Madagascar | Both | 10 to 19 | Pertussis | Rate | 2021 | 4.778524407 | 9.326820382 | 1.770058009 |
| Prevalence | Djibouti | Both | 10 to 19 | Pertussis | Number | 2021 | 18.46514781 | 26.84853454 | 11.79461501 |
| Prevalence | Djibouti | Both | 10 to 19 | Pertussis | Rate | 2021 | 7.568355571 | 11.00447492 | 4.83428788 |
| Prevalence | Kenya | Both | 10 to 19 | Pertussis | Number | 2021 | 354.1157569 | 510.304669 | 229.1542463 |
| Prevalence | Kenya | Both | 10 to 19 | Pertussis | Rate | 2021 | 2.867715703 | 4.132571578 | 1.855746935 |
| Prevalence | United Republic of Tanzania | Both | 10 to 19 | Pertussis | Number | 2021 | 395.5415133 | 595.8593933 | 239.802201 |
| Prevalence | United Republic of Tanzania | Both | 10 to 19 | Pertussis | Rate | 2021 | 2.856987543 | 4.303879131 | 1.732085958 |
| Prevalence | Burkina Faso | Both | 10 to 19 | Pertussis | Number | 2021 | 90.80444108 | 133.3547403 | 57.38777024 |
| Prevalence | Burkina Faso | Both | 10 to 19 | Pertussis | Rate | 2021 | 1.711007156 | 2.512772637 | 1.081344529 |
| Prevalence | Cabo Verde | Both | 10 to 19 | Pertussis | Number | 2021 | 0.219447196 | 0.341628507 | 0.132030731 |
| Prevalence | Cabo Verde | Both | 10 to 19 | Pertussis | Rate | 2021 | 0.218810649 | 0.340637551 | 0.131647752 |
| Prevalence | Cameroon | Both | 10 to 19 | Pertussis | Number | 2021 | 335.0227126 | 552.8555047 | 171.8944862 |
| Prevalence | Cameroon | Both | 10 to 19 | Pertussis | Rate | 2021 | 4.47344249 | 7.382088476 | 2.295247664 |
| Prevalence | Burundi | Both | 10 to 19 | Pertussis | Number | 2021 | 42.35484145 | 61.14038558 | 26.87905197 |
| Prevalence | Burundi | Both | 10 to 19 | Pertussis | Rate | 2021 | 1.342315603 | 1.937669714 | 0.851854702 |
| Prevalence | Coted'Ivoire | Both | 10 to 19 | Pertussis | Number | 2021 | 143.0829569 | 227.4973245 | 83.29296492 |
| Prevalence | Coted'Ivoire | Both | 10 to 19 | Pertussis | Rate | 2021 | 2.328776529 | 3.702680188 | 1.355652035 |
| Prevalence | Niger | Both | 10 to 19 | Pertussis | Number | 2021 | 377.3756285 | 559.9981715 | 235.4718461 |
| Prevalence | Niger | Both | 10 to 19 | Pertussis | Rate | 2021 | 6.025093722 | 8.940803837 | 3.759490105 |
| Prevalence | Gambia | Both | 10 to 19 | Pertussis | Number | 2021 | 11.51989781 | 16.93080274 | 7.286341625 |
| Prevalence | Gambia | Both | 10 to 19 | Pertussis | Rate | 2021 | 1.987406967 | 2.920893558 | 1.257035986 |
| Prevalence | Sao Tome and Principe | Both | 10 to 19 | Pertussis | Number | 2021 | 0.826800008 | 1.215065747 | 0.522602509 |
| Prevalence | Sao Tome and Principe | Both | 10 to 19 | Pertussis | Rate | 2021 | 1.634310962 | 2.401784289 | 1.033012822 |
| Prevalence | Ghana | Both | 10 to 19 | Pertussis | Number | 2021 | 124.3288249 | 182.5206976 | 78.58883408 |
| Prevalence | Ghana | Both | 10 to 19 | Pertussis | Rate | 2021 | 1.70018037 | 2.495946595 | 1.074691996 |
| Prevalence | Nigeria | Both | 10 to 19 | Pertussis | Number | 2021 | 3655.462031 | 5412.396186 | 2279.122724 |
| Prevalence | Nigeria | Both | 10 to 19 | Pertussis | Rate | 2021 | 6.427235227 | 9.516373891 | 4.007279444 |
| Prevalence | Rwanda | Both | 10 to 19 | Pertussis | Number | 2021 | 35.08775768 | 50.58955796 | 22.24959365 |
| Prevalence | Rwanda | Both | 10 to 19 | Pertussis | Rate | 2021 | 1.156021083 | 1.66675215 | 0.733047679 |
| Prevalence | Somalia | Both | 10 to 19 | Pertussis | Number | 2021 | 623.0957546 | 920.7682512 | 395.3602788 |
| Prevalence | Somalia | Both | 10 to 19 | Pertussis | Rate | 2021 | 11.84451593 | 17.50301481 | 7.515459842 |
| Prevalence | Sierra Leone | Both | 10 to 19 | Pertussis | Number | 2021 | 54.77325069 | 80.59917493 | 34.50448598 |
| Prevalence | Sierra Leone | Both | 10 to 19 | Pertussis | Rate | 2021 | 2.701563608 | 3.975367448 | 1.701853778 |
| Prevalence | Eswatini | Both | 10 to 19 | Pertussis | Number | 2021 | 2.599484236 | 5.266904362 | 1.0303063 |
| Prevalence | Eswatini | Both | 10 to 19 | Pertussis | Rate | 2021 | 1.015624894 | 2.057792507 | 0.402543209 |
| Prevalence | South Africa | Both | 10 to 19 | Pertussis | Number | 2021 | 205.1682698 | 413.9232981 | 81.90665179 |
| Prevalence | South Africa | Both | 10 to 19 | Pertussis | Rate | 2021 | 2.096227164 | 4.22910064 | 0.836849424 |
| Prevalence | Uganda | Both | 10 to 19 | Pertussis | Number | 2021 | 527.1148269 | 1102.642638 | 195.3686149 |
| Prevalence | Uganda | Both | 10 to 19 | Pertussis | Rate | 2021 | 4.778627467 | 9.996149085 | 1.771139383 |
| Prevalence | Zambia | Both | 10 to 19 | Pertussis | Number | 2021 | 157.0378527 | 251.681076 | 88.49577813 |
| Prevalence | Zambia | Both | 10 to 19 | Pertussis | Rate | 2021 | 3.326582627 | 5.331440034 | 1.874634128 |
| Prevalence | Niue | Both | 10 to 19 | Pertussis | Number | 2021 | 0.000164961 | 0.000294938 | 8.02E-05 |
| Prevalence | Niue | Both | 10 to 19 | Pertussis | Rate | 2021 | 0.058906442 | 0.105320352 | 0.028628515 |
| Prevalence | Guinea-Bissau | Both | 10 to 19 | Pertussis | Number | 2021 | 22.57642917 | 33.36080684 | 14.12384927 |
| Prevalence | Guinea-Bissau | Both | 10 to 19 | Pertussis | Rate | 2021 | 4.667010032 | 6.896361645 | 2.919688748 |
| Prevalence | Mauritania | Both | 10 to 19 | Pertussis | Number | 2021 | 53.91578273 | 79.81201844 | 33.67134327 |
| Prevalence | Mauritania | Both | 10 to 19 | Pertussis | Rate | 2021 | 5.161356492 | 7.640402469 | 3.223356824 |
| Prevalence | Benin | Both | 10 to 19 | Pertussis | Number | 2021 | 109.9943753 | 162.3807816 | 69.03019515 |
| Prevalence | Benin | Both | 10 to 19 | Pertussis | Rate | 2021 | 3.49887753 | 5.165268374 | 2.195823178 |
| Prevalence | Greenland | Both | 10 to 19 | Pertussis | Number | 2021 | 0.018263165 | 0.032239137 | 0.0085367 |
| Prevalence | Greenland | Both | 10 to 19 | Pertussis | Rate | 2021 | 0.246441799 | 0.435032524 | 0.115193592 |
| Prevalence | Saint Kitts and Nevis | Both | 10 to 19 | Pertussis | Number | 2021 | 0.000765613 | 0.003487416 | 0.000220226 |
| Prevalence | Saint Kitts and Nevis | Both | 10 to 19 | Pertussis | Rate | 2021 | 0.009897816 | 0.045085169 | 0.002847072 |
| Prevalence | Tokelau | Both | 10 to 19 | Pertussis | Number | 2021 | 0.000264495 | 0.000468321 | 0.000129123 |
| Prevalence | Tokelau | Both | 10 to 19 | Pertussis | Rate | 2021 | 0.098609326 | 0.17460007 | 0.048139938 |
| Prevalence | Monaco | Both | 10 to 19 | Pertussis | Number | 2021 | 0.001099806 | 0.001932586 | 0.00052075 |
| Prevalence | Monaco | Both | 10 to 19 | Pertussis | Rate | 2021 | 0.031816304 | 0.055907821 | 0.015064802 |
| Prevalence | Guinea | Both | 10 to 19 | Pertussis | Number | 2021 | 243.8228209 | 360.9948574 | 151.4876087 |
| Prevalence | Guinea | Both | 10 to 19 | Pertussis | Rate | 2021 | 7.705257637 | 11.40811336 | 4.787292057 |
| Prevalence | Liberia | Both | 10 to 19 | Pertussis | Number | 2021 | 31.73128484 | 46.69619457 | 20.02817873 |
| Prevalence | Liberia | Both | 10 to 19 | Pertussis | Rate | 2021 | 2.445918859 | 3.599447785 | 1.543817097 |
| Prevalence | Chad | Both | 10 to 19 | Pertussis | Number | 2021 | 360.5241559 | 534.5474919 | 223.337673 |
| Prevalence | Chad | Both | 10 to 19 | Pertussis | Rate | 2021 | 8.314198856 | 12.32742404 | 5.150483802 |
| Prevalence | Nauru | Both | 10 to 19 | Pertussis | Number | 2021 | 0.000865059 | 0.001559226 | 0.000411302 |
| Prevalence | Nauru | Both | 10 to 19 | Pertussis | Rate | 2021 | 0.036037637 | 0.064956049 | 0.017134496 |
| Prevalence | United States Virgin Islands | Both | 10 to 19 | Pertussis | Number | 2021 | 0.005438643 | 0.025202156 | 0.001572382 |
| Prevalence | United States Virgin Islands | Both | 10 to 19 | Pertussis | Rate | 2021 | 0.055408659 | 0.256758482 | 0.016019361 |
| Prevalence | Cook Islands | Both | 10 to 19 | Pertussis | Number | 2021 | 0.00198507 | 0.003535057 | 0.000967892 |
| Prevalence | Cook Islands | Both | 10 to 19 | Pertussis | Rate | 2021 | 0.07068403 | 0.125875702 | 0.034464527 |
| Prevalence | Bermuda | Both | 10 to 19 | Pertussis | Number | 2021 | 0.000229331 | 0.001036417 | 6.31E-05 |
| Prevalence | Bermuda | Both | 10 to 19 | Pertussis | Rate | 2021 | 0.003713449 | 0.016782182 | 0.001022225 |
| Prevalence | Togo | Both | 10 to 19 | Pertussis | Number | 2021 | 106.8083547 | 173.6816297 | 56.80153141 |
| Prevalence | Togo | Both | 10 to 19 | Pertussis | Rate | 2021 | 5.695570415 | 9.26159713 | 3.028949586 |
| Prevalence | Mali | Both | 10 to 19 | Pertussis | Number | 2021 | 273.3855512 | 404.065785 | 170.9744131 |
| Prevalence | Mali | Both | 10 to 19 | Pertussis | Rate | 2021 | 4.683859887 | 6.922778156 | 2.929270372 |
| Prevalence | Sudan | Both | 10 to 19 | Pertussis | Number | 2021 | 232.4625427 | 435.787878 | 105.238826 |
| Prevalence | Sudan | Both | 10 to 19 | Pertussis | Rate | 2021 | 2.292434278 | 4.297531369 | 1.037814907 |
| Prevalence | San Marino | Both | 10 to 19 | Pertussis | Number | 2021 | 0.007972408 | 0.013612674 | 0.003849381 |
| Prevalence | San Marino | Both | 10 to 19 | Pertussis | Rate | 2021 | 0.223449389 | 0.381533871 | 0.107889855 |
| Prevalence | American Samoa | Both | 10 to 19 | Pertussis | Number | 2021 | 0.01095193 | 0.019392302 | 0.00534822 |
| Prevalence | American Samoa | Both | 10 to 19 | Pertussis | Rate | 2021 | 0.104668261 | 0.185333406 | 0.051113267 |
| Prevalence | Puerto Rico | Both | 10 to 19 | Pertussis | Number | 2021 | 0.086689843 | 0.395626058 | 0.025172684 |
| Prevalence | Puerto Rico | Both | 10 to 19 | Pertussis | Rate | 2021 | 0.021776621 | 0.09938187 | 0.006323417 |
| Prevalence | Palau | Both | 10 to 19 | Pertussis | Number | 2021 | 0.003353754 | 0.005884789 | 0.001634439 |
| Prevalence | Palau | Both | 10 to 19 | Pertussis | Rate | 2021 | 0.14003919 | 0.245724987 | 0.068247547 |
| Prevalence | Northern Mariana Islands | Both | 10 to 19 | Pertussis | Number | 2021 | 0.004877058 | 0.008674069 | 0.00235898 |
| Prevalence | Northern Mariana Islands | Both | 10 to 19 | Pertussis | Rate | 2021 | 0.060609834 | 0.107797335 | 0.029316314 |
| Prevalence | Guam | Both | 10 to 19 | Pertussis | Number | 2021 | 0.012637745 | 0.022477884 | 0.006088468 |
| Prevalence | Guam | Both | 10 to 19 | Pertussis | Rate | 2021 | 0.052416227 | 0.093229123 | 0.025252489 |
| Prevalence | South Sudan | Both | 10 to 19 | Pertussis | Number | 2021 | 193.3397231 | 281.2686765 | 123.5156892 |
| Prevalence | South Sudan | Both | 10 to 19 | Pertussis | Rate | 2021 | 7.569528409 | 11.01207348 | 4.835816993 |

Sup_Table 5 Incidence of Adolescent Pertussis in 204 Countries and Territories in 2021

| measure | location | sex | age | cause | metric | year | val | upper | lower |
| --- | --- | --- | --- | --- | --- | --- | --- | --- | --- |
| Incidence | Democratic People's Republic of Korea | Both | 10 to 19 | Pertussis | Number | 2021 | 16.15764439 | 29.02728414 | 8.160408767 |
| Incidence | Democratic People's Republic of Korea | Both | 10 to 19 | Pertussis | Rate | 2021 | 0.459923558 | 0.826254836 | 0.232284122 |
| Incidence | China | Both | 10 to 19 | Pertussis | Number | 2021 | 5086.356087 | 8751.967585 | 2677.358875 |
| Incidence | China | Both | 10 to 19 | Pertussis | Rate | 2021 | 3.161876496 | 5.440562974 | 1.664350265 |
| Incidence | Taiwan (Province of China) | Both | 10 to 19 | Pertussis | Number | 2021 | 42.1707919 | 72.24279056 | 22.05154038 |
| Incidence | Taiwan (Province of China) | Both | 10 to 19 | Pertussis | Rate | 2021 | 2.027482755 | 3.473281042 | 1.060191564 |
| Incidence | Sri Lanka | Both | 10 to 19 | Pertussis | Number | 2021 | 9.217367845 | 28.11447204 | 1.942812043 |
| Incidence | Sri Lanka | Both | 10 to 19 | Pertussis | Rate | 2021 | 0.25462523 | 0.776648391 | 0.053669222 |
| Incidence | Cambodia | Both | 10 to 19 | Pertussis | Number | 2021 | 8.431633195 | 18.14598447 | 2.706731539 |
| Incidence | Cambodia | Both | 10 to 19 | Pertussis | Rate | 2021 | 0.266296543 | 0.573105212 | 0.085486789 |
| Incidence | Lao People's Democratic Republic | Both | 10 to 19 | Pertussis | Number | 2021 | 164.2867331 | 318.9142771 | 69.40584547 |
| Incidence | Lao People's Democratic Republic | Both | 10 to 19 | Pertussis | Rate | 2021 | 11.70681587 | 22.72533302 | 4.945752088 |
| Incidence | Timor-Leste | Both | 10 to 19 | Pertussis | Number | 2021 | 12.0049471 | 20.88735513 | 5.80781635 |
| Incidence | Timor-Leste | Both | 10 to 19 | Pertussis | Rate | 2021 | 3.627958786 | 6.312269679 | 1.755152953 |
| Incidence | Maldives | Both | 10 to 19 | Pertussis | Number | 2021 | 1.175055005 | 2.062891806 | 0.576133323 |
| Incidence | Maldives | Both | 10 to 19 | Pertussis | Rate | 2021 | 1.845159406 | 3.239307268 | 0.904687708 |
| Incidence | Armenia | Both | 10 to 19 | Pertussis | Number | 2021 | 59.9958269 | 132.5714695 | 17.87321732 |
| Incidence | Armenia | Both | 10 to 19 | Pertussis | Rate | 2021 | 16.22556717 | 35.8532817 | 4.833720995 |
| Incidence | Papua New Guinea | Both | 10 to 19 | Pertussis | Number | 2021 | 117.5650205 | 206.704144 | 56.35098244 |
| Incidence | Papua New Guinea | Both | 10 to 19 | Pertussis | Rate | 2021 | 5.599588921 | 9.84526035 | 2.683981475 |
| Incidence | Solomon Islands | Both | 10 to 19 | Pertussis | Number | 2021 | 2.516921393 | 4.417054109 | 1.233834136 |
| Incidence | Solomon Islands | Both | 10 to 19 | Pertussis | Rate | 2021 | 1.683419642 | 2.95430587 | 0.825238573 |
| Incidence | Philippines | Both | 10 to 19 | Pertussis | Number | 2021 | 1776.025761 | 3340.480445 | 757.7958729 |
| Incidence | Philippines | Both | 10 to 19 | Pertussis | Rate | 2021 | 8.020045979 | 15.08469491 | 3.421998642 |
| Incidence | Kiribati | Both | 10 to 19 | Pertussis | Number | 2021 | 1.173822083 | 2.050878101 | 0.565231129 |
| Incidence | Kiribati | Both | 10 to 19 | Pertussis | Rate | 2021 | 4.721580048 | 8.249448756 | 2.27358478 |
| Incidence | Micronesia (Federated States of) | Both | 10 to 19 | Pertussis | Number | 2021 | 0.596995267 | 1.041488373 | 0.28950842 |
| Incidence | Micronesia (Federated States of) | Both | 10 to 19 | Pertussis | Rate | 2021 | 2.745069189 | 4.788911743 | 1.331200914 |
| Incidence | Georgia | Both | 10 to 19 | Pertussis | Number | 2021 | 2.435185383 | 8.193624354 | 0.507617528 |
| Incidence | Georgia | Both | 10 to 19 | Pertussis | Rate | 2021 | 0.573458824 | 1.929506568 | 0.11953823 |
| Incidence | Indonesia | Both | 10 to 19 | Pertussis | Number | 2021 | 1067.89266 | 2201.804425 | 392.9251707 |
| Incidence | Indonesia | Both | 10 to 19 | Pertussis | Rate | 2021 | 2.335175592 | 4.814716071 | 0.859214884 |
| Incidence | Kyrgyzstan | Both | 10 to 19 | Pertussis | Number | 2021 | 114.2731746 | 260.9301226 | 33.25482141 |
| Incidence | Kyrgyzstan | Both | 10 to 19 | Pertussis | Rate | 2021 | 9.266499805 | 21.15902475 | 2.696659099 |
| Incidence | Thailand | Both | 10 to 19 | Pertussis | Number | 2021 | 27.23489665 | 76.78929301 | 4.420919287 |
| Incidence | Thailand | Both | 10 to 19 | Pertussis | Rate | 2021 | 0.360154201 | 1.015461406 | 0.058462225 |
| Incidence | Viet Nam | Both | 10 to 19 | Pertussis | Number | 2021 | 361.1370775 | 834.4357713 | 100.4767156 |
| Incidence | Viet Nam | Both | 10 to 19 | Pertussis | Rate | 2021 | 2.448225028 | 5.656817499 | 0.681153017 |
| Incidence | Malaysia | Both | 10 to 19 | Pertussis | Number | 2021 | 503.5504949 | 957.3988188 | 226.3904608 |
| Incidence | Malaysia | Both | 10 to 19 | Pertussis | Rate | 2021 | 9.612647129 | 18.27649282 | 4.321734631 |
| Incidence | Vanuatu | Both | 10 to 19 | Pertussis | Number | 2021 | 1.177175945 | 2.066448005 | 0.577232842 |
| Incidence | Vanuatu | Both | 10 to 19 | Pertussis | Rate | 2021 | 1.796950941 | 3.154418594 | 0.881141943 |
| Incidence | Myanmar | Both | 10 to 19 | Pertussis | Number | 2021 | 315.4796794 | 552.0564212 | 152.974666 |
| Incidence | Myanmar | Both | 10 to 19 | Pertussis | Rate | 2021 | 3.048738508 | 5.334973311 | 1.478319478 |
| Incidence | Fiji | Both | 10 to 19 | Pertussis | Number | 2021 | 0.932697404 | 1.662429909 | 0.454524453 |
| Incidence | Fiji | Both | 10 to 19 | Pertussis | Rate | 2021 | 0.560448434 | 0.998937314 | 0.273119146 |
| Incidence | Tonga | Both | 10 to 19 | Pertussis | Number | 2021 | 0.01886026 | 0.034655224 | 0.008613611 |
| Incidence | Tonga | Both | 10 to 19 | Pertussis | Rate | 2021 | 0.084941255 | 0.156077288 | 0.038793258 |
| Incidence | Samoa | Both | 10 to 19 | Pertussis | Number | 2021 | 1.953010172 | 3.412169294 | 0.940451844 |
| Incidence | Samoa | Both | 10 to 19 | Pertussis | Rate | 2021 | 4.344773366 | 7.590898646 | 2.092180667 |
| Incidence | Azerbaijan | Both | 10 to 19 | Pertussis | Number | 2021 | 46.34823123 | 113.2633426 | 10.120524 |
| Incidence | Azerbaijan | Both | 10 to 19 | Pertussis | Rate | 2021 | 3.116490225 | 7.615913072 | 0.680511711 |
| Incidence | North Macedonia | Both | 10 to 19 | Pertussis | Number | 2021 | 1.198126422 | 2.553433165 | 0.441559711 |
| Incidence | North Macedonia | Both | 10 to 19 | Pertussis | Rate | 2021 | 0.514542948 | 1.096587976 | 0.189630602 |
| Incidence | Marshall Islands | Both | 10 to 19 | Pertussis | Number | 2021 | 0.276157656 | 0.481619236 | 0.13432002 |
| Incidence | Marshall Islands | Both | 10 to 19 | Pertussis | Rate | 2021 | 2.390733313 | 4.169441359 | 1.162826159 |
| Incidence | Kazakhstan | Both | 10 to 19 | Pertussis | Number | 2021 | 98.63288332 | 194.439528 | 38.14463761 |
| Incidence | Kazakhstan | Both | 10 to 19 | Pertussis | Rate | 2021 | 3.350845977 | 6.605676404 | 1.295884305 |
| Incidence | Poland | Both | 10 to 19 | Pertussis | Number | 2021 | 12.38381227 | 45.45193791 | 1.27801866 |
| Incidence | Poland | Both | 10 to 19 | Pertussis | Rate | 2021 | 0.319723913 | 1.173473168 | 0.032995746 |
| Incidence | Tajikistan | Both | 10 to 19 | Pertussis | Number | 2021 | 68.99273827 | 123.9952059 | 31.89440115 |
| Incidence | Tajikistan | Both | 10 to 19 | Pertussis | Rate | 2021 | 3.622906012 | 6.51116318 | 1.674819996 |
| Incidence | Bosnia and Herzegovina | Both | 10 to 19 | Pertussis | Number | 2021 | 2.693100987 | 5.761124049 | 0.990407947 |
| Incidence | Bosnia and Herzegovina | Both | 10 to 19 | Pertussis | Rate | 2021 | 0.774689722 | 1.657228455 | 0.284897915 |
| Incidence | Estonia | Both | 10 to 19 | Pertussis | Number | 2021 | 0.827761503 | 2.23915643 | 0.185283207 |
| Incidence | Estonia | Both | 10 to 19 | Pertussis | Rate | 2021 | 0.590036735 | 1.596093252 | 0.132071735 |
| Incidence | Uzbekistan | Both | 10 to 19 | Pertussis | Number | 2021 | 146.6081674 | 264.1370425 | 67.73060877 |
| Incidence | Uzbekistan | Both | 10 to 19 | Pertussis | Rate | 2021 | 2.715277548 | 4.891987902 | 1.254414434 |
| Incidence | Croatia | Both | 10 to 19 | Pertussis | Number | 2021 | 1.318529339 | 2.799976516 | 0.484382222 |
| Incidence | Croatia | Both | 10 to 19 | Pertussis | Rate | 2021 | 0.309683207 | 0.657630953 | 0.113766934 |
| Incidence | Mongolia | Both | 10 to 19 | Pertussis | Number | 2021 | 7.942453539 | 30.11163927 | 0.502179178 |
| Incidence | Mongolia | Both | 10 to 19 | Pertussis | Rate | 2021 | 1.496299691 | 5.672810842 | 0.094606855 |
| Incidence | Serbia | Both | 10 to 19 | Pertussis | Number | 2021 | 6.023408724 | 12.8515353 | 2.216962022 |
| Incidence | Serbia | Both | 10 to 19 | Pertussis | Rate | 2021 | 0.555874405 | 1.186012748 | 0.204593861 |
| Incidence | Hungary | Both | 10 to 19 | Pertussis | Number | 2021 | 0.600507062 | 1.289178687 | 0.213282917 |
| Incidence | Hungary | Both | 10 to 19 | Pertussis | Rate | 2021 | 0.062378869 | 0.133916007 | 0.022155188 |
| Incidence | Montenegro | Both | 10 to 19 | Pertussis | Number | 2021 | 0.426878068 | 0.909767238 | 0.157212767 |
| Incidence | Montenegro | Both | 10 to 19 | Pertussis | Rate | 2021 | 0.553574097 | 1.179783211 | 0.203873008 |
| Incidence | Bulgaria | Both | 10 to 19 | Pertussis | Number | 2021 | 2.546495287 | 5.41358405 | 0.936987604 |
| Incidence | Bulgaria | Both | 10 to 19 | Pertussis | Rate | 2021 | 0.382017508 | 0.812129477 | 0.140564042 |
| Incidence | Lithuania | Both | 10 to 19 | Pertussis | Number | 2021 | 33.93328591 | 65.14499695 | 14.75829037 |
| Incidence | Lithuania | Both | 10 to 19 | Pertussis | Rate | 2021 | 12.93559374 | 24.83370507 | 5.625958208 |
| Incidence | Albania | Both | 10 to 19 | Pertussis | Number | 2021 | 0.069888262 | 0.152422363 | 0.024275239 |
| Incidence | Albania | Both | 10 to 19 | Pertussis | Rate | 2021 | 0.021456806 | 0.046796085 | 0.007452884 |
| Incidence | Turkmenistan | Both | 10 to 19 | Pertussis | Number | 2021 | 2.94397089 | 5.430254133 | 1.294550414 |
| Incidence | Turkmenistan | Both | 10 to 19 | Pertussis | Rate | 2021 | 0.324988297 | 0.599451934 | 0.142906894 |
| Incidence | Romania | Both | 10 to 19 | Pertussis | Number | 2021 | 6.706672835 | 23.00635932 | 0.90249954 |
| Incidence | Romania | Both | 10 to 19 | Pertussis | Rate | 2021 | 0.316518178 | 1.085773992 | 0.042593029 |
| Incidence | Singapore | Both | 10 to 19 | Pertussis | Number | 2021 | 0.638540364 | 2.239436431 | 0.066449131 |
| Incidence | Singapore | Both | 10 to 19 | Pertussis | Rate | 2021 | 0.137787806 | 0.483238099 | 0.014338765 |
| Incidence | Slovenia | Both | 10 to 19 | Pertussis | Number | 2021 | 0.587592685 | 1.809244512 | 0.124857242 |
| Incidence | Slovenia | Both | 10 to 19 | Pertussis | Rate | 2021 | 0.293705498 | 0.904342538 | 0.062409318 |
| Incidence | Russian Federation | Both | 10 to 19 | Pertussis | Number | 2021 | 314.3724285 | 697.8031677 | 78.96974129 |
| Incidence | Russian Federation | Both | 10 to 19 | Pertussis | Rate | 2021 | 1.889011612 | 4.192983123 | 0.474516035 |
| Incidence | Belgium | Both | 10 to 19 | Pertussis | Number | 2021 | 12.24328862 | 26.05152208 | 4.162596712 |
| Incidence | Belgium | Both | 10 to 19 | Pertussis | Rate | 2021 | 0.939022953 | 1.998072411 | 0.319258492 |
| Incidence | Andorra | Both | 10 to 19 | Pertussis | Number | 2021 | 0.023388334 | 0.04078168 | 0.011068479 |
| Incidence | Andorra | Both | 10 to 19 | Pertussis | Rate | 2021 | 0.275776704 | 0.480865263 | 0.130510741 |
| Incidence | Germany | Both | 10 to 19 | Pertussis | Number | 2021 | 13.19241776 | 31.4198306 | 4.327535226 |
| Incidence | Germany | Both | 10 to 19 | Pertussis | Rate | 2021 | 0.167750818 | 0.399525118 | 0.055027637 |
| Incidence | Belarus | Both | 10 to 19 | Pertussis | Number | 2021 | 40.14178679 | 97.3562941 | 9.551928492 |
| Incidence | Belarus | Both | 10 to 19 | Pertussis | Rate | 2021 | 4.104384483 | 9.954406487 | 0.976657748 |
| Incidence | Slovakia | Both | 10 to 19 | Pertussis | Number | 2021 | 1.881477703 | 4.37316833 | 0.549835025 |
| Incidence | Slovakia | Both | 10 to 19 | Pertussis | Rate | 2021 | 0.338900499 | 0.787715382 | 0.099038837 |
| Incidence | Japan | Both | 10 to 19 | Pertussis | Number | 2021 | 1.718835332 | 9.442776153 | 0.067005019 |
| Incidence | Japan | Both | 10 to 19 | Pertussis | Rate | 2021 | 0.01510171 | 0.082964357 | 0.000588707 |
| Incidence | Latvia | Both | 10 to 19 | Pertussis | Number | 2021 | 0.020319981 | 0.115119012 | 0.000872368 |
| Incidence | Latvia | Both | 10 to 19 | Pertussis | Rate | 2021 | 0.010687636 | 0.06054878 | 0.000458837 |
| Incidence | Iceland | Both | 10 to 19 | Pertussis | Number | 2021 | 10.48511275 | 19.02471038 | 4.974614601 |
| Incidence | Iceland | Both | 10 to 19 | Pertussis | Rate | 2021 | 23.21801386 | 42.12791981 | 11.01568228 |
| Incidence | Denmark | Both | 10 to 19 | Pertussis | Number | 2021 | 1.888338283 | 5.421264273 | 0.317144389 |
| Incidence | Denmark | Both | 10 to 19 | Pertussis | Rate | 2021 | 0.278900877 | 0.800701535 | 0.046841103 |
| Incidence | Australia | Both | 10 to 19 | Pertussis | Number | 2021 | 6.014623048 | 13.67422745 | 1.488197231 |
| Incidence | Australia | Both | 10 to 19 | Pertussis | Rate | 2021 | 0.193274964 | 0.439410051 | 0.047821994 |
| Incidence | France | Both | 10 to 19 | Pertussis | Number | 2021 | 86.30057577 | 184.9805398 | 29.83916962 |
| Incidence | France | Both | 10 to 19 | Pertussis | Rate | 2021 | 1.039728612 | 2.228601121 | 0.35949515 |
| Incidence | Czechia | Both | 10 to 19 | Pertussis | Number | 2021 | 2.178191724 | 4.656242131 | 0.784870747 |
| Incidence | Czechia | Both | 10 to 19 | Pertussis | Rate | 2021 | 0.198664886 | 0.424678784 | 0.071585185 |
| Incidence | Brunei Darussalam | Both | 10 to 19 | Pertussis | Number | 2021 | 0.008241636 | 0.037548745 | 0.001236189 |
| Incidence | Brunei Darussalam | Both | 10 to 19 | Pertussis | Rate | 2021 | 0.012443279 | 0.056691353 | 0.001866407 |
| Incidence | Austria | Both | 10 to 19 | Pertussis | Number | 2021 | 13.56530522 | 23.17498104 | 6.535220592 |
| Incidence | Austria | Both | 10 to 19 | Pertussis | Rate | 2021 | 1.527227728 | 2.609117382 | 0.735757135 |
| Incidence | Norway | Both | 10 to 19 | Pertussis | Number | 2021 | 3.000282891 | 9.920525815 | 0.517493045 |
| Incidence | Norway | Both | 10 to 19 | Pertussis | Rate | 2021 | 0.46395395 | 1.53407772 | 0.080023435 |
| Incidence | Israel | Both | 10 to 19 | Pertussis | Number | 2021 | 9.653732536 | 16.84312184 | 4.591866186 |
| Incidence | Israel | Both | 10 to 19 | Pertussis | Rate | 2021 | 0.617846199 | 1.077972564 | 0.293882916 |
| Incidence | Republic of Moldova | Both | 10 to 19 | Pertussis | Number | 2021 | 11.42259782 | 23.97635316 | 3.754468056 |
| Incidence | Republic of Moldova | Both | 10 to 19 | Pertussis | Rate | 2021 | 3.162504807 | 6.63818628 | 1.039476611 |
| Incidence | Republic of Korea | Both | 10 to 19 | Pertussis | Number | 2021 | 0.171519441 | 0.224330659 | 0.128987674 |
| Incidence | Republic of Korea | Both | 10 to 19 | Pertussis | Rate | 2021 | 0.003706249 | 0.004847411 | 0.002787208 |
| Incidence | Ukraine | Both | 10 to 19 | Pertussis | Number | 2021 | 146.5427831 | 243.6039868 | 74.94880916 |
| Incidence | Ukraine | Both | 10 to 19 | Pertussis | Rate | 2021 | 3.199488214 | 5.318638478 | 1.636367391 |
| Incidence | Cyprus | Both | 10 to 19 | Pertussis | Number | 2021 | 1.110795088 | 1.921158493 | 0.528475619 |
| Incidence | Cyprus | Both | 10 to 19 | Pertussis | Rate | 2021 | 0.810819471 | 1.402340296 | 0.385758207 |
| Incidence | Finland | Both | 10 to 19 | Pertussis | Number | 2021 | 9.496619698 | 21.73269003 | 2.742170737 |
| Incidence | Finland | Both | 10 to 19 | Pertussis | Rate | 2021 | 1.54612766 | 3.538260374 | 0.446447911 |
| Incidence | New Zealand | Both | 10 to 19 | Pertussis | Number | 2021 | 1.222576152 | 2.824990611 | 0.302941601 |
| Incidence | New Zealand | Both | 10 to 19 | Pertussis | Rate | 2021 | 0.185598486 | 0.428859978 | 0.045989366 |
| Incidence | Argentina | Both | 10 to 19 | Pertussis | Number | 2021 | 198.0979422 | 398.9718373 | 75.00912704 |
| Incidence | Argentina | Both | 10 to 19 | Pertussis | Rate | 2021 | 2.800001254 | 5.639239016 | 1.060211163 |
| Incidence | Luxembourg | Both | 10 to 19 | Pertussis | Number | 2021 | 0.156749965 | 0.275384116 | 0.074219088 |
| Incidence | Luxembourg | Both | 10 to 19 | Pertussis | Rate | 2021 | 0.230913676 | 0.405677657 | 0.109334649 |
| Incidence | Ireland | Both | 10 to 19 | Pertussis | Number | 2021 | 10.34277078 | 31.51558391 | 2.05329287 |
| Incidence | Ireland | Both | 10 to 19 | Pertussis | Rate | 2021 | 1.513671655 | 4.612327496 | 0.300500831 |
| Incidence | Italy | Both | 10 to 19 | Pertussis | Number | 2021 | 6.065933248 | 10.9124266 | 2.842737569 |
| Incidence | Italy | Both | 10 to 19 | Pertussis | Rate | 2021 | 0.105823381 | 0.190372995 | 0.049593045 |
| Incidence | Greece | Both | 10 to 19 | Pertussis | Number | 2021 | 0.045338874 | 0.142598729 | 0.007615552 |
| Incidence | Greece | Both | 10 to 19 | Pertussis | Rate | 2021 | 0.004382633 | 0.013784151 | 0.000736149 |
| Incidence | Malta | Both | 10 to 19 | Pertussis | Number | 2021 | 0.254011388 | 0.442214012 | 0.12100194 |
| Incidence | Malta | Both | 10 to 19 | Pertussis | Rate | 2021 | 0.627092113 | 1.091718449 | 0.298724253 |
| Incidence | Uruguay | Both | 10 to 19 | Pertussis | Number | 2021 | 6.634825481 | 11.83573837 | 3.037507198 |
| Incidence | Uruguay | Both | 10 to 19 | Pertussis | Rate | 2021 | 1.408371906 | 2.512367726 | 0.644770509 |
| Incidence | Bahamas | Both | 10 to 19 | Pertussis | Number | 2021 | 0.106499522 | 0.485986775 | 0.030948581 |
| Incidence | Bahamas | Both | 10 to 19 | Pertussis | Rate | 2021 | 0.156445027 | 0.713901925 | 0.04546266 |
| Incidence | Spain | Both | 10 to 19 | Pertussis | Number | 2021 | 45.53670784 | 121.5483604 | 11.12020951 |
| Incidence | Spain | Both | 10 to 19 | Pertussis | Rate | 2021 | 0.952583702 | 2.542673652 | 0.232623983 |
| Incidence | Netherlands | Both | 10 to 19 | Pertussis | Number | 2021 | 19.20132674 | 57.54717719 | 2.80833038 |
| Incidence | Netherlands | Both | 10 to 19 | Pertussis | Rate | 2021 | 0.987012627 | 2.958118015 | 0.144357605 |
| Incidence | Canada | Both | 10 to 19 | Pertussis | Number | 2021 | 98.92946216 | 243.7556425 | 22.45802735 |
| Incidence | Canada | Both | 10 to 19 | Pertussis | Rate | 2021 | 2.339025848 | 5.763204772 | 0.530983443 |
| Incidence | Belize | Both | 10 to 19 | Pertussis | Number | 2021 | 0.20221973 | 0.925230391 | 0.058496901 |
| Incidence | Belize | Both | 10 to 19 | Pertussis | Rate | 2021 | 0.224884083 | 1.028928224 | 0.065053108 |
| Incidence | Grenada | Both | 10 to 19 | Pertussis | Number | 2021 | 0.027141023 | 0.123843489 | 0.007894929 |
| Incidence | Grenada | Both | 10 to 19 | Pertussis | Rate | 2021 | 0.169073309 | 0.771475289 | 0.049180969 |
| Incidence | Switzerland | Both | 10 to 19 | Pertussis | Number | 2021 | 6.251399253 | 10.83830862 | 2.981146434 |
| Incidence | Switzerland | Both | 10 to 19 | Pertussis | Rate | 2021 | 0.718818242 | 1.246244823 | 0.342787647 |
| Incidence | Guyana | Both | 10 to 19 | Pertussis | Number | 2021 | 0.200946035 | 0.916094387 | 0.058428337 |
| Incidence | Guyana | Both | 10 to 19 | Pertussis | Rate | 2021 | 0.149023072 | 0.679382404 | 0.043330889 |
| Incidence | Jamaica | Both | 10 to 19 | Pertussis | Number | 2021 | 0.400272475 | 1.823705628 | 0.115651286 |
| Incidence | Jamaica | Both | 10 to 19 | Pertussis | Rate | 2021 | 0.088879256 | 0.404948153 | 0.025680008 |
| Incidence | United States of America | Both | 10 to 19 | Pertussis | Number | 2021 | 1521.64944 | 2892.323932 | 662.0497442 |
| Incidence | United States of America | Both | 10 to 19 | Pertussis | Rate | 2021 | 3.542566308 | 6.733646426 | 1.541324208 |
| Incidence | Chile | Both | 10 to 19 | Pertussis | Number | 2021 | 18.7893314 | 43.42061188 | 4.924701437 |
| Incidence | Chile | Both | 10 to 19 | Pertussis | Rate | 2021 | 0.743740706 | 1.718724093 | 0.194935139 |
| Incidence | Cuba | Both | 10 to 19 | Pertussis | Number | 2021 | 1.087503964 | 4.953969329 | 0.313775814 |
| Incidence | Cuba | Both | 10 to 19 | Pertussis | Rate | 2021 | 0.087723402 | 0.399611458 | 0.025310696 |
| Incidence | Ecuador | Both | 10 to 19 | Pertussis | Number | 2021 | 324.1776823 | 891.5847781 | 62.2112328 |
| Incidence | Ecuador | Both | 10 to 19 | Pertussis | Rate | 2021 | 10.00377939 | 27.51336046 | 1.919772651 |
| Incidence | Saint Vincent and the Grenadines | Both | 10 to 19 | Pertussis | Number | 2021 | 0.011009328 | 0.049749747 | 0.003167571 |
| Incidence | Saint Vincent and the Grenadines | Both | 10 to 19 | Pertussis | Rate | 2021 | 0.061973316 | 0.280049503 | 0.017830781 |
| Incidence | Trinidad and Tobago | Both | 10 to 19 | Pertussis | Number | 2021 | 0.583934688 | 2.686980782 | 0.169408307 |
| Incidence | Trinidad and Tobago | Both | 10 to 19 | Pertussis | Rate | 2021 | 0.309746488 | 1.425301285 | 0.089862153 |
| Incidence | Honduras | Both | 10 to 19 | Pertussis | Number | 2021 | 278.8194922 | 634.3700173 | 81.28841003 |
| Incidence | Honduras | Both | 10 to 19 | Pertussis | Rate | 2021 | 12.90480734 | 29.36101344 | 3.762331185 |
| Incidence | United Kingdom | Both | 10 to 19 | Pertussis | Number | 2021 | 82.70320751 | 141.2624165 | 39.49853533 |
| Incidence | United Kingdom | Both | 10 to 19 | Pertussis | Rate | 2021 | 1.035806082 | 1.769223643 | 0.494694515 |
| Incidence | Portugal | Both | 10 to 19 | Pertussis | Number | 2021 | 3.797389813 | 12.69944927 | 0.43868837 |
| Incidence | Portugal | Both | 10 to 19 | Pertussis | Rate | 2021 | 0.358249802 | 1.198079579 | 0.041386328 |
| Incidence | Peru | Both | 10 to 19 | Pertussis | Number | 2021 | 2328.113517 | 5428.465707 | 618.9949805 |
| Incidence | Peru | Both | 10 to 19 | Pertussis | Rate | 2021 | 38.7473483 | 90.34724895 | 10.30208103 |
| Incidence | Bolivia (Plurinational State of) | Both | 10 to 19 | Pertussis | Number | 2021 | 20.10640853 | 51.76472792 | 4.373749316 |
| Incidence | Bolivia (Plurinational State of) | Both | 10 to 19 | Pertussis | Rate | 2021 | 0.92383765 | 2.37845583 | 0.200962508 |
| Incidence | Guatemala | Both | 10 to 19 | Pertussis | Number | 2021 | 305.2401778 | 653.7600079 | 110.9120373 |
| Incidence | Guatemala | Both | 10 to 19 | Pertussis | Rate | 2021 | 9.177347294 | 19.65594006 | 3.3346799 |
| Incidence | Sweden | Both | 10 to 19 | Pertussis | Number | 2021 | 28.06027764 | 67.27459495 | 7.616810797 |
| Incidence | Sweden | Both | 10 to 19 | Pertussis | Rate | 2021 | 2.302031799 | 5.519127746 | 0.624874097 |
| Incidence | Nicaragua | Both | 10 to 19 | Pertussis | Number | 2021 | 2.099160661 | 4.485064831 | 0.664806331 |
| Incidence | Nicaragua | Both | 10 to 19 | Pertussis | Rate | 2021 | 0.164997946 | 0.352534468 | 0.052255019 |
| Incidence | Colombia | Both | 10 to 19 | Pertussis | Number | 2021 | 239.2962029 | 613.248062 | 53.31461864 |
| Incidence | Colombia | Both | 10 to 19 | Pertussis | Rate | 2021 | 3.1423536 | 8.052957931 | 0.700108827 |
| Incidence | Dominican Republic | Both | 10 to 19 | Pertussis | Number | 2021 | 6.306188111 | 29.06904144 | 1.829052309 |
| Incidence | Dominican Republic | Both | 10 to 19 | Pertussis | Rate | 2021 | 0.334965091 | 1.544057033 | 0.097153568 |
| Incidence | Dominica | Both | 10 to 19 | Pertussis | Number | 2021 | 0.009837761 | 0.044818296 | 0.00284169 |
| Incidence | Dominica | Both | 10 to 19 | Pertussis | Rate | 2021 | 0.087519063 | 0.398714224 | 0.025280354 |
| Incidence | Barbados | Both | 10 to 19 | Pertussis | Number | 2021 | 0.050715125 | 0.231204593 | 0.014743533 |
| Incidence | Barbados | Both | 10 to 19 | Pertussis | Rate | 2021 | 0.135538947 | 0.617906923 | 0.039402899 |
| Incidence | Brazil | Both | 10 to 19 | Pertussis | Number | 2021 | 3642.710764 | 8230.240555 | 1021.293393 |
| Incidence | Brazil | Both | 10 to 19 | Pertussis | Rate | 2021 | 11.62769726 | 26.27129952 | 3.260014633 |
| Incidence | Antigua and Barbuda | Both | 10 to 19 | Pertussis | Number | 2021 | 0.01343268 | 0.061221669 | 0.003900997 |
| Incidence | Antigua and Barbuda | Both | 10 to 19 | Pertussis | Rate | 2021 | 0.106674942 | 0.486188749 | 0.030979565 |
| Incidence | Venezuela (Bolivarian Republic of) | Both | 10 to 19 | Pertussis | Number | 2021 | 355.3191356 | 590.4217066 | 186.6778579 |
| Incidence | Venezuela (Bolivarian Republic of) | Both | 10 to 19 | Pertussis | Rate | 2021 | 8.159515384 | 13.55838883 | 4.286852862 |
| Incidence | Bahrain | Both | 10 to 19 | Pertussis | Number | 2021 | 10.8610641 | 20.73403003 | 4.76380109 |
| Incidence | Bahrain | Both | 10 to 19 | Pertussis | Rate | 2021 | 5.247602134 | 10.01779745 | 2.301665153 |
| Incidence | Costa Rica | Both | 10 to 19 | Pertussis | Number | 2021 | 1.596153707 | 3.390883117 | 0.494957098 |
| Incidence | Costa Rica | Both | 10 to 19 | Pertussis | Rate | 2021 | 0.22457376 | 0.477086491 | 0.069638893 |
| Incidence | Libya | Both | 10 to 19 | Pertussis | Number | 2021 | 132.5316342 | 249.1775742 | 59.9085183 |
| Incidence | Libya | Both | 10 to 19 | Pertussis | Rate | 2021 | 11.38758544 | 21.41021601 | 5.147551185 |
| Incidence | Egypt | Both | 10 to 19 | Pertussis | Number | 2021 | 1085.740425 | 2453.708279 | 348.8247673 |
| Incidence | Egypt | Both | 10 to 19 | Pertussis | Rate | 2021 | 5.221948616 | 11.80129086 | 1.677698434 |
| Incidence | Iraq | Both | 10 to 19 | Pertussis | Number | 2021 | 1496.292408 | 2809.295256 | 676.809071 |
| Incidence | Iraq | Both | 10 to 19 | Pertussis | Rate | 2021 | 17.201012 | 32.294972 | 7.780431748 |
| Incidence | Jordan | Both | 10 to 19 | Pertussis | Number | 2021 | 240.0602975 | 563.6547035 | 66.4946602 |
| Incidence | Jordan | Both | 10 to 19 | Pertussis | Rate | 2021 | 9.19402378 | 21.58730453 | 2.546666373 |
| Incidence | Lebanon | Both | 10 to 19 | Pertussis | Number | 2021 | 99.29247023 | 187.3767363 | 44.83894896 |
| Incidence | Lebanon | Both | 10 to 19 | Pertussis | Rate | 2021 | 12.14460633 | 22.91832093 | 5.48431701 |
| Incidence | Mexico | Both | 10 to 19 | Pertussis | Number | 2021 | 1723.078853 | 3220.155563 | 807.0770153 |
| Incidence | Mexico | Both | 10 to 19 | Pertussis | Rate | 2021 | 7.706658866 | 14.4024984 | 3.609740335 |
| Incidence | Panama | Both | 10 to 19 | Pertussis | Number | 2021 | 128.5712173 | 313.5842439 | 27.66365162 |
| Incidence | Panama | Both | 10 to 19 | Pertussis | Rate | 2021 | 17.49875634 | 42.67933672 | 3.765068959 |
| Incidence | Saint Lucia | Both | 10 to 19 | Pertussis | Number | 2021 | 0.037397072 | 0.1706648 | 0.010861583 |
| Incidence | Saint Lucia | Both | 10 to 19 | Pertussis | Rate | 2021 | 0.161379724 | 0.736470446 | 0.046871029 |
| Incidence | Haiti | Both | 10 to 19 | Pertussis | Number | 2021 | 11.89853487 | 55.4012341 | 3.431353142 |
| Incidence | Haiti | Both | 10 to 19 | Pertussis | Rate | 2021 | 0.458828036 | 2.136367182 | 0.132318898 |
| Incidence | El Salvador | Both | 10 to 19 | Pertussis | Number | 2021 | 10.2765687 | 25.45483844 | 2.10385812 |
| Incidence | El Salvador | Both | 10 to 19 | Pertussis | Rate | 2021 | 0.892727898 | 2.211267698 | 0.182762641 |
| Incidence | Oman | Both | 10 to 19 | Pertussis | Number | 2021 | 10.59105035 | 21.49580089 | 3.734949261 |
| Incidence | Oman | Both | 10 to 19 | Pertussis | Rate | 2021 | 1.704576294 | 3.459641057 | 0.601121302 |
| Incidence | Kuwait | Both | 10 to 19 | Pertussis | Number | 2021 | 27.78739128 | 53.09081561 | 12.19502069 |
| Incidence | Kuwait | Both | 10 to 19 | Pertussis | Rate | 2021 | 5.175714634 | 9.888762442 | 2.271459974 |
| Incidence | India | Both | 10 to 19 | Pertussis | Number | 2021 | 63288.19654 | 125955.2241 | 23766.18181 |
| Incidence | India | Both | 10 to 19 | Pertussis | Rate | 2021 | 23.75090982 | 47.26870619 | 8.919016052 |
| Incidence | Afghanistan | Both | 10 to 19 | Pertussis | Number | 2021 | 1535.704834 | 2897.075228 | 697.4099156 |
| Incidence | Afghanistan | Both | 10 to 19 | Pertussis | Rate | 2021 | 20.22187871 | 38.14815357 | 9.183365454 |
| Incidence | Yemen | Both | 10 to 19 | Pertussis | Number | 2021 | 1685.785825 | 3179.77302 | 765.4730191 |
| Incidence | Yemen | Both | 10 to 19 | Pertussis | Rate | 2021 | 20.7813574 | 39.19833623 | 9.43629265 |
| Incidence | Bhutan | Both | 10 to 19 | Pertussis | Number | 2021 | 1.014841823 | 1.881708504 | 0.426050889 |
| Incidence | Bhutan | Both | 10 to 19 | Pertussis | Rate | 2021 | 0.765589219 | 1.419547077 | 0.321409662 |
| Incidence | Paraguay | Both | 10 to 19 | Pertussis | Number | 2021 | 48.1823835 | 108.6336415 | 12.81431676 |
| Incidence | Paraguay | Both | 10 to 19 | Pertussis | Rate | 2021 | 3.585829007 | 8.084732105 | 0.953666992 |
| Incidence | Algeria | Both | 10 to 19 | Pertussis | Number | 2021 | 594.4756416 | 1130.005543 | 264.0779807 |
| Incidence | Algeria | Both | 10 to 19 | Pertussis | Rate | 2021 | 8.374988999 | 15.91954881 | 3.720337771 |
| Incidence | Qatar | Both | 10 to 19 | Pertussis | Number | 2021 | 1.498760303 | 3.17262922 | 0.502023781 |
| Incidence | Qatar | Both | 10 to 19 | Pertussis | Rate | 2021 | 0.610658067 | 1.292662759 | 0.204545631 |
| Incidence | Iran (Islamic Republic of) | Both | 10 to 19 | Pertussis | Number | 2021 | 1166.560359 | 2192.550071 | 568.8772054 |
| Incidence | Iran (Islamic Republic of) | Both | 10 to 19 | Pertussis | Rate | 2021 | 9.486186158 | 17.82928588 | 4.625971583 |
| Incidence | Congo | Both | 10 to 19 | Pertussis | Number | 2021 | 770.8120825 | 1338.316291 | 393.4753651 |
| Incidence | Congo | Both | 10 to 19 | Pertussis | Rate | 2021 | 64.44526451 | 111.8925732 | 32.89728399 |
| Incidence | Suriname | Both | 10 to 19 | Pertussis | Number | 2021 | 0.304945378 | 1.403450247 | 0.088465136 |
| Incidence | Suriname | Both | 10 to 19 | Pertussis | Rate | 2021 | 0.315656753 | 1.45274721 | 0.091572523 |
| Incidence | Syrian Arab Republic | Both | 10 to 19 | Pertussis | Number | 2021 | 590.1470071 | 1112.739739 | 267.8814458 |
| Incidence | Syrian Arab Republic | Both | 10 to 19 | Pertussis | Rate | 2021 | 17.66785697 | 33.31326996 | 8.019851008 |
| Incidence | Palestine | Both | 10 to 19 | Pertussis | Number | 2021 | 20.57824719 | 39.71078127 | 8.933116872 |
| Incidence | Palestine | Both | 10 to 19 | Pertussis | Rate | 2021 | 1.784059876 | 3.442781635 | 0.774469042 |
| Incidence | Ethiopia | Both | 10 to 19 | Pertussis | Number | 2021 | 13741.85291 | 20055.46884 | 8789.409409 |
| Incidence | Ethiopia | Both | 10 to 19 | Pertussis | Rate | 2021 | 51.45377616 | 75.0939201 | 32.91028563 |
| Incidence | Morocco | Both | 10 to 19 | Pertussis | Number | 2021 | 364.6855077 | 696.2091874 | 159.9582802 |
| Incidence | Morocco | Both | 10 to 19 | Pertussis | Rate | 2021 | 5.724407275 | 10.92827889 | 2.510838308 |
| Incidence | Madagascar | Both | 10 to 19 | Pertussis | Number | 2021 | 2395.895395 | 4676.356988 | 887.4860673 |
| Incidence | Madagascar | Both | 10 to 19 | Pertussis | Rate | 2021 | 34.88322817 | 68.08578879 | 12.92142347 |
| Incidence | Tunisia | Both | 10 to 19 | Pertussis | Number | 2021 | 77.80758296 | 148.6315883 | 34.22431882 |
| Incidence | Tunisia | Both | 10 to 19 | Pertussis | Rate | 2021 | 4.497944411 | 8.592178246 | 1.978458625 |
| Incidence | Nepal | Both | 10 to 19 | Pertussis | Number | 2021 | 972.4608798 | 1878.200466 | 387.5944277 |
| Incidence | Nepal | Both | 10 to 19 | Pertussis | Rate | 2021 | 15.39854339 | 29.7405808 | 6.137408443 |
| Incidence | Malawi | Both | 10 to 19 | Pertussis | Number | 2021 | 687.103108 | 993.5597594 | 435.9516254 |
| Incidence | Malawi | Both | 10 to 19 | Pertussis | Rate | 2021 | 13.27859064 | 19.20100952 | 8.424970145 |
| Incidence | Seychelles | Both | 10 to 19 | Pertussis | Number | 2021 | 0.089973104 | 0.160381743 | 0.04390998 |
| Incidence | Seychelles | Both | 10 to 19 | Pertussis | Rate | 2021 | 0.606470067 | 1.081064475 | 0.295978326 |
| Incidence | Rwanda | Both | 10 to 19 | Pertussis | Number | 2021 | 256.1406311 | 369.3037731 | 162.4220337 |
| Incidence | Rwanda | Both | 10 to 19 | Pertussis | Rate | 2021 | 8.438953904 | 12.1672907 | 5.351248059 |
| Incidence | United Arab Emirates | Both | 10 to 19 | Pertussis | Number | 2021 | 41.42417621 | 79.08030478 | 18.16926324 |
| Incidence | United Arab Emirates | Both | 10 to 19 | Pertussis | Rate | 2021 | 5.371638669 | 10.25465952 | 2.356081061 |
| Incidence | Central African Republic | Both | 10 to 19 | Pertussis | Number | 2021 | 1139.641701 | 2000.561401 | 578.2728832 |
| Incidence | Central African Republic | Both | 10 to 19 | Pertussis | Rate | 2021 | 87.52916035 | 153.651327 | 44.41373099 |
| Incidence | Eritrea | Both | 10 to 19 | Pertussis | Number | 2021 | 231.9613148 | 334.7211759 | 147.9326439 |
| Incidence | Eritrea | Both | 10 to 19 | Pertussis | Rate | 2021 | 15.96245595 | 23.03389266 | 10.18000917 |
| Incidence | Saudi Arabia | Both | 10 to 19 | Pertussis | Number | 2021 | 274.9909144 | 524.4242406 | 120.5285604 |
| Incidence | Saudi Arabia | Both | 10 to 19 | Pertussis | Rate | 2021 | 5.396130665 | 10.29074627 | 2.365124906 |
| Incidence | Equatorial Guinea | Both | 10 to 19 | Pertussis | Number | 2021 | 282.1183429 | 493.3855097 | 143.2254226 |
| Incidence | Equatorial Guinea | Both | 10 to 19 | Pertussis | Rate | 2021 | 73.49675704 | 128.5355449 | 37.31272478 |
| Incidence | Turkey | Both | 10 to 19 | Pertussis | Number | 2021 | 731.6136184 | 1394.10805 | 320.3758062 |
| Incidence | Turkey | Both | 10 to 19 | Pertussis | Rate | 2021 | 5.783241812 | 11.02011193 | 2.532499001 |
| Incidence | Angola | Both | 10 to 19 | Pertussis | Number | 2021 | 7324.195663 | 12887.29699 | 3708.153747 |
| Incidence | Angola | Both | 10 to 19 | Pertussis | Rate | 2021 | 93.76950756 | 164.992246 | 47.47439402 |
| Incidence | Mozambique | Both | 10 to 19 | Pertussis | Number | 2021 | 780.9538852 | 1126.508267 | 495.8225529 |
| Incidence | Mozambique | Both | 10 to 19 | Pertussis | Rate | 2021 | 10.16241866 | 14.65905843 | 6.45205365 |
| Incidence | Pakistan | Both | 10 to 19 | Pertussis | Number | 2021 | 10491.69224 | 15957.70323 | 6359.816678 |
| Incidence | Pakistan | Both | 10 to 19 | Pertussis | Rate | 2021 | 20.75315006 | 31.56522344 | 12.58007068 |
| Incidence | Gabon | Both | 10 to 19 | Pertussis | Number | 2021 | 199.1955131 | 345.0847776 | 102.0755525 |
| Incidence | Gabon | Both | 10 to 19 | Pertussis | Rate | 2021 | 50.39735667 | 87.30799375 | 25.8255718 |
| Incidence | Djibouti | Both | 10 to 19 | Pertussis | Number | 2021 | 134.795579 | 195.9943021 | 86.10068961 |
| Incidence | Djibouti | Both | 10 to 19 | Pertussis | Rate | 2021 | 55.24899567 | 80.33266693 | 35.29030153 |
| Incidence | Mauritius | Both | 10 to 19 | Pertussis | Number | 2021 | 1.261409679 | 2.227529935 | 0.615809043 |
| Incidence | Mauritius | Both | 10 to 19 | Pertussis | Rate | 2021 | 0.760589735 | 1.343129382 | 0.371313178 |
| Incidence | Bangladesh | Both | 10 to 19 | Pertussis | Number | 2021 | 2824.632381 | 5127.736611 | 1337.941827 |
| Incidence | Bangladesh | Both | 10 to 19 | Pertussis | Rate | 2021 | 9.081292674 | 16.48585396 | 4.301530135 |
| Incidence | Comoros | Both | 10 to 19 | Pertussis | Number | 2021 | 38.6305689 | 55.49345521 | 24.87899436 |
| Incidence | Comoros | Both | 10 to 19 | Pertussis | Rate | 2021 | 25.39706101 | 36.48330085 | 16.35630423 |
| Incidence | Somalia | Both | 10 to 19 | Pertussis | Number | 2021 | 4548.599008 | 6721.608234 | 2886.130036 |
| Incidence | Somalia | Both | 10 to 19 | Pertussis | Rate | 2021 | 86.4649663 | 127.7720081 | 54.86285685 |
| Incidence | United Republic of Tanzania | Both | 10 to 19 | Pertussis | Number | 2021 | 2887.453047 | 4349.773571 | 1750.556067 |
| Incidence | United Republic of Tanzania | Both | 10 to 19 | Pertussis | Rate | 2021 | 20.85600906 | 31.41831765 | 12.64422749 |
| Incidence | Democratic Republic of the Congo | Both | 10 to 19 | Pertussis | Number | 2021 | 17211.39278 | 30094.85847 | 8738.42921 |
| Incidence | Democratic Republic of the Congo | Both | 10 to 19 | Pertussis | Rate | 2021 | 80.10466739 | 140.0664466 | 40.67009418 |
| Incidence | Burundi | Both | 10 to 19 | Pertussis | Number | 2021 | 309.1903426 | 446.3248147 | 196.2170793 |
| Incidence | Burundi | Both | 10 to 19 | Pertussis | Rate | 2021 | 9.7989039 | 14.14498891 | 6.218539324 |
| Incidence | Kenya | Both | 10 to 19 | Pertussis | Number | 2021 | 2585.045026 | 3725.224083 | 1672.825998 |
| Incidence | Kenya | Both | 10 to 19 | Pertussis | Rate | 2021 | 20.93432463 | 30.16777252 | 13.54695263 |
| Incidence | Uganda | Both | 10 to 19 | Pertussis | Number | 2021 | 3847.938237 | 8049.291255 | 1426.190889 |
| Incidence | Uganda | Both | 10 to 19 | Pertussis | Rate | 2021 | 34.88398051 | 72.97188832 | 12.92931749 |
| Incidence | Lesotho | Both | 10 to 19 | Pertussis | Number | 2021 | 32.39653064 | 65.58918493 | 12.8695996 |
| Incidence | Lesotho | Both | 10 to 19 | Pertussis | Rate | 2021 | 7.67275998 | 15.53407304 | 3.048022329 |
| Incidence | South Africa | Both | 10 to 19 | Pertussis | Number | 2021 | 1497.72837 | 3021.640076 | 597.918558 |
| Incidence | South Africa | Both | 10 to 19 | Pertussis | Rate | 2021 | 15.3024583 | 30.87243467 | 6.109000794 |
| Incidence | Zambia | Both | 10 to 19 | Pertussis | Number | 2021 | 1146.376325 | 1837.271855 | 646.0191803 |
| Incidence | Zambia | Both | 10 to 19 | Pertussis | Rate | 2021 | 24.28405318 | 38.91951225 | 13.68482913 |
| Incidence | Zimbabwe | Both | 10 to 19 | Pertussis | Number | 2021 | 217.7494501 | 439.6314275 | 85.64411097 |
| Incidence | Zimbabwe | Both | 10 to 19 | Pertussis | Rate | 2021 | 5.966434982 | 12.04610311 | 2.346687991 |
| Incidence | Cabo Verde | Both | 10 to 19 | Pertussis | Number | 2021 | 1.60196453 | 2.493888104 | 0.963824337 |
| Incidence | Cabo Verde | Both | 10 to 19 | Pertussis | Rate | 2021 | 1.597317736 | 2.486654121 | 0.961028587 |
| Incidence | Botswana | Both | 10 to 19 | Pertussis | Number | 2021 | 43.40576201 | 87.92893416 | 17.27704049 |
| Incidence | Botswana | Both | 10 to 19 | Pertussis | Rate | 2021 | 9.691675466 | 19.63284722 | 3.857632299 |
| Incidence | Coted'Ivoire | Both | 10 to 19 | Pertussis | Number | 2021 | 1044.505585 | 1660.730469 | 608.0386439 |
| Incidence | Coted'Ivoire | Both | 10 to 19 | Pertussis | Rate | 2021 | 17.00006866 | 27.02956537 | 9.896259857 |
| Incidence | Namibia | Both | 10 to 19 | Pertussis | Number | 2021 | 30.93318615 | 62.41557308 | 12.18039778 |
| Incidence | Namibia | Both | 10 to 19 | Pertussis | Rate | 2021 | 5.986454069 | 12.07919416 | 2.357254487 |
| Incidence | Liberia | Both | 10 to 19 | Pertussis | Number | 2021 | 231.6383793 | 340.8822204 | 146.2057047 |
| Incidence | Liberia | Both | 10 to 19 | Pertussis | Rate | 2021 | 17.85520767 | 26.27596883 | 11.26986481 |
| Incidence | Eswatini | Both | 10 to 19 | Pertussis | Number | 2021 | 18.97623492 | 38.44840184 | 7.521235993 |
| Incidence | Eswatini | Both | 10 to 19 | Pertussis | Rate | 2021 | 7.414061725 | 15.0218853 | 2.938565428 |
| Incidence | Benin | Both | 10 to 19 | Pertussis | Number | 2021 | 802.9589396 | 1185.379706 | 503.9204246 |
| Incidence | Benin | Both | 10 to 19 | Pertussis | Rate | 2021 | 25.54180597 | 37.70645913 | 16.0295092 |
| Incidence | Cameroon | Both | 10 to 19 | Pertussis | Number | 2021 | 2445.665802 | 4035.845184 | 1254.829749 |
| Incidence | Cameroon | Both | 10 to 19 | Pertussis | Rate | 2021 | 32.65613018 | 53.88924587 | 16.75530795 |
| Incidence | Ghana | Both | 10 to 19 | Pertussis | Number | 2021 | 907.6004217 | 1332.401092 | 573.6984888 |
| Incidence | Ghana | Both | 10 to 19 | Pertussis | Rate | 2021 | 12.4113167 | 18.22041015 | 7.845251574 |
| Incidence | Mauritania | Both | 10 to 19 | Pertussis | Number | 2021 | 393.585214 | 582.6277346 | 245.8008059 |
| Incidence | Mauritania | Both | 10 to 19 | Pertussis | Rate | 2021 | 37.67790239 | 55.77493803 | 23.53050482 |
| Incidence | Chad | Both | 10 to 19 | Pertussis | Number | 2021 | 2631.826338 | 3902.196691 | 1630.365013 |
| Incidence | Chad | Both | 10 to 19 | Pertussis | Rate | 2021 | 60.69365165 | 89.99019546 | 37.59853176 |
| Incidence | Nigeria | Both | 10 to 19 | Pertussis | Number | 2021 | 26684.87283 | 39510.49216 | 16637.59588 |
| Incidence | Nigeria | Both | 10 to 19 | Pertussis | Rate | 2021 | 46.91881716 | 69.4695294 | 29.25313994 |
| Incidence | Burkina Faso | Both | 10 to 19 | Pertussis | Number | 2021 | 662.8724199 | 973.489604 | 418.9307228 |
| Incidence | Burkina Faso | Both | 10 to 19 | Pertussis | Rate | 2021 | 12.49035224 | 18.34324025 | 7.893815058 |
| Incidence | Gambia | Both | 10 to 19 | Pertussis | Number | 2021 | 84.09525405 | 123.59486 | 53.19029386 |
| Incidence | Gambia | Both | 10 to 19 | Pertussis | Rate | 2021 | 14.50807086 | 21.32252297 | 9.176362697 |
| Incidence | Guinea-Bissau | Both | 10 to 19 | Pertussis | Number | 2021 | 164.8079329 | 243.5338899 | 103.1040996 |
| Incidence | Guinea-Bissau | Both | 10 to 19 | Pertussis | Rate | 2021 | 34.06917323 | 50.34344001 | 21.31372786 |
| Incidence | Mali | Both | 10 to 19 | Pertussis | Number | 2021 | 1995.714524 | 2949.68023 | 1248.113216 |
| Incidence | Mali | Both | 10 to 19 | Pertussis | Rate | 2021 | 34.19217717 | 50.53628054 | 21.38367372 |
| Incidence | Senegal | Both | 10 to 19 | Pertussis | Number | 2021 | 570.6331203 | 978.3098276 | 296.5907291 |
| Incidence | Senegal | Both | 10 to 19 | Pertussis | Rate | 2021 | 15.28146234 | 26.19897839 | 7.942651586 |
| Incidence | Guinea | Both | 10 to 19 | Pertussis | Number | 2021 | 1779.906593 | 2635.262459 | 1105.859544 |
| Incidence | Guinea | Both | 10 to 19 | Pertussis | Rate | 2021 | 56.24838075 | 83.27922755 | 34.94723201 |
| Incidence | Niger | Both | 10 to 19 | Pertussis | Number | 2021 | 2754.842088 | 4087.986652 | 1718.944476 |
| Incidence | Niger | Both | 10 to 19 | Pertussis | Rate | 2021 | 43.98318417 | 65.26786801 | 27.44427777 |
| Incidence | Togo | Both | 10 to 19 | Pertussis | Number | 2021 | 779.7009896 | 1267.875897 | 414.6511793 |
| Incidence | Togo | Both | 10 to 19 | Pertussis | Rate | 2021 | 41.57766403 | 67.60965905 | 22.11133198 |
| Incidence | Bermuda | Both | 10 to 19 | Pertussis | Number | 2021 | 0.001674119 | 0.007565841 | 0.000460845 |
| Incidence | Bermuda | Both | 10 to 19 | Pertussis | Rate | 2021 | 0.027108181 | 0.122509926 | 0.007462243 |
| Incidence | Nauru | Both | 10 to 19 | Pertussis | Number | 2021 | 0.006314933 | 0.011382352 | 0.003002505 |
| Incidence | Nauru | Both | 10 to 19 | Pertussis | Rate | 2021 | 0.263074753 | 0.47417916 | 0.12508182 |
| Incidence | Sao Tome and Principe | Both | 10 to 19 | Pertussis | Number | 2021 | 6.035640058 | 8.86997995 | 3.814998316 |
| Incidence | Sao Tome and Principe | Both | 10 to 19 | Pertussis | Rate | 2021 | 11.93047002 | 17.53302531 | 7.5409936 |
| Incidence | Greenland | Both | 10 to 19 | Pertussis | Number | 2021 | 0.133321106 | 0.235345699 | 0.062317907 |
| Incidence | Greenland | Both | 10 to 19 | Pertussis | Rate | 2021 | 1.79902513 | 3.175737422 | 0.84091322 |
| Incidence | American Samoa | Both | 10 to 19 | Pertussis | Number | 2021 | 0.07994909 | 0.141563804 | 0.03904201 |
| Incidence | American Samoa | Both | 10 to 19 | Pertussis | Rate | 2021 | 0.764078303 | 1.352933864 | 0.373126853 |
| Incidence | Monaco | Both | 10 to 19 | Pertussis | Number | 2021 | 0.008028581 | 0.014107876 | 0.003801478 |
| Incidence | Monaco | Both | 10 to 19 | Pertussis | Rate | 2021 | 0.232259018 | 0.408127093 | 0.109973057 |
| Incidence | Northern Mariana Islands | Both | 10 to 19 | Pertussis | Number | 2021 | 0.035602525 | 0.063320702 | 0.017220552 |
| Incidence | Northern Mariana Islands | Both | 10 to 19 | Pertussis | Rate | 2021 | 0.44245179 | 0.786920547 | 0.214009089 |
| Incidence | Sierra Leone | Both | 10 to 19 | Pertussis | Number | 2021 | 399.84473 | 588.373977 | 251.8827476 |
| Incidence | Sierra Leone | Both | 10 to 19 | Pertussis | Rate | 2021 | 19.72141434 | 29.02018237 | 12.42353258 |
| Incidence | Cook Islands | Both | 10 to 19 | Pertussis | Number | 2021 | 0.014491011 | 0.025805917 | 0.007065611 |
| Incidence | Cook Islands | Both | 10 to 19 | Pertussis | Rate | 2021 | 0.515993417 | 0.918892626 | 0.251591049 |
| Incidence | Saint Kitts and Nevis | Both | 10 to 19 | Pertussis | Number | 2021 | 0.005588976 | 0.025458135 | 0.001607649 |
| Incidence | Saint Kitts and Nevis | Both | 10 to 19 | Pertussis | Rate | 2021 | 0.072254058 | 0.329121736 | 0.020783624 |
| Incidence | Tokelau | Both | 10 to 19 | Pertussis | Number | 2021 | 0.001930813 | 0.003418745 | 0.000942601 |
| Incidence | Tokelau | Both | 10 to 19 | Pertussis | Rate | 2021 | 0.719848079 | 1.274580512 | 0.351421548 |
| Incidence | Puerto Rico | Both | 10 to 19 | Pertussis | Number | 2021 | 0.632835852 | 2.888070222 | 0.183760591 |
| Incidence | Puerto Rico | Both | 10 to 19 | Pertussis | Rate | 2021 | 0.158969333 | 0.725487653 | 0.046160941 |
| Incidence | United States Virgin Islands | Both | 10 to 19 | Pertussis | Number | 2021 | 0.039702092 | 0.18397574 | 0.011478389 |
| Incidence | United States Virgin Islands | Both | 10 to 19 | Pertussis | Rate | 2021 | 0.404483208 | 1.874336917 | 0.116941335 |
| Incidence | San Marino | Both | 10 to 19 | Pertussis | Number | 2021 | 0.058198578 | 0.099372519 | 0.028100485 |
| Incidence | San Marino | Both | 10 to 19 | Pertussis | Rate | 2021 | 1.631180536 | 2.785197257 | 0.787595942 |
| Incidence | Guam | Both | 10 to 19 | Pertussis | Number | 2021 | 0.092255538 | 0.164088555 | 0.044445816 |
| Incidence | Guam | Both | 10 to 19 | Pertussis | Rate | 2021 | 0.382638457 | 0.680572601 | 0.184343172 |
| Incidence | Tuvalu | Both | 10 to 19 | Pertussis | Number | 2021 | 0.030116189 | 0.052650211 | 0.014742027 |
| Incidence | Tuvalu | Both | 10 to 19 | Pertussis | Rate | 2021 | 1.26121701 | 2.204905204 | 0.617372099 |
| Incidence | Niue | Both | 10 to 19 | Pertussis | Number | 2021 | 0.001204214 | 0.002153046 | 0.000585248 |
| Incidence | Niue | Both | 10 to 19 | Pertussis | Rate | 2021 | 0.430017025 | 0.768838571 | 0.208988161 |
| Incidence | Palau | Both | 10 to 19 | Pertussis | Number | 2021 | 0.024482402 | 0.04295896 | 0.011931402 |
| Incidence | Palau | Both | 10 to 19 | Pertussis | Rate | 2021 | 1.022286085 | 1.793792405 | 0.498207095 |
| Incidence | Sudan | Both | 10 to 19 | Pertussis | Number | 2021 | 1696.976561 | 3181.251509 | 768.2434301 |
| Incidence | Sudan | Both | 10 to 19 | Pertussis | Rate | 2021 | 16.73477023 | 31.37197899 | 7.576048822 |
| Incidence | South Sudan | Both | 10 to 19 | Pertussis | Number | 2021 | 1411.379978 | 2053.261338 | 901.6645311 |
| Incidence | South Sudan | Both | 10 to 19 | Pertussis | Rate | 2021 | 55.25755739 | 80.3881364 | 35.30146405 |

Sup_Table 6: Deaths of Adolescent Pertussis in 204 Countries and Territories in 2021

| measure | location | sex | age | cause | metric | year | val | upper | lower |
| --- | --- | --- | --- | --- | --- | --- | --- | --- | --- |
| Deaths | Congo | Both | 10 to 19 | Pertussis | Number | 2021 | 6.004620766 | 22.12207588 | 0.555222825 |
| Deaths | Congo | Both | 10 to 19 | Pertussis | Rate | 2021 | 0.502028163 | 1.849559788 | 0.046420499 |
| Deaths | Monaco | Both | 10 to 19 | Pertussis | Number | 2021 | 1.66E-05 | 7.10E-05 | 0 |
| Deaths | Monaco | Both | 10 to 19 | Pertussis | Rate | 2021 | 0.000481066 | 0.00205261 | 0 |
| Deaths | New Zealand | Both | 10 to 19 | Pertussis | Number | 2021 | 9.98E-07 | 3.22E-06 | 1.33E-07 |
| Deaths | New Zealand | Both | 10 to 19 | Pertussis | Rate | 2021 | 1.51E-07 | 4.89E-07 | 2.01E-08 |
| Deaths | Bolivia (Plurinational State of) | Both | 10 to 19 | Pertussis | Number | 2021 | 0.117214182 | 0.51296081 | 0 |
| Deaths | Bolivia (Plurinational State of) | Both | 10 to 19 | Pertussis | Rate | 2021 | 0.00538569 | 0.023569227 | 0 |
| Deaths | Chad | Both | 10 to 19 | Pertussis | Number | 2021 | 26.88545021 | 86.43328813 | 1.510754192 |
| Deaths | Chad | Both | 10 to 19 | Pertussis | Rate | 2021 | 0.62001665 | 1.99327433 | 0.034840136 |
| Deaths | Antigua and Barbuda | Both | 10 to 19 | Pertussis | Number | 2021 | 4.41E-07 | 1.75E-06 | 0 |
| Deaths | Antigua and Barbuda | Both | 10 to 19 | Pertussis | Rate | 2021 | 3.51E-06 | 1.39E-05 | 0 |
| Deaths | Malta | Both | 10 to 19 | Pertussis | Number | 2021 | 1.15E-06 | 3.66E-06 | 1.49E-07 |
| Deaths | Malta | Both | 10 to 19 | Pertussis | Rate | 2021 | 2.84E-06 | 9.04E-06 | 3.69E-07 |
| Deaths | Micronesia (Federated States of) | Both | 10 to 19 | Pertussis | Number | 2021 | 0.117433484 | 0.353633657 | 0.011444069 |
| Deaths | Micronesia (Federated States of) | Both | 10 to 19 | Pertussis | Rate | 2021 | 0.53997587 | 1.626057878 | 0.052621458 |
| Deaths | Kiribati | Both | 10 to 19 | Pertussis | Number | 2021 | 0.407347017 | 1.250137827 | 0.037140402 |
| Deaths | Kiribati | Both | 10 to 19 | Pertussis | Rate | 2021 | 1.638511982 | 5.028552375 | 0.149393491 |
| Deaths | Tunisia | Both | 10 to 19 | Pertussis | Number | 2021 | 0.003866215 | 0.058670184 | 0 |
| Deaths | Tunisia | Both | 10 to 19 | Pertussis | Rate | 2021 | 0.0002235 | 0.003391639 | 0 |
| Deaths | Belarus | Both | 10 to 19 | Pertussis | Number | 2021 | 7.44E-06 | 2.88E-05 | 5.02E-07 |
| Deaths | Belarus | Both | 10 to 19 | Pertussis | Rate | 2021 | 7.61E-07 | 2.94E-06 | 5.14E-08 |
| Deaths | Portugal | Both | 10 to 19 | Pertussis | Number | 2021 | 6.37E-06 | 2.57E-05 | 0 |
| Deaths | Portugal | Both | 10 to 19 | Pertussis | Rate | 2021 | 6.01E-07 | 2.42E-06 | 0 |
| Deaths | Estonia | Both | 10 to 19 | Pertussis | Number | 2021 | 1.83E-07 | 5.75E-07 | 3.82E-09 |
| Deaths | Estonia | Both | 10 to 19 | Pertussis | Rate | 2021 | 1.30E-07 | 4.10E-07 | 2.72E-09 |
| Deaths | Uganda | Both | 10 to 19 | Pertussis | Number | 2021 | 35.87434292 | 154.0190813 | 2.414826169 |
| Deaths | Uganda | Both | 10 to 19 | Pertussis | Rate | 2021 | 0.325223484 | 1.396279852 | 0.021891918 |
| Deaths | United Arab Emirates | Both | 10 to 19 | Pertussis | Number | 2021 | 0.01361073 | 0.058692309 | 0 |
| Deaths | United Arab Emirates | Both | 10 to 19 | Pertussis | Rate | 2021 | 0.001764958 | 0.007610867 | 0 |
| Deaths | Fiji | Both | 10 to 19 | Pertussis | Number | 2021 | 0.010558428 | 0.044579207 | 9.34E-05 |
| Deaths | Fiji | Both | 10 to 19 | Pertussis | Rate | 2021 | 0.006344452 | 0.026787195 | 5.61E-05 |
| Deaths | Uzbekistan | Both | 10 to 19 | Pertussis | Number | 2021 | 5.72E-05 | 0.000160533 | 1.00E-05 |
| Deaths | Uzbekistan | Both | 10 to 19 | Pertussis | Rate | 2021 | 1.06E-06 | 2.97E-06 | 1.86E-07 |
| Deaths | United Republic of Tanzania | Both | 10 to 19 | Pertussis | Number | 2021 | 27.60297557 | 98.0081278 | 2.288660004 |
| Deaths | United Republic of Tanzania | Both | 10 to 19 | Pertussis | Rate | 2021 | 0.199375678 | 0.707910525 | 0.01653094 |
| Deaths | Bosnia and Herzegovina | Both | 10 to 19 | Pertussis | Number | 2021 | 0.055543126 | 0.197848556 | 0.003409875 |
| Deaths | Bosnia and Herzegovina | Both | 10 to 19 | Pertussis | Rate | 2021 | 0.015977377 | 0.056912549 | 0.000980875 |
| Deaths | Democratic People's Republic of Korea | Both | 10 to 19 | Pertussis | Number | 2021 | 0.065764257 | 0.248611934 | 0.003907255 |
| Deaths | Democratic People's Republic of Korea | Both | 10 to 19 | Pertussis | Rate | 2021 | 0.001871964 | 0.00707668 | 0.000111219 |
| Deaths | Luxembourg | Both | 10 to 19 | Pertussis | Number | 2021 | 1.40E-07 | 4.18E-07 | 1.64E-08 |
| Deaths | Luxembourg | Both | 10 to 19 | Pertussis | Rate | 2021 | 2.06E-07 | 6.15E-07 | 2.42E-08 |
| Deaths | Andorra | Both | 10 to 19 | Pertussis | Number | 2021 | 4.17E-05 | 0.000134619 | 3.84E-06 |
| Deaths | Andorra | Both | 10 to 19 | Pertussis | Rate | 2021 | 0.000492035 | 0.00158732 | 4.52E-05 |
| Deaths | Cambodia | Both | 10 to 19 | Pertussis | Number | 2021 | 0.20386632 | 1.088675971 | 0.005766438 |
| Deaths | Cambodia | Both | 10 to 19 | Pertussis | Rate | 2021 | 0.006438717 | 0.034383688 | 0.000182122 |
| Deaths | Nauru | Both | 10 to 19 | Pertussis | Number | 2021 | 0.001294396 | 0.004653185 | 0.000127589 |
| Deaths | Nauru | Both | 10 to 19 | Pertussis | Rate | 2021 | 0.053923438 | 0.193847748 | 0.005315233 |
| Deaths | Bahrain | Both | 10 to 19 | Pertussis | Number | 2021 | 0.035456728 | 0.108006397 | 0.003458682 |
| Deaths | Bahrain | Both | 10 to 19 | Pertussis | Rate | 2021 | 0.017131176 | 0.052184077 | 0.001671088 |
| Deaths | Seychelles | Both | 10 to 19 | Pertussis | Number | 2021 | 0.00031818 | 0.001422129 | 0 |
| Deaths | Seychelles | Both | 10 to 19 | Pertussis | Rate | 2021 | 0.002144714 | 0.009585962 | 0 |
| Deaths | Iran (Islamic Republic of) | Both | 10 to 19 | Pertussis | Number | 2021 | 2.774800698 | 9.16655914 | 0.256962832 |
| Deaths | Iran (Islamic Republic of) | Both | 10 to 19 | Pertussis | Rate | 2021 | 0.022564007 | 0.074540238 | 0.002089559 |
| Deaths | Belize | Both | 10 to 19 | Pertussis | Number | 2021 | 2.02E-07 | 9.97E-07 | 0 |
| Deaths | Belize | Both | 10 to 19 | Pertussis | Rate | 2021 | 2.25E-07 | 1.11E-06 | 0 |
| Deaths | Indonesia | Both | 10 to 19 | Pertussis | Number | 2021 | 16.07429482 | 70.22207646 | 0.760289945 |
| Deaths | Indonesia | Both | 10 to 19 | Pertussis | Rate | 2021 | 0.035149882 | 0.153555582 | 0.001662536 |
| Deaths | Turkmenistan | Both | 10 to 19 | Pertussis | Number | 2021 | 0.000601611 | 0.001829681 | 6.77E-05 |
| Deaths | Turkmenistan | Both | 10 to 19 | Pertussis | Rate | 2021 | 6.64E-05 | 0.000201981 | 7.47E-06 |
| Deaths | Syrian Arab Republic | Both | 10 to 19 | Pertussis | Number | 2021 | 3.617635105 | 11.47969499 | 0.304677405 |
| Deaths | Syrian Arab Republic | Both | 10 to 19 | Pertussis | Rate | 2021 | 0.10830498 | 0.343679806 | 0.009121451 |
| Deaths | Belgium | Both | 10 to 19 | Pertussis | Number | 2021 | 2.71E-05 | 7.42E-05 | 3.74E-06 |
| Deaths | Belgium | Both | 10 to 19 | Pertussis | Rate | 2021 | 2.08E-06 | 5.69E-06 | 2.87E-07 |
| Deaths | Albania | Both | 10 to 19 | Pertussis | Number | 2021 | 0.000862847 | 0.002895515 | 2.19E-05 |
| Deaths | Albania | Both | 10 to 19 | Pertussis | Rate | 2021 | 0.000264908 | 0.000888969 | 6.74E-06 |
| Deaths | Papua New Guinea | Both | 10 to 19 | Pertussis | Number | 2021 | 0.000536145 | 0 | 0 |
| Deaths | Papua New Guinea | Both | 10 to 19 | Pertussis | Rate | 2021 | 2.55E-05 | 0 | 0 |
| Deaths | Democratic Republic of the Congo | Both | 10 to 19 | Pertussis | Number | 2021 | 142.5084414 | 534.7225479 | 6.824926637 |
| Deaths | Democratic Republic of the Congo | Both | 10 to 19 | Pertussis | Rate | 2021 | 0.663257846 | 2.488687138 | 0.031764337 |
| Deaths | Bahamas | Both | 10 to 19 | Pertussis | Number | 2021 | 1.05E-06 | 3.72E-06 | 4.99E-08 |
| Deaths | Bahamas | Both | 10 to 19 | Pertussis | Rate | 2021 | 1.54E-06 | 5.46E-06 | 7.33E-08 |
| Deaths | Algeria | Both | 10 to 19 | Pertussis | Number | 2021 | 2.31279937 | 7.692636474 | 0.205413506 |
| Deaths | Algeria | Both | 10 to 19 | Pertussis | Rate | 2021 | 0.03258278 | 0.108374072 | 0.002893871 |
| Deaths | Somalia | Both | 10 to 19 | Pertussis | Number | 2021 | 68.03541794 | 205.7405557 | 5.250319225 |
| Deaths | Somalia | Both | 10 to 19 | Pertussis | Rate | 2021 | 1.293294948 | 3.910951523 | 0.099804066 |
| Deaths | Guinea-Bissau | Both | 10 to 19 | Pertussis | Number | 2021 | 2.238231862 | 8.280939841 | 0.144462435 |
| Deaths | Guinea-Bissau | Both | 10 to 19 | Pertussis | Rate | 2021 | 0.462688341 | 1.71183977 | 0.029863342 |
| Deaths | Ghana | Both | 10 to 19 | Pertussis | Number | 2021 | 6.678959507 | 21.49094489 | 0.432343759 |
| Deaths | Ghana | Both | 10 to 19 | Pertussis | Rate | 2021 | 0.091333895 | 0.293885852 | 0.005912244 |
| Deaths | Equatorial Guinea | Both | 10 to 19 | Pertussis | Number | 2021 | 1.586334715 | 5.692451964 | 0.096151636 |
| Deaths | Equatorial Guinea | Both | 10 to 19 | Pertussis | Rate | 2021 | 0.413267907 | 1.482983186 | 0.025049181 |
| Deaths | Austria | Both | 10 to 19 | Pertussis | Number | 2021 | 1.47E-06 | 4.57E-06 | 1.80E-07 |
| Deaths | Austria | Both | 10 to 19 | Pertussis | Rate | 2021 | 1.65E-07 | 5.14E-07 | 2.03E-08 |
| Deaths | Gabon | Both | 10 to 19 | Pertussis | Number | 2021 | 1.302199407 | 4.250839624 | 0.09916045 |
| Deaths | Gabon | Both | 10 to 19 | Pertussis | Rate | 2021 | 0.32946228 | 1.075481457 | 0.025088038 |
| Deaths | Lao People's Democratic Republic | Both | 10 to 19 | Pertussis | Number | 2021 | 0.351316026 | 1.593236336 | 0.009523292 |
| Deaths | Lao People's Democratic Republic | Both | 10 to 19 | Pertussis | Rate | 2021 | 0.025034231 | 0.113531532 | 0.000678615 |
| Deaths | Latvia | Both | 10 to 19 | Pertussis | Number | 2021 | 9.18E-08 | 3.87E-07 | 0 |
| Deaths | Latvia | Both | 10 to 19 | Pertussis | Rate | 2021 | 4.83E-08 | 2.03E-07 | 0 |
| Deaths | Cuba | Both | 10 to 19 | Pertussis | Number | 2021 | 7.66E-07 | 3.07E-06 | 2.40E-09 |
| Deaths | Cuba | Both | 10 to 19 | Pertussis | Rate | 2021 | 6.18E-08 | 2.48E-07 | 1.94E-10 |
| Deaths | Cyprus | Both | 10 to 19 | Pertussis | Number | 2021 | 0.001010468 | 0.004064364 | 1.66E-05 |
| Deaths | Cyprus | Both | 10 to 19 | Pertussis | Rate | 2021 | 0.000737586 | 0.002966762 | 1.21E-05 |
| Deaths | Burundi | Both | 10 to 19 | Pertussis | Number | 2021 | 3.343181948 | 11.03068892 | 0.237162048 |
| Deaths | Burundi | Both | 10 to 19 | Pertussis | Rate | 2021 | 0.105952593 | 0.349586147 | 0.007516173 |
| Deaths | Ecuador | Both | 10 to 19 | Pertussis | Number | 2021 | 0.075802425 | 0.201392346 | 0.018425916 |
| Deaths | Ecuador | Both | 10 to 19 | Pertussis | Rate | 2021 | 0.002339182 | 0.006214754 | 0.000568604 |
| Deaths | Egypt | Both | 10 to 19 | Pertussis | Number | 2021 | 5.021626762 | 19.92828146 | 0.234043523 |
| Deaths | Egypt | Both | 10 to 19 | Pertussis | Rate | 2021 | 0.024151884 | 0.095846539 | 0.00112565 |
| Deaths | Northern Mariana Islands | Both | 10 to 19 | Pertussis | Number | 2021 | 0.000241414 | 0.000855267 | 1.73E-05 |
| Deaths | Northern Mariana Islands | Both | 10 to 19 | Pertussis | Rate | 2021 | 0.00300018 | 0.010628867 | 0.000214498 |
| Deaths | Russian Federation | Both | 10 to 19 | Pertussis | Number | 2021 | 0.002485445 | 0.007201567 | 0.000216966 |
| Deaths | Russian Federation | Both | 10 to 19 | Pertussis | Rate | 2021 | 1.49E-05 | 4.33E-05 | 1.30E-06 |
| Deaths | Vanuatu | Both | 10 to 19 | Pertussis | Number | 2021 | 0.001737463 | 0.006516794 | 4.05E-06 |
| Deaths | Vanuatu | Both | 10 to 19 | Pertussis | Rate | 2021 | 0.002652225 | 0.009947841 | 6.18E-06 |
| Deaths | Yemen | Both | 10 to 19 | Pertussis | Number | 2021 | 5.81023955 | 25.5217696 | 0.138504935 |
| Deaths | Yemen | Both | 10 to 19 | Pertussis | Rate | 2021 | 0.071625151 | 0.314617081 | 0.001707406 |
| Deaths | Taiwan (Province of China) | Both | 10 to 19 | Pertussis | Number | 2021 | 1.38E-05 | 5.37E-05 | 8.08E-07 |
| Deaths | Taiwan (Province of China) | Both | 10 to 19 | Pertussis | Rate | 2021 | 6.63E-07 | 2.58E-06 | 3.88E-08 |
| Deaths | Spain | Both | 10 to 19 | Pertussis | Number | 2021 | 0.000173237 | 0.000776973 | 0 |
| Deaths | Spain | Both | 10 to 19 | Pertussis | Rate | 2021 | 3.62E-06 | 1.63E-05 | 0 |
| Deaths | Zambia | Both | 10 to 19 | Pertussis | Number | 2021 | 11.35605282 | 38.2882832 | 0.926526414 |
| Deaths | Zambia | Both | 10 to 19 | Pertussis | Rate | 2021 | 0.240558868 | 0.811072843 | 0.019626903 |
| Deaths | Colombia | Both | 10 to 19 | Pertussis | Number | 2021 | 0.016876647 | 0.047275479 | 0.003267264 |
| Deaths | Colombia | Both | 10 to 19 | Pertussis | Rate | 2021 | 0.000221618 | 0.000620805 | 4.29E-05 |
| Deaths | United Kingdom | Both | 10 to 19 | Pertussis | Number | 2021 | 0.000667973 | 0.001319733 | 0.000258032 |
| Deaths | United Kingdom | Both | 10 to 19 | Pertussis | Rate | 2021 | 8.37E-06 | 1.65E-05 | 3.23E-06 |
| Deaths | Bulgaria | Both | 10 to 19 | Pertussis | Number | 2021 | 1.76E-06 | 5.34E-06 | 1.05E-07 |
| Deaths | Bulgaria | Both | 10 to 19 | Pertussis | Rate | 2021 | 2.65E-07 | 8.01E-07 | 1.58E-08 |
| Deaths | Republic of Moldova | Both | 10 to 19 | Pertussis | Number | 2021 | 3.82E-07 | 1.15E-06 | 2.78E-08 |
| Deaths | Republic of Moldova | Both | 10 to 19 | Pertussis | Rate | 2021 | 1.06E-07 | 3.20E-07 | 7.71E-09 |
| Deaths | Costa Rica | Both | 10 to 19 | Pertussis | Number | 2021 | 1.45E-06 | 5.63E-06 | 0 |
| Deaths | Costa Rica | Both | 10 to 19 | Pertussis | Rate | 2021 | 2.03E-07 | 7.92E-07 | 0 |
| Deaths | Lithuania | Both | 10 to 19 | Pertussis | Number | 2021 | 7.44E-06 | 2.22E-05 | 1.40E-06 |
| Deaths | Lithuania | Both | 10 to 19 | Pertussis | Rate | 2021 | 2.83E-06 | 8.46E-06 | 5.34E-07 |
| Deaths | Dominica | Both | 10 to 19 | Pertussis | Number | 2021 | 5.31E-05 | 0.000208504 | 0 |
| Deaths | Dominica | Both | 10 to 19 | Pertussis | Rate | 2021 | 0.00047245 | 0.0018549 | 0 |
| Deaths | Ukraine | Both | 10 to 19 | Pertussis | Number | 2021 | 5.76E-05 | 0.000347191 | 0 |
| Deaths | Ukraine | Both | 10 to 19 | Pertussis | Rate | 2021 | 1.26E-06 | 7.58E-06 | 0 |
| Deaths | Bangladesh | Both | 10 to 19 | Pertussis | Number | 2021 | 24.43856667 | 75.44644713 | 2.676368575 |
| Deaths | Bangladesh | Both | 10 to 19 | Pertussis | Rate | 2021 | 0.078570853 | 0.242562987 | 0.008604619 |
| Deaths | Iraq | Both | 10 to 19 | Pertussis | Number | 2021 | 0.86585439 | 5.564834309 | 0 |
| Deaths | Iraq | Both | 10 to 19 | Pertussis | Rate | 2021 | 0.009953651 | 0.063971976 | 0 |
| Deaths | Samoa | Both | 10 to 19 | Pertussis | Number | 2021 | 0.298734239 | 0.902327656 | 0.028480095 |
| Deaths | Samoa | Both | 10 to 19 | Pertussis | Rate | 2021 | 0.664580545 | 2.00736751 | 0.063358379 |
| Deaths | Peru | Both | 10 to 19 | Pertussis | Number | 2021 | 6.152279484 | 24.56315344 | 0.397212494 |
| Deaths | Peru | Both | 10 to 19 | Pertussis | Rate | 2021 | 0.102393854 | 0.408810419 | 0.006610902 |
| Deaths | Denmark | Both | 10 to 19 | Pertussis | Number | 2021 | 1.11E-07 | 6.21E-07 | 0 |
| Deaths | Denmark | Both | 10 to 19 | Pertussis | Rate | 2021 | 1.64E-08 | 9.17E-08 | 0 |
| Deaths | Palau | Both | 10 to 19 | Pertussis | Number | 2021 | 0.002461987 | 0.008075201 | 0.000210596 |
| Deaths | Palau | Both | 10 to 19 | Pertussis | Rate | 2021 | 0.102802636 | 0.337187732 | 0.00879365 |
| Deaths | Sri Lanka | Both | 10 to 19 | Pertussis | Number | 2021 | 0.040342056 | 0.190368352 | 0.000517575 |
| Deaths | Sri Lanka | Both | 10 to 19 | Pertussis | Rate | 2021 | 0.001114429 | 0.005258832 | 1.43E-05 |
| Deaths | Netherlands | Both | 10 to 19 | Pertussis | Number | 2021 | 0.00060501 | 0.002619016 | 4.00E-06 |
| Deaths | Netherlands | Both | 10 to 19 | Pertussis | Rate | 2021 | 3.11E-05 | 0.000134626 | 2.06E-07 |
| Deaths | Jordan | Both | 10 to 19 | Pertussis | Number | 2021 | 0.771948068 | 2.548914223 | 0.077679633 |
| Deaths | Jordan | Both | 10 to 19 | Pertussis | Rate | 2021 | 0.029564693 | 0.097620382 | 0.002975038 |
| Deaths | Turkey | Both | 10 to 19 | Pertussis | Number | 2021 | 2.131704162 | 7.091952784 | 0.199823032 |
| Deaths | Turkey | Both | 10 to 19 | Pertussis | Rate | 2021 | 0.016850644 | 0.056060299 | 0.001579556 |
| Deaths | Marshall Islands | Both | 10 to 19 | Pertussis | Number | 2021 | 0.070213399 | 0.223685721 | 0.007033215 |
| Deaths | Marshall Islands | Both | 10 to 19 | Pertussis | Rate | 2021 | 0.607846673 | 1.936476839 | 0.060887473 |
| Deaths | Namibia | Both | 10 to 19 | Pertussis | Number | 2021 | 0.225131263 | 0.816317474 | 0.012252844 |
| Deaths | Namibia | Both | 10 to 19 | Pertussis | Rate | 2021 | 0.043569323 | 0.157980721 | 0.002371275 |
| Deaths | Comoros | Both | 10 to 19 | Pertussis | Number | 2021 | 0.371651544 | 1.130712501 | 0.029124487 |
| Deaths | Comoros | Both | 10 to 19 | Pertussis | Rate | 2021 | 0.244336473 | 0.743369181 | 0.019147437 |
| Deaths | Hungary | Both | 10 to 19 | Pertussis | Number | 2021 | 1.17E-06 | 4.06E-06 | 9.92E-09 |
| Deaths | Hungary | Both | 10 to 19 | Pertussis | Rate | 2021 | 1.21E-07 | 4.22E-07 | 1.03E-09 |
| Deaths | Dominican Republic | Both | 10 to 19 | Pertussis | Number | 2021 | 0.344700285 | 1.482416304 | 0 |
| Deaths | Dominican Republic | Both | 10 to 19 | Pertussis | Rate | 2021 | 0.018309407 | 0.078741342 | 0 |
| Deaths | Libya | Both | 10 to 19 | Pertussis | Number | 2021 | 0.486570594 | 1.483731732 | 0.046550175 |
| Deaths | Libya | Both | 10 to 19 | Pertussis | Rate | 2021 | 0.041807862 | 0.127487463 | 0.003999755 |
| Deaths | Sweden | Both | 10 to 19 | Pertussis | Number | 2021 | 0.000240471 | 0.000847541 | 1.85E-05 |
| Deaths | Sweden | Both | 10 to 19 | Pertussis | Rate | 2021 | 1.97E-05 | 6.95E-05 | 1.51E-06 |
| Deaths | Myanmar | Both | 10 to 19 | Pertussis | Number | 2021 | 1.227208213 | 4.976337 | 0.035236242 |
| Deaths | Myanmar | Both | 10 to 19 | Pertussis | Rate | 2021 | 0.011859518 | 0.04809042 | 0.000340517 |
| Deaths | Lesotho | Both | 10 to 19 | Pertussis | Number | 2021 | 0.142823341 | 0.877557261 | 0 |
| Deaths | Lesotho | Both | 10 to 19 | Pertussis | Rate | 2021 | 0.033826129 | 0.207839732 | 0 |
| Deaths | Barbados | Both | 10 to 19 | Pertussis | Number | 2021 | 1.08E-06 | 7.36E-06 | 0 |
| Deaths | Barbados | Both | 10 to 19 | Pertussis | Rate | 2021 | 2.88E-06 | 1.97E-05 | 0 |
| Deaths | Georgia | Both | 10 to 19 | Pertussis | Number | 2021 | 9.75E-05 | 0.000268405 | 1.78E-05 |
| Deaths | Georgia | Both | 10 to 19 | Pertussis | Rate | 2021 | 2.30E-05 | 6.32E-05 | 4.19E-06 |
| Deaths | Czechia | Both | 10 to 19 | Pertussis | Number | 2021 | 1.83E-05 | 6.37E-05 | 4.11E-09 |
| Deaths | Czechia | Both | 10 to 19 | Pertussis | Rate | 2021 | 1.67E-06 | 5.81E-06 | 3.75E-10 |
| Deaths | South Africa | Both | 10 to 19 | Pertussis | Number | 2021 | 13.07799217 | 39.3145301 | 1.08689224 |
| Deaths | South Africa | Both | 10 to 19 | Pertussis | Rate | 2021 | 0.133619309 | 0.401680952 | 0.0111049 |
| Deaths | Norway | Both | 10 to 19 | Pertussis | Number | 2021 | 1.42E-05 | 3.74E-05 | 3.00E-06 |
| Deaths | Norway | Both | 10 to 19 | Pertussis | Rate | 2021 | 2.20E-06 | 5.78E-06 | 4.64E-07 |
| Deaths | Maldives | Both | 10 to 19 | Pertussis | Number | 2021 | 0.007540904 | 0.025311288 | 0.000677973 |
| Deaths | Maldives | Both | 10 to 19 | Pertussis | Rate | 2021 | 0.011841293 | 0.039745681 | 0.001064604 |
| Deaths | Bhutan | Both | 10 to 19 | Pertussis | Number | 2021 | 0.007636795 | 0.027122625 | 0.000701661 |
| Deaths | Bhutan | Both | 10 to 19 | Pertussis | Rate | 2021 | 0.005761142 | 0.020461109 | 0.000529328 |
| Deaths | Kazakhstan | Both | 10 to 19 | Pertussis | Number | 2021 | 2.57E-05 | 9.95E-05 | 2.14E-06 |
| Deaths | Kazakhstan | Both | 10 to 19 | Pertussis | Rate | 2021 | 8.72E-07 | 3.38E-06 | 7.27E-08 |
| Deaths | Djibouti | Both | 10 to 19 | Pertussis | Number | 2021 | 1.532377161 | 5.005115421 | 0.131608351 |
| Deaths | Djibouti | Both | 10 to 19 | Pertussis | Rate | 2021 | 0.628079198 | 2.051458974 | 0.053942639 |
| Deaths | Argentina | Both | 10 to 19 | Pertussis | Number | 2021 | 0.002413417 | 0.007645047 | 0.000373175 |
| Deaths | Argentina | Both | 10 to 19 | Pertussis | Rate | 2021 | 3.41E-05 | 0.000108058 | 5.27E-06 |
| Deaths | Montenegro | Both | 10 to 19 | Pertussis | Number | 2021 | 0.002803887 | 0.011308631 | 0.000141835 |
| Deaths | Montenegro | Both | 10 to 19 | Pertussis | Rate | 2021 | 0.003636071 | 0.014664996 | 0.000183931 |
| Deaths | Croatia | Both | 10 to 19 | Pertussis | Number | 2021 | 2.59E-06 | 9.98E-06 | 1.34E-11 |
| Deaths | Croatia | Both | 10 to 19 | Pertussis | Rate | 2021 | 6.08E-07 | 2.34E-06 | 3.15E-12 |
| Deaths | Greece | Both | 10 to 19 | Pertussis | Number | 2021 | 7.35E-06 | 3.51E-05 | 1.96E-08 |
| Deaths | Greece | Both | 10 to 19 | Pertussis | Rate | 2021 | 7.10E-07 | 3.39E-06 | 1.90E-09 |
| Deaths | North Macedonia | Both | 10 to 19 | Pertussis | Number | 2021 | 0.008626822 | 0.04256121 | 0 |
| Deaths | North Macedonia | Both | 10 to 19 | Pertussis | Rate | 2021 | 0.003704843 | 0.01827818 | 0 |
| Deaths | Morocco | Both | 10 to 19 | Pertussis | Number | 2021 | 2.324154818 | 7.314641254 | 0.168166544 |
| Deaths | Morocco | Both | 10 to 19 | Pertussis | Rate | 2021 | 0.036481869 | 0.114816697 | 0.002639682 |
| Deaths | Mali | Both | 10 to 19 | Pertussis | Number | 2021 | 16.25853225 | 59.61841255 | 1.146507234 |
| Deaths | Mali | Both | 10 to 19 | Pertussis | Rate | 2021 | 0.278554176 | 1.02143032 | 0.019642879 |
| Deaths | Finland | Both | 10 to 19 | Pertussis | Number | 2021 | 3.53E-07 | 1.05E-06 | 6.25E-08 |
| Deaths | Finland | Both | 10 to 19 | Pertussis | Rate | 2021 | 5.75E-08 | 1.71E-07 | 1.02E-08 |
| Deaths | Honduras | Both | 10 to 19 | Pertussis | Number | 2021 | 1.508001599 | 6.223769055 | 0.069207063 |
| Deaths | Honduras | Both | 10 to 19 | Pertussis | Rate | 2021 | 0.069795946 | 0.288059274 | 0.003203161 |
| Deaths | Bermuda | Both | 10 to 19 | Pertussis | Number | 2021 | 1.22E-11 | 0 | 0 |
| Deaths | Bermuda | Both | 10 to 19 | Pertussis | Rate | 2021 | 1.97E-10 | 0 | 0 |
| Deaths | Iceland | Both | 10 to 19 | Pertussis | Number | 2021 | 0.000205476 | 0.000567668 | 4.47E-05 |
| Deaths | Iceland | Both | 10 to 19 | Pertussis | Rate | 2021 | 0.000455002 | 0.001257031 | 9.90E-05 |
| Deaths | Nigeria | Both | 10 to 19 | Pertussis | Number | 2021 | 162.9669481 | 544.6150549 | 10.86631434 |
| Deaths | Nigeria | Both | 10 to 19 | Pertussis | Rate | 2021 | 0.286537489 | 0.957572268 | 0.019105754 |
| Deaths | Lebanon | Both | 10 to 19 | Pertussis | Number | 2021 | 0.090317268 | 0.452234042 | 0.003355928 |
| Deaths | Lebanon | Both | 10 to 19 | Pertussis | Rate | 2021 | 0.011046836 | 0.055313403 | 0.000410468 |
| Deaths | Sudan | Both | 10 to 19 | Pertussis | Number | 2021 | 5.79080318 | 24.52665427 | 0.32187392 |
| Deaths | Sudan | Both | 10 to 19 | Pertussis | Rate | 2021 | 0.057106128 | 0.241870119 | 0.003174166 |
| Deaths | Cook Islands | Both | 10 to 19 | Pertussis | Number | 2021 | 0.000798874 | 0.002491074 | 7.17E-05 |
| Deaths | Cook Islands | Both | 10 to 19 | Pertussis | Rate | 2021 | 0.028446174 | 0.08870172 | 0.002553343 |
| Deaths | Philippines | Both | 10 to 19 | Pertussis | Number | 2021 | 10.76323831 | 41.89760223 | 0.720679551 |
| Deaths | Philippines | Both | 10 to 19 | Pertussis | Rate | 2021 | 0.048603837 | 0.189198098 | 0.003254391 |
| Deaths | Republic of Korea | Both | 10 to 19 | Pertussis | Number | 2021 | 0.021515017 | 0.096546203 | 0 |
| Deaths | Republic of Korea | Both | 10 to 19 | Pertussis | Rate | 2021 | 0.000464904 | 0.002086202 | 0 |
| Deaths | Guinea | Both | 10 to 19 | Pertussis | Number | 2021 | 16.23262453 | 58.53095618 | 1.313225122 |
| Deaths | Guinea | Both | 10 to 19 | Pertussis | Rate | 2021 | 0.512981326 | 1.849687799 | 0.041500373 |
| Deaths | Saint Kitts and Nevis | Both | 10 to 19 | Pertussis | Number | 2021 | 1.77E-08 | 9.85E-08 | 0 |
| Deaths | Saint Kitts and Nevis | Both | 10 to 19 | Pertussis | Rate | 2021 | 2.28E-07 | 1.27E-06 | 0 |
| Deaths | Germany | Both | 10 to 19 | Pertussis | Number | 2021 | 3.34E-05 | 0.000177761 | 0 |
| Deaths | Germany | Both | 10 to 19 | Pertussis | Rate | 2021 | 4.25E-07 | 2.26E-06 | 0 |
| Deaths | Haiti | Both | 10 to 19 | Pertussis | Number | 2021 | 0.040619448 | 0.252555497 | 0 |
| Deaths | Haiti | Both | 10 to 19 | Pertussis | Rate | 2021 | 0.001566356 | 0.009738976 | 0 |
| Deaths | Azerbaijan | Both | 10 to 19 | Pertussis | Number | 2021 | 0.828678093 | 2.460170306 | 0.078626708 |
| Deaths | Azerbaijan | Both | 10 to 19 | Pertussis | Rate | 2021 | 0.055720944 | 0.165423718 | 0.005286919 |
| Deaths | France | Both | 10 to 19 | Pertussis | Number | 2021 | 0.000461306 | 0.001343201 | 7.13E-05 |
| Deaths | France | Both | 10 to 19 | Pertussis | Rate | 2021 | 5.56E-06 | 1.62E-05 | 8.59E-07 |
| Deaths | Eritrea | Both | 10 to 19 | Pertussis | Number | 2021 | 3.089739713 | 9.617683353 | 0.258585731 |
| Deaths | Eritrea | Both | 10 to 19 | Pertussis | Rate | 2021 | 0.212620945 | 0.661842459 | 0.017794619 |
| Deaths | Jamaica | Both | 10 to 19 | Pertussis | Number | 2021 | 6.29E-08 | 3.22E-07 | 0 |
| Deaths | Jamaica | Both | 10 to 19 | Pertussis | Rate | 2021 | 1.40E-08 | 7.15E-08 | 0 |
| Deaths | Madagascar | Both | 10 to 19 | Pertussis | Number | 2021 | 18.10336575 | 79.54740103 | 0.529097498 |
| Deaths | Madagascar | Both | 10 to 19 | Pertussis | Rate | 2021 | 0.263577383 | 1.158176666 | 0.007703437 |
| Deaths | Botswana | Both | 10 to 19 | Pertussis | Number | 2021 | 0.158242422 | 0.76588055 | 0 |
| Deaths | Botswana | Both | 10 to 19 | Pertussis | Rate | 2021 | 0.035332503 | 0.171006461 | 0 |
| Deaths | Burkina Faso | Both | 10 to 19 | Pertussis | Number | 2021 | 4.550042603 | 18.01970516 | 0.266160773 |
| Deaths | Burkina Faso | Both | 10 to 19 | Pertussis | Rate | 2021 | 0.085735404 | 0.339541151 | 0.005015206 |
| Deaths | Ethiopia | Both | 10 to 19 | Pertussis | Number | 2021 | 107.2894315 | 376.6657095 | 8.196864311 |
| Deaths | Ethiopia | Both | 10 to 19 | Pertussis | Rate | 2021 | 0.401725039 | 1.410353701 | 0.030691612 |
| Deaths | El Salvador | Both | 10 to 19 | Pertussis | Number | 2021 | 0.0086492 | 0.031929922 | 0 |
| Deaths | El Salvador | Both | 10 to 19 | Pertussis | Rate | 2021 | 0.000751358 | 0.00277376 | 0 |
| Deaths | Japan | Both | 10 to 19 | Pertussis | Number | 2021 | 0.000163842 | 0.00039972 | 3.18E-05 |
| Deaths | Japan | Both | 10 to 19 | Pertussis | Rate | 2021 | 1.44E-06 | 3.51E-06 | 2.79E-07 |
| Deaths | Slovakia | Both | 10 to 19 | Pertussis | Number | 2021 | 0.010019393 | 0.040354269 | 0.000154739 |
| Deaths | Slovakia | Both | 10 to 19 | Pertussis | Rate | 2021 | 0.00180474 | 0.007268798 | 2.79E-05 |
| Deaths | Greenland | Both | 10 to 19 | Pertussis | Number | 2021 | 0.00143279 | 0.003876389 | 0.000167746 |
| Deaths | Greenland | Both | 10 to 19 | Pertussis | Rate | 2021 | 0.019333956 | 0.05230771 | 0.002263548 |
| Deaths | Puerto Rico | Both | 10 to 19 | Pertussis | Number | 2021 | 1.42E-06 | 5.09E-06 | 1.34E-07 |
| Deaths | Puerto Rico | Both | 10 to 19 | Pertussis | Rate | 2021 | 3.55E-07 | 1.28E-06 | 3.36E-08 |
| Deaths | Kenya | Both | 10 to 19 | Pertussis | Number | 2021 | 17.89747678 | 54.57643189 | 1.048957334 |
| Deaths | Kenya | Both | 10 to 19 | Pertussis | Rate | 2021 | 0.144938129 | 0.441973247 | 0.008494712 |
| Deaths | Canada | Both | 10 to 19 | Pertussis | Number | 2021 | 2.13E-05 | 7.58E-05 | 1.73E-06 |
| Deaths | Canada | Both | 10 to 19 | Pertussis | Rate | 2021 | 5.04E-07 | 1.79E-06 | 4.09E-08 |
| Deaths | Solomon Islands | Both | 10 to 19 | Pertussis | Number | 2021 | 0.670067222 | 2.144748748 | 0.055018495 |
| Deaths | Solomon Islands | Both | 10 to 19 | Pertussis | Rate | 2021 | 0.448168276 | 1.434495403 | 0.036798613 |
| Deaths | Kuwait | Both | 10 to 19 | Pertussis | Number | 2021 | 1.85E-06 | 4.53E-06 | 3.03E-07 |
| Deaths | Kuwait | Both | 10 to 19 | Pertussis | Rate | 2021 | 3.45E-07 | 8.43E-07 | 5.64E-08 |
| Deaths | China | Both | 10 to 19 | Pertussis | Number | 2021 | 43.02095646 | 119.7471967 | 4.218234342 |
| Deaths | China | Both | 10 to 19 | Pertussis | Rate | 2021 | 0.026743497 | 0.074439508 | 0.002622218 |
| Deaths | Afghanistan | Both | 10 to 19 | Pertussis | Number | 2021 | 24.72041923 | 79.0685338 | 2.320307726 |
| Deaths | Afghanistan | Both | 10 to 19 | Pertussis | Rate | 2021 | 0.325513932 | 1.041159905 | 0.030553385 |
| Deaths | Guyana | Both | 10 to 19 | Pertussis | Number | 2021 | 2.90E-06 | 1.36E-05 | 0 |
| Deaths | Guyana | Both | 10 to 19 | Pertussis | Rate | 2021 | 2.15E-06 | 1.01E-05 | 0 |
| Deaths | Malaysia | Both | 10 to 19 | Pertussis | Number | 2021 | 1.417490486 | 5.346197134 | 0.074924909 |
| Deaths | Malaysia | Both | 10 to 19 | Pertussis | Rate | 2021 | 0.027059522 | 0.102057504 | 0.001430297 |
| Deaths | Switzerland | Both | 10 to 19 | Pertussis | Number | 2021 | 1.87E-06 | 6.61E-06 | 2.97E-07 |
| Deaths | Switzerland | Both | 10 to 19 | Pertussis | Rate | 2021 | 2.15E-07 | 7.60E-07 | 3.41E-08 |
| Deaths | Zimbabwe | Both | 10 to 19 | Pertussis | Number | 2021 | 1.92286187 | 8.44314296 | 0.021461488 |
| Deaths | Zimbabwe | Both | 10 to 19 | Pertussis | Rate | 2021 | 0.052687299 | 0.231345996 | 0.000588055 |
| Deaths | Tokelau | Both | 10 to 19 | Pertussis | Number | 2021 | 0.000257222 | 0.000803094 | 2.36E-05 |
| Deaths | Tokelau | Both | 10 to 19 | Pertussis | Rate | 2021 | 0.095897741 | 0.299410611 | 0.008781475 |
| Deaths | India | Both | 10 to 19 | Pertussis | Number | 2021 | 420.4986943 | 1356.904818 | 35.02967333 |
| Deaths | India | Both | 10 to 19 | Pertussis | Rate | 2021 | 0.157805517 | 0.509221715 | 0.013146 |
| Deaths | Mexico | Both | 10 to 19 | Pertussis | Number | 2021 | 0.303389304 | 0.495972183 | 0.156523735 |
| Deaths | Mexico | Both | 10 to 19 | Pertussis | Rate | 2021 | 0.001356942 | 0.00221829 | 0.00070007 |
| Deaths | Brunei Darussalam | Both | 10 to 19 | Pertussis | Number | 2021 | 0.000471841 | 0.002071695 | 0 |
| Deaths | Brunei Darussalam | Both | 10 to 19 | Pertussis | Rate | 2021 | 0.000712388 | 0.00312786 | 0 |
| Deaths | Benin | Both | 10 to 19 | Pertussis | Number | 2021 | 5.701571586 | 19.90591617 | 0.383381421 |
| Deaths | Benin | Both | 10 to 19 | Pertussis | Rate | 2021 | 0.181364735 | 0.633199312 | 0.012195211 |
| Deaths | Tonga | Both | 10 to 19 | Pertussis | Number | 2021 | 0.00220739 | 0.006756591 | 0.000233358 |
| Deaths | Tonga | Both | 10 to 19 | Pertussis | Rate | 2021 | 0.009941457 | 0.030429767 | 0.001050978 |
| Deaths | Mauritania | Both | 10 to 19 | Pertussis | Number | 2021 | 2.549095979 | 8.64549192 | 0.229421577 |
| Deaths | Mauritania | Both | 10 to 19 | Pertussis | Rate | 2021 | 0.244024892 | 0.827632719 | 0.021962522 |
| Deaths | Romania | Both | 10 to 19 | Pertussis | Number | 2021 | 2.43E-06 | 8.26E-06 | 2.45E-08 |
| Deaths | Romania | Both | 10 to 19 | Pertussis | Rate | 2021 | 1.15E-07 | 3.90E-07 | 1.15E-09 |
| Deaths | Serbia | Both | 10 to 19 | Pertussis | Number | 2021 | 0.064591459 | 0.237646389 | 0.001452279 |
| Deaths | Serbia | Both | 10 to 19 | Pertussis | Rate | 2021 | 0.005960867 | 0.02193136 | 0.000134025 |
| Deaths | Saudi Arabia | Both | 10 to 19 | Pertussis | Number | 2021 | 0.687900995 | 2.298712427 | 0.062078924 |
| Deaths | Saudi Arabia | Both | 10 to 19 | Pertussis | Rate | 2021 | 0.013498641 | 0.0451075 | 0.001218171 |
| Deaths | Oman | Both | 10 to 19 | Pertussis | Number | 2021 | 0.041649275 | 0.126306414 | 0.003328218 |
| Deaths | Oman | Both | 10 to 19 | Pertussis | Rate | 2021 | 0.006703241 | 0.020328382 | 0.00053566 |
| Deaths | Grenada | Both | 10 to 19 | Pertussis | Number | 2021 | 2.61E-06 | 6.73E-06 | 3.05E-07 |
| Deaths | Grenada | Both | 10 to 19 | Pertussis | Rate | 2021 | 1.63E-05 | 4.19E-05 | 1.90E-06 |
| Deaths | Nicaragua | Both | 10 to 19 | Pertussis | Number | 2021 | 0.005906455 | 0.020165505 | 0.000297749 |
| Deaths | Nicaragua | Both | 10 to 19 | Pertussis | Rate | 2021 | 0.000464258 | 0.001585046 | 2.34E-05 |
| Deaths | Timor-Leste | Both | 10 to 19 | Pertussis | Number | 2021 | 0.000353556 | 0.000195393 | 0 |
| Deaths | Timor-Leste | Both | 10 to 19 | Pertussis | Rate | 2021 | 0.000106847 | 5.90E-05 | 0 |
| Deaths | Mauritius | Both | 10 to 19 | Pertussis | Number | 2021 | 1.97E-07 | 7.24E-07 | 0 |
| Deaths | Mauritius | Both | 10 to 19 | Pertussis | Rate | 2021 | 1.19E-07 | 4.37E-07 | 0 |
| Deaths | United States Virgin Islands | Both | 10 to 19 | Pertussis | Number | 2021 | 0.002316167 | 0.007293223 | 0 |
| Deaths | United States Virgin Islands | Both | 10 to 19 | Pertussis | Rate | 2021 | 0.023597006 | 0.074303046 | 0 |
| Deaths | Niger | Both | 10 to 19 | Pertussis | Number | 2021 | 22.78257474 | 84.72502762 | 1.705925102 |
| Deaths | Niger | Both | 10 to 19 | Pertussis | Rate | 2021 | 0.363741423 | 1.352700581 | 0.027236413 |
| Deaths | Chile | Both | 10 to 19 | Pertussis | Number | 2021 | 0.001462702 | 0.004584312 | 0.000234093 |
| Deaths | Chile | Both | 10 to 19 | Pertussis | Rate | 2021 | 5.79E-05 | 0.000181461 | 9.27E-06 |
| Deaths | Israel | Both | 10 to 19 | Pertussis | Number | 2021 | 2.36E-05 | 0.000103705 | 0 |
| Deaths | Israel | Both | 10 to 19 | Pertussis | Rate | 2021 | 1.51E-06 | 6.64E-06 | 0 |
| Deaths | Togo | Both | 10 to 19 | Pertussis | Number | 2021 | 6.336044964 | 20.30379418 | 0.478450882 |
| Deaths | Togo | Both | 10 to 19 | Pertussis | Rate | 2021 | 0.337870482 | 1.082702657 | 0.02551346 |
| Deaths | Senegal | Both | 10 to 19 | Pertussis | Number | 2021 | 5.049137783 | 18.54258073 | 0.517292911 |
| Deaths | Senegal | Both | 10 to 19 | Pertussis | Rate | 2021 | 0.13521509 | 0.496567302 | 0.01385302 |
| Deaths | Tuvalu | Both | 10 to 19 | Pertussis | Number | 2021 | 0.005426848 | 0.016076218 | 0.000616742 |
| Deaths | Tuvalu | Both | 10 to 19 | Pertussis | Rate | 2021 | 0.227267581 | 0.673245885 | 0.025828133 |
| Deaths | Qatar | Both | 10 to 19 | Pertussis | Number | 2021 | 0.005809039 | 0.017035606 | 0.000728005 |
| Deaths | Qatar | Both | 10 to 19 | Pertussis | Rate | 2021 | 0.002366847 | 0.006941023 | 0.00029662 |
| Deaths | Poland | Both | 10 to 19 | Pertussis | Number | 2021 | 1.42E-05 | 4.08E-05 | 1.78E-06 |
| Deaths | Poland | Both | 10 to 19 | Pertussis | Rate | 2021 | 3.68E-07 | 1.05E-06 | 4.58E-08 |
| Deaths | Panama | Both | 10 to 19 | Pertussis | Number | 2021 | 0.000543569 | 0.001841743 | 6.67E-05 |
| Deaths | Panama | Both | 10 to 19 | Pertussis | Rate | 2021 | 7.40E-05 | 0.000250664 | 9.08E-06 |
| Deaths | Viet Nam | Both | 10 to 19 | Pertussis | Number | 2021 | 1.823731845 | 6.405545421 | 0.126674492 |
| Deaths | Viet Nam | Both | 10 to 19 | Pertussis | Rate | 2021 | 0.012363466 | 0.043424554 | 0.000858753 |
| Deaths | Guatemala | Both | 10 to 19 | Pertussis | Number | 2021 | 0.096760012 | 0.301118321 | 0.004643728 |
| Deaths | Guatemala | Both | 10 to 19 | Pertussis | Rate | 2021 | 0.002909185 | 0.00905342 | 0.000139618 |
| Deaths | Malawi | Both | 10 to 19 | Pertussis | Number | 2021 | 6.906678318 | 23.44970984 | 0.60873809 |
| Deaths | Malawi | Both | 10 to 19 | Pertussis | Rate | 2021 | 0.133474806 | 0.453176669 | 0.01176415 |
| Deaths | Nepal | Both | 10 to 19 | Pertussis | Number | 2021 | 5.551171066 | 20.57288416 | 0.284869197 |
| Deaths | Nepal | Both | 10 to 19 | Pertussis | Rate | 2021 | 0.087900655 | 0.325763695 | 0.004510794 |
| Deaths | Eswatini | Both | 10 to 19 | Pertussis | Number | 2021 | 0.169242201 | 0.664913462 | 0.004527153 |
| Deaths | Eswatini | Both | 10 to 19 | Pertussis | Rate | 2021 | 0.066123345 | 0.259783327 | 0.00176877 |
| Deaths | Mozambique | Both | 10 to 19 | Pertussis | Number | 2021 | 10.63323703 | 38.94870542 | 0.772549569 |
| Deaths | Mozambique | Both | 10 to 19 | Pertussis | Rate | 2021 | 0.138368486 | 0.506832808 | 0.010053055 |
| Deaths | Suriname | Both | 10 to 19 | Pertussis | Number | 2021 | 0.00975617 | 0.043256025 | 0 |
| Deaths | Suriname | Both | 10 to 19 | Pertussis | Rate | 2021 | 0.010098861 | 0.044775417 | 0 |
| Deaths | Armenia | Both | 10 to 19 | Pertussis | Number | 2021 | 4.54E-05 | 0.000147028 | 6.70E-06 |
| Deaths | Armenia | Both | 10 to 19 | Pertussis | Rate | 2021 | 1.23E-05 | 3.98E-05 | 1.81E-06 |
| Deaths | Tajikistan | Both | 10 to 19 | Pertussis | Number | 2021 | 0.544217411 | 1.679070921 | 0.03690729 |
| Deaths | Tajikistan | Both | 10 to 19 | Pertussis | Rate | 2021 | 0.028577624 | 0.088170383 | 0.001938054 |
| Deaths | Trinidad and Tobago | Both | 10 to 19 | Pertussis | Number | 2021 | 1.62E-06 | 7.79E-06 | 3.09E-09 |
| Deaths | Trinidad and Tobago | Both | 10 to 19 | Pertussis | Rate | 2021 | 8.57E-07 | 4.13E-06 | 1.64E-09 |
| Deaths | Pakistan | Both | 10 to 19 | Pertussis | Number | 2021 | 63.8141821 | 268.6768335 | 3.256216688 |
| Deaths | Pakistan | Both | 10 to 19 | Pertussis | Rate | 2021 | 0.126227997 | 0.531457702 | 0.006440977 |
| Deaths | Palestine | Both | 10 to 19 | Pertussis | Number | 2021 | 0.061206501 | 0.200712324 | 0.005612097 |
| Deaths | Palestine | Both | 10 to 19 | Pertussis | Rate | 2021 | 0.005306383 | 0.017401035 | 0.000486549 |
| Deaths | Ireland | Both | 10 to 19 | Pertussis | Number | 2021 | 3.64E-07 | 1.50E-06 | 2.84E-08 |
| Deaths | Ireland | Both | 10 to 19 | Pertussis | Rate | 2021 | 5.32E-08 | 2.19E-07 | 4.15E-09 |
| Deaths | Cabo Verde | Both | 10 to 19 | Pertussis | Number | 2021 | 0.006840452 | 0.022087167 | 0.000537346 |
| Deaths | Cabo Verde | Both | 10 to 19 | Pertussis | Rate | 2021 | 0.00682061 | 0.022023099 | 0.000535788 |
| Deaths | Italy | Both | 10 to 19 | Pertussis | Number | 2021 | 6.93E-05 | 0.000139131 | 1.93E-05 |
| Deaths | Italy | Both | 10 to 19 | Pertussis | Rate | 2021 | 1.21E-06 | 2.43E-06 | 3.38E-07 |
| Deaths | Uruguay | Both | 10 to 19 | Pertussis | Number | 2021 | 5.06E-06 | 2.01E-05 | 1.32E-07 |
| Deaths | Uruguay | Both | 10 to 19 | Pertussis | Rate | 2021 | 1.07E-06 | 4.27E-06 | 2.80E-08 |
| Deaths | Rwanda | Both | 10 to 19 | Pertussis | Number | 2021 | 2.464463211 | 8.641750862 | 0.224628696 |
| Deaths | Rwanda | Both | 10 to 19 | Pertussis | Rate | 2021 | 0.081195597 | 0.284716005 | 0.007400744 |
| Deaths | Angola | Both | 10 to 19 | Pertussis | Number | 2021 | 65.33677529 | 215.4062486 | 5.689114561 |
| Deaths | Angola | Both | 10 to 19 | Pertussis | Rate | 2021 | 0.83648738 | 2.757782394 | 0.072836049 |
| Deaths | Saint Lucia | Both | 10 to 19 | Pertussis | Number | 2021 | 4.88E-09 | 3.87E-08 | 0 |
| Deaths | Saint Lucia | Both | 10 to 19 | Pertussis | Rate | 2021 | 2.11E-08 | 1.67E-07 | 0 |
| Deaths | Australia | Both | 10 to 19 | Pertussis | Number | 2021 | 3.51E-06 | 2.94E-05 | 0 |
| Deaths | Australia | Both | 10 to 19 | Pertussis | Rate | 2021 | 1.13E-07 | 9.43E-07 | 0 |
| Deaths | Singapore | Both | 10 to 19 | Pertussis | Number | 2021 | 3.23E-08 | 1.56E-07 | 0 |
| Deaths | Singapore | Both | 10 to 19 | Pertussis | Rate | 2021 | 6.96E-09 | 3.36E-08 | 0 |
| Deaths | Sierra Leone | Both | 10 to 19 | Pertussis | Number | 2021 | 2.960384723 | 10.94438964 | 0.228040017 |
| Deaths | Sierra Leone | Both | 10 to 19 | Pertussis | Rate | 2021 | 0.146014113 | 0.539806646 | 0.011247545 |
| Deaths | Brazil | Both | 10 to 19 | Pertussis | Number | 2021 | 0.01935263 | 0.046907815 | 0.005176315 |
| Deaths | Brazil | Both | 10 to 19 | Pertussis | Rate | 2021 | 6.18E-05 | 0.000149732 | 1.65E-05 |
| Deaths | United States of America | Both | 10 to 19 | Pertussis | Number | 2021 | 0.02986167 | 0.057535856 | 0.010651776 |
| Deaths | United States of America | Both | 10 to 19 | Pertussis | Rate | 2021 | 6.95E-05 | 0.00013395 | 2.48E-05 |
| Deaths | Thailand | Both | 10 to 19 | Pertussis | Number | 2021 | 0.237843529 | 0.88879341 | 0.00403211 |
| Deaths | Thailand | Both | 10 to 19 | Pertussis | Rate | 2021 | 0.003145242 | 0.011753402 | 5.33E-05 |
| Deaths | South Sudan | Both | 10 to 19 | Pertussis | Number | 2021 | 14.94511777 | 53.52620781 | 1.211099693 |
| Deaths | South Sudan | Both | 10 to 19 | Pertussis | Rate | 2021 | 0.585122869 | 2.09562807 | 0.047416296 |
| Deaths | Saint Vincent and the Grenadines | Both | 10 to 19 | Pertussis | Number | 2021 | 5.49E-08 | 2.01E-07 | 4.95E-09 |
| Deaths | Saint Vincent and the Grenadines | Both | 10 to 19 | Pertussis | Rate | 2021 | 3.09E-07 | 1.13E-06 | 2.78E-08 |
| Deaths | Slovenia | Both | 10 to 19 | Pertussis | Number | 2021 | 1.07E-06 | 4.19E-06 | 8.37E-09 |
| Deaths | Slovenia | Both | 10 to 19 | Pertussis | Rate | 2021 | 5.34E-07 | 2.10E-06 | 4.18E-09 |
| Deaths | Paraguay | Both | 10 to 19 | Pertussis | Number | 2021 | 0.37452956 | 1.269147937 | 0.027964691 |
| Deaths | Paraguay | Both | 10 to 19 | Pertussis | Rate | 2021 | 0.027873236 | 0.094452519 | 0.002081188 |
| Deaths | Central African Republic | Both | 10 to 19 | Pertussis | Number | 2021 | 16.84246595 | 56.36484463 | 1.18246655 |
| Deaths | Central African Republic | Both | 10 to 19 | Pertussis | Rate | 2021 | 1.293570516 | 4.329051421 | 0.090818285 |
| Deaths | Mongolia | Both | 10 to 19 | Pertussis | Number | 2021 | 0.078324823 | 0.33374538 | 0 |
| Deaths | Mongolia | Both | 10 to 19 | Pertussis | Rate | 2021 | 0.014755819 | 0.062875169 | 0 |
| Deaths | Kyrgyzstan | Both | 10 to 19 | Pertussis | Number | 2021 | 1.19E-05 | 3.73E-05 | 1.75E-06 |
| Deaths | Kyrgyzstan | Both | 10 to 19 | Pertussis | Rate | 2021 | 9.69E-07 | 3.02E-06 | 1.42E-07 |
| Deaths | Cameroon | Both | 10 to 19 | Pertussis | Number | 2021 | 19.18114137 | 73.37065749 | 1.192142098 |
| Deaths | Cameroon | Both | 10 to 19 | Pertussis | Rate | 2021 | 0.256119151 | 0.979693031 | 0.015918261 |
| Deaths | Venezuela (Bolivarian Republic of) | Both | 10 to 19 | Pertussis | Number | 2021 | 0.00588155 | 0.017277903 | 0.001301496 |
| Deaths | Venezuela (Bolivarian Republic of) | Both | 10 to 19 | Pertussis | Rate | 2021 | 0.000135063 | 0.000396768 | 2.99E-05 |
| Deaths | Niue | Both | 10 to 19 | Pertussis | Number | 2021 | 0.000161688 | 0.000490173 | 1.59E-05 |
| Deaths | Niue | Both | 10 to 19 | Pertussis | Rate | 2021 | 0.057737557 | 0.175037483 | 0.005684847 |
| Deaths | American Samoa | Both | 10 to 19 | Pertussis | Number | 2021 | 0.010995882 | 0.032522368 | 0.001155543 |
| Deaths | American Samoa | Both | 10 to 19 | Pertussis | Rate | 2021 | 0.105088309 | 0.310818239 | 0.011043594 |
| Deaths | Coted'Ivoire | Both | 10 to 19 | Pertussis | Number | 2021 | 6.107498647 | 23.10073536 | 0.315633355 |
| Deaths | Coted'Ivoire | Both | 10 to 19 | Pertussis | Rate | 2021 | 0.099403869 | 0.37598084 | 0.005137157 |
| Deaths | Gambia | Both | 10 to 19 | Pertussis | Number | 2021 | 0.736849411 | 2.39315796 | 0.041453054 |
| Deaths | Gambia | Both | 10 to 19 | Pertussis | Rate | 2021 | 0.127120889 | 0.412866406 | 0.00715146 |
| Deaths | Liberia | Both | 10 to 19 | Pertussis | Number | 2021 | 1.56696483 | 5.693298892 | 0.095859074 |
| Deaths | Liberia | Both | 10 to 19 | Pertussis | Rate | 2021 | 0.120785176 | 0.438852294 | 0.007389033 |
| Deaths | Sao Tome and Principe | Both | 10 to 19 | Pertussis | Number | 2021 | 0.046388462 | 0.152985332 | 0.003570748 |
| Deaths | Sao Tome and Principe | Both | 10 to 19 | Pertussis | Rate | 2021 | 0.091694691 | 0.302401552 | 0.007058191 |
| Deaths | Guam | Both | 10 to 19 | Pertussis | Number | 2021 | 0.001521856 | 0.004677034 | 0.000123741 |
| Deaths | Guam | Both | 10 to 19 | Pertussis | Rate | 2021 | 0.00631204 | 0.019398434 | 0.000513225 |
| Deaths | San Marino | Both | 10 to 19 | Pertussis | Number | 2021 | 7.43E-05 | 0.000336003 | 3.02E-06 |
| Deaths | San Marino | Both | 10 to 19 | Pertussis | Rate | 2021 | 0.002081185 | 0.009417427 | 8.47E-05 |

Sup_Table 7 DALYs of Adolescent Pertussis in 204 Countries and Territories in 2021

| measure | location | sex | age | cause | metric | year | val | upper | lower |
| --- | --- | --- | --- | --- | --- | --- | --- | --- | --- |
| DALYs | Norway | Both | 10 to 19 | Pertussis | Number | 2021 | 0.021766269 | 0.070804017 | 0.004282137 |
| DALYs | Norway | Both | 10 to 19 | Pertussis | Rate | 2021 | 0.003365865 | 0.010948902 | 0.000662176 |
| DALYs | Slovakia | Both | 10 to 19 | Pertussis | Number | 2021 | 0.777751264 | 3.104463845 | 0.020817298 |
| DALYs | Slovakia | Both | 10 to 19 | Pertussis | Rate | 2021 | 0.140092168 | 0.55919044 | 0.003749708 |
| DALYs | Denmark | Both | 10 to 19 | Pertussis | Number | 2021 | 0.01303033 | 0.039166698 | 0.001765416 |
| DALYs | Denmark | Both | 10 to 19 | Pertussis | Rate | 2021 | 0.001924534 | 0.005784783 | 0.000260746 |
| DALYs | Belize | Both | 10 to 19 | Pertussis | Number | 2021 | 0.001385923 | 0.00627879 | 0.000346831 |
| DALYs | Belize | Both | 10 to 19 | Pertussis | Rate | 2021 | 0.001541254 | 0.006982503 | 0.000385703 |
| DALYs | United Arab Emirates | Both | 10 to 19 | Pertussis | Number | 2021 | 1.269739668 | 4.729293927 | 0.139179769 |
| DALYs | United Arab Emirates | Both | 10 to 19 | Pertussis | Rate | 2021 | 0.164652223 | 0.613266466 | 0.018047997 |
| DALYs | Democratic People's Republic of Korea | Both | 10 to 19 | Pertussis | Number | 2021 | 5.122856412 | 19.03016905 | 0.381140003 |
| DALYs | Democratic People's Republic of Korea | Both | 10 to 19 | Pertussis | Rate | 2021 | 0.145820906 | 0.541689299 | 0.010849061 |
| DALYs | Haiti | Both | 10 to 19 | Pertussis | Number | 2021 | 3.033448398 | 18.36499029 | 0.020418717 |
| DALYs | Haiti | Both | 10 to 19 | Pertussis | Rate | 2021 | 0.116975004 | 0.708185714 | 0.000787381 |
| DALYs | Albania | Both | 10 to 19 | Pertussis | Number | 2021 | 0.066588832 | 0.222002757 | 0.002194282 |
| DALYs | Albania | Both | 10 to 19 | Pertussis | Rate | 2021 | 0.020443828 | 0.068158371 | 0.00067368 |
| DALYs | Timor-Leste | Both | 10 to 19 | Pertussis | Number | 2021 | 0.109167107 | 0.183086639 | 0.034620514 |
| DALYs | Timor-Leste | Both | 10 to 19 | Pertussis | Rate | 2021 | 0.03299088 | 0.055329755 | 0.010462503 |
| DALYs | Liberia | Both | 10 to 19 | Pertussis | Number | 2021 | 121.9402435 | 439.6727763 | 8.707824762 |
| DALYs | Liberia | Both | 10 to 19 | Pertussis | Rate | 2021 | 9.399428442 | 33.89096725 | 0.671218733 |
| DALYs | Zimbabwe | Both | 10 to 19 | Pertussis | Number | 2021 | 149.5971979 | 651.0642283 | 2.366969461 |
| DALYs | Zimbabwe | Both | 10 to 19 | Pertussis | Rate | 2021 | 4.099031957 | 17.839459 | 0.064856051 |
| DALYs | Jordan | Both | 10 to 19 | Pertussis | Number | 2021 | 60.64137005 | 196.3699823 | 6.874637902 |
| DALYs | Jordan | Both | 10 to 19 | Pertussis | Rate | 2021 | 2.322492324 | 7.520736688 | 0.263290452 |
| DALYs | Panama | Both | 10 to 19 | Pertussis | Number | 2021 | 0.934501182 | 2.443260019 | 0.197055358 |
| DALYs | Panama | Both | 10 to 19 | Pertussis | Rate | 2021 | 0.127187164 | 0.332531749 | 0.026819562 |
| DALYs | Oman | Both | 10 to 19 | Pertussis | Number | 2021 | 3.260777567 | 9.75268146 | 0.339681146 |
| DALYs | Oman | Both | 10 to 19 | Pertussis | Rate | 2021 | 0.524805752 | 1.569645038 | 0.054669972 |
| DALYs | United States of America | Both | 10 to 19 | Pertussis | Number | 2021 | 12.82629941 | 26.18315314 | 5.421447858 |
| DALYs | United States of America | Both | 10 to 19 | Pertussis | Rate | 2021 | 0.029861028 | 0.060957244 | 0.012621724 |
| DALYs | Finland | Both | 10 to 19 | Pertussis | Number | 2021 | 0.066071165 | 0.169986333 | 0.016200848 |
| DALYs | Finland | Both | 10 to 19 | Pertussis | Rate | 2021 | 0.010756928 | 0.027675171 | 0.002637631 |
| DALYs | Kenya | Both | 10 to 19 | Pertussis | Number | 2021 | 1388.250618 | 4193.49737 | 99.38719311 |
| DALYs | Kenya | Both | 10 to 19 | Pertussis | Rate | 2021 | 11.24239185 | 33.95996372 | 0.804861712 |
| DALYs | Japan | Both | 10 to 19 | Pertussis | Number | 2021 | 0.024303867 | 0.07998262 | 0.004799254 |
| DALYs | Japan | Both | 10 to 19 | Pertussis | Rate | 2021 | 0.000213534 | 0.000702728 | 4.22E-05 |
| DALYs | Bosnia and Herzegovina | Both | 10 to 19 | Pertussis | Number | 2021 | 4.278014724 | 15.18667766 | 0.282182976 |
| DALYs | Bosnia and Herzegovina | Both | 10 to 19 | Pertussis | Rate | 2021 | 1.230601472 | 4.368556231 | 0.081171947 |
| DALYs | Taiwan (Province of China) | Both | 10 to 19 | Pertussis | Number | 2021 | 0.291702855 | 0.570641411 | 0.13691901 |
| DALYs | Taiwan (Province of China) | Both | 10 to 19 | Pertussis | Rate | 2021 | 0.014024458 | 0.027435236 | 0.006582777 |
| DALYs | Slovenia | Both | 10 to 19 | Pertussis | Number | 2021 | 0.004122421 | 0.0129339 | 0.000909218 |
| DALYs | Slovenia | Both | 10 to 19 | Pertussis | Rate | 2021 | 0.002060573 | 0.00646495 | 0.000454468 |
| DALYs | Bhutan | Both | 10 to 19 | Pertussis | Number | 2021 | 0.589040314 | 2.071965601 | 0.062295531 |
| DALYs | Bhutan | Both | 10 to 19 | Pertussis | Rate | 2021 | 0.444367687 | 1.563075634 | 0.046995291 |
| DALYs | United States Virgin Islands | Both | 10 to 19 | Pertussis | Number | 2021 | 0.179505208 | 0.564580022 | 9.18E-05 |
| DALYs | United States Virgin Islands | Both | 10 to 19 | Pertussis | Rate | 2021 | 1.828791331 | 5.751916957 | 0.000935557 |
| DALYs | Angola | Both | 10 to 19 | Pertussis | Number | 2021 | 5068.478409 | 16630.93546 | 465.0298114 |
| DALYs | Angola | Both | 10 to 19 | Pertussis | Rate | 2021 | 64.89022772 | 212.9209403 | 5.953638928 |
| DALYs | Republic of Korea | Both | 10 to 19 | Pertussis | Number | 2021 | 1.665890587 | 7.472085514 | 0.00104823 |
| DALYs | Republic of Korea | Both | 10 to 19 | Pertussis | Rate | 2021 | 0.035997114 | 0.161459292 | 2.27E-05 |
| DALYs | Coted'Ivoire | Both | 10 to 19 | Pertussis | Number | 2021 | 476.4031466 | 1784.84116 | 28.89115825 |
| DALYs | Coted'Ivoire | Both | 10 to 19 | Pertussis | Rate | 2021 | 7.753798846 | 29.04955482 | 0.470224076 |
| DALYs | Sierra Leone | Both | 10 to 19 | Pertussis | Number | 2021 | 230.2560044 | 844.8924869 | 19.76918474 |
| DALYs | Sierra Leone | Both | 10 to 19 | Pertussis | Rate | 2021 | 11.35684361 | 41.67236317 | 0.975069206 |
| DALYs | Viet Nam | Both | 10 to 19 | Pertussis | Number | 2021 | 139.7233068 | 484.1320934 | 10.28183696 |
| DALYs | Viet Nam | Both | 10 to 19 | Pertussis | Rate | 2021 | 0.947214003 | 3.28203439 | 0.069702759 |
| DALYs | Costa Rica | Both | 10 to 19 | Pertussis | Number | 2021 | 0.011225081 | 0.026505581 | 0.003364223 |
| DALYs | Costa Rica | Both | 10 to 19 | Pertussis | Rate | 2021 | 0.001579333 | 0.003729251 | 0.000473335 |
| DALYs | France | Both | 10 to 19 | Pertussis | Number | 2021 | 0.632714535 | 1.425112121 | 0.198820373 |
| DALYs | France | Both | 10 to 19 | Pertussis | Rate | 2021 | 0.007622793 | 0.017169409 | 0.00239534 |
| DALYs | Portugal | Both | 10 to 19 | Pertussis | Number | 2021 | 0.026658892 | 0.092932104 | 0.002660043 |
| DALYs | Portugal | Both | 10 to 19 | Pertussis | Rate | 2021 | 0.002515028 | 0.008767314 | 0.000250951 |
| DALYs | Jamaica | Both | 10 to 19 | Pertussis | Number | 2021 | 0.002719636 | 0.012267819 | 0.000646816 |
| DALYs | Jamaica | Both | 10 to 19 | Pertussis | Rate | 2021 | 0.000603887 | 0.002724031 | 0.000143623 |
| DALYs | Bahrain | Both | 10 to 19 | Pertussis | Number | 2021 | 2.79381694 | 8.340868023 | 0.330438699 |
| DALYs | Bahrain | Both | 10 to 19 | Pertussis | Rate | 2021 | 1.349852979 | 4.029951063 | 0.159653861 |
| DALYs | Botswana | Both | 10 to 19 | Pertussis | Number | 2021 | 12.35242599 | 58.97826843 | 0.145387588 |
| DALYs | Botswana | Both | 10 to 19 | Pertussis | Rate | 2021 | 2.758060183 | 13.16871795 | 0.032462264 |
| DALYs | American Samoa | Both | 10 to 19 | Pertussis | Number | 2021 | 0.841586844 | 2.4898572 | 0.088871181 |
| DALYs | American Samoa | Both | 10 to 19 | Pertussis | Rate | 2021 | 8.043096506 | 23.79571625 | 0.849347267 |
| DALYs | Antigua and Barbuda | Both | 10 to 19 | Pertussis | Number | 2021 | 0.000124266 | 0.000448305 | 2.94E-05 |
| DALYs | Antigua and Barbuda | Both | 10 to 19 | Pertussis | Rate | 2021 | 0.000986853 | 0.003560189 | 0.000233561 |
| DALYs | Puerto Rico | Both | 10 to 19 | Pertussis | Number | 2021 | 0.004392554 | 0.019679852 | 0.001107791 |
| DALYs | Puerto Rico | Both | 10 to 19 | Pertussis | Rate | 2021 | 0.001103416 | 0.004943609 | 0.000278279 |
| DALYs | Myanmar | Both | 10 to 19 | Pertussis | Number | 2021 | 94.81304073 | 384.1838724 | 4.220955929 |
| DALYs | Myanmar | Both | 10 to 19 | Pertussis | Rate | 2021 | 0.916256061 | 3.712683391 | 0.040790554 |
| DALYs | Mali | Both | 10 to 19 | Pertussis | Number | 2021 | 1264.545154 | 4604.038057 | 99.03302712 |
| DALYs | Mali | Both | 10 to 19 | Pertussis | Rate | 2021 | 21.66519883 | 78.8800618 | 1.696713016 |
| DALYs | Ghana | Both | 10 to 19 | Pertussis | Number | 2021 | 519.0196259 | 1654.411694 | 37.46837208 |
| DALYs | Ghana | Both | 10 to 19 | Pertussis | Rate | 2021 | 7.097525297 | 22.62386288 | 0.512375073 |
| DALYs | Sweden | Both | 10 to 19 | Pertussis | Number | 2021 | 0.212745144 | 0.539071801 | 0.051199186 |
| DALYs | Sweden | Both | 10 to 19 | Pertussis | Rate | 2021 | 0.017453359 | 0.044224809 | 0.00420032 |
| DALYs | Singapore | Both | 10 to 19 | Pertussis | Number | 2021 | 0.004420095 | 0.016168108 | 0.000408032 |
| DALYs | Singapore | Both | 10 to 19 | Pertussis | Rate | 2021 | 0.000953793 | 0.003488845 | 8.80E-05 |
| DALYs | Saint Vincent and the Grenadines | Both | 10 to 19 | Pertussis | Number | 2021 | 7.89E-05 | 0.000339737 | 2.10E-05 |
| DALYs | Saint Vincent and the Grenadines | Both | 10 to 19 | Pertussis | Rate | 2021 | 0.000444154 | 0.001912436 | 0.000118385 |
| DALYs | Belarus | Both | 10 to 19 | Pertussis | Number | 2021 | 0.278562059 | 0.665613991 | 0.060563232 |
| DALYs | Belarus | Both | 10 to 19 | Pertussis | Rate | 2021 | 0.028482185 | 0.068057153 | 0.00619242 |
| DALYs | Tajikistan | Both | 10 to 19 | Pertussis | Number | 2021 | 42.29677801 | 129.5570651 | 3.302230809 |
| DALYs | Tajikistan | Both | 10 to 19 | Pertussis | Rate | 2021 | 2.22106348 | 6.803224252 | 0.173404798 |
| DALYs | Germany | Both | 10 to 19 | Pertussis | Number | 2021 | 0.09349452 | 0.234937255 | 0.028416845 |
| DALYs | Germany | Both | 10 to 19 | Pertussis | Rate | 2021 | 0.001188848 | 0.002987391 | 0.00036134 |
| DALYs | Cabo Verde | Both | 10 to 19 | Pertussis | Number | 2021 | 0.533919099 | 1.697222646 | 0.050685445 |
| DALYs | Cabo Verde | Both | 10 to 19 | Pertussis | Rate | 2021 | 0.532370368 | 1.692299538 | 0.050538422 |
| DALYs | Niue | Both | 10 to 19 | Pertussis | Number | 2021 | 0.012412397 | 0.037600191 | 0.001229746 |
| DALYs | Niue | Both | 10 to 19 | Pertussis | Rate | 2021 | 4.432385506 | 13.42678162 | 0.439134362 |
| DALYs | Seychelles | Both | 10 to 19 | Pertussis | Number | 2021 | 0.025294212 | 0.110836691 | 0.000527502 |
| DALYs | Seychelles | Both | 10 to 19 | Pertussis | Rate | 2021 | 0.170497421 | 0.747102554 | 0.003555665 |
| DALYs | Samoa | Both | 10 to 19 | Pertussis | Number | 2021 | 22.92025995 | 69.13048825 | 2.204964861 |
| DALYs | Samoa | Both | 10 to 19 | Pertussis | Rate | 2021 | 50.98966529 | 153.7914694 | 4.905285563 |
| DALYs | Cook Islands | Both | 10 to 19 | Pertussis | Number | 2021 | 0.061279124 | 0.190888338 | 0.00564162 |
| DALYs | Cook Islands | Both | 10 to 19 | Pertussis | Rate | 2021 | 2.182016441 | 6.797118865 | 0.200885839 |
| DALYs | Guyana | Both | 10 to 19 | Pertussis | Number | 2021 | 0.001586679 | 0.00654323 | 0.000374236 |
| DALYs | Guyana | Both | 10 to 19 | Pertussis | Rate | 2021 | 0.001176693 | 0.004852508 | 0.000277536 |
| DALYs | San Marino | Both | 10 to 19 | Pertussis | Number | 2021 | 0.006045074 | 0.025992924 | 0.000589509 |
| DALYs | San Marino | Both | 10 to 19 | Pertussis | Rate | 2021 | 0.169430376 | 0.728525575 | 0.016522658 |
| DALYs | Spain | Both | 10 to 19 | Pertussis | Number | 2021 | 0.327418182 | 0.930814211 | 0.069644564 |
| DALYs | Spain | Both | 10 to 19 | Pertussis | Rate | 2021 | 0.00684927 | 0.019471729 | 0.001456897 |
| DALYs | Malaysia | Both | 10 to 19 | Pertussis | Number | 2021 | 112.0691653 | 415.1440618 | 8.651531313 |
| DALYs | Malaysia | Both | 10 to 19 | Pertussis | Rate | 2021 | 2.139371029 | 7.924991464 | 0.165155468 |
| DALYs | Namibia | Both | 10 to 19 | Pertussis | Number | 2021 | 17.49262605 | 63.01771754 | 1.12054089 |
| DALYs | Namibia | Both | 10 to 19 | Pertussis | Rate | 2021 | 3.385322219 | 12.19572629 | 0.216856632 |
| DALYs | Sao Tome and Principe | Both | 10 to 19 | Pertussis | Number | 2021 | 3.598294807 | 11.78629087 | 0.309780572 |
| DALYs | Sao Tome and Principe | Both | 10 to 19 | Pertussis | Rate | 2021 | 7.112642225 | 23.29761028 | 0.612334035 |
| DALYs | Micronesia (Federated States of) | Both | 10 to 19 | Pertussis | Number | 2021 | 8.991202257 | 27.08370405 | 0.88043847 |
| DALYs | Micronesia (Federated States of) | Both | 10 to 19 | Pertussis | Rate | 2021 | 41.34282743 | 124.5347252 | 4.048381369 |
| DALYs | Bangladesh | Both | 10 to 19 | Pertussis | Number | 2021 | 1893.812651 | 5812.762691 | 231.2549244 |
| DALYs | Bangladesh | Both | 10 to 19 | Pertussis | Rate | 2021 | 6.088674431 | 18.68823695 | 0.743492734 |
| DALYs | Kyrgyzstan | Both | 10 to 19 | Pertussis | Number | 2021 | 0.795371168 | 1.964683773 | 0.203740261 |
| DALYs | Kyrgyzstan | Both | 10 to 19 | Pertussis | Rate | 2021 | 0.064497261 | 0.159317721 | 0.016521455 |
| DALYs | Lao People's Democratic Republic | Both | 10 to 19 | Pertussis | Number | 2021 | 27.70711845 | 123.8706939 | 1.291019541 |
| DALYs | Lao People's Democratic Republic | Both | 10 to 19 | Pertussis | Rate | 2021 | 1.974365963 | 8.826832072 | 0.091996035 |
| DALYs | North Macedonia | Both | 10 to 19 | Pertussis | Number | 2021 | 0.662511694 | 3.264670415 | 0.00550496 |
| DALYs | North Macedonia | Both | 10 to 19 | Pertussis | Rate | 2021 | 0.284519825 | 1.402033298 | 0.00236414 |
| DALYs | Niger | Both | 10 to 19 | Pertussis | Number | 2021 | 1769.715975 | 6538.220732 | 148.7217576 |
| DALYs | Niger | Both | 10 to 19 | Pertussis | Rate | 2021 | 28.25488401 | 104.3877498 | 2.374457862 |
| DALYs | Republic of Moldova | Both | 10 to 19 | Pertussis | Number | 2021 | 0.079457399 | 0.199148344 | 0.023746023 |
| DALYs | Republic of Moldova | Both | 10 to 19 | Pertussis | Rate | 2021 | 0.021998884 | 0.055136984 | 0.006574416 |
| DALYs | United Kingdom | Both | 10 to 19 | Pertussis | Number | 2021 | 0.622763143 | 1.184661422 | 0.298320743 |
| DALYs | United Kingdom | Both | 10 to 19 | Pertussis | Rate | 2021 | 0.00779972 | 0.014837145 | 0.003736281 |
| DALYs | Hungary | Both | 10 to 19 | Pertussis | Number | 2021 | 0.00424745 | 0.010034834 | 0.001524595 |
| DALYs | Hungary | Both | 10 to 19 | Pertussis | Rate | 2021 | 0.000441212 | 0.001042388 | 0.00015837 |
| DALYs | Sri Lanka | Both | 10 to 19 | Pertussis | Number | 2021 | 3.140356918 | 14.6976906 | 0.097410166 |
| DALYs | Sri Lanka | Both | 10 to 19 | Pertussis | Rate | 2021 | 0.086750807 | 0.406016437 | 0.002690908 |
| DALYs | Iceland | Both | 10 to 19 | Pertussis | Number | 2021 | 0.08862406 | 0.167585505 | 0.037816243 |
| DALYs | Iceland | Both | 10 to 19 | Pertussis | Rate | 2021 | 0.19624726 | 0.371097829 | 0.083739495 |
| DALYs | Palestine | Both | 10 to 19 | Pertussis | Number | 2021 | 4.829940247 | 15.46925585 | 0.577621312 |
| DALYs | Palestine | Both | 10 to 19 | Pertussis | Rate | 2021 | 0.418738414 | 1.341128738 | 0.050077686 |
| DALYs | Austria | Both | 10 to 19 | Pertussis | Number | 2021 | 0.093978196 | 0.185148254 | 0.042193983 |
| DALYs | Austria | Both | 10 to 19 | Pertussis | Rate | 2021 | 0.010580382 | 0.020844614 | 0.004750341 |
| DALYs | Burkina Faso | Both | 10 to 19 | Pertussis | Number | 2021 | 354.2959514 | 1389.360769 | 23.91483428 |
| DALYs | Burkina Faso | Both | 10 to 19 | Pertussis | Rate | 2021 | 6.675916957 | 26.17940477 | 0.450621711 |
| DALYs | Colombia | Both | 10 to 19 | Pertussis | Number | 2021 | 2.94108722 | 7.358532725 | 0.89741082 |
| DALYs | Colombia | Both | 10 to 19 | Pertussis | Rate | 2021 | 0.038621323 | 0.096629664 | 0.011784483 |
| DALYs | Djibouti | Both | 10 to 19 | Pertussis | Number | 2021 | 118.4656163 | 384.3233889 | 10.60578435 |
| DALYs | Djibouti | Both | 10 to 19 | Pertussis | Rate | 2021 | 48.55579369 | 157.5235732 | 4.347018931 |
| DALYs | Armenia | Both | 10 to 19 | Pertussis | Number | 2021 | 0.417944415 | 0.978864973 | 0.123167972 |
| DALYs | Armenia | Both | 10 to 19 | Pertussis | Rate | 2021 | 0.113030948 | 0.264729069 | 0.033310153 |
| DALYs | Guatemala | Both | 10 to 19 | Pertussis | Number | 2021 | 9.312314962 | 26.33759773 | 1.365206735 |
| DALYs | Guatemala | Both | 10 to 19 | Pertussis | Rate | 2021 | 0.279983943 | 0.791865878 | 0.041046288 |
| DALYs | India | Both | 10 to 19 | Pertussis | Number | 2021 | 32593.30051 | 104374.4173 | 3142.109794 |
| DALYs | India | Both | 10 to 19 | Pertussis | Rate | 2021 | 12.23167326 | 39.16982166 | 1.179176694 |
| DALYs | Maldives | Both | 10 to 19 | Pertussis | Number | 2021 | 0.562620663 | 1.883478318 | 0.054649867 |
| DALYs | Maldives | Both | 10 to 19 | Pertussis | Rate | 2021 | 0.883469117 | 2.957578768 | 0.085815316 |
| DALYs | Ukraine | Both | 10 to 19 | Pertussis | Number | 2021 | 1.020450627 | 1.961615757 | 0.441470657 |
| DALYs | Ukraine | Both | 10 to 19 | Pertussis | Rate | 2021 | 0.022279635 | 0.04282822 | 0.009638688 |
| DALYs | Poland | Both | 10 to 19 | Pertussis | Number | 2021 | 0.087299135 | 0.323370422 | 0.009270561 |
| DALYs | Poland | Both | 10 to 19 | Pertussis | Rate | 2021 | 0.00225388 | 0.008348742 | 0.000239346 |
| DALYs | Uganda | Both | 10 to 19 | Pertussis | Number | 2021 | 2778.80073 | 11864.81606 | 201.0636227 |
| DALYs | Uganda | Both | 10 to 19 | Pertussis | Rate | 2021 | 25.19157652 | 107.5620207 | 1.822768211 |
| DALYs | Guinea | Both | 10 to 19 | Pertussis | Number | 2021 | 1258.46456 | 4511.416035 | 116.6097102 |
| DALYs | Guinea | Both | 10 to 19 | Pertussis | Rate | 2021 | 39.76983624 | 142.5691932 | 3.685085164 |
| DALYs | Netherlands | Both | 10 to 19 | Pertussis | Number | 2021 | 0.18015014 | 0.564226714 | 0.02247569 |
| DALYs | Netherlands | Both | 10 to 19 | Pertussis | Rate | 2021 | 0.009260322 | 0.029003146 | 0.001155326 |
| DALYs | Rwanda | Both | 10 to 19 | Pertussis | Number | 2021 | 190.3576657 | 662.3421157 | 19.12912358 |
| DALYs | Rwanda | Both | 10 to 19 | Pertussis | Rate | 2021 | 6.271631172 | 21.82189745 | 0.63023891 |
| DALYs | Marshall Islands | Both | 10 to 19 | Pertussis | Number | 2021 | 5.383503967 | 17.15087585 | 0.540868922 |
| DALYs | Marshall Islands | Both | 10 to 19 | Pertussis | Rate | 2021 | 46.60570514 | 148.4773983 | 4.682373723 |
| DALYs | Honduras | Both | 10 to 19 | Pertussis | Number | 2021 | 117.420913 | 478.9679699 | 6.166762337 |
| DALYs | Honduras | Both | 10 to 19 | Pertussis | Rate | 2021 | 5.434678357 | 22.16842634 | 0.285420791 |
| DALYs | Trinidad and Tobago | Both | 10 to 19 | Pertussis | Number | 2021 | 0.004076673 | 0.018300505 | 0.001013 |
| DALYs | Trinidad and Tobago | Both | 10 to 19 | Pertussis | Rate | 2021 | 0.00216246 | 0.00970745 | 0.000537343 |
| DALYs | Saudi Arabia | Both | 10 to 19 | Pertussis | Number | 2021 | 54.61285128 | 178.142301 | 5.866189328 |
| DALYs | Saudi Arabia | Both | 10 to 19 | Pertussis | Rate | 2021 | 1.071664794 | 3.495675978 | 0.115111891 |
| DALYs | Gabon | Both | 10 to 19 | Pertussis | Number | 2021 | 101.2274396 | 327.1237321 | 8.31693033 |
| DALYs | Gabon | Both | 10 to 19 | Pertussis | Rate | 2021 | 25.61099544 | 82.76376883 | 2.10422061 |
| DALYs | Barbados | Both | 10 to 19 | Pertussis | Number | 2021 | 0.000426968 | 0.001712178 | 9.42E-05 |
| DALYs | Barbados | Both | 10 to 19 | Pertussis | Rate | 2021 | 0.001141097 | 0.00457589 | 0.00025188 |
| DALYs | Turkey | Both | 10 to 19 | Pertussis | Number | 2021 | 169.0432232 | 550.7522811 | 19.83534777 |
| DALYs | Turkey | Both | 10 to 19 | Pertussis | Rate | 2021 | 1.336248823 | 4.353573444 | 0.156793982 |
| DALYs | Egypt | Both | 10 to 19 | Pertussis | Number | 2021 | 393.1816218 | 1545.480515 | 21.46639208 |
| DALYs | Egypt | Both | 10 to 19 | Pertussis | Rate | 2021 | 1.891035996 | 7.433102474 | 0.103244195 |
| DALYs | Iraq | Both | 10 to 19 | Pertussis | Number | 2021 | 76.76884556 | 441.1372566 | 4.451642355 |
| DALYs | Iraq | Both | 10 to 19 | Pertussis | Rate | 2021 | 0.882515895 | 5.07120614 | 0.051174993 |
| DALYs | Saint Lucia | Both | 10 to 19 | Pertussis | Number | 2021 | 0.000253894 | 0.001151616 | 6.19E-05 |
| DALYs | Saint Lucia | Both | 10 to 19 | Pertussis | Rate | 2021 | 0.001095628 | 0.004969575 | 0.000267313 |
| DALYs | Canada | Both | 10 to 19 | Pertussis | Number | 2021 | 0.683142938 | 1.764784773 | 0.147795394 |
| DALYs | Canada | Both | 10 to 19 | Pertussis | Rate | 2021 | 0.016151801 | 0.041725459 | 0.003494381 |
| DALYs | Comoros | Both | 10 to 19 | Pertussis | Number | 2021 | 28.75064975 | 87.09108352 | 2.543890196 |
| DALYs | Comoros | Both | 10 to 19 | Pertussis | Rate | 2021 | 18.90166328 | 57.25666549 | 1.672440669 |
| DALYs | Chile | Both | 10 to 19 | Pertussis | Number | 2021 | 0.243567442 | 0.574498357 | 0.07943884 |
| DALYs | Chile | Both | 10 to 19 | Pertussis | Rate | 2021 | 0.009641164 | 0.022740448 | 0.003144439 |
| DALYs | Montenegro | Both | 10 to 19 | Pertussis | Number | 2021 | 0.214386432 | 0.860325568 | 0.013231313 |
| DALYs | Montenegro | Both | 10 to 19 | Pertussis | Rate | 2021 | 0.278015632 | 1.115667413 | 0.017158324 |
| DALYs | United Republic of Tanzania | Both | 10 to 19 | Pertussis | Number | 2021 | 2138.019215 | 7538.593957 | 192.0068506 |
| DALYs | United Republic of Tanzania | Both | 10 to 19 | Pertussis | Rate | 2021 | 15.44286518 | 54.4510963 | 1.386861206 |
| DALYs | Nicaragua | Both | 10 to 19 | Pertussis | Number | 2021 | 0.463417018 | 1.553045443 | 0.034252071 |
| DALYs | Nicaragua | Both | 10 to 19 | Pertussis | Rate | 2021 | 0.036425443 | 0.122072271 | 0.002692277 |
| DALYs | Central African Republic | Both | 10 to 19 | Pertussis | Number | 2021 | 1300.449319 | 4334.920106 | 101.2416046 |
| DALYs | Central African Republic | Both | 10 to 19 | Pertussis | Rate | 2021 | 99.87984551 | 332.9396571 | 7.775770789 |
| DALYs | Ecuador | Both | 10 to 19 | Pertussis | Number | 2021 | 8.034757103 | 20.64750975 | 2.463489855 |
| DALYs | Ecuador | Both | 10 to 19 | Pertussis | Rate | 2021 | 0.247944081 | 0.637160248 | 0.076020684 |
| DALYs | Thailand | Both | 10 to 19 | Pertussis | Number | 2021 | 18.40707199 | 68.68463624 | 0.400222099 |
| DALYs | Thailand | Both | 10 to 19 | Pertussis | Rate | 2021 | 0.243415071 | 0.908285447 | 0.005292536 |
| DALYs | Vanuatu | Both | 10 to 19 | Pertussis | Number | 2021 | 0.135178416 | 0.486992081 | 0.00880383 |
| DALYs | Vanuatu | Both | 10 to 19 | Pertussis | Rate | 2021 | 0.206348917 | 0.743390044 | 0.013438986 |
| DALYs | Russian Federation | Both | 10 to 19 | Pertussis | Number | 2021 | 2.35902568 | 5.602096996 | 0.577892819 |
| DALYs | Russian Federation | Both | 10 to 19 | Pertussis | Rate | 2021 | 0.014174993 | 0.033662069 | 0.003472462 |
| DALYs | Papua New Guinea | Both | 10 to 19 | Pertussis | Number | 2021 | 0.857248062 | 1.711768425 | 0.337201757 |
| DALYs | Papua New Guinea | Both | 10 to 19 | Pertussis | Rate | 2021 | 0.040830485 | 0.08153105 | 0.016060825 |
| DALYs | Italy | Both | 10 to 19 | Pertussis | Number | 2021 | 0.047108352 | 0.091842609 | 0.02294184 |
| DALYs | Italy | Both | 10 to 19 | Pertussis | Rate | 2021 | 0.00082183 | 0.001602242 | 0.000400232 |
| DALYs | Yemen | Both | 10 to 19 | Pertussis | Number | 2021 | 459.8901941 | 1983.931049 | 19.39104422 |
| DALYs | Yemen | Both | 10 to 19 | Pertussis | Rate | 2021 | 5.669250712 | 24.45671305 | 0.239041173 |
| DALYs | Andorra | Both | 10 to 19 | Pertussis | Number | 2021 | 0.003349858 | 0.010471493 | 0.000422376 |
| DALYs | Andorra | Both | 10 to 19 | Pertussis | Rate | 2021 | 0.039498869 | 0.123471548 | 0.004980325 |
| DALYs | Cameroon | Both | 10 to 19 | Pertussis | Number | 2021 | 1491.123853 | 5656.714348 | 104.8035695 |
| DALYs | Cameroon | Both | 10 to 19 | Pertussis | Rate | 2021 | 19.91046145 | 75.5321516 | 1.399405842 |
| DALYs | Cyprus | Both | 10 to 19 | Pertussis | Number | 2021 | 0.085337161 | 0.320872206 | 0.007496668 |
| DALYs | Cyprus | Both | 10 to 19 | Pertussis | Rate | 2021 | 0.062291446 | 0.234219106 | 0.005472156 |
| DALYs | Kazakhstan | Both | 10 to 19 | Pertussis | Number | 2021 | 0.686982646 | 1.522462627 | 0.242800653 |
| DALYs | Kazakhstan | Both | 10 to 19 | Pertussis | Rate | 2021 | 0.023338799 | 0.051722484 | 0.008248645 |
| DALYs | Kuwait | Both | 10 to 19 | Pertussis | Number | 2021 | 0.192264088 | 0.395138738 | 0.078475452 |
| DALYs | Kuwait | Both | 10 to 19 | Pertussis | Rate | 2021 | 0.035811352 | 0.073599041 | 0.014616937 |
| DALYs | Algeria | Both | 10 to 19 | Pertussis | Number | 2021 | 181.7938255 | 597.3051276 | 20.26924733 |
| DALYs | Algeria | Both | 10 to 19 | Pertussis | Rate | 2021 | 2.561116356 | 8.414850876 | 0.285553707 |
| DALYs | Tonga | Both | 10 to 19 | Pertussis | Number | 2021 | 0.169263539 | 0.518005695 | 0.018046304 |
| DALYs | Tonga | Both | 10 to 19 | Pertussis | Rate | 2021 | 0.762314905 | 2.332950529 | 0.081275427 |
| DALYs | Bulgaria | Both | 10 to 19 | Pertussis | Number | 2021 | 0.017783748 | 0.041065236 | 0.006188868 |
| DALYs | Bulgaria | Both | 10 to 19 | Pertussis | Rate | 2021 | 0.002667864 | 0.006160482 | 0.000928435 |
| DALYs | Ethiopia | Both | 10 to 19 | Pertussis | Number | 2021 | 8306.087306 | 28977.7637 | 712.4302852 |
| DALYs | Ethiopia | Both | 10 to 19 | Pertussis | Rate | 2021 | 31.10057718 | 108.5017702 | 2.667560821 |
| DALYs | Somalia | Both | 10 to 19 | Pertussis | Number | 2021 | 5249.722181 | 15810.40146 | 438.0592297 |
| DALYs | Somalia | Both | 10 to 19 | Pertussis | Rate | 2021 | 99.79271653 | 300.5421729 | 8.32713028 |
| DALYs | Uzbekistan | Both | 10 to 19 | Pertussis | Number | 2021 | 1.020360331 | 1.957338467 | 0.412307882 |
| DALYs | Uzbekistan | Both | 10 to 19 | Pertussis | Rate | 2021 | 0.01889773 | 0.036251167 | 0.007636207 |
| DALYs | El Salvador | Both | 10 to 19 | Pertussis | Number | 2021 | 0.699215652 | 2.412275022 | 0.033467412 |
| DALYs | El Salvador | Both | 10 to 19 | Pertussis | Rate | 2021 | 0.060741025 | 0.209554889 | 0.002907322 |
| DALYs | Togo | Both | 10 to 19 | Pertussis | Number | 2021 | 492.0049317 | 1566.056386 | 40.82128034 |
| DALYs | Togo | Both | 10 to 19 | Pertussis | Rate | 2021 | 26.23623161 | 83.51017521 | 2.176800468 |
| DALYs | Qatar | Both | 10 to 19 | Pertussis | Number | 2021 | 0.450935804 | 1.301760976 | 0.065750637 |
| DALYs | Qatar | Both | 10 to 19 | Pertussis | Rate | 2021 | 0.183730238 | 0.530392245 | 0.026789578 |
| DALYs | Mauritania | Both | 10 to 19 | Pertussis | Number | 2021 | 198.4954348 | 666.4252236 | 19.89352995 |
| DALYs | Mauritania | Both | 10 to 19 | Pertussis | Rate | 2021 | 19.00196286 | 63.79686949 | 1.904407109 |
| DALYs | Pakistan | Both | 10 to 19 | Pertussis | Number | 2021 | 4957.571548 | 20707.88667 | 300.7931732 |
| DALYs | Pakistan | Both | 10 to 19 | Pertussis | Rate | 2021 | 9.806351916 | 40.96135016 | 0.594985606 |
| DALYs | Burundi | Both | 10 to 19 | Pertussis | Number | 2021 | 258.822404 | 849.0998948 | 19.84753151 |
| DALYs | Burundi | Both | 10 to 19 | Pertussis | Rate | 2021 | 8.202636092 | 26.9097935 | 0.629010765 |
| DALYs | Cambodia | Both | 10 to 19 | Pertussis | Number | 2021 | 15.49789476 | 83.23509486 | 0.47310638 |
| DALYs | Cambodia | Both | 10 to 19 | Pertussis | Rate | 2021 | 0.489470509 | 2.62881668 | 0.014942134 |
| DALYs | Lesotho | Both | 10 to 19 | Pertussis | Number | 2021 | 11.22082339 | 67.96786478 | 0.093645716 |
| DALYs | Lesotho | Both | 10 to 19 | Pertussis | Rate | 2021 | 2.657527919 | 16.09743706 | 0.022178952 |
| DALYs | Monaco | Both | 10 to 19 | Pertussis | Number | 2021 | 0.001340318 | 0.005541686 | 3.86E-05 |
| DALYs | Monaco | Both | 10 to 19 | Pertussis | Rate | 2021 | 0.038774107 | 0.160315578 | 0.001117208 |
| DALYs | Nigeria | Both | 10 to 19 | Pertussis | Number | 2021 | 12712.03198 | 42112.41024 | 1008.107951 |
| DALYs | Nigeria | Both | 10 to 19 | Pertussis | Rate | 2021 | 22.3509967 | 74.04436547 | 1.772511076 |
| DALYs | Syrian Arab Republic | Both | 10 to 19 | Pertussis | Number | 2021 | 280.702772 | 879.8722111 | 27.5329416 |
| DALYs | Syrian Arab Republic | Both | 10 to 19 | Pertussis | Rate | 2021 | 8.403696651 | 26.34166774 | 0.824282879 |
| DALYs | Malta | Both | 10 to 19 | Pertussis | Number | 2021 | 0.00184554 | 0.003517846 | 0.000847641 |
| DALYs | Malta | Both | 10 to 19 | Pertussis | Rate | 2021 | 0.004556188 | 0.008684703 | 0.002092619 |
| DALYs | Venezuela (Bolivarian Republic of) | Both | 10 to 19 | Pertussis | Number | 2021 | 2.898361305 | 5.044612872 | 1.477986135 |
| DALYs | Venezuela (Bolivarian Republic of) | Both | 10 to 19 | Pertussis | Rate | 2021 | 0.066557698 | 0.115844018 | 0.033940335 |
| DALYs | Serbia | Both | 10 to 19 | Pertussis | Number | 2021 | 4.988003337 | 18.13617035 | 0.138226234 |
| DALYs | Serbia | Both | 10 to 19 | Pertussis | Rate | 2021 | 0.460321309 | 1.673708918 | 0.012756303 |
| DALYs | Gambia | Both | 10 to 19 | Pertussis | Number | 2021 | 57.16299593 | 184.0477739 | 3.76006559 |
| DALYs | Gambia | Both | 10 to 19 | Pertussis | Rate | 2021 | 9.861731257 | 31.75182922 | 0.648684621 |
| DALYs | Palau | Both | 10 to 19 | Pertussis | Number | 2021 | 0.188541 | 0.618069366 | 0.016376711 |
| DALYs | Palau | Both | 10 to 19 | Pertussis | Rate | 2021 | 7.872709433 | 25.80807643 | 0.683825222 |
| DALYs | Dominica | Both | 10 to 19 | Pertussis | Number | 2021 | 0.00391841 | 0.015406724 | 2.39E-05 |
| DALYs | Dominica | Both | 10 to 19 | Pertussis | Rate | 2021 | 0.034859111 | 0.13706188 | 0.000212446 |
| DALYs | Grenada | Both | 10 to 19 | Pertussis | Number | 2021 | 0.000377926 | 0.001139302 | 0.000105729 |
| DALYs | Grenada | Both | 10 to 19 | Pertussis | Rate | 2021 | 0.002354269 | 0.00709721 | 0.000658632 |
| DALYs | Madagascar | Both | 10 to 19 | Pertussis | Number | 2021 | 1406.060875 | 6125.39352 | 46.04645325 |
| DALYs | Madagascar | Both | 10 to 19 | Pertussis | Rate | 2021 | 20.47165432 | 89.18315058 | 0.670416972 |
| DALYs | Benin | Both | 10 to 19 | Pertussis | Number | 2021 | 443.7265723 | 1536.961636 | 34.53792567 |
| DALYs | Benin | Both | 10 to 19 | Pertussis | Rate | 2021 | 14.11476659 | 48.89014114 | 1.098637742 |
| DALYs | Suriname | Both | 10 to 19 | Pertussis | Number | 2021 | 0.749756789 | 3.326492918 | 0.000823442 |
| DALYs | Suriname | Both | 10 to 19 | Pertussis | Rate | 2021 | 0.776092409 | 3.443337815 | 0.000852366 |
| DALYs | Northern Mariana Islands | Both | 10 to 19 | Pertussis | Number | 2021 | 0.018934834 | 0.066573949 | 0.00164513 |
| DALYs | Northern Mariana Islands | Both | 10 to 19 | Pertussis | Rate | 2021 | 0.235313408 | 0.827350398 | 0.020444918 |
| DALYs | Nepal | Both | 10 to 19 | Pertussis | Number | 2021 | 430.2296091 | 1568.814135 | 25.59857958 |
| DALYs | Nepal | Both | 10 to 19 | Pertussis | Rate | 2021 | 6.812520115 | 24.84156744 | 0.405343646 |
| DALYs | Nauru | Both | 10 to 19 | Pertussis | Number | 2021 | 0.09936773 | 0.356967013 | 0.009828786 |
| DALYs | Nauru | Both | 10 to 19 | Pertussis | Rate | 2021 | 4.13957554 | 14.87094366 | 0.409458897 |
| DALYs | South Sudan | Both | 10 to 19 | Pertussis | Number | 2021 | 1155.920812 | 4122.333983 | 106.7326676 |
| DALYs | South Sudan | Both | 10 to 19 | Pertussis | Rate | 2021 | 45.25596337 | 161.3953082 | 4.178737544 |
| DALYs | Australia | Both | 10 to 19 | Pertussis | Number | 2021 | 0.04177337 | 0.099456227 | 0.009834719 |
| DALYs | Australia | Both | 10 to 19 | Pertussis | Rate | 2021 | 0.001342353 | 0.003195944 | 0.000316031 |
| DALYs | Guam | Both | 10 to 19 | Pertussis | Number | 2021 | 0.116766518 | 0.35709368 | 0.009791715 |
| DALYs | Guam | Both | 10 to 19 | Pertussis | Rate | 2021 | 0.484300037 | 1.481079378 | 0.040612053 |
| DALYs | Fiji | Both | 10 to 19 | Pertussis | Number | 2021 | 0.814229545 | 3.42555749 | 0.011379295 |
| DALYs | Fiji | Both | 10 to 19 | Pertussis | Rate | 2021 | 0.489262296 | 2.058382841 | 0.006837703 |
| DALYs | Bermuda | Both | 10 to 19 | Pertussis | Number | 2021 | 1.14E-05 | 4.99E-05 | 2.73E-06 |
| DALYs | Bermuda | Both | 10 to 19 | Pertussis | Rate | 2021 | 0.000183828 | 0.000808229 | 4.42E-05 |
| DALYs | Latvia | Both | 10 to 19 | Pertussis | Number | 2021 | 0.000148201 | 0.000779382 | 8.03E-06 |
| DALYs | Latvia | Both | 10 to 19 | Pertussis | Rate | 2021 | 7.79E-05 | 0.000409929 | 4.22E-06 |
| DALYs | Iran (Islamic Republic of) | Both | 10 to 19 | Pertussis | Number | 2021 | 221.0675111 | 711.8370746 | 26.0778456 |
| DALYs | Iran (Islamic Republic of) | Both | 10 to 19 | Pertussis | Rate | 2021 | 1.797667431 | 5.788486598 | 0.212058721 |
| DALYs | Israel | Both | 10 to 19 | Pertussis | Number | 2021 | 0.068621617 | 0.132010636 | 0.029239977 |
| DALYs | Israel | Both | 10 to 19 | Pertussis | Rate | 2021 | 0.004391836 | 0.008448781 | 0.001871381 |
| DALYs | Greenland | Both | 10 to 19 | Pertussis | Number | 2021 | 0.111221395 | 0.29908887 | 0.013682468 |
| DALYs | Greenland | Both | 10 to 19 | Pertussis | Rate | 2021 | 1.500813256 | 4.035883052 | 0.184630215 |
| DALYs | Azerbaijan | Both | 10 to 19 | Pertussis | Number | 2021 | 63.90056191 | 189.4271028 | 6.178914511 |
| DALYs | Azerbaijan | Both | 10 to 19 | Pertussis | Rate | 2021 | 4.296722255 | 12.73722208 | 0.415474899 |
| DALYs | Georgia | Both | 10 to 19 | Pertussis | Number | 2021 | 0.023952329 | 0.064382499 | 0.006288758 |
| DALYs | Georgia | Both | 10 to 19 | Pertussis | Rate | 2021 | 0.005640505 | 0.015161356 | 0.001480932 |
| DALYs | Croatia | Both | 10 to 19 | Pertussis | Number | 2021 | 0.009327248 | 0.021437794 | 0.003352813 |
| DALYs | Croatia | Both | 10 to 19 | Pertussis | Rate | 2021 | 0.002190692 | 0.005035098 | 0.000787476 |
| DALYs | Kiribati | Both | 10 to 19 | Pertussis | Number | 2021 | 31.24582007 | 95.75456662 | 2.857554701 |
| DALYs | Kiribati | Both | 10 to 19 | Pertussis | Rate | 2021 | 125.6831361 | 385.1630139 | 11.49422341 |
| DALYs | Chad | Both | 10 to 19 | Pertussis | Number | 2021 | 2085.991542 | 6671.027011 | 134.7351694 |
| DALYs | Chad | Both | 10 to 19 | Pertussis | Rate | 2021 | 48.10592634 | 153.8433534 | 3.107184284 |
| DALYs | Indonesia | Both | 10 to 19 | Pertussis | Number | 2021 | 1228.059801 | 5350.920157 | 67.35060042 |
| DALYs | Indonesia | Both | 10 to 19 | Pertussis | Rate | 2021 | 2.685415285 | 11.70093083 | 0.147276486 |
| DALYs | Malawi | Both | 10 to 19 | Pertussis | Number | 2021 | 534.0525049 | 1799.622679 | 51.61602093 |
| DALYs | Malawi | Both | 10 to 19 | Pertussis | Rate | 2021 | 10.32081577 | 34.77855445 | 0.997503874 |
| DALYs | Mexico | Both | 10 to 19 | Pertussis | Number | 2021 | 34.70211519 | 54.10757869 | 19.07530807 |
| DALYs | Mexico | Both | 10 to 19 | Pertussis | Rate | 2021 | 0.15520901 | 0.242002071 | 0.085316404 |
| DALYs | Mongolia | Both | 10 to 19 | Pertussis | Number | 2021 | 6.116759929 | 25.97833242 | 0.010208844 |
| DALYs | Mongolia | Both | 10 to 19 | Pertussis | Rate | 2021 | 1.152352475 | 4.894126305 | 0.001923271 |
| DALYs | Argentina | Both | 10 to 19 | Pertussis | Number | 2021 | 1.570676973 | 3.615727882 | 0.534009844 |
| DALYs | Argentina | Both | 10 to 19 | Pertussis | Rate | 2021 | 0.022200622 | 0.051106248 | 0.007547924 |
| DALYs | Tunisia | Both | 10 to 19 | Pertussis | Number | 2021 | 0.832959516 | 5.831797589 | 0.218150461 |
| DALYs | Tunisia | Both | 10 to 19 | Pertussis | Rate | 2021 | 0.048152191 | 0.337127827 | 0.012610964 |
| DALYs | Cuba | Both | 10 to 19 | Pertussis | Number | 2021 | 0.007435439 | 0.033368262 | 0.001814747 |
| DALYs | Cuba | Both | 10 to 19 | Pertussis | Rate | 2021 | 0.000599779 | 0.002691648 | 0.000146386 |
| DALYs | China | Both | 10 to 19 | Pertussis | Number | 2021 | 3326.007679 | 9226.253833 | 357.7589119 |
| DALYs | China | Both | 10 to 19 | Pertussis | Rate | 2021 | 2.067575555 | 5.735397727 | 0.222396835 |
| DALYs | Eritrea | Both | 10 to 19 | Pertussis | Number | 2021 | 238.8651553 | 739.7111657 | 21.33933258 |
| DALYs | Eritrea | Both | 10 to 19 | Pertussis | Rate | 2021 | 16.43754487 | 50.90334528 | 1.468469674 |
| DALYs | Czechia | Both | 10 to 19 | Pertussis | Number | 2021 | 0.016426706 | 0.036996359 | 0.005810889 |
| DALYs | Czechia | Both | 10 to 19 | Pertussis | Rate | 2021 | 0.00149822 | 0.003374302 | 0.00052999 |
| DALYs | Zambia | Both | 10 to 19 | Pertussis | Number | 2021 | 878.590266 | 2939.892927 | 75.2525871 |
| DALYs | Zambia | Both | 10 to 19 | Pertussis | Rate | 2021 | 18.61145618 | 62.27668403 | 1.594099413 |
| DALYs | Lebanon | Both | 10 to 19 | Pertussis | Number | 2021 | 7.542086983 | 35.64028906 | 0.707386249 |
| DALYs | Lebanon | Both | 10 to 19 | Pertussis | Rate | 2021 | 0.92248362 | 4.35921555 | 0.08652144 |
| DALYs | Bahamas | Both | 10 to 19 | Pertussis | Number | 2021 | 0.000802441 | 0.003333669 | 0.00021107 |
| DALYs | Bahamas | Both | 10 to 19 | Pertussis | Rate | 2021 | 0.001178765 | 0.004897073 | 0.000310056 |
| DALYs | Switzerland | Both | 10 to 19 | Pertussis | Number | 2021 | 0.043407508 | 0.085144577 | 0.018703125 |
| DALYs | Switzerland | Both | 10 to 19 | Pertussis | Rate | 2021 | 0.00499122 | 0.009790364 | 0.002150582 |
| DALYs | Bolivia (Plurinational State of) | Both | 10 to 19 | Pertussis | Number | 2021 | 8.866209046 | 38.93077997 | 0.075275345 |
| DALYs | Bolivia (Plurinational State of) | Both | 10 to 19 | Pertussis | Rate | 2021 | 0.407379454 | 1.788769 | 0.003458708 |
| DALYs | Solomon Islands | Both | 10 to 19 | Pertussis | Number | 2021 | 51.454308 | 164.6257683 | 4.234187024 |
| DALYs | Solomon Islands | Both | 10 to 19 | Pertussis | Rate | 2021 | 34.41473897 | 110.1084256 | 2.831996908 |
| DALYs | Philippines | Both | 10 to 19 | Pertussis | Number | 2021 | 836.4825195 | 3236.752624 | 66.22356984 |
| DALYs | Philippines | Both | 10 to 19 | Pertussis | Rate | 2021 | 3.77732599 | 14.61628848 | 0.299047506 |
| DALYs | Senegal | Both | 10 to 19 | Pertussis | Number | 2021 | 391.2616749 | 1428.529623 | 42.29119698 |
| DALYs | Senegal | Both | 10 to 19 | Pertussis | Rate | 2021 | 10.47792415 | 38.25579143 | 1.132551391 |
| DALYs | Congo | Both | 10 to 19 | Pertussis | Number | 2021 | 465.6958864 | 1700.17404 | 48.05737816 |
| DALYs | Congo | Both | 10 to 19 | Pertussis | Rate | 2021 | 38.93542312 | 142.1464041 | 4.01793189 |
| DALYs | Tuvalu | Both | 10 to 19 | Pertussis | Number | 2021 | 0.415990804 | 1.231659252 | 0.04746373 |
| DALYs | Tuvalu | Both | 10 to 19 | Pertussis | Rate | 2021 | 17.42101842 | 51.5798866 | 1.987703843 |
| DALYs | Estonia | Both | 10 to 19 | Pertussis | Number | 2021 | 0.005749542 | 0.016399006 | 0.001149231 |
| DALYs | Estonia | Both | 10 to 19 | Pertussis | Rate | 2021 | 0.004098331 | 0.011689377 | 0.000819183 |
| DALYs | Eswatini | Both | 10 to 19 | Pertussis | Number | 2021 | 13.15511978 | 51.29535486 | 0.441328221 |
| DALYs | Eswatini | Both | 10 to 19 | Pertussis | Rate | 2021 | 5.139737699 | 20.04122149 | 0.172428023 |
| DALYs | Afghanistan | Both | 10 to 19 | Pertussis | Number | 2021 | 1912.985166 | 6109.472366 | 187.7727324 |
| DALYs | Afghanistan | Both | 10 to 19 | Pertussis | Rate | 2021 | 25.1898367 | 80.44840805 | 2.472556793 |
| DALYs | Saint Kitts and Nevis | Both | 10 to 19 | Pertussis | Number | 2021 | 3.92E-05 | 0.000173152 | 9.81E-06 |
| DALYs | Saint Kitts and Nevis | Both | 10 to 19 | Pertussis | Rate | 2021 | 0.000506633 | 0.002238507 | 0.000126772 |
| DALYs | Belgium | Both | 10 to 19 | Pertussis | Number | 2021 | 0.086839155 | 0.208701427 | 0.026388199 |
| DALYs | Belgium | Both | 10 to 19 | Pertussis | Rate | 2021 | 0.006660299 | 0.016006764 | 0.002023895 |
| DALYs | Luxembourg | Both | 10 to 19 | Pertussis | Number | 2021 | 0.001095385 | 0.00214596 | 0.000459174 |
| DALYs | Luxembourg | Both | 10 to 19 | Pertussis | Rate | 2021 | 0.001613649 | 0.003161287 | 0.000676425 |
| DALYs | Turkmenistan | Both | 10 to 19 | Pertussis | Number | 2021 | 0.064566716 | 0.162741942 | 0.020149778 |
| DALYs | Turkmenistan | Both | 10 to 19 | Pertussis | Rate | 2021 | 0.007127593 | 0.017965268 | 0.002224357 |
| DALYs | Democratic Republic of the Congo | Both | 10 to 19 | Pertussis | Number | 2021 | 11054.20675 | 41242.8375 | 647.1690947 |
| DALYs | Democratic Republic of the Congo | Both | 10 to 19 | Pertussis | Rate | 2021 | 51.44810567 | 191.950984 | 3.012031956 |
| DALYs | Paraguay | Both | 10 to 19 | Pertussis | Number | 2021 | 29.09619757 | 98.14485556 | 2.394751424 |
| DALYs | Paraguay | Both | 10 to 19 | Pertussis | Rate | 2021 | 2.165397012 | 7.304135752 | 0.178222174 |
| DALYs | Morocco | Both | 10 to 19 | Pertussis | Number | 2021 | 181.1917632 | 563.6827725 | 14.71547503 |
| DALYs | Morocco | Both | 10 to 19 | Pertussis | Rate | 2021 | 2.84413673 | 8.848033977 | 0.230986345 |
| DALYs | Mozambique | Both | 10 to 19 | Pertussis | Number | 2021 | 823.0639522 | 3001.652764 | 65.98685959 |
| DALYs | Mozambique | Both | 10 to 19 | Pertussis | Rate | 2021 | 10.71038973 | 39.05999143 | 0.858675661 |
| DALYs | New Zealand | Both | 10 to 19 | Pertussis | Number | 2021 | 0.008515058 | 0.020207201 | 0.002116382 |
| DALYs | New Zealand | Both | 10 to 19 | Pertussis | Rate | 2021 | 0.001292665 | 0.003067642 | 0.000321287 |
| DALYs | Romania | Both | 10 to 19 | Pertussis | Number | 2021 | 0.046858569 | 0.170184417 | 0.005784915 |
| DALYs | Romania | Both | 10 to 19 | Pertussis | Rate | 2021 | 0.002211467 | 0.008031771 | 0.000273016 |
| DALYs | Tokelau | Both | 10 to 19 | Pertussis | Number | 2021 | 0.019797227 | 0.061803353 | 0.001823581 |
| DALYs | Tokelau | Both | 10 to 19 | Pertussis | Rate | 2021 | 7.380825652 | 23.04159979 | 0.679869658 |
| DALYs | Libya | Both | 10 to 19 | Pertussis | Number | 2021 | 38.14622866 | 114.4664791 | 4.366364473 |
| DALYs | Libya | Both | 10 to 19 | Pertussis | Rate | 2021 | 3.277658505 | 9.835363601 | 0.375173435 |
| DALYs | Equatorial Guinea | Both | 10 to 19 | Pertussis | Number | 2021 | 123.571595 | 439.6448325 | 9.066480876 |
| DALYs | Equatorial Guinea | Both | 10 to 19 | Pertussis | Rate | 2021 | 32.19255934 | 114.5351596 | 2.361976663 |
| DALYs | Guinea-Bissau | Both | 10 to 19 | Pertussis | Number | 2021 | 173.2996429 | 636.8284624 | 12.39160352 |
| DALYs | Guinea-Bissau | Both | 10 to 19 | Pertussis | Rate | 2021 | 35.82458351 | 131.6454786 | 2.561598095 |
| DALYs | Dominican Republic | Both | 10 to 19 | Pertussis | Number | 2021 | 26.62634255 | 114.0801266 | 0.015324795 |
| DALYs | Dominican Republic | Both | 10 to 19 | Pertussis | Rate | 2021 | 1.414308468 | 6.059581363 | 0.000814005 |
| DALYs | Brunei Darussalam | Both | 10 to 19 | Pertussis | Number | 2021 | 0.036030702 | 0.158890492 | 1.50E-05 |
| DALYs | Brunei Darussalam | Both | 10 to 19 | Pertussis | Rate | 2021 | 0.054399402 | 0.239893956 | 2.26E-05 |
| DALYs | Uruguay | Both | 10 to 19 | Pertussis | Number | 2021 | 0.046635524 | 0.097682459 | 0.018248333 |
| DALYs | Uruguay | Both | 10 to 19 | Pertussis | Rate | 2021 | 0.009899305 | 0.020735019 | 0.003873567 |
| DALYs | Sudan | Both | 10 to 19 | Pertussis | Number | 2021 | 454.8436534 | 1894.547602 | 31.33588169 |
| DALYs | Sudan | Both | 10 to 19 | Pertussis | Rate | 2021 | 4.485450301 | 18.68312122 | 0.30901946 |
| DALYs | Brazil | Both | 10 to 19 | Pertussis | Number | 2021 | 26.74412922 | 65.98572808 | 5.774047081 |
| DALYs | Brazil | Both | 10 to 19 | Pertussis | Rate | 2021 | 0.085368468 | 0.210629424 | 0.018431019 |
| DALYs | Greece | Both | 10 to 19 | Pertussis | Number | 2021 | 0.000883995 | 0.003263239 | 7.22E-05 |
| DALYs | Greece | Both | 10 to 19 | Pertussis | Rate | 2021 | 8.55E-05 | 0.000315437 | 6.98E-06 |
| DALYs | Lithuania | Both | 10 to 19 | Pertussis | Number | 2021 | 0.23469089 | 0.468740327 | 0.094950088 |
| DALYs | Lithuania | Both | 10 to 19 | Pertussis | Rate | 2021 | 0.089465724 | 0.178686923 | 0.036195603 |
| DALYs | Peru | Both | 10 to 19 | Pertussis | Number | 2021 | 487.4759441 | 1918.443849 | 38.09475018 |
| DALYs | Peru | Both | 10 to 19 | Pertussis | Rate | 2021 | 8.113178356 | 31.92911835 | 0.634020009 |
| DALYs | South Africa | Both | 10 to 19 | Pertussis | Number | 2021 | 1017.188355 | 3043.765617 | 89.25060103 |
| DALYs | South Africa | Both | 10 to 19 | Pertussis | Rate | 2021 | 10.39272721 | 31.0984938 | 0.911883375 |
| DALYs | Mauritius | Both | 10 to 19 | Pertussis | Number | 2021 | 0.008777404 | 0.017192272 | 0.003614033 |
| DALYs | Mauritius | Both | 10 to 19 | Pertussis | Rate | 2021 | 0.005292494 | 0.010366391 | 0.002179147 |
| DALYs | Ireland | Both | 10 to 19 | Pertussis | Number | 2021 | 0.071240707 | 0.239039207 | 0.012680813 |
| DALYs | Ireland | Both | 10 to 19 | Pertussis | Rate | 2021 | 0.010426127 | 0.034983553 | 0.001855846 |

Sup_Table 8 EAPC of adolescent pertussis prevalence in 204 countries and territories, 1990-2021

| location | measure | EAPC | LCI | UCI |
| --- | --- | --- | --- | --- |
| Philippines | Prevalence | -2.220592136 | -3.537721641 | -0.885478087 |
| Germany | Prevalence | -8.163008487 | -10.61712932 | -5.641506633 |
| North Macedonia | Prevalence | -1.570022186 | -4.278367052 | 1.21495250393608 |
| Armenia | Prevalence | -2.168253613 | -3.507278195 | -0.810647455 |
| Haiti | Prevalence | -4.552385307 | -7.641653492 | -1.359785065 |
| Lebanon | Prevalence | 1.00423279907509 | -0.410693992 | 2.43926232863749 |
| Ethiopia | Prevalence | -2.468934819 | -3.160088841 | -1.772847976 |
| Tuvalu | Prevalence | -5.895629486 | -7.727269983 | -4.027630394 |
| South Africa | Prevalence | -0.865843652 | -2.492148392 | 0.787585746065345 |
| Brunei Darussalam | Prevalence | -6.666321341 | -10.67925695 | -2.473095558 |
| Mexico | Prevalence | -1.750838299 | -3.320583374 | -0.155605899 |
| Argentina | Prevalence | -4.549483988 | -6.152940605 | -2.918630954 |
| American Samoa | Prevalence | -4.060878346 | -5.976833757 | -2.105880589 |
| Chile | Prevalence | -4.575288372 | -7.089568081 | -1.992968914 |
| Niger | Prevalence | -3.249026826 | -3.780958392 | -2.71415456 |
| Nepal | Prevalence | -5.27187349 | -6.168383847 | -4.366797461 |
| Uruguay | Prevalence | -4.65518975 | -6.528659794 | -2.744169264 |
| Japan | Prevalence | -10.95254824 | -14.33717439 | -7.43419203 |
| India | Prevalence | -3.40146582 | -4.267676799 | -2.527417139 |
| Azerbaijan | Prevalence | -5.742314901 | -7.468442571 | -3.983987226 |
| Sri Lanka | Prevalence | -0.151885684 | -3.09905416 | 2.88491867554337 |
| Greece | Prevalence | -16.35837244 | -19.32226624 | -13.28559275 |
| Kenya | Prevalence | -2.070166896 | -2.808414678 | -1.326311532 |
| Nicaragua | Prevalence | -16.71732733 | -18.84983019 | -14.52878555 |
| Eswatini | Prevalence | -2.704357211 | -4.166662949 | -1.219738381 |
| Panama | Prevalence | -0.158433994 | -1.549261247 | 1.25204166949724 |
| Zimbabwe | Prevalence | -2.21004666 | -4.576582329 | 0.215179960714273 |
| Cook Islands | Prevalence | -6.32218503 | -8.293275921 | -4.30872866 |
| Timor-Leste | Prevalence | -5.107077103 | -6.907879475 | -3.27143946 |
| Pakistan | Prevalence | -3.051412816 | -3.782589035 | -2.314680237 |
| Saint Lucia | Prevalence | -4.458576518 | -7.500509243 | -1.316606976 |
| Bermuda | Prevalence | -6.254379894 | -9.32645128 | -3.078224982 |
| Romania | Prevalence | -0.558420263 | -3.61648065 | 2.59666639411207 |
| Montenegro | Prevalence | -2.083907585 | -4.627207153 | 0.527213973517693 |
| Jamaica | Prevalence | -4.763675064 | -8.131587948 | -1.272293873 |
| Poland | Prevalence | -5.426351246 | -7.507356302 | -3.298525364 |
| Thailand | Prevalence | -2.984019493 | -5.751769293 | -0.134990299 |
| Papua New Guinea | Prevalence | -2.189425903 | -4.039482454 | -0.303701462 |
| Morocco | Prevalence | -3.831654445 | -5.05378329 | -2.5937946 |
| Equatorial Guinea | Prevalence | -1.11944407 | -1.500936607 | -0.736473991 |
| Algeria | Prevalence | -1.55277785 | -2.959237641 | -0.125933541 |
| Iceland | Prevalence | 5.37934821317789 | 4.47657323084252 | 6.28992401289772 |
| Viet Nam | Prevalence | -2.809097223 | -4.800255117 | -0.776293106 |
| Libya | Prevalence | -0.128255021 | -1.258025779 | 1.01444217531788 |
| Micronesia (Federated States of) | Prevalence | -1.858734226 | -3.778589296 | 0.0994266998601834 |
| Latvia | Prevalence | -15.28571661 | -18.11775315 | -12.35572928 |
| Israel | Prevalence | -3.098017853 | -5.180638816 | -0.969654016 |
| Niue | Prevalence | -2.000662458 | -4.25354649 | 0.305231229511005 |
| Saint Vincent and the Grenadines | Prevalence | -2.461128002 | -5.79198131 | 0.98749217849845 |
| Austria | Prevalence | -2.347153512 | -4.384251094 | -0.266655479 |
| Peru | Prevalence | -2.174155298 | -3.425578646 | -0.906515852 |
| United States Virgin Islands | Prevalence | -3.423790227 | -6.539357528 | -0.204363597 |
| Mauritius | Prevalence | -6.197649512 | -7.903485799 | -4.460217268 |
| Mongolia | Prevalence | -3.252509631 | -5.528969036 | -0.921194601 |
| Oman | Prevalence | -5.272223015 | -6.595554393 | -3.930143001 |
| Madagascar | Prevalence | -1.883434682 | -2.708824392 | -1.051042609 |
| Andorra | Prevalence | -8.854061411 | -10.70470467 | -6.965063605 |
| Ireland | Prevalence | -7.283608068 | -8.625361314 | -5.922152398 |
| Gambia | Prevalence | -2.5918333 | -3.363929666 | -1.81356809 |
| Singapore | Prevalence | -4.715855442 | -7.800081998 | -1.528456849 |
| Kyrgyzstan | Prevalence | -3.267759489 | -4.114710031 | -2.41332787 |
| Malawi | Prevalence | -3.584155014 | -4.41767625 | -2.743365094 |
| Cambodia | Prevalence | -8.249631019 | -10.96086681 | -5.455838272 |
| Spain | Prevalence | -5.54102968 | -7.176124525 | -3.8771326 |
| Estonia | Prevalence | -7.08662671 | -9.224444418 | -4.898462137 |
| Venezuela (Bolivarian Republic of) | Prevalence | -2.973958563 | -4.399517512 | -1.527142207 |
| United Arab Emirates | Prevalence | -3.068179173 | -4.055644137 | -2.070551162 |
| Georgia | Prevalence | -5.251231029 | -7.81434612 | -2.616851499 |
| Bahrain | Prevalence | -1.495492399 | -2.606563791 | -0.371745825 |
| Barbados | Prevalence | -4.907110681 | -8.105049957 | -1.597883291 |
| Portugal | Prevalence | -6.53971569 | -8.380010197 | -4.662456718 |
| Northern Mariana Islands | Prevalence | -2.670965194 | -4.874462056 | -0.416426325 |
| Suriname | Prevalence | -2.266992202 | -5.53690481 | 1.11611094364386 |
| Serbia | Prevalence | -2.138266475 | -4.876961458 | 0.679278492532887 |
| Yemen | Prevalence | -2.261978399 | -3.290858446 | -1.222152188 |
| Uganda | Prevalence | -2.886758731 | -3.539138823 | -2.229966488 |
| Australia | Prevalence | -4.679048607 | -8.062793603 | -1.170765018 |
| Zambia | Prevalence | -2.581774822 | -3.106780305 | -2.053924654 |
| Qatar | Prevalence | -6.700197274 | -8.647777903 | -4.711095265 |
| Sao Tome and Principe | Prevalence | -4.831960398 | -5.529132241 | -4.129643597 |
| Belgium | Prevalence | -7.731908042 | -9.239764452 | -6.199000673 |
| Albania | Prevalence | -11.63115896 | -13.99308238 | -9.204372347 |
| Greenland | Prevalence | -3.317453216 | -5.019398661 | -1.585010824 |
| South Sudan | Prevalence | -1.788862088 | -2.597713224 | -0.973294064 |
| Senegal | Prevalence | -5.288844579 | -6.025118736 | -4.546801863 |
| Gabon | Prevalence | -0.952629075 | -1.851345879 | -0.045683001 |
| Egypt | Prevalence | -5.014619743 | -6.265793169 | -3.746745531 |
| Sierra Leone | Prevalence | -4.513969449 | -5.311347337 | -3.709876801 |
| Republic of Korea | Prevalence | -9.723465084 | -14.34225225 | -4.855626362 |
| Palestine | Prevalence | -7.782777845 | -9.030865894 | -6.517566145 |
| Lao People's Democratic Republic | Prevalence | -4.150533968 | -5.430768108 | -2.852968616 |
| Belize | Prevalence | -3.913632311 | -7.216443993 | -0.493250604 |
| Lithuania | Prevalence | -3.242254841 | -4.056012892 | -2.421594824 |
| United States of America | Prevalence | -0.30051707 | -1.684682243 | 1.10313553601289 |
| Republic of Moldova | Prevalence | -2.875590649 | -4.573196101 | -1.14778546 |
| Ghana | Prevalence | -4.608466829 | -5.292235253 | -3.919761753 |
| Kazakhstan | Prevalence | -6.832209276 | -8.617111727 | -5.012443877 |
| Switzerland | Prevalence | -5.188463738 | -6.967247525 | -3.375669653 |
| Burkina Faso | Prevalence | -6.940057307 | -7.703351714 | -6.170450445 |
| Angola | Prevalence | -1.04395663 | -1.309379391 | -0.77782003 |
| Costa Rica | Prevalence | -6.734482744 | -9.660706188 | -3.713474592 |
| Palau | Prevalence | -2.150468667 | -4.686210526 | 0.452734434583113 |
| Nigeria | Prevalence | -1.416001063 | -2.163890383 | -0.662394646 |
| Colombia | Prevalence | -3.463195423 | -4.956169074 | -1.946769745 |
| Monaco | Prevalence | -1.610290523 | -3.429558044 | 0.243249745137541 |
| Taiwan (Province of China) | Prevalence | -5.503205563 | -6.410214091 | -4.587406926 |
| Burundi | Prevalence | -7.744712948 | -8.817346936 | -6.659460948 |
| Canada | Prevalence | -2.13468457 | -4.103814957 | -0.125120099 |
| Paraguay | Prevalence | -5.717930128 | -7.387147957 | -4.018626971 |
| Guinea | Prevalence | -1.491491859 | -2.053564872 | -0.926193348 |
| Slovenia | Prevalence | -1.194607185 | -3.985307502 | 1.67720580310733 |
| Trinidad and Tobago | Prevalence | -3.775998457 | -7.015359661 | -0.423785701 |
| Indonesia | Prevalence | -3.276175863 | -5.325171673 | -1.182834751 |
| Cuba | Prevalence | -5.320940297 | -8.631221545 | -1.890727908 |
| China | Prevalence | -5.044118639 | -6.144264379 | -3.931077355 |
| Guam | Prevalence | -4.283967389 | -6.121731834 | -2.4102268 |
| Puerto Rico | Prevalence | -5.814395271 | -8.870576446 | -2.655719829 |
| Solomon Islands | Prevalence | -4.384911342 | -6.363352578 | -2.364667779 |
| Samoa | Prevalence | -3.163935626 | -5.477411447 | -0.793836616 |
| Brazil | Prevalence | -4.835275123 | -7.003908003 | -2.616070565 |
| Sweden | Prevalence | 1.67782456916892 | 0.506161211009082 | 2.8631467419558 |
| Denmark | Prevalence | -4.426016499 | -7.150114744 | -1.621996656 |
| Italy | Prevalence | -12.7780192 | -14.52893896 | -10.99123092 |
| Central African Republic | Prevalence | 0.317270312820139 | -0.132384191 | 0.76894938837031 |
| Benin | Prevalence | -2.453527895 | -3.121259731 | -1.781193751 |
| Cabo Verde | Prevalence | -7.60329224 | -9.491290683 | -5.675910425 |
| Belarus | Prevalence | -5.207530136 | -6.560642068 | -3.834823549 |
| El Salvador | Prevalence | -6.367084686 | -8.679680422 | -3.995924776 |
| Cameroon | Prevalence | -2.845667886 | -3.476279629 | -2.210936211 |
| Guinea-Bissau | Prevalence | -2.293142873 | -2.97639243 | -1.605081808 |
| Bolivia (Plurinational State of) | Prevalence | -7.937235609 | -9.861273906 | -5.972128134 |
| Sudan | Prevalence | -3.334352976 | -4.277992522 | -2.381410913 |
| Lesotho | Prevalence | -2.339992156 | -4.024394988 | -0.626027511 |
| Afghanistan | Prevalence | -3.235480816 | -4.404175447 | -2.052498457 |
| Netherlands | Prevalence | -0.555143711 | -2.327290478 | 1.24915639850089 |
| Iraq | Prevalence | 0.0613874576594231 | -0.8524741 | 0.983672251113421 |
| Kiribati | Prevalence | -2.15891951 | -4.070993763 | -0.208733447 |
| Croatia | Prevalence | -5.50354929 | -7.919751133 | -3.023945889 |
| Liberia | Prevalence | -4.064787278 | -5.217319579 | -2.89824049 |
| Bulgaria | Prevalence | -0.248804225 | -3.068954213 | 2.65339631764396 |
| Botswana | Prevalence | -0.349122268 | -2.011236244 | 1.34118497120899 |
| Slovakia | Prevalence | -1.930390324 | -4.124725495 | 0.314167460027859 |
| Mozambique | Prevalence | -6.251771221 | -7.343807397 | -5.146864422 |
| Malta | Prevalence | -6.824081185 | -9.139426646 | -4.449735165 |
| Malaysia | Prevalence | -2.239705207 | -3.128687104 | -1.34256518 |
| Marshall Islands | Prevalence | -3.430909171 | -5.453262242 | -1.365298004 |
| New Zealand | Prevalence | -6.875947677 | -9.88949642 | -3.761617386 |
| Uzbekistan | Prevalence | 0.0630129588956363 | -1.879913272 | 2.04441207015185 |
| Dominica | Prevalence | -4.170095281 | -7.269522394 | -0.967072796 |
| Maldives | Prevalence | -4.182232136 | -6.219725706 | -2.100471475 |
| Finland | Prevalence | -1.052407473 | -3.031042495 | 0.966601259 |
| Democratic People's Republic of Korea | Prevalence | -12.13707294 | -14.3488137 | -9.868219172 |
| Tonga | Prevalence | -9.343421056 | -11.24438044 | -7.401747109 |
| Turkey | Prevalence | -5.026152173 | -6.466132909 | -3.56400252 |
| Djibouti | Prevalence | 0.571591975799102 | -0.182182431 | 1.33105851156614 |
| United Kingdom | Prevalence | -4.247692832 | -6.042999169 | -2.418082241 |
| Cyprus | Prevalence | -4.255407636 | -6.449695496 | -2.009651218 |
| Tajikistan | Prevalence | -4.430141669 | -5.860356827 | -2.978197987 |
| Comoros | Prevalence | -2.362740696 | -3.221468668 | -1.496393124 |
| Jordan | Prevalence | 0.181853054508507 | -1.584496387 | 1.97990472001848 |
| Kuwait | Prevalence | -4.121298708 | -5.639993647 | -2.578160846 |
| Saint Kitts and Nevis | Prevalence | -1.79918241 | -5.051799412 | 1.56485868612792 |
| France | Prevalence | -4.975491959 | -6.519748621 | -3.405724791 |
| Bosnia and Herzegovina | Prevalence | -4.436037435 | -6.98438371 | -1.817874186 |
| Norway | Prevalence | -3.980261165 | -6.278390674 | -1.625779666 |
| Myanmar | Prevalence | -2.31137093 | -4.333524735 | -0.246473771 |
| Eritrea | Prevalence | -7.507123248 | -9.144806506 | -5.839920418 |
| Syrian Arab Republic | Prevalence | 2.36648002105373 | 0.841303655805103 | 3.91472394751766 |
| Tunisia | Prevalence | -3.78037714 | -5.113522736 | -2.428500979 |
| Somalia | Prevalence | -0.37368954 | -1.113039765 | 0.371188600723626 |
| Czechia | Prevalence | -2.794087605 | -5.241001938 | -0.283987824 |
| Ecuador | Prevalence | -2.221901647 | -3.337661499 | -1.093262735 |
| Saudi Arabia | Prevalence | -2.31923077 | -3.260096029 | -1.369214919 |
| Rwanda | Prevalence | -6.812122754 | -7.726261072 | -5.888928242 |
| Russian Federation | Prevalence | -7.939310228 | -9.268746081 | -6.590394873 |
| Vanuatu | Prevalence | -4.15970022 | -6.537436171 | -1.721473436 |
| Grenada | Prevalence | -5.874411338 | -8.809754417 | -2.844581848 |
| Guatemala | Prevalence | -3.292228992 | -4.575040756 | -1.992172201 |
| Bhutan | Prevalence | -13.36279883 | -14.44185585 | -12.27013276 |
| Iran (Islamic Republic of) | Prevalence | -3.757905253 | -5.425399944 | -2.061010083 |
| United Republic of Tanzania | Prevalence | -3.317280994 | -3.7851779 | -2.847108686 |
| San Marino | Prevalence | 2.90887787052347 | 0.756386665391529 | 5.10735343993753 |
| Namibia | Prevalence | -5.505882082 | -7.174396321 | -3.807376766 |
| Dominican Republic | Prevalence | -4.461319572 | -7.58598492 | -1.231004302 |
| Fiji | Prevalence | -4.841889231 | -7.543319774 | -2.061527376 |
| Mauritania | Prevalence | -1.948278885 | -2.664134271 | -1.22715875 |
| Tokelau | Prevalence | -6.02287234 | -7.624064406 | -4.393926119 |
| Nauru | Prevalence | -9.534777664 | -12.20111586 | -6.787466236 |
| Bangladesh | Prevalence | -6.104135755 | -6.916714565 | -5.284463467 |
| Ukraine | Prevalence | 3.49913995210951 | 0.173547468285351 | 6.93513648618425 |
| Togo | Prevalence | -1.677289566 | -2.381244156 | -0.968258574 |
| Luxembourg | Prevalence | -7.982716265 | -9.676675898 | -6.256987438 |
| Turkmenistan | Prevalence | -10.88732273 | -12.48511919 | -9.260354621 |
| Mali | Prevalence | -2.869944794 | -3.548793885 | -2.186317782 |
| Honduras | Prevalence | -1.324431381 | -2.276761985 | -0.362820143 |
| Coted'Ivoire | Prevalence | -2.545140118 | -3.450097667 | -1.631700445 |
| Guyana | Prevalence | -5.977105248 | -9.130934561 | -2.713814709 |
| Seychelles | Prevalence | -2.930797398 | -4.910043275 | -0.910354592 |
| Antigua and Barbuda | Prevalence | -2.423570281 | -5.489533225 | 0.74185390763184 |
| Chad | Prevalence | -1.540443121 | -2.165049196 | -0.911849383 |
| Hungary | Prevalence | -5.193201578 | -7.701193786 | -2.617060873 |
| Congo | Prevalence | 0.0959485521478953 | -0.755244951 | 0.954442495515262 |
| Bahamas | Prevalence | -4.918835032 | -7.819127694 | -1.927290277 |
| Democratic Republic of the Congo | Prevalence | -1.87814762 | -2.344997443 | -1.409065973 |

Sup_Table 9 EAPC of adolescent pertussis incidence in 204 countries and territories, 1990-2021

| location | measure | EAPC | LCI | UCI |
| --- | --- | --- | --- | --- |
| Mexico | Incidence | -1.750838299 | -3.320583374 | -0.155605899 |
| Ethiopia | Incidence | -2.468934819 | -3.160088841 | -1.772847976 |
| Argentina | Incidence | -4.549483988 | -6.152940605 | -2.918630954 |
| India | Incidence | -3.40146582 | -4.267676799 | -2.527417139 |
| Germany | Incidence | -8.163008487 | -10.61712932 | -5.641506633 |
| South Africa | Incidence | -0.865843652 | -2.492148392 | 0.787585746065278 |
| Haiti | Incidence | -4.552385307 | -7.641653492 | -1.359785065 |
| Lebanon | Incidence | 1.00423279907595 | -0.410693992 | 2.43926232863882 |
| Chile | Incidence | -4.575288372 | -7.089568081 | -1.992968914 |
| Eswatini | Incidence | -2.704357211 | -4.166662949 | -1.219738381 |
| Tuvalu | Incidence | -5.895629486 | -7.727269983 | -4.027630394 |
| North Macedonia | Incidence | -1.570022186 | -4.278367052 | 1.21495250393608 |
| Niger | Incidence | -3.249026826 | -3.780958392 | -2.71415456 |
| Andorra | Incidence | -8.854061411 | -10.70470467 | -6.965063605 |
| Philippines | Incidence | -2.220592136 | -3.537721641 | -0.885478087 |
| Nicaragua | Incidence | -16.71732733 | -18.84983019 | -14.52878555 |
| Kenya | Incidence | -2.070166896 | -2.808414678 | -1.326311532 |
| American Samoa | Incidence | -4.060878346 | -5.976833757 | -2.105880589 |
| Sri Lanka | Incidence | -0.151885684 | -3.09905416 | 2.88491867554324 |
| Brunei Darussalam | Incidence | -6.666321341 | -10.67925695 | -2.473095558 |
| Bermuda | Incidence | -6.254379894 | -9.32645128 | -3.078224982 |
| Libya | Incidence | -0.128255021 | -1.258025779 | 1.01444217531823 |
| Barbados | Incidence | -4.907110681 | -8.105049957 | -1.597883291 |
| Armenia | Incidence | -2.168253613 | -3.507278195 | -0.810647455 |
| Zimbabwe | Incidence | -2.21004666 | -4.576582329 | 0.215179960714607 |
| Uruguay | Incidence | -4.65518975 | -6.528659794 | -2.744169264 |
| Montenegro | Incidence | -2.083907585 | -4.627207153 | 0.527213973518048 |
| Japan | Incidence | -10.95254824 | -14.33717439 | -7.43419203 |
| Panama | Incidence | -0.158433994 | -1.549261247 | 1.25204166949799 |
| Cook Islands | Incidence | -6.32218503 | -8.293275921 | -4.30872866 |
| Nepal | Incidence | -5.27187349 | -6.168383847 | -4.366797461 |
| Saint Lucia | Incidence | -4.458576518 | -7.500509243 | -1.316606976 |
| Austria | Incidence | -2.347153512 | -4.384251094 | -0.266655479 |
| Madagascar | Incidence | -1.883434682 | -2.708824392 | -1.051042609 |
| United States Virgin Islands | Incidence | -3.423790227 | -6.539357528 | -0.204363597 |
| Azerbaijan | Incidence | -5.742314901 | -7.468442571 | -3.983987226 |
| Timor-Leste | Incidence | -5.107077103 | -6.907879475 | -3.27143946 |
| Senegal | Incidence | -5.288844579 | -6.025118736 | -4.546801863 |
| Cambodia | Incidence | -8.249631019 | -10.96086681 | -5.455838272 |
| Samoa | Incidence | -3.163935626 | -5.477411447 | -0.793836616 |
| Albania | Incidence | -11.63115896 | -13.99308238 | -9.204372347 |
| Jamaica | Incidence | -4.763675064 | -8.131587948 | -1.272293873 |
| Greece | Incidence | -16.35837244 | -19.32226624 | -13.28559275 |
| Micronesia (Federated States of) | Incidence | -1.858734226 | -3.778589296 | 0.0994266998599835 |
| Poland | Incidence | -5.426351246 | -7.507356302 | -3.298525364 |
| Estonia | Incidence | -7.08662671 | -9.224444418 | -4.898462137 |
| Palestine | Incidence | -7.782777845 | -9.030865894 | -6.517566145 |
| Belgium | Incidence | -7.731908042 | -9.239764452 | -6.199000673 |
| Morocco | Incidence | -3.831654445 | -5.05378329 | -2.5937946 |
| Georgia | Incidence | -5.251231029 | -7.81434612 | -2.616851499 |
| Bahrain | Incidence | -1.495492399 | -2.606563791 | -0.371745825 |
| Malawi | Incidence | -3.584155014 | -4.41767625 | -2.743365094 |
| Lithuania | Incidence | -3.242254841 | -4.056012892 | -2.421594824 |
| Thailand | Incidence | -2.984019493 | -5.751769293 | -0.134990299 |
| Ghana | Incidence | -4.608466829 | -5.292235253 | -3.919761753 |
| Peru | Incidence | -2.174155298 | -3.425578646 | -0.906515852 |
| Pakistan | Incidence | -3.051412816 | -3.782589035 | -2.314680237 |
| Niue | Incidence | -2.000662458 | -4.25354649 | 0.305231229511382 |
| South Sudan | Incidence | -1.788862088 | -2.597713224 | -0.973294064 |
| Venezuela (Bolivarian Republic of) | Incidence | -2.973958563 | -4.399517512 | -1.527142207 |
| Uganda | Incidence | -2.886758731 | -3.539138823 | -2.229966488 |
| Belize | Incidence | -3.913632311 | -7.216443993 | -0.493250604 |
| Egypt | Incidence | -5.014619743 | -6.265793169 | -3.746745531 |
| Northern Mariana Islands | Incidence | -2.670965194 | -4.874462056 | -0.416426325 |
| Saint Vincent and the Grenadines | Incidence | -2.461128002 | -5.79198131 | 0.987492178498828 |
| Nigeria | Incidence | -1.416001063 | -2.163890383 | -0.662394646 |
| Gabon | Incidence | -0.952629075 | -1.851345879 | -0.045683001 |
| Equatorial Guinea | Incidence | -1.11944407 | -1.500936607 | -0.736473991 |
| Sao Tome and Principe | Incidence | -4.831960398 | -5.529132241 | -4.129643597 |
| Sudan | Incidence | -3.334352976 | -4.277992522 | -2.381410913 |
| Mauritius | Incidence | -6.197649512 | -7.903485799 | -4.460217268 |
| Sweden | Incidence | 1.67782456916807 | 0.506161211008371 | 2.8631467419548 |
| Algeria | Incidence | -1.55277785 | -2.959237641 | -0.125933541 |
| Romania | Incidence | -0.558420263 | -3.61648065 | 2.59666639411236 |
| Iceland | Incidence | 5.37934821317863 | 4.47657323084316 | 6.28992401289856 |
| Republic of Korea | Incidence | -9.723465084 | -14.34225225 | -4.855626361 |
| Republic of Moldova | Incidence | -2.875590649 | -4.573196101 | -1.14778546 |
| Latvia | Incidence | -15.28571661 | -18.11775315 | -12.35572928 |
| Papua New Guinea | Incidence | -2.189425903 | -4.039482454 | -0.303701462 |
| Zambia | Incidence | -2.581774822 | -3.106780305 | -2.053924654 |
| Guinea | Incidence | -1.491491859 | -2.053564872 | -0.926193348 |
| United Arab Emirates | Incidence | -3.068179173 | -4.055644137 | -2.070551162 |
| Indonesia | Incidence | -3.276175863 | -5.325171673 | -1.182834751 |
| Oman | Incidence | -5.272223015 | -6.595554393 | -3.930143001 |
| Colombia | Incidence | -3.463195423 | -4.956169074 | -1.946769745 |
| Puerto Rico | Incidence | -5.814395271 | -8.870576446 | -2.655719829 |
| Sierra Leone | Incidence | -4.513969449 | -5.311347337 | -3.709876801 |
| Ireland | Incidence | -7.283608068 | -8.625361314 | -5.922152398 |
| Palau | Incidence | -2.150468667 | -4.686210526 | 0.452734434583713 |
| United States of America | Incidence | -0.30051707 | -1.684682243 | 1.10313553601304 |
| Singapore | Incidence | -4.715855442 | -7.800081998 | -1.528456849 |
| Portugal | Incidence | -6.53971569 | -8.380010197 | -4.662456718 |
| Botswana | Incidence | -0.349122268 | -2.011236244 | 1.34118497120965 |
| Angola | Incidence | -1.04395663 | -1.309379391 | -0.77782003 |
| Canada | Incidence | -2.13468457 | -4.103814957 | -0.125120099 |
| Costa Rica | Incidence | -6.734482744 | -9.660706188 | -3.713474592 |
| China | Incidence | -5.044118639 | -6.144264379 | -3.931077355 |
| Greenland | Incidence | -3.317453216 | -5.019398661 | -1.585010824 |
| Cuba | Incidence | -5.320940297 | -8.631221545 | -1.890727908 |
| Brazil | Incidence | -4.835275123 | -7.003908003 | -2.616070565 |
| Croatia | Incidence | -5.50354929 | -7.919751133 | -3.023945889 |
| Israel | Incidence | -3.098017853 | -5.180638816 | -0.969654016 |
| Yemen | Incidence | -2.261978399 | -3.290858446 | -1.222152188 |
| Solomon Islands | Incidence | -4.384911342 | -6.363352578 | -2.364667779 |
| Malaysia | Incidence | -2.239705207 | -3.128687104 | -1.34256518 |
| Gambia | Incidence | -2.5918333 | -3.363929666 | -1.81356809 |
| Suriname | Incidence | -2.266992202 | -5.53690481 | 1.11611094364417 |
| Switzerland | Incidence | -5.188463738 | -6.967247525 | -3.375669653 |
| Spain | Incidence | -5.54102968 | -7.176124525 | -3.8771326 |
| Finland | Incidence | -1.052407473 | -3.031042495 | 0.966601259375044 |
| Serbia | Incidence | -2.138266475 | -4.876961458 | 0.679278492533331 |
| Taiwan (Province of China) | Incidence | -5.503205563 | -6.410214091 | -4.587406926 |
| Kyrgyzstan | Incidence | -3.267759489 | -4.114710031 | -2.41332787 |
| Kazakhstan | Incidence | -6.832209276 | -8.617111727 | -5.012443877 |
| Cameroon | Incidence | -2.845667886 | -3.476279629 | -2.210936211 |
| Viet Nam | Incidence | -2.809097223 | -4.800255117 | -0.776293106 |
| Democratic People's Republic of Korea | Incidence | -12.13707294 | -14.3488137 | -9.868219172 |
| Afghanistan | Incidence | -3.235480816 | -4.404175447 | -2.052498457 |
| Qatar | Incidence | -6.700197274 | -8.647777903 | -4.711095265 |
| Cyprus | Incidence | -4.255407636 | -6.449695496 | -2.009651218 |
| Trinidad and Tobago | Incidence | -3.775998457 | -7.015359661 | -0.423785701 |
| Mozambique | Incidence | -6.251771221 | -7.343807397 | -5.146864422 |
| Bosnia and Herzegovina | Incidence | -4.436037435 | -6.98438371 | -1.817874186 |
| El Salvador | Incidence | -6.367084686 | -8.679680422 | -3.995924776 |
| Paraguay | Incidence | -5.717930128 | -7.387147957 | -4.018626971 |
| Djibouti | Incidence | 0.57159197579888 | -0.182182431 | 1.3310585115659 |
| Benin | Incidence | -2.453527895 | -3.121259731 | -1.781193751 |
| Lao People's Democratic Republic | Incidence | -4.150533968 | -5.430768108 | -2.852968616 |
| Grenada | Incidence | -5.874411338 | -8.809754417 | -2.844581848 |
| Bolivia (Plurinational State of) | Incidence | -7.937235609 | -9.861273906 | -5.972128134 |
| Australia | Incidence | -4.679048607 | -8.062793603 | -1.170765018 |
| Monaco | Incidence | -1.610290523 | -3.429558044 | 0.243249745137963 |
| Mongolia | Incidence | -3.252509631 | -5.528969036 | -0.921194601 |
| Slovenia | Incidence | -1.194607185 | -3.985307502 | 1.67720580310757 |
| Tonga | Incidence | -9.343421056 | -11.24438044 | -7.401747109 |
| Czechia | Incidence | -2.794087605 | -5.241001938 | -0.283987824 |
| Bulgaria | Incidence | -0.248804225 | -3.068954213 | 2.65339631764414 |
| Dominica | Incidence | -4.170095281 | -7.269522394 | -0.967072796 |
| Fiji | Incidence | -4.841889231 | -7.543319774 | -2.061527376 |
| Marshall Islands | Incidence | -3.430909171 | -5.453262242 | -1.365298004 |
| Lesotho | Incidence | -2.339992156 | -4.024394988 | -0.626027511 |
| Congo | Incidence | 0.0959485521478065 | -0.755244951 | 0.954442495515062 |
| Saint Kitts and Nevis | Incidence | -1.79918241 | -5.051799412 | 1.5648586861283 |
| Maldives | Incidence | -4.182232136 | -6.219725706 | -2.100471475 |
| Central African Republic | Incidence | 0.317270312820095 | -0.132384191 | 0.768949388370244 |
| France | Incidence | -4.975491959 | -6.519748621 | -3.405724791 |
| Malta | Incidence | -6.824081185 | -9.139426646 | -4.449735165 |
| Cabo Verde | Incidence | -7.60329224 | -9.491290683 | -5.675910425 |
| Kiribati | Incidence | -2.15891951 | -4.070993763 | -0.208733447 |
| Kuwait | Incidence | -4.121298708 | -5.639993647 | -2.578160846 |
| Mali | Incidence | -2.869944794 | -3.548793885 | -2.186317782 |
| Tokelau | Incidence | -6.02287234 | -7.624064406 | -4.393926119 |
| Eritrea | Incidence | -7.507123248 | -9.144806506 | -5.839920418 |
| Burkina Faso | Incidence | -6.940057307 | -7.703351714 | -6.170450445 |
| Somalia | Incidence | -0.37368954 | -1.113039765 | 0.371188600723626 |
| Slovakia | Incidence | -1.930390324 | -4.124725495 | 0.314167460027814 |
| Guam | Incidence | -4.283967389 | -6.121731834 | -2.4102268 |
| Tunisia | Incidence | -3.78037714 | -5.113522736 | -2.428500979 |
| Russian Federation | Incidence | -7.939310228 | -9.268746081 | -6.590394874 |
| Iran (Islamic Republic of) | Incidence | -3.757905253 | -5.425399944 | -2.061010083 |
| Rwanda | Incidence | -6.812122754 | -7.726261072 | -5.888928242 |
| Comoros | Incidence | -2.362740696 | -3.221468668 | -1.496393124 |
| Myanmar | Incidence | -2.31137093 | -4.333524735 | -0.246473771 |
| Mauritania | Incidence | -1.948278885 | -2.664134271 | -1.22715875 |
| Guinea-Bissau | Incidence | -2.293142873 | -2.97639243 | -1.605081808 |
| Burundi | Incidence | -7.744712948 | -8.817346936 | -6.659460948 |
| Iraq | Incidence | 0.0613874576594231 | -0.8524741 | 0.983672251113688 |
| Vanuatu | Incidence | -4.15970022 | -6.537436171 | -1.721473436 |
| Togo | Incidence | -1.677289566 | -2.381244156 | -0.968258574 |
| New Zealand | Incidence | -6.875947677 | -9.88949642 | -3.761617386 |
| Democratic Republic of the Congo | Incidence | -1.87814762 | -2.344997443 | -1.409065973 |
| Luxembourg | Incidence | -7.982716265 | -9.676675898 | -6.256987438 |
| Uzbekistan | Incidence | 0.0630129588961692 | -1.879913272 | 2.04441207015233 |
| San Marino | Incidence | 2.90887787052412 | 0.756386665392239 | 5.10735343993813 |
| Ecuador | Incidence | -2.221901647 | -3.337661499 | -1.093262735 |
| Guyana | Incidence | -5.977105248 | -9.130934561 | -2.713814709 |
| United Republic of Tanzania | Incidence | -3.317280994 | -3.7851779 | -2.847108686 |
| Tajikistan | Incidence | -4.430141669 | -5.860356827 | -2.978197987 |
| Italy | Incidence | -12.7780192 | -14.52893896 | -10.99123092 |
| Antigua and Barbuda | Incidence | -2.423570281 | -5.489533225 | 0.741853907631707 |
| Seychelles | Incidence | -2.930797398 | -4.910043275 | -0.910354592 |
| Turkey | Incidence | -5.026152173 | -6.466132909 | -3.56400252 |
| Saudi Arabia | Incidence | -2.31923077 | -3.260096029 | -1.369214919 |
| Honduras | Incidence | -1.324431381 | -2.276761985 | -0.362820143 |
| Ukraine | Incidence | 3.49913995211037 | 0.173547468285706 | 6.93513648618569 |
| Syrian Arab Republic | Incidence | 2.36648002105364 | 0.841303655804904 | 3.91472394751771 |
| Bangladesh | Incidence | -6.104135755 | -6.916714565 | -5.284463467 |
| Guatemala | Incidence | -3.292228992 | -4.575040756 | -1.992172201 |
| Liberia | Incidence | -4.064787278 | -5.217319579 | -2.89824049 |
| United Kingdom | Incidence | -4.247692832 | -6.042999169 | -2.418082241 |
| Dominican Republic | Incidence | -4.461319572 | -7.58598492 | -1.231004302 |
| Netherlands | Incidence | -0.555143711 | -2.327290478 | 1.24915639850105 |
| Denmark | Incidence | -4.426016499 | -7.150114744 | -1.621996656 |
| Jordan | Incidence | 0.181853054508396 | -1.584496387 | 1.9799047200187 |
| Bhutan | Incidence | -13.36279883 | -14.44185585 | -12.27013276 |
| Chad | Incidence | -1.540443121 | -2.165049196 | -0.911849383 |
| Namibia | Incidence | -5.505882082 | -7.174396321 | -3.807376766 |
| Hungary | Incidence | -5.193201578 | -7.701193786 | -2.617060873 |
| Bahamas | Incidence | -4.918835032 | -7.819127694 | -1.927290277 |
| Turkmenistan | Incidence | -10.88732273 | -12.48511919 | -9.260354621 |
| Nauru | Incidence | -9.534777664 | -12.20111586 | -6.787466236 |
| Belarus | Incidence | -5.207530136 | -6.560642068 | -3.834823549 |
| Norway | Incidence | -3.980261165 | -6.278390674 | -1.625779666 |
| Coted'Ivoire | Incidence | -2.545140118 | -3.450097667 | -1.631700445 |

Sup_Table 10 EAPC of adolescent pertussis deaths in 204 countries and territories, 1990-2021

| location | measure | EAPC | LCI | UCI |
| --- | --- | --- | --- | --- |
| Mexico | Deaths | -0.983084415 | -3.287291645 | 1.37602119404088 |
| Ethiopia | Deaths | -4.790441108 | -5.724625598 | -3.846999686 |
| Argentina | Deaths | -3.530072434 | -6.131289375 | -0.856772587 |
| India | Deaths | -4.995763162 | -5.819629636 | -4.16468971 |
| Germany | Deaths | -7.317694586 | -12.63819808 | -1.673162086 |
| South Africa | Deaths | -1.360390094 | -2.711519412 | 0.00950352571040813 |
| Haiti | Deaths | -6.347147498 | -10.17163549 | -2.359830002 |
| Lebanon | Deaths | -2.551119269 | -4.202913671 | -0.870843575 |
| Chile | Deaths | 0.737815482084736 | -5.301393061 | 7.16216210686433 |
| Eswatini | Deaths | -2.979955553 | -4.706035066 | -1.2226112 |
| Tuvalu | Deaths | -5.090417125 | -5.837097504 | -4.337815821 |
| North Macedonia | Deaths | -3.319249158 | -5.357835305 | -1.236751996 |
| Niger | Deaths | -4.458921076 | -5.167967331 | -3.744573378 |
| Andorra | Deaths | -10.29616035 | -11.69618187 | -8.873942052 |
| Philippines | Deaths | -2.749848438 | -4.139318601 | -1.340238347 |
| Nicaragua | Deaths | -18.7413982 | -20.80625195 | -16.6227066 |
| Kenya | Deaths | -1.83471774 | -2.799520648 | -0.860338289 |
| American Samoa | Deaths | -2.329646771 | -2.584224181 | -2.074404072 |
| Sri Lanka | Deaths | -2.56504949 | -5.018453568 | -0.04827319 |
| Brunei Darussalam | Deaths | -6.891263748 | -9.250921939 | -4.470249707 |
| Bermuda | Deaths | -14.20042059 | -21.01383648 | -6.799273456 |
| Libya | Deaths | -2.287574729 | -3.176700017 | -1.390284631 |
| Barbados | Deaths | -4.63326409 | -7.264300672 | -1.927581459 |
| Armenia | Deaths | -0.289269184 | -4.006294603 | 3.57168523545404 |
| Zimbabwe | Deaths | -0.237828314 | -2.964613609 | 2.56558220429166 |
| Uruguay | Deaths | 7.51310584530633 | 2.05131028900567 | 13.2672172044551 |
| Montenegro | Deaths | -3.685939936 | -5.478164302 | -1.859733282 |
| Japan | Deaths | -7.661411571 | -10.2073591 | -5.043277184 |
| Panama | Deaths | -13.43831374 | -16.217173 | -10.56728692 |
| Cook Islands | Deaths | -6.376961309 | -6.918921099 | -5.831845986 |
| Nepal | Deaths | -6.842973173 | -7.79136931 | -5.884822469 |
| Saint Lucia | Deaths | -12.89112811 | -17.32568104 | -8.218710986 |
| Austria | Deaths | -1.438544194 | -4.005678206 | 1.1972415549065 |
| Madagascar | Deaths | -3.080994363 | -4.119841142 | -2.030891839 |
| United States Virgin Islands | Deaths | -3.213825257 | -4.182197588 | -2.235666175 |
| Azerbaijan | Deaths | -6.83613887 | -7.841043201 | -5.820277029 |
| Timor-Leste | Deaths | -8.987931749 | -13.81627416 | -3.889087103 |
| Senegal | Deaths | -5.878430106 | -6.664160405 | -5.086085284 |
| Cambodia | Deaths | -9.353778712 | -11.39876295 | -7.261594681 |
| Samoa | Deaths | -1.764400545 | -2.075866684 | -1.451943729 |
| Albania | Deaths | -13.85917398 | -15.09525293 | -12.60509967 |
| Jamaica | Deaths | -3.910098278 | -6.673669482 | -1.064692445 |
| Greece | Deaths | 3.32681111370703 | 0.292791335109621 | 6.45261491680289 |
| Micronesia (Federated States of) | Deaths | -1.504923063 | -1.839321685 | -1.169385263 |
| Poland | Deaths | -9.94704579 | -11.54384592 | -8.321420415 |
| Estonia | Deaths | -3.108237564 | -4.792293635 | -1.394393517 |
| Palestine | Deaths | -10.11685845 | -11.41357284 | -8.801162954 |
| Belgium | Deaths | 7.38190281081332 | 4.16188976440068 | 10.7014578686326 |
| Morocco | Deaths | -5.47147225 | -6.548881935 | -4.381640974 |
| Georgia | Deaths | -9.278977337 | -10.78915237 | -7.74323783 |
| Bahrain | Deaths | -5.442589092 | -6.016077791 | -4.865600971 |
| Malawi | Deaths | -4.210650249 | -5.213062491 | -3.197637071 |
| Lithuania | Deaths | -3.256757414 | -6.269930314 | -0.146719005 |
| Thailand | Deaths | -5.250965907 | -7.125473357 | -3.338624852 |
| Ghana | Deaths | -5.428283575 | -6.226340562 | -4.623434757 |
| Peru | Deaths | -5.560091312 | -6.731577299 | -4.373891026 |
| Pakistan | Deaths | -4.081118834 | -5.398672214 | -2.745215321 |
| Niue | Deaths | -1.655084583 | -2.369597152 | -0.935342821 |
| South Sudan | Deaths | -1.588470397 | -2.408068856 | -0.761988771 |
| Venezuela (Bolivarian Republic of) | Deaths | -3.018296218 | -4.697385644 | -1.309623749 |
| Uganda | Deaths | -2.546739829 | -3.447641354 | -1.637432258 |
| Belize | Deaths | -10.62696723 | -13.50551967 | -7.652616028 |
| Egypt | Deaths | -7.30911151 | -8.44438909 | -6.159756637 |
| Northern Mariana Islands | Deaths | -3.879703066 | -5.875660792 | -1.841419972 |
| Saint Vincent and the Grenadines | Deaths | -4.559684006 | -6.632037997 | -2.441332962 |
| Nigeria | Deaths | -2.638045073 | -3.580547798 | -1.686329357 |
| Gabon | Deaths | -2.849973839 | -3.816147446 | -1.874094949 |
| Equatorial Guinea | Deaths | -5.304894537 | -5.617427691 | -4.991326478 |
| Sao Tome and Principe | Deaths | -6.063158128 | -6.74506954 | -5.37626035 |
| Sudan | Deaths | -5.641257892 | -6.943557231 | -4.320733232 |
| Mauritius | Deaths | -15.87436328 | -18.11080944 | -13.57683835 |
| Sweden | Deaths | -4.888889957 | -8.005893203 | -1.66627441 |
| Algeria | Deaths | -4.296413099 | -5.445016578 | -3.133856999 |
| Romania | Deaths | -12.12407894 | -14.06061624 | -10.14390417 |
| Iceland | Deaths | -4.356722241 | -4.825416199 | -3.885720167 |
| Republic of Korea | Deaths | -11.38847763 | -13.1446392 | -9.596807555 |
| Republic of Moldova | Deaths | -4.826617554 | -6.288840543 | -3.341578755 |
| Latvia | Deaths | -2.62774543 | -5.514750624 | 0.347472463920173 |
| Papua New Guinea | Deaths | -6.433992159 | -12.61023774 | 0.178757750410274 |
| Zambia | Deaths | -3.777277707 | -4.49174318 | -3.057467554 |
| Guinea | Deaths | -2.072080155 | -2.863844135 | -1.273862449 |
| United Arab Emirates | Deaths | -6.845087289 | -9.062398457 | -4.57371192 |
| Indonesia | Deaths | -4.521577504 | -6.061703247 | -2.956201279 |
| Oman | Deaths | -7.271669964 | -8.438321242 | -6.090153568 |
| Colombia | Deaths | -3.849378226 | -5.569082243 | -2.098356267 |
| Puerto Rico | Deaths | -0.242911073 | -4.539750903 | 4.24733735098775 |
| Sierra Leone | Deaths | -4.627752015 | -5.645204525 | -3.599328046 |
| Ireland | Deaths | -12.34388939 | -15.46089259 | -9.111960588 |
| Palau | Deaths | -1.339932202 | -2.40481825 | -0.26342691 |
| United States of America | Deaths | 0.463974666055278 | -1.170419682 | 2.12539781319891 |
| Singapore | Deaths | -5.356585212 | -9.180286109 | -1.371898469 |
| Portugal | Deaths | -0.333337271 | -5.312947899 | 4.90815205525523 |
| Botswana | Deaths | -2.14971642 | -4.468799405 | 0.225663835698708 |
| Angola | Deaths | -2.156089159 | -2.555725918 | -1.754813417 |
| Canada | Deaths | -1.037172126 | -4.244904649 | 2.27801731959603 |
| Costa Rica | Deaths | -6.819030674 | -10.63351917 | -2.841725845 |
| China | Deaths | -9.034958059 | -9.682325253 | -8.382950751 |
| Greenland | Deaths | -5.108215923 | -6.050577379 | -4.156402093 |
| Cuba | Deaths | -6.633995512 | -8.40297796 | -4.830849302 |
| Brazil | Deaths | -2.121779798 | -4.38931383 | 0.199531805606679 |
| Croatia | Deaths | 2.24129068704164 | -0.089405863 | 4.62635731111256 |
| Israel | Deaths | 1.23200930791754 | -3.322573851 | 6.00116404305702 |
| Yemen | Deaths | -4.024775632 | -5.237581628 | -2.796447676 |
| Solomon Islands | Deaths | -2.339016023 | -2.579550935 | -2.097887222 |
| Malaysia | Deaths | -4.680256135 | -5.532818407 | -3.819999526 |
| Gambia | Deaths | -3.427944444 | -4.320727928 | -2.526830397 |
| Suriname | Deaths | -2.350740586 | -4.623472722 | -0.0238514 |
| Switzerland | Deaths | -2.70277974 | -5.375735691 | 0.0456821458674872 |
| Spain | Deaths | 15.5445229439669 | 11.206840636251 | 20.0513988704837 |
| Finland | Deaths | -6.075261676 | -8.22702447 | -3.873047394 |
| Serbia | Deaths | -5.048852643 | -6.468700639 | -3.607450704 |
| Taiwan (Province of China) | Deaths | 5.54296549158353 | 3.18448416883734 | 7.95535447491018 |
| Kyrgyzstan | Deaths | -12.29777134 | -13.86624723 | -10.70073387 |
| Kazakhstan | Deaths | -7.237250162 | -8.614821885 | -5.838912447 |
| Cameroon | Deaths | -3.8706806 | -4.693330926 | -3.040929475 |
| Viet Nam | Deaths | -4.771855892 | -6.356674429 | -3.160215905 |
| Democratic People's Republic of Korea | Deaths | -14.34606248 | -16.59482744 | -12.03666646 |
| Afghanistan | Deaths | -4.113830629 | -5.425252041 | -2.784224382 |
| Qatar | Deaths | -9.889141955 | -11.41244607 | -8.339643916 |
| Cyprus | Deaths | -8.202510643 | -10.40722386 | -5.943543494 |
| Trinidad and Tobago | Deaths | 0.181079218024949 | -3.141470631 | 3.61760289656428 |
| Mozambique | Deaths | -6.111696572 | -7.382640038 | -4.823312559 |
| Bosnia and Herzegovina | Deaths | -5.965179449 | -7.479512458 | -4.42606053 |
| El Salvador | Deaths | -9.778215983 | -12.64788983 | -6.814268188 |
| Paraguay | Deaths | -6.838131253 | -7.872576487 | -5.792070835 |
| Djibouti | Deaths | -0.636592536 | -1.614630208 | 0.351167695680754 |
| Benin | Deaths | -3.660359711 | -4.562636955 | -2.749552222 |
| Lao People's Democratic Republic | Deaths | -6.080763618 | -8.120038137 | -3.996227429 |
| Grenada | Deaths | -2.089385944 | -3.785077124 | -0.363809914 |
| Bolivia (Plurinational State of) | Deaths | -10.38350378 | -12.58060363 | -8.131184511 |
| Australia | Deaths | -3.841499072 | -7.198195957 | -0.363388448 |
| Monaco | Deaths | -3.246283858 | -4.630283519 | -1.842199677 |
| Mongolia | Deaths | -5.804892704 | -7.538449365 | -4.038833681 |
| Slovenia | Deaths | 4.99517775825222 | -0.786681161 | 11.1139863220035 |
| Tonga | Deaths | -7.281144801 | -7.812666844 | -6.746558175 |
| Czechia | Deaths | 8.56193472688713 | 5.58337689739008 | 11.6245191049221 |
| Bulgaria | Deaths | -0.742459832 | -2.896154948 | 1.45900273012514 |
| Dominica | Deaths | -4.168349509 | -7.398715202 | -0.825293561 |
| Fiji | Deaths | -5.328782976 | -7.894413379 | -2.691686123 |
| Marshall Islands | Deaths | -1.569408615 | -2.462997172 | -0.667633416 |
| Lesotho | Deaths | -2.822627417 | -5.496623835 | -0.072969609 |
| Congo | Deaths | -0.783827667 | -1.737736762 | 0.17934177293133 |
| Saint Kitts and Nevis | Deaths | -15.90828596 | -20.17546192 | -11.41299981 |
| Maldives | Deaths | -7.354144908 | -9.090192927 | -5.58494466 |
| Central African Republic | Deaths | 0.046027993686959 | -0.398793649 | 0.492836221227111 |
| France | Deaths | -3.749846736 | -5.792940818 | -1.662443517 |
| Malta | Deaths | -11.35548305 | -14.1025401 | -8.52057332 |
| Cabo Verde | Deaths | -8.961524838 | -10.79539838 | -7.089950413 |
| Kiribati | Deaths | -0.237470612 | -0.643025227 | 0.169739393635715 |
| Kuwait | Deaths | -6.716526082 | -8.084267989 | -5.328431642 |
| Mali | Deaths | -3.74692916 | -4.583727181 | -2.902792444 |
| Tokelau | Deaths | -6.162057195 | -6.470597171 | -5.852499389 |
| Eritrea | Deaths | -7.55566311 | -8.848061045 | -6.244940908 |
| Burkina Faso | Deaths | -7.235587292 | -8.108745237 | -6.354132531 |
| Somalia | Deaths | 0.31444638608642 | -0.498231075 | 1.13376136419718 |
| Slovakia | Deaths | -3.717159249 | -5.196170854 | -2.215073941 |
| Guam | Deaths | -3.815489179 | -4.970035146 | -2.646916307 |
| Tunisia | Deaths | -8.590689603 | -11.60185846 | -5.47694915 |
| Russian Federation | Deaths | -8.447365395 | -9.597654227 | -7.282440169 |
| Iran (Islamic Republic of) | Deaths | -6.644426951 | -8.121507563 | -5.143600117 |
| Rwanda | Deaths | -9.057467572 | -10.09417174 | -8.008809167 |
| Comoros | Deaths | -3.833677046 | -4.906144684 | -2.749114128 |
| Myanmar | Deaths | -5.467280898 | -7.768575402 | -3.108566092 |
| Mauritania | Deaths | -4.428534335 | -5.106173882 | -3.746055744 |
| Guinea-Bissau | Deaths | -3.501466065 | -4.252558472 | -2.744481701 |
| Burundi | Deaths | -8.440860399 | -9.600537023 | -7.26630703 |
| Iraq | Deaths | -5.019788476 | -6.967650072 | -3.031143596 |
| Vanuatu | Deaths | -4.767507004 | -8.162457246 | -1.247055939 |
| Togo | Deaths | -2.743883964 | -3.570909718 | -1.90976521 |
| New Zealand | Deaths | -4.344251227 | -8.255175925 | -0.266610509 |
| Democratic Republic of the Congo | Deaths | -2.307695661 | -2.745847079 | -1.867570275 |
| Luxembourg | Deaths | -10.48688574 | -11.66542215 | -9.292625621 |
| Uzbekistan | Deaths | -13.01814113 | -14.46691807 | -11.54482451 |
| San Marino | Deaths | 1.60447380343571 | -0.237725035 | 3.48069047619353 |
| Ecuador | Deaths | -7.015019062 | -8.278488372 | -5.734145387 |
| Guyana | Deaths | 0.941292875691779 | -1.900038207 | 3.8649192230743 |
| United Republic of Tanzania | Deaths | -3.602063767 | -4.313014467 | -2.885830729 |
| Tajikistan | Deaths | -5.225337586 | -6.442781059 | -3.992051738 |
| Italy | Deaths | -10.34914051 | -12.26061396 | -8.396024058 |
| Antigua and Barbuda | Deaths | -3.661747438 | -5.505142787 | -1.782391333 |
| Seychelles | Deaths | -4.357303513 | -6.22489299 | -2.452519832 |
| Turkey | Deaths | -10.02329477 | -11.32824575 | -8.699139295 |
| Saudi Arabia | Deaths | -6.507299098 | -7.209822675 | -5.799456645 |
| Honduras | Deaths | -3.652642019 | -4.645760875 | -2.649179784 |
| Ukraine | Deaths | -7.040134494 | -9.562416334 | -4.447506839 |
| Syrian Arab Republic | Deaths | -0.300032677 | -1.542001799 | 0.957602894792697 |
| Bangladesh | Deaths | -7.948334903 | -8.574434051 | -7.317948113 |
| Guatemala | Deaths | -8.717034253 | -10.48756475 | -6.911483162 |
| Liberia | Deaths | -5.813681122 | -6.97425185 | -4.638631346 |
| United Kingdom | Deaths | -2.875894738 | -6.660643274 | 1.06231876630796 |
| Dominican Republic | Deaths | -4.564604038 | -6.299337545 | -2.797754425 |
| Netherlands | Deaths | 10.9991355398725 | 6.3165561537964 | 15.8879532626682 |
| Denmark | Deaths | -6.04897027 | -10.98210371 | -0.84245578 |
| Jordan | Deaths | -3.132747584 | -4.903566688 | -1.328953528 |
| Bhutan | Deaths | -13.58118066 | -14.7216986 | -12.42540936 |
| Chad | Deaths | -1.425309726 | -2.327066281 | -0.515227785 |
| Namibia | Deaths | -6.187751386 | -7.896326887 | -4.447480838 |
| Hungary | Deaths | -6.977926846 | -8.922133083 | -4.992218342 |
| Bahamas | Deaths | -4.368329751 | -6.264434528 | -2.433870127 |
| Turkmenistan | Deaths | -6.7664562 | -9.013250778 | -4.464180071 |
| Nauru | Deaths | -8.602942805 | -10.24080833 | -6.935190609 |
| Belarus | Deaths | -6.464966317 | -8.014436445 | -4.889395814 |
| Norway | Deaths | -2.914380373 | -4.727440846 | -1.066816909 |
| Coted'Ivoire | Deaths | -3.753216635 | -4.97733179 | -2.513332 |

Sup_Table 11 EAPC of adolescent pertussis DALYs in 204 countries and territories, 1990-2021

| location | measure | EAPC | LCI | UCI |
| --- | --- | --- | --- | --- |
| Mexico | DALYs (Disability-Adjusted Life Years) | -1.479446695 | -3.196577647 | 0.268143290661182 |
| Ethiopia | DALYs (Disability-Adjusted Life Years) | -4.780006852 | -5.711676389 | -3.839131423 |
| Argentina | DALYs (Disability-Adjusted Life Years) | -4.255794818 | -5.926397909 | -2.555524374 |
| India | DALYs (Disability-Adjusted Life Years) | -4.984336782 | -5.808563444 | -4.152897686 |
| Germany | DALYs (Disability-Adjusted Life Years) | -8.137551759 | -10.58747394 | -5.620501196 |
| South Africa | DALYs (Disability-Adjusted Life Years) | -1.347176851 | -2.697512677 | 0.0218985455900267 |
| Haiti | DALYs (Disability-Adjusted Life Years) | -6.360697904 | -10.20382581 | -2.353090471 |
| Lebanon | DALYs (Disability-Adjusted Life Years) | -2.431550098 | -4.06516837 | -0.770113893 |
| Chile | DALYs (Disability-Adjusted Life Years) | -3.444651597 | -6.146073175 | -0.665474309 |
| Eswatini | DALYs (Disability-Adjusted Life Years) | -2.971817368 | -4.690881689 | -1.221746757 |
| Tuvalu | DALYs (Disability-Adjusted Life Years) | -5.08866896 | -5.83223607 | -4.339230499 |
| North Macedonia | DALYs (Disability-Adjusted Life Years) | -3.281193362 | -5.337596005 | -1.180118372 |
| Niger | DALYs (Disability-Adjusted Life Years) | -4.450304854 | -5.158054453 | -3.737273738 |
| Andorra | DALYs (Disability-Adjusted Life Years) | -10.17844011 | -11.60847117 | -8.725273476 |
| Philippines | DALYs (Disability-Adjusted Life Years) | -2.746033887 | -4.134400629 | -1.337560223 |
| Nicaragua | DALYs (Disability-Adjusted Life Years) | -18.71820241 | -20.78719157 | -16.59517255 |
| Kenya | DALYs (Disability-Adjusted Life Years) | -1.834654391 | -2.797391736 | -0.862381672 |
| American Samoa | DALYs (Disability-Adjusted Life Years) | -2.342666131 | -2.592118286 | -2.092575154 |
| Sri Lanka | DALYs (Disability-Adjusted Life Years) | -2.511585748 | -4.979265994 | 0.0201799397369751 |
| Brunei Darussalam | DALYs (Disability-Adjusted Life Years) | -6.87848483 | -9.253214368 | -4.4416116 |
| Bermuda | DALYs (Disability-Adjusted Life Years) | -8.112925598 | -11.30521294 | -4.805742007 |
| Libya | DALYs (Disability-Adjusted Life Years) | -2.229191742 | -3.124462641 | -1.325647238 |
| Barbados | DALYs (Disability-Adjusted Life Years) | -4.835922671 | -7.96115981 | -1.604566125 |
| Armenia | DALYs (Disability-Adjusted Life Years) | -2.101240322 | -3.438592113 | -0.745366536 |
| Zimbabwe | DALYs (Disability-Adjusted Life Years) | -0.246324579 | -2.963119166 | 2.5465335918988 |
| Uruguay | DALYs (Disability-Adjusted Life Years) | -4.563813457 | -6.445768334 | -2.644000813 |
| Montenegro | DALYs (Disability-Adjusted Life Years) | -3.617895543 | -5.430743259 | -1.770296398 |
| Japan | DALYs (Disability-Adjusted Life Years) | -10.51242749 | -13.55916988 | -7.358297894 |
| Panama | DALYs (Disability-Adjusted Life Years) | -3.556872449 | -5.082917621 | -2.006292032 |
| Cook Islands | DALYs (Disability-Adjusted Life Years) | -6.360629621 | -6.906472325 | -5.811586434 |
| Nepal | DALYs (Disability-Adjusted Life Years) | -6.833261672 | -7.780609607 | -5.876181857 |
| Saint Lucia | DALYs (Disability-Adjusted Life Years) | -4.706065311 | -7.736290446 | -1.576318226 |
| Austria | DALYs (Disability-Adjusted Life Years) | -2.332365744 | -4.357870692 | -0.263964738 |
| Madagascar | DALYs (Disability-Adjusted Life Years) | -3.070774147 | -4.10616186 | -2.024207115 |
| United States Virgin Islands | DALYs (Disability-Adjusted Life Years) | -3.551354775 | -4.859366681 | -2.225360067 |
| Azerbaijan | DALYs (Disability-Adjusted Life Years) | -6.813230964 | -7.824556045 | -5.790809887 |
| Timor-Leste | DALYs (Disability-Adjusted Life Years) | -8.245105394 | -12.31920934 | -3.981697467 |
| Senegal | DALYs (Disability-Adjusted Life Years) | -5.877581321 | -6.662475136 | -5.086087177 |
| Cambodia | DALYs (Disability-Adjusted Life Years) | -9.354834436 | -11.4094023 | -7.252617624 |
| Samoa | DALYs (Disability-Adjusted Life Years) | -1.76074146 | -2.072084953 | -1.448408108 |
| Albania | DALYs (Disability-Adjusted Life Years) | -13.78216367 | -15.03834395 | -12.50741044 |
| Jamaica | DALYs (Disability-Adjusted Life Years) | -4.771127001 | -8.150367736 | -1.267560586 |
| Greece | DALYs (Disability-Adjusted Life Years) | -15.54965614 | -18.06335168 | -12.95884413 |
| Micronesia (Federated States of) | DALYs (Disability-Adjusted Life Years) | -1.502133328 | -1.834086096 | -1.169058046 |
| Poland | DALYs (Disability-Adjusted Life Years) | -5.475543871 | -7.543060582 | -3.361793472 |
| Estonia | DALYs (Disability-Adjusted Life Years) | -7.10470529 | -9.241642634 | -4.917453006 |
| Palestine | DALYs (Disability-Adjusted Life Years) | -10.06950947 | -11.36504508 | -8.755037614 |
| Belgium | DALYs (Disability-Adjusted Life Years) | -7.567315549 | -9.064996361 | -6.044968243 |
| Morocco | DALYs (Disability-Adjusted Life Years) | -5.453258113 | -6.532038692 | -4.362026555 |
| Georgia | DALYs (Disability-Adjusted Life Years) | -5.355112733 | -7.690901422 | -2.960219266 |
| Bahrain | DALYs (Disability-Adjusted Life Years) | -5.319057943 | -5.906995336 | -4.727446841 |
| Malawi | DALYs (Disability-Adjusted Life Years) | -4.205381751 | -5.206588244 | -3.193600531 |
| Lithuania | DALYs (Disability-Adjusted Life Years) | -3.20831057 | -4.021304805 | -2.388429811 |
| Thailand | DALYs (Disability-Adjusted Life Years) | -5.189490843 | -7.082127486 | -3.258303237 |
| Ghana | DALYs (Disability-Adjusted Life Years) | -5.41843257 | -6.215145976 | -4.614950988 |
| Peru | DALYs (Disability-Adjusted Life Years) | -5.497953987 | -6.669743693 | -4.311452106 |
| Pakistan | DALYs (Disability-Adjusted Life Years) | -4.071612245 | -5.384093447 | -2.740924729 |
| Niue | DALYs (Disability-Adjusted Life Years) | -1.646231351 | -2.362820824 | -0.924382607 |
| South Sudan | DALYs (Disability-Adjusted Life Years) | -1.585965876 | -2.405687294 | -0.759359394 |
| Venezuela (Bolivarian Republic of) | DALYs (Disability-Adjusted Life Years) | -2.919034851 | -4.350992961 | -1.465638945 |
| Uganda | DALYs (Disability-Adjusted Life Years) | -2.547455021 | -3.446405455 | -1.640135018 |
| Belize | DALYs (Disability-Adjusted Life Years) | -4.051598173 | -7.340042899 | -0.646448571 |
| Egypt | DALYs (Disability-Adjusted Life Years) | -7.274864755 | -8.412872578 | -6.122716715 |
| Northern Mariana Islands | DALYs (Disability-Adjusted Life Years) | -3.803419017 | -5.766121132 | -1.799837766 |
| Saint Vincent and the Grenadines | DALYs (Disability-Adjusted Life Years) | -2.482480297 | -5.794947502 | 0.946460903391566 |
| Nigeria | DALYs (Disability-Adjusted Life Years) | -2.62202071 | -3.562853528 | -1.672009205 |
| Gabon | DALYs (Disability-Adjusted Life Years) | -2.830159744 | -3.794915743 | -1.855729057 |
| Equatorial Guinea | DALYs (Disability-Adjusted Life Years) | -5.269997031 | -5.58346709 | -4.955486227 |
| Sao Tome and Principe | DALYs (Disability-Adjusted Life Years) | -6.066028012 | -6.746217167 | -5.380877587 |
| Sudan | DALYs (Disability-Adjusted Life Years) | -5.618105922 | -6.915157409 | -4.302981218 |
| Mauritius | DALYs (Disability-Adjusted Life Years) | -6.227926705 | -7.9341921 | -4.490038913 |
| Sweden | DALYs (Disability-Adjusted Life Years) | 0.723109307623648 | -0.424090649 | 1.88352599291539 |
| Algeria | DALYs (Disability-Adjusted Life Years) | -4.247907321 | -5.40423344 | -3.077446425 |
| Romania | DALYs (Disability-Adjusted Life Years) | -0.609410087 | -3.66142778 | 2.53929589776454 |
| Iceland | DALYs (Disability-Adjusted Life Years) | 0.622365524148338 | 0.00565543038335559 | 1.24287871622928 |
| Republic of Korea | DALYs (Disability-Adjusted Life Years) | -11.22646372 | -13.02949423 | -9.386053653 |
| Republic of Moldova | DALYs (Disability-Adjusted Life Years) | -2.844203382 | -4.536086967 | -1.122334959 |
| Latvia | DALYs (Disability-Adjusted Life Years) | -15.23120657 | -18.04430378 | -12.32155089 |
| Papua New Guinea | DALYs (Disability-Adjusted Life Years) | -4.812723891 | -9.37370007 | -0.022206136 |
| Zambia | DALYs (Disability-Adjusted Life Years) | -3.765612333 | -4.479397137 | -3.04649372 |
| Guinea | DALYs (Disability-Adjusted Life Years) | -2.068919682 | -2.85953135 | -1.271873344 |
| United Arab Emirates | DALYs (Disability-Adjusted Life Years) | -6.714282339 | -8.846499801 | -4.532189105 |
| Indonesia | DALYs (Disability-Adjusted Life Years) | -4.512852049 | -6.061383055 | -2.938794285 |
| Oman | DALYs (Disability-Adjusted Life Years) | -7.21942464 | -8.38948375 | -6.034421413 |
| Colombia | DALYs (Disability-Adjusted Life Years) | -3.652522179 | -5.118317746 | -2.164082025 |
| Puerto Rico | DALYs (Disability-Adjusted Life Years) | -5.726736858 | -8.79630806 | -2.553855504 |
| Sierra Leone | DALYs (Disability-Adjusted Life Years) | -4.632069524 | -5.647154308 | -3.606064061 |
| Ireland | DALYs (Disability-Adjusted Life Years) | -7.244505944 | -8.591037337 | -5.878139004 |
| Palau | DALYs (Disability-Adjusted Life Years) | -1.334413605 | -2.402817818 | -0.254313485 |
| United States of America | DALYs (Disability-Adjusted Life Years) | -0.198457012 | -1.543359093 | 1.16481621758902 |
| Singapore | DALYs (Disability-Adjusted Life Years) | -4.720020342 | -7.805244788 | -1.531551305 |
| Portugal | DALYs (Disability-Adjusted Life Years) | -6.49810241 | -8.344495979 | -4.614513375 |
| Botswana | DALYs (Disability-Adjusted Life Years) | -2.127772857 | -4.439698489 | 0.240086044795196 |
| Angola | DALYs (Disability-Adjusted Life Years) | -2.149530482 | -2.547314881 | -1.750122399 |
| Canada | DALYs (Disability-Adjusted Life Years) | -2.118650738 | -4.090120631 | -0.106656412 |
| Costa Rica | DALYs (Disability-Adjusted Life Years) | -6.7161476 | -9.638004832 | -3.699812045 |
| China | DALYs (Disability-Adjusted Life Years) | -8.942236728 | -9.589289108 | -8.290553517 |
| Greenland | DALYs (Disability-Adjusted Life Years) | -5.071555509 | -6.023080349 | -4.110396393 |
| Cuba | DALYs (Disability-Adjusted Life Years) | -5.334258405 | -8.649806248 | -1.898373023 |
| Brazil | DALYs (Disability-Adjusted Life Years) | -3.997864923 | -5.656216389 | -2.310363368 |
| Croatia | DALYs (Disability-Adjusted Life Years) | -5.49054558 | -7.896936524 | -3.021282489 |
| Israel | DALYs (Disability-Adjusted Life Years) | -3.008127064 | -5.14577201 | -0.822307924 |
| Yemen | DALYs (Disability-Adjusted Life Years) | -4.004109075 | -5.212133639 | -2.780688834 |
| Solomon Islands | DALYs (Disability-Adjusted Life Years) | -2.344645784 | -2.588008651 | -2.100674927 |
| Malaysia | DALYs (Disability-Adjusted Life Years) | -4.628611286 | -5.480745743 | -3.768794445 |
| Gambia | DALYs (Disability-Adjusted Life Years) | -3.421341936 | -4.311994016 | -2.522399776 |
| Suriname | DALYs (Disability-Adjusted Life Years) | -2.344481055 | -4.623927363 | -0.010556981 |
| Switzerland | DALYs (Disability-Adjusted Life Years) | -5.18658568 | -6.965981405 | -3.373156714 |
| Spain | DALYs (Disability-Adjusted Life Years) | -5.173571199 | -6.872821898 | -3.443315019 |
| Finland | DALYs (Disability-Adjusted Life Years) | -1.054879941 | -3.031059211 | 0.961572888753537 |
| Serbia | DALYs (Disability-Adjusted Life Years) | -4.944454289 | -6.396538524 | -3.469843658 |
| Taiwan (Province of China) | DALYs (Disability-Adjusted Life Years) | -5.486909464 | -6.397895129 | -4.567057599 |
| Kyrgyzstan | DALYs (Disability-Adjusted Life Years) | -3.304882418 | -4.146905484 | -2.455462586 |
| Kazakhstan | DALYs (Disability-Adjusted Life Years) | -6.756207447 | -8.527499873 | -4.950615348 |
| Cameroon | DALYs (Disability-Adjusted Life Years) | -3.861492221 | -4.682284585 | -3.033631914 |
| Viet Nam | DALYs (Disability-Adjusted Life Years) | -4.740142488 | -6.342968024 | -3.109886553 |
| Democratic People's Republic of Korea | DALYs (Disability-Adjusted Life Years) | -14.32317237 | -16.57166216 | -12.01408319 |
| Afghanistan | DALYs (Disability-Adjusted Life Years) | -4.106992326 | -5.417036214 | -2.778803362 |
| Qatar | DALYs (Disability-Adjusted Life Years) | -9.749389977 | -11.26978876 | -8.20293905 |
| Cyprus | DALYs (Disability-Adjusted Life Years) | -7.849528328 | -10.03153042 | -5.614606215 |
| Trinidad and Tobago | DALYs (Disability-Adjusted Life Years) | -3.736046642 | -6.96827192 | -0.391523328 |
| Mozambique | DALYs (Disability-Adjusted Life Years) | -6.110261415 | -7.378914678 | -4.824231104 |
| Bosnia and Herzegovina | DALYs (Disability-Adjusted Life Years) | -5.907842355 | -7.435515013 | -4.35495718 |
| El Salvador | DALYs (Disability-Adjusted Life Years) | -9.714190768 | -12.57296816 | -6.761934189 |
| Paraguay | DALYs (Disability-Adjusted Life Years) | -6.809456307 | -7.852470989 | -5.754635778 |
| Djibouti | DALYs (Disability-Adjusted Life Years) | -0.630678697 | -1.605987243 | 0.354297376679802 |
| Benin | DALYs (Disability-Adjusted Life Years) | -3.650602242 | -4.550650476 | -2.742066922 |
| Lao People's Democratic Republic | DALYs (Disability-Adjusted Life Years) | -6.065086722 | -8.091812571 | -3.993668254 |
| Grenada | DALYs (Disability-Adjusted Life Years) | -5.342687677 | -7.955735856 | -2.655457572 |
| Bolivia (Plurinational State of) | DALYs (Disability-Adjusted Life Years) | -10.38247468 | -12.58880298 | -8.120456901 |
| Australia | DALYs (Disability-Adjusted Life Years) | -4.659526214 | -8.041219755 | -1.153474226 |
| Monaco | DALYs (Disability-Adjusted Life Years) | -3.149794065 | -4.550940721 | -1.728079246 |
| Mongolia | DALYs (Disability-Adjusted Life Years) | -5.76937214 | -7.50691056 | -3.999193013 |
| Slovenia | DALYs (Disability-Adjusted Life Years) | -1.082876192 | -3.882164711 | 1.79793742788004 |
| Tonga | DALYs (Disability-Adjusted Life Years) | -7.293331868 | -7.822874061 | -6.760747544 |
| Czechia | DALYs (Disability-Adjusted Life Years) | -2.644600783 | -5.060537223 | -0.167185705 |
| Bulgaria | DALYs (Disability-Adjusted Life Years) | -0.249486492 | -3.065509834 | 2.64834454809013 |
| Dominica | DALYs (Disability-Adjusted Life Years) | -4.197291345 | -7.455402704 | -0.824475401 |
| Fiji | DALYs (Disability-Adjusted Life Years) | -5.323950359 | -7.891402926 | -2.684932131 |
| Marshall Islands | DALYs (Disability-Adjusted Life Years) | -1.572374308 | -2.464766299 | -0.671817437 |
| Lesotho | DALYs (Disability-Adjusted Life Years) | -2.807344966 | -5.465311784 | -0.074645923 |
| Congo | DALYs (Disability-Adjusted Life Years) | -0.776043666 | -1.728970029 | 0.18612314893065 |
| Saint Kitts and Nevis | DALYs (Disability-Adjusted Life Years) | -4.2932332 | -7.40385229 | -1.07811785 |
| Maldives | DALYs (Disability-Adjusted Life Years) | -7.331045808 | -9.084874178 | -5.543384631 |
| Central African Republic | DALYs (Disability-Adjusted Life Years) | 0.0459189473736465 | -0.398525772 | 0.492346881665062 |
| France | DALYs (Disability-Adjusted Life Years) | -4.91761557 | -6.418707209 | -3.392445656 |
| Malta | DALYs (Disability-Adjusted Life Years) | -6.784450801 | -9.083584248 | -4.427175877 |
| Cabo Verde | DALYs (Disability-Adjusted Life Years) | -8.942772635 | -10.77650132 | -7.071357005 |
| Kiribati | DALYs (Disability-Adjusted Life Years) | -0.240970385 | -0.646111552 | 0.165822849111286 |
| Kuwait | DALYs (Disability-Adjusted Life Years) | -4.121145498 | -5.638591725 | -2.579296891 |
| Mali | DALYs (Disability-Adjusted Life Years) | -3.738291295 | -4.572989807 | -2.896291689 |
| Tokelau | DALYs (Disability-Adjusted Life Years) | -6.157794958 | -6.464787121 | -5.849795216 |
| Eritrea | DALYs (Disability-Adjusted Life Years) | -7.555565659 | -8.848618603 | -6.244169759 |
| Burkina Faso | DALYs (Disability-Adjusted Life Years) | -7.228755996 | -8.100359599 | -6.348885845 |
| Somalia | DALYs (Disability-Adjusted Life Years) | 0.31643045662304 | -0.496516032 | 1.1360187432401 |
| Slovakia | DALYs (Disability-Adjusted Life Years) | -3.643643198 | -5.146285209 | -2.117196816 |
| Guam | DALYs (Disability-Adjusted Life Years) | -3.826027599 | -4.986159535 | -2.651730293 |
| Tunisia | DALYs (Disability-Adjusted Life Years) | -8.005421482 | -10.52236792 | -5.417675009 |
| Russian Federation | DALYs (Disability-Adjusted Life Years) | -7.935119361 | -9.197967953 | -6.654707434 |
| Iran (Islamic Republic of) | DALYs (Disability-Adjusted Life Years) | -6.581276679 | -8.066503135 | -5.07205573 |
| Rwanda | DALYs (Disability-Adjusted Life Years) | -9.04738187 | -10.08245678 | -8.000391824 |
| Comoros | DALYs (Disability-Adjusted Life Years) | -3.820028469 | -4.888742509 | -2.739305865 |
| Myanmar | DALYs (Disability-Adjusted Life Years) | -5.447291382 | -7.749719315 | -3.087398318 |
| Mauritania | DALYs (Disability-Adjusted Life Years) | -4.4050928 | -5.082826411 | -3.722519993 |
| Guinea-Bissau | DALYs (Disability-Adjusted Life Years) | -3.49501139 | -4.24444325 | -2.739714093 |
| Burundi | DALYs (Disability-Adjusted Life Years) | -8.43598389 | -9.594021522 | -7.263112602 |
| Iraq | DALYs (Disability-Adjusted Life Years) | -4.89162893 | -6.772479629 | -2.97283236 |
| Vanuatu | DALYs (Disability-Adjusted Life Years) | -4.765733221 | -8.158266094 | -1.247883906 |
| Togo | DALYs (Disability-Adjusted Life Years) | -2.735669197 | -3.561488156 | -1.902778613 |
| New Zealand | DALYs (Disability-Adjusted Life Years) | -6.90302763 | -9.902923494 | -3.803246446 |
| Democratic Republic of the Congo | DALYs (Disability-Adjusted Life Years) | -2.303245647 | -2.740624137 | -1.863900251 |
| Luxembourg | DALYs (Disability-Adjusted Life Years) | -7.98536925 | -9.674200798 | -6.264961431 |
| Uzbekistan | DALYs (Disability-Adjusted Life Years) | -0.290361355 | -2.140203783 | 1.59444862066036 |
| San Marino | DALYs (Disability-Adjusted Life Years) | 1.70132872401914 | -0.165407242 | 3.60296945677512 |
| Ecuador | DALYs (Disability-Adjusted Life Years) | -6.082136364 | -7.208925989 | -4.941663798 |
| Guyana | DALYs (Disability-Adjusted Life Years) | -5.709260412 | -8.82107212 | -2.49124684 |
| United Republic of Tanzania | DALYs (Disability-Adjusted Life Years) | -3.596336359 | -4.305631572 | -2.881783787 |
| Tajikistan | DALYs (Disability-Adjusted Life Years) | -5.217897933 | -6.437450465 | -3.982448995 |
| Italy | DALYs (Disability-Adjusted Life Years) | -12.66958629 | -14.36888314 | -10.93656794 |
| Antigua and Barbuda | DALYs (Disability-Adjusted Life Years) | -2.423599139 | -5.343546111 | 0.586421883817478 |
| Seychelles | DALYs (Disability-Adjusted Life Years) | -4.317197214 | -6.178551357 | -2.418915064 |
| Turkey | DALYs (Disability-Adjusted Life Years) | -9.902470173 | -11.20378835 | -8.582081037 |
| Saudi Arabia | DALYs (Disability-Adjusted Life Years) | -6.413563373 | -7.119230465 | -5.702534934 |
| Honduras | DALYs (Disability-Adjusted Life Years) | -3.63301893 | -4.625491475 | -2.630218659 |
| Ukraine | DALYs (Disability-Adjusted Life Years) | 3.30247616904935 | 0.0374315250391444 | 6.67408608932529 |
| Syrian Arab Republic | DALYs (Disability-Adjusted Life Years) | -0.253855994 | -1.500630865 | 1.00870017137649 |
| Bangladesh | DALYs (Disability-Adjusted Life Years) | -7.933336147 | -8.560900438 | -7.30146476 |
| Guatemala | DALYs (Disability-Adjusted Life Years) | -8.184575472 | -9.895569687 | -6.441091166 |
| Liberia | DALYs (Disability-Adjusted Life Years) | -5.802215326 | -6.96260374 | -4.627354223 |
| United Kingdom | DALYs (Disability-Adjusted Life Years) | -4.058860084 | -5.946928114 | -2.132890039 |
| Dominican Republic | DALYs (Disability-Adjusted Life Years) | -4.553690108 | -6.292159579 | -2.782968522 |
| Netherlands | DALYs (Disability-Adjusted Life Years) | 0.315260838996445 | -1.47550356 | 2.13857386555127 |
| Denmark | DALYs (Disability-Adjusted Life Years) | -4.439903066 | -7.161576324 | -1.638440589 |
| Jordan | DALYs (Disability-Adjusted Life Years) | -3.050677319 | -4.822862675 | -1.245494112 |
| Bhutan | DALYs (Disability-Adjusted Life Years) | -13.5738519 | -14.71417149 | -12.4182856 |
| Chad | DALYs (Disability-Adjusted Life Years) | -1.423797061 | -2.324411912 | -0.514878117 |
| Namibia | DALYs (Disability-Adjusted Life Years) | -6.17468003 | -7.881553637 | -4.43617956 |
| Hungary | DALYs (Disability-Adjusted Life Years) | -5.204903542 | -7.702255784 | -2.639979028 |
| Bahamas | DALYs (Disability-Adjusted Life Years) | -4.891170601 | -7.749267366 | -1.944524761 |
| Turkmenistan | DALYs (Disability-Adjusted Life Years) | -9.478209085 | -10.83876282 | -8.096894017 |
| Nauru | DALYs (Disability-Adjusted Life Years) | -8.60143523 | -10.24029617 | -6.93265145 |
| Belarus | DALYs (Disability-Adjusted Life Years) | -5.212128139 | -6.566533063 | -3.838089857 |
| Norway | DALYs (Disability-Adjusted Life Years) | -3.952418087 | -6.225582087 | -1.624150843 |
| Coted'Ivoire | DALYs (Disability-Adjusted Life Years) | -3.741279319 | -4.961896486 | -2.504985216 |

Sup_Table 12 Decomposition Analysis of Global Adolescent Pertussis Prevalence Changes (1990-2021): Global, 5 SDI Regions, and 21 GBD Regions

| location | sex | cause | measure | Overll difference | Aging | Population | Epidemiological change |
| --- | --- | --- | --- | --- | --- | --- | --- |
| Global | Both | Pertussis | Prevalence | -89747.66(-74.87%) | 907.55 (0.76%) | 16168.92 (13.49%) | -106824.13 (-89.11%) |
| High SDI | Both | Pertussis | Prevalence | -5005.33(-93.77%) | 55.43 (1.04%) | -153.33 (-2.87%) | -4907.44 (-91.93%) |
| High-middle SDI | Both | Pertussis | Prevalence | -12336.59(-94.84%) | 332.33 (2.55%) | -1331.23 (-10.23%) | -11337.69 (-87.16%) |
| Middle SDI | Both | Pertussis | Prevalence | -30856.62(-85.64%) | 545.2 (1.51%) | 278.39 (0.77%) | -31680.21 (-87.93%) |
| Low-middle SDI | Both | Pertussis | Prevalence | -32211.78(-75.61%) | -961.44 (-2.26%) | 11538.98 (27.08%) | -42789.33 (-100.43%) |
| Low SDI | Both | Pertussis | Prevalence | -9262.3(-40.6%) | -523.73 (-2.3%) | 19144.84 (83.92%) | -27883.4 (-122.23%) |
| Andean Latin America | Both | Pertussis | Prevalence | -620.83(-62.91%) | -16.29 (-1.65%) | 194.66 (19.72%) | -799.21 (-80.98%) |
| Australasia | Both | Pertussis | Prevalence | -131.58(-99.25%) | 4.42 (3.34%) | 12.05 (9.09%) | -148.05 (-111.68%) |
| Caribbean | Both | Pertussis | Prevalence | -737.09(-99.58%) | 5.05 (0.68%) | 14.72 (1.99%) | -756.86 (-102.25%) |
| Central Asia | Both | Pertussis | Prevalence | -1193.02(-94.08%) | 16.87 (1.33%) | 67.08 (5.29%) | -1276.98 (-100.7%) |
| Central Europe | Both | Pertussis | Prevalence | -681.05(-99.22%) | 0.02 (0%) | -139.58 (-20.34%) | -541.49 (-78.89%) |
| Central Latin America | Both | Pertussis | Prevalence | -4405.08(-91.35%) | -82.8 (-1.72%) | 351.25 (7.28%) | -4673.54 (-96.92%) |
| Central Sub-Saharan Africa | Both | Pertussis | Prevalence | 1263.78(52.12%) | 1.8 (0.07%) | 3101.26 (127.89%) | -1839.28 (-75.85%) |
| East Asia | Both | Pertussis | Prevalence | -13688.85(-95.1%) | 809.93 (5.63%) | -2354.9 (-16.36%) | -12143.87 (-84.37%) |
| Eastern Europe | Both | Pertussis | Prevalence | -3949.91(-98.14%) | 64.25 (1.6%) | -590.76 (-14.68%) | -3423.4 (-85.06%) |
| Eastern Sub-Saharan Africa | Both | Pertussis | Prevalence | -2708.19(-36.07%) | -211.96 (-2.82%) | 6191.71 (82.48%) | -8687.94 (-115.73%) |
| High-income Asia Pacific | Both | Pertussis | Prevalence | -1837.09(-99.98%) | 32.76 (1.78%) | -385.66 (-20.99%) | -1484.19 (-80.78%) |
| High-income North America | Both | Pertussis | Prevalence | -407.84(-64.75%) | -0.14 (-0.02%) | 78.84 (12.52%) | -486.54 (-77.25%) |
| North Africa and Middle East | Both | Pertussis | Prevalence | -6559.31(-80.1%) | -61.98 (-0.76%) | 2030.81 (24.8%) | -8528.13 (-104.14%) |
| Oceania | Both | Pertussis | Prevalence | -204.72(-91.86%) | -0.76 (-0.34%) | 102.76 (46.11%) | -306.71 (-137.63%) |
| South Asia | Both | Pertussis | Prevalence | -30576.49(-74.21%) | -1269.2 (-3.08%) | 12022.32 (29.18%) | -41329.62 (-100.31%) |
| Southeast Asia | Both | Pertussis | Prevalence | -13145.47(-95.76%) | -187.06 (-1.36%) | 742.41 (5.41%) | -13700.82 (-99.8%) |
| Southern Latin America | Both | Pertussis | Prevalence | -639.8(-95.43%) | -7.14 (-1.07%) | 31.63 (4.72%) | -664.29 (-99.09%) |
| Southern Sub-Saharan Africa | Both | Pertussis | Prevalence | -825.36(-76.59%) | -0.24 (-0.02%) | 157.78 (14.64%) | -982.9 (-91.21%) |
| Tropical Latin America | Both | Pertussis | Prevalence | -3486.3(-87.33%) | -97.05 (-2.43%) | -54.52 (-1.37%) | -3334.73 (-83.54%) |
| Western Europe | Both | Pertussis | Prevalence | -2844.2(-98.28%) | 58.43 (2.02%) | -125.59 (-4.34%) | -2777.04 (-95.96%) |
| Western Sub-Saharan Africa | Both | Pertussis | Prevalence | -2369.25(-28.06%) | -63.45 (-0.75%) | 9120.85 (108.02%) | -11426.65 (-135.33%) |

Sup_Table 13 Decomposition Analysis of Global Adolescent Pertussis Incidence Changes (1990-2021): Global, 5 SDI Regions, and 21 GBD Regions.

| location | sex | cause | measure | Overll difference | Aging | Population | Epidemiological change |
| --- | --- | --- | --- | --- | --- | --- | --- |
| Global | Both | Pertussis | Incidence | -655157.91(-74.87%) | 6625.11 (0.76%) | 118033.14 (13.49%) | -779816.15 (-89.11%) |
| High SDI | Both | Pertussis | Incidence | -36538.93(-93.77%) | 404.66 (1.04%) | -1119.31 (-2.87%) | -35824.28 (-91.93%) |
| High-middle SDI | Both | Pertussis | Incidence | -90057.08(-94.84%) | 2426.03 (2.55%) | -9718.01 (-10.23%) | -82765.11 (-87.16%) |
| Middle SDI | Both | Pertussis | Incidence | -225253.33(-85.64%) | 3979.98 (1.51%) | 2032.24 (0.77%) | -231265.55 (-87.93%) |
| Low-middle SDI | Both | Pertussis | Incidence | -235146.03(-75.61%) | -7018.53 (-2.26%) | 84234.58 (27.08%) | -312362.08 (-100.43%) |
| Low SDI | Both | Pertussis | Incidence | -67614.76(-40.6%) | -3823.21 (-2.3%) | 139757.3 (83.92%) | -203548.85 (-122.23%) |
| Andean Latin America | Both | Pertussis | Incidence | -4532.08(-62.91%) | -118.91 (-1.65%) | 1421.04 (19.72%) | -5834.21 (-80.98%) |
| Australasia | Both | Pertussis | Incidence | -960.51(-99.25%) | 32.29 (3.34%) | 87.97 (9.09%) | -1080.77 (-111.68%) |
| Caribbean | Both | Pertussis | Incidence | -5380.75(-99.58%) | 36.87 (0.68%) | 107.48 (1.99%) | -5525.1 (-102.25%) |
| Central Asia | Both | Pertussis | Incidence | -8709.06(-94.08%) | 123.15 (1.33%) | 489.71 (5.29%) | -9321.92 (-100.7%) |
| Central Europe | Both | Pertussis | Incidence | -4971.65(-99.22%) | 0.16 (0%) | -1018.96 (-20.34%) | -3952.86 (-78.89%) |
| Central Latin America | Both | Pertussis | Incidence | -32157.09(-91.35%) | -604.41 (-1.72%) | 2564.14 (7.28%) | -34116.81 (-96.92%) |
| Central Sub-Saharan Africa | Both | Pertussis | Incidence | 9225.58(52.12%) | 13.11 (0.07%) | 22639.22 (127.89%) | -13426.75 (-75.85%) |
| East Asia | Both | Pertussis | Incidence | -99928.57(-95.1%) | 5912.49 (5.63%) | -17190.8 (-16.36%) | -88650.27 (-84.37%) |
| Eastern Europe | Both | Pertussis | Incidence | -28834.35(-98.14%) | 469.03 (1.6%) | -4312.56 (-14.68%) | -24990.83 (-85.06%) |
| Eastern Sub-Saharan Africa | Both | Pertussis | Incidence | -19769.78(-36.07%) | -1547.31 (-2.82%) | 45199.49 (82.48%) | -63421.96 (-115.73%) |
| High-income Asia Pacific | Both | Pertussis | Incidence | -13410.75(-99.98%) | 239.17 (1.78%) | -2815.33 (-20.99%) | -10834.58 (-80.78%) |
| High-income North America | Both | Pertussis | Incidence | -2977.26(-64.75%) | -1.03 (-0.02%) | 575.53 (12.52%) | -3551.76 (-77.25%) |
| North Africa and Middle East | Both | Pertussis | Incidence | -47882.96(-80.1%) | -452.49 (-0.76%) | 14824.88 (24.8%) | -62255.35 (-104.14%) |
| Oceania | Both | Pertussis | Incidence | -1494.43(-91.86%) | -5.53 (-0.34%) | 750.12 (46.11%) | -2239.02 (-137.63%) |
| South Asia | Both | Pertussis | Incidence | -223208.41(-74.21%) | -9265.16 (-3.08%) | 87762.94 (29.18%) | -301706.2 (-100.31%) |
| Southeast Asia | Both | Pertussis | Incidence | -95961.94(-95.76%) | -1365.54 (-1.36%) | 5419.59 (5.41%) | -100015.99 (-99.8%) |
| Southern Latin America | Both | Pertussis | Incidence | -4670.54(-95.43%) | -52.13 (-1.07%) | 230.93 (4.72%) | -4849.35 (-99.09%) |
| Southern Sub-Saharan Africa | Both | Pertussis | Incidence | -6025.14(-76.59%) | -1.78 (-0.02%) | 1151.81 (14.64%) | -7175.17 (-91.21%) |
| Tropical Latin America | Both | Pertussis | Incidence | -25450(-87.33%) | -708.45 (-2.43%) | -398.03 (-1.37%) | -24343.53 (-83.54%) |
| Western Europe | Both | Pertussis | Incidence | -20762.66(-98.28%) | 426.57 (2.02%) | -916.84 (-4.34%) | -20272.39 (-95.96%) |
| Western Sub-Saharan Africa | Both | Pertussis | Incidence | -17295.55(-28.06%) | -463.19 (-0.75%) | 66582.2 (108.02%) | -83414.56 (-135.33%) |

Sup_Table 14 Decomposition Analysis of Global Adolescent Pertussis Deaths Changes (1990-2021): Global, 5 SDI Regions, and 21 GBD Regions

| location | sex | cause | measure | Overll difference | Aging | Population | Epidemiological change |
| --- | --- | --- | --- | --- | --- | --- | --- |
| Global | Both | Pertussis | Deaths | -6136.24(-79.86%) | 54.02 (0.7%) | 1001.42 (13.03%) | -7191.69 (-93.6%) |
| High SDI | Both | Pertussis | Deaths | -36.15(-97.05%) | 0.36 (0.97%) | -1.04 (-2.78%) | -35.48 (-95.25%) |
| High-middle SDI | Both | Pertussis | Deaths | -329.17(-94.85%) | 8.32 (2.4%) | -35.48 (-10.22%) | -302.01 (-87.03%) |
| Middle SDI | Both | Pertussis | Deaths | -1739.1(-90.16%) | 26.55 (1.38%) | 14.33 (0.74%) | -1779.97 (-92.28%) |
| Low-middle SDI | Both | Pertussis | Deaths | -2657.76(-83.7%) | -64.14 (-2.02%) | 819.03 (25.79%) | -3412.65 (-107.47%) |
| Low SDI | Both | Pertussis | Deaths | -1369.4(-62.53%) | -43.3 (-1.98%) | 1699.4 (77.6%) | -3025.5 (-138.16%) |
| Andean Latin America | Both | Pertussis | Deaths | -51.05(-88.94%) | -0.79 (-1.38%) | 9.54 (16.63%) | -59.8 (-104.2%) |
| Australasia | Both | Pertussis | Deaths | 0(-99.46%) | 0 (-3.47%) | 0 (8.7%) | 0 (-104.69%) |
| Caribbean | Both | Pertussis | Deaths | -50.47(-99.19%) | 0.36 (0.71%) | 1.02 (2%) | -51.85 (-101.9%) |
| Central Asia | Both | Pertussis | Deaths | -20.27(-93.32%) | 0.28 (1.29%) | 1.16 (5.32%) | -21.71 (-99.93%) |
| Central Europe | Both | Pertussis | Deaths | -7.06(-97.99%) | 0 (0%) | -1.49 (-20.75%) | -5.57 (-77.25%) |
| Central Latin America | Both | Pertussis | Deaths | -31.98(-94.26%) | -0.51 (-1.51%) | 2.41 (7.11%) | -33.88 (-99.86%) |
| Central Sub-Saharan Africa | Both | Pertussis | Deaths | 8.59(3.82%) | 0.14 (0.06%) | 254.17 (112.97%) | -245.72 (-109.21%) |
| East Asia | Both | Pertussis | Deaths | -819.29(-95%) | 47.32 (5.49%) | -141.19 (-16.37%) | -725.42 (-84.12%) |
| Eastern Europe | Both | Pertussis | Deaths | -0.11(-97.67%) | 0 (1.17%) | -0.02 (-14.72%) | -0.09 (-84.12%) |
| Eastern Sub-Saharan Africa | Both | Pertussis | Deaths | -476.08(-59.08%) | -19.04 (-2.36%) | 612.04 (75.95%) | -1069.09 (-132.67%) |
| High-income Asia Pacific | Both | Pertussis | Deaths | -21.78(-99.9%) | 0.37 (1.7%) | -4.58 (-21%) | -17.57 (-80.59%) |
| High-income North America | Both | Pertussis | Deaths | -0.06(-67.14%) | 0 (-0.02%) | 0.01 (12.32%) | -0.08 (-79.44%) |
| North Africa and Middle East | Both | Pertussis | Deaths | -469.92(-89.08%) | -3.72 (-0.7%) | 123.61 (23.43%) | -589.81 (-111.8%) |
| Oceania | Both | Pertussis | Deaths | -14.71(-89.76%) | -0.06 (-0.34%) | 7.64 (46.6%) | -22.29 (-136.02%) |
| South Asia | Both | Pertussis | Deaths | -2782.94(-84.4%) | -88.1 (-2.67%) | 905.69 (27.47%) | -3600.53 (-109.2%) |
| Southeast Asia | Both | Pertussis | Deaths | -912.02(-96.59%) | -11.86 (-1.26%) | 50.71 (5.37%) | -950.87 (-100.71%) |
| Southern Latin America | Both | Pertussis | Deaths | -0.05(-93.31%) | 0 (-1.53%) | 0 (4.8%) | -0.06 (-96.58%) |
| Southern Sub-Saharan Africa | Both | Pertussis | Deaths | -43.06(-73.29%) | -0.01 (-0.02%) | 8.8 (14.97%) | -51.85 (-88.23%) |
| Tropical Latin America | Both | Pertussis | Deaths | -4.88(-92.53%) | -0.12 (-2.26%) | -0.07 (-1.3%) | -4.69 (-88.97%) |
| Western Europe | Both | Pertussis | Deaths | -0.2(-98.21%) | 0 (0.77%) | -0.01 (-4.31%) | -0.2 (-94.68%) |
| Western Sub-Saharan Africa | Both | Pertussis | Deaths | -438.9(-58.7%) | -5.04 (-0.67%) | 735.26 (98.33%) | -1169.12 (-156.35%) |

Sup_Table 15 Decomposition Analysis of Global Adolescent Pertussis DALYs Changes (1990-2021): Global, 5 SDI Regions, and 21 GBD Regions

| location | sex | cause | measure | Overll difference | Aging | Population | Epidemiological change |
| --- | --- | --- | --- | --- | --- | --- | --- |
| Global | Both | Pertussis | DALYs | -475253.63(-79.82%) | 4312.6 (0.72%) | 77623.28 (13.04%) | -557189.52 (-93.58%) |
| High SDI | Both | Pertussis | DALYs | -3016.8(-96.77%) | 31.33 (1%) | -86.91 (-2.79%) | -2961.21 (-94.99%) |
| High-middle SDI | Both | Pertussis | DALYs | -25794.77(-94.85%) | 674.03 (2.48%) | -2781.8 (-10.23%) | -23687.01 (-87.1%) |
| Middle SDI | Both | Pertussis | DALYs | -134711.59(-90.11%) | 2125.64 (1.42%) | 1110.95 (0.74%) | -137948.18 (-92.28%) |
| Low-middle SDI | Both | Pertussis | DALYs | -205632.49(-83.65%) | -5125.54 (-2.09%) | 63401.15 (25.79%) | -263908.1 (-107.36%) |
| Low SDI | Both | Pertussis | DALYs | -105736.69(-62.43%) | -3454.79 (-2.04%) | 131462.76 (77.62%) | -233744.66 (-138.01%) |
| Andean Latin America | Both | Pertussis | DALYs | -3951.92(-88.68%) | -63.21 (-1.42%) | 742.3 (16.66%) | -4631.01 (-103.92%) |
| Australasia | Both | Pertussis | DALYs | -6.69(-99.25%) | 0.22 (3.27%) | 0.61 (9.09%) | -7.53 (-111.61%) |
| Caribbean | Both | Pertussis | DALYs | -3911.86(-99.2%) | 28.68 (0.73%) | 78.75 (2%) | -4019.29 (-101.92%) |
| Central Asia | Both | Pertussis | DALYs | -1611.96(-93.32%) | 23.05 (1.33%) | 91.95 (5.32%) | -1726.97 (-99.98%) |
| Central Europe | Both | Pertussis | DALYs | -575.27(-98.07%) | 0.02 (0%) | -121.55 (-20.72%) | -453.74 (-77.35%) |
| Central Latin America | Both | Pertussis | DALYs | -2671.3(-94.04%) | -44.83 (-1.58%) | 202.38 (7.12%) | -2828.85 (-99.58%) |
| Central Sub-Saharan Africa | Both | Pertussis | DALYs | 719.56(4.14%) | 10.9 (0.06%) | 19667.37 (113.07%) | -18958.71 (-109%) |
| East Asia | Both | Pertussis | DALYs | -63269.54(-95%) | 3774.13 (5.67%) | -10916.37 (-16.39%) | -56127.3 (-84.27%) |
| Eastern Europe | Both | Pertussis | DALYs | -205.27(-98.1%) | 3.29 (1.57%) | -30.72 (-14.68%) | -177.83 (-84.99%) |
| Eastern Sub-Saharan Africa | Both | Pertussis | DALYs | -36750.35(-59.02%) | -1519.65 (-2.44%) | 47294.46 (75.96%) | -82525.17 (-132.54%) |
| High-income Asia Pacific | Both | Pertussis | DALYs | -1756.49(-99.9%) | 30.91 (1.76%) | -369.44 (-21.01%) | -1417.96 (-80.65%) |
| High-income North America | Both | Pertussis | DALYs | -25.69(-65.35%) | -0.01 (-0.02%) | 4.9 (12.47%) | -30.58 (-77.79%) |
| North Africa and Middle East | Both | Pertussis | DALYs | -36402.86(-88.98%) | -296.32 (-0.72%) | 9591.7 (23.44%) | -45698.25 (-111.7%) |
| Oceania | Both | Pertussis | DALYs | -1139.57(-89.79%) | -4.48 (-0.35%) | 591.32 (46.59%) | -1726.41 (-136.03%) |
| South Asia | Both | Pertussis | DALYs | -215156.59(-84.36%) | -7045.08 (-2.76%) | 70043.1 (27.46%) | -278154.62 (-109.07%) |
| Southeast Asia | Both | Pertussis | DALYs | -70593.84(-96.61%) | -948.39 (-1.3%) | 3922.75 (5.37%) | -73568.2 (-100.68%) |
| Southern Latin America | Both | Pertussis | DALYs | -36.11(-95.1%) | -0.42 (-1.12%) | 1.8 (4.73%) | -37.48 (-98.71%) |
| Southern Sub-Saharan Africa | Both | Pertussis | DALYs | -3332.99(-73.19%) | -1.07 (-0.02%) | 682.16 (14.98%) | -4014.08 (-88.14%) |
| Tropical Latin America | Both | Pertussis | DALYs | -547.52(-90.75%) | -14.22 (-2.36%) | -7.98 (-1.32%) | -525.32 (-87.07%) |
| Western Europe | Both | Pertussis | DALYs | -157.27(-98.25%) | 3.03 (1.89%) | -6.94 (-4.34%) | -153.36 (-95.81%) |
| Western Sub-Saharan Africa | Both | Pertussis | DALYs | -33870.1(-58.48%) | -400.47 (-0.69%) | 56984.06 (98.39%) | -90453.68 (-156.19%) |

Sup_Table 16 Global Adolescent Pertussis Forecasts: Prevalence, 1992 to 2046

| measure | location | cause | sex | year | ASR | case |
| --- | --- | --- | --- | --- | --- | --- |
| Prevalence | Global | Pertussis | Both | 1992 | 10.46 | 110805 |
| Prevalence | Global | Pertussis | Both | 1993 | 10.06 | 108066 |
| Prevalence | Global | Pertussis | Both | 1994 | 9.75 | 106295 |
| Prevalence | Global | Pertussis | Both | 1995 | 9.43 | 104600 |
| Prevalence | Global | Pertussis | Both | 1996 | 9.13 | 103165 |
| Prevalence | Global | Pertussis | Both | 1997 | 8.87 | 102098 |
| Prevalence | Global | Pertussis | Both | 1998 | 8.64 | 101105 |
| Prevalence | Global | Pertussis | Both | 1999 | 8.50 | 100746 |
| Prevalence | Global | Pertussis | Both | 2000 | 8.42 | 100710 |
| Prevalence | Global | Pertussis | Both | 2001 | 8.36 | 100516 |
| Prevalence | Global | Pertussis | Both | 2002 | 8.28 | 99568 |
| Prevalence | Global | Pertussis | Both | 2003 | 8.19 | 98401 |
| Prevalence | Global | Pertussis | Both | 2004 | 8.04 | 96210 |
| Prevalence | Global | Pertussis | Both | 2005 | 7.89 | 93992 |
| Prevalence | Global | Pertussis | Both | 2006 | 7.76 | 92041 |
| Prevalence | Global | Pertussis | Both | 2007 | 7.66 | 90668 |
| Prevalence | Global | Pertussis | Both | 2008 | 7.59 | 89554 |
| Prevalence | Global | Pertussis | Both | 2009 | 7.52 | 88610 |
| Prevalence | Global | Pertussis | Both | 2010 | 7.44 | 87571 |
| Prevalence | Global | Pertussis | Both | 2011 | 7.35 | 86616 |
| Prevalence | Global | Pertussis | Both | 2012 | 7.25 | 85556 |
| Prevalence | Global | Pertussis | Both | 2013 | 7.14 | 84653 |
| Prevalence | Global | Pertussis | Both | 2014 | 7.08 | 84399 |
| Prevalence | Global | Pertussis | Both | 2015 | 7.07 | 84833 |
| Prevalence | Global | Pertussis | Both | 2016 | 7.02 | 84974 |
| Prevalence | Global | Pertussis | Both | 2017 | 6.94 | 85003 |
| Prevalence | Global | Pertussis | Both | 2018 | 6.85 | 85051 |
| Prevalence | Global | Pertussis | Both | 2019 | 6.73 | 84629 |
| Prevalence | Global | Pertussis | Both | 2020 | 3.55 | 45171 |
| Prevalence | Global | Pertussis | Both | 2021 | 2.35 | 30128 |
| Prevalence | Global | Pertussis | Both | 2022 | 4.79 | 61612 |
| Prevalence | Global | Pertussis | Both | 2023 | 4.64 | 60138 |
| Prevalence | Global | Pertussis | Both | 2024 | 4.49 | 58516 |
| Prevalence | Global | Pertussis | Both | 2025 | 4.41 | 57813 |
| Prevalence | Global | Pertussis | Both | 2026 | 4.34 | 57041 |
| Prevalence | Global | Pertussis | Both | 2027 | 4.26 | 56161 |
| Prevalence | Global | Pertussis | Both | 2028 | 4.18 | 55090 |
| Prevalence | Global | Pertussis | Both | 2029 | 4.11 | 53858 |
| Prevalence | Global | Pertussis | Both | 2030 | 4.06 | 52862 |
| Prevalence | Global | Pertussis | Both | 2031 | 4.01 | 51735 |
| Prevalence | Global | Pertussis | Both | 2032 | 3.96 | 50560 |
| Prevalence | Global | Pertussis | Both | 2033 | 3.92 | 49505 |
| Prevalence | Global | Pertussis | Both | 2034 | 3.87 | 48605 |
| Prevalence | Global | Pertussis | Both | 2035 | 3.84 | 48161 |
| Prevalence | Global | Pertussis | Both | 2036 | 3.82 | 47844 |
| Prevalence | Global | Pertussis | Both | 2037 | 3.80 | 47575 |
| Prevalence | Global | Pertussis | Both | 2038 | 3.78 | 47328 |
| Prevalence | Global | Pertussis | Both | 2039 | 3.75 | 47104 |
| Prevalence | Global | Pertussis | Both | 2040 | 3.73 | 46912 |
| Prevalence | Global | Pertussis | Both | 2041 | 3.71 | 46741 |
| Prevalence | Global | Pertussis | Both | 2042 | 3.68 | 46580 |
| Prevalence | Global | Pertussis | Both | 2043 | 3.66 | 46421 |
| Prevalence | Global | Pertussis | Both | 2044 | 3.64 | 46261 |
| Prevalence | Global | Pertussis | Both | 2045 | 3.62 | 46100 |
| Prevalence | Global | Pertussis | Both | 2046 | 3.59 | 45936 |

Sup_Table 17 Global Adolescent Pertussis Forecasts: Incidence, 1992 to 2046

| measure | location | cause | sex | year | ASR | case |
| --- | --- | --- | --- | --- | --- | --- |
| Incidence | Global | Pertussis | Both | 1992 | 76.32 | 808877 |
| Incidence | Global | Pertussis | Both | 1993 | 73.44 | 788881 |
| Incidence | Global | Pertussis | Both | 1994 | 71.15 | 775956 |
| Incidence | Global | Pertussis | Both | 1995 | 68.85 | 763582 |
| Incidence | Global | Pertussis | Both | 1996 | 66.66 | 753103 |
| Incidence | Global | Pertussis | Both | 1997 | 64.77 | 745318 |
| Incidence | Global | Pertussis | Both | 1998 | 63.10 | 738068 |
| Incidence | Global | Pertussis | Both | 1999 | 62.05 | 735448 |
| Incidence | Global | Pertussis | Both | 2000 | 61.45 | 735184 |
| Incidence | Global | Pertussis | Both | 2001 | 61.05 | 733770 |
| Incidence | Global | Pertussis | Both | 2002 | 60.42 | 726843 |
| Incidence | Global | Pertussis | Both | 2003 | 59.82 | 718329 |
| Incidence | Global | Pertussis | Both | 2004 | 58.70 | 702331 |
| Incidence | Global | Pertussis | Both | 2005 | 57.62 | 686139 |
| Incidence | Global | Pertussis | Both | 2006 | 56.64 | 671903 |
| Incidence | Global | Pertussis | Both | 2007 | 55.95 | 661873 |
| Incidence | Global | Pertussis | Both | 2008 | 55.38 | 653747 |
| Incidence | Global | Pertussis | Both | 2009 | 54.89 | 646852 |
| Incidence | Global | Pertussis | Both | 2010 | 54.28 | 639270 |
| Incidence | Global | Pertussis | Both | 2011 | 53.66 | 632296 |
| Incidence | Global | Pertussis | Both | 2012 | 52.90 | 624556 |
| Incidence | Global | Pertussis | Both | 2013 | 52.15 | 617964 |
| Incidence | Global | Pertussis | Both | 2014 | 51.71 | 616114 |
| Incidence | Global | Pertussis | Both | 2015 | 51.60 | 619278 |
| Incidence | Global | Pertussis | Both | 2016 | 51.22 | 620313 |
| Incidence | Global | Pertussis | Both | 2017 | 50.65 | 620524 |
| Incidence | Global | Pertussis | Both | 2018 | 50.03 | 620873 |
| Incidence | Global | Pertussis | Both | 2019 | 49.13 | 617794 |
| Incidence | Global | Pertussis | Both | 2020 | 25.92 | 329749 |
| Incidence | Global | Pertussis | Both | 2021 | 17.13 | 219934 |
| Incidence | Global | Pertussis | Both | 2022 | 35.02 | 449961 |
| Incidence | Global | Pertussis | Both | 2023 | 33.90 | 439267 |
| Incidence | Global | Pertussis | Both | 2024 | 32.79 | 427500 |
| Incidence | Global | Pertussis | Both | 2025 | 32.23 | 422414 |
| Incidence | Global | Pertussis | Both | 2026 | 31.68 | 416831 |
| Incidence | Global | Pertussis | Both | 2027 | 31.13 | 410464 |
| Incidence | Global | Pertussis | Both | 2028 | 30.58 | 402695 |
| Incidence | Global | Pertussis | Both | 2029 | 30.02 | 393752 |
| Incidence | Global | Pertussis | Both | 2030 | 29.68 | 386507 |
| Incidence | Global | Pertussis | Both | 2031 | 29.33 | 378298 |
| Incidence | Global | Pertussis | Both | 2032 | 28.98 | 369749 |
| Incidence | Global | Pertussis | Both | 2033 | 28.64 | 362068 |
| Incidence | Global | Pertussis | Both | 2034 | 28.29 | 355521 |
| Incidence | Global | Pertussis | Both | 2035 | 28.12 | 352290 |
| Incidence | Global | Pertussis | Both | 2036 | 27.95 | 349993 |
| Incidence | Global | Pertussis | Both | 2037 | 27.79 | 348039 |
| Incidence | Global | Pertussis | Both | 2038 | 27.62 | 346254 |
| Incidence | Global | Pertussis | Both | 2039 | 27.45 | 344634 |
| Incidence | Global | Pertussis | Both | 2040 | 27.29 | 343245 |
| Incidence | Global | Pertussis | Both | 2041 | 27.13 | 342010 |
| Incidence | Global | Pertussis | Both | 2042 | 26.96 | 340848 |
| Incidence | Global | Pertussis | Both | 2043 | 26.80 | 339703 |
| Incidence | Global | Pertussis | Both | 2044 | 26.64 | 338549 |
| Incidence | Global | Pertussis | Both | 2045 | 26.47 | 337388 |
| Incidence | Global | Pertussis | Both | 2046 | 26.31 | 336210 |

Sup_Table 18 Global Adolescent Pertussis Forecasts: Deaths, 1992 to 2046

| measure | location | cause | sex | year | ASR | case |
| --- | --- | --- | --- | --- | --- | --- |
| Deaths | Global | Pertussis | Both | 1992 | 0.66 | 7016 |
| Deaths | Global | Pertussis | Both | 1993 | 0.64 | 6836 |
| Deaths | Global | Pertussis | Both | 1994 | 0.61 | 6676 |
| Deaths | Global | Pertussis | Both | 1995 | 0.58 | 6458 |
| Deaths | Global | Pertussis | Both | 1996 | 0.58 | 6495 |
| Deaths | Global | Pertussis | Both | 1997 | 0.55 | 6383 |
| Deaths | Global | Pertussis | Both | 1998 | 0.55 | 6382 |
| Deaths | Global | Pertussis | Both | 1999 | 0.54 | 6420 |
| Deaths | Global | Pertussis | Both | 2000 | 0.55 | 6603 |
| Deaths | Global | Pertussis | Both | 2001 | 0.55 | 6578 |
| Deaths | Global | Pertussis | Both | 2002 | 0.53 | 6382 |
| Deaths | Global | Pertussis | Both | 2003 | 0.52 | 6286 |
| Deaths | Global | Pertussis | Both | 2004 | 0.51 | 6109 |
| Deaths | Global | Pertussis | Both | 2005 | 0.51 | 6028 |
| Deaths | Global | Pertussis | Both | 2006 | 0.49 | 5811 |
| Deaths | Global | Pertussis | Both | 2007 | 0.47 | 5583 |
| Deaths | Global | Pertussis | Both | 2008 | 0.47 | 5527 |
| Deaths | Global | Pertussis | Both | 2009 | 0.44 | 5231 |
| Deaths | Global | Pertussis | Both | 2010 | 0.43 | 5009 |
| Deaths | Global | Pertussis | Both | 2011 | 0.42 | 4893 |
| Deaths | Global | Pertussis | Both | 2012 | 0.41 | 4878 |
| Deaths | Global | Pertussis | Both | 2013 | 0.40 | 4789 |
| Deaths | Global | Pertussis | Both | 2014 | 0.40 | 4787 |
| Deaths | Global | Pertussis | Both | 2015 | 0.40 | 4748 |
| Deaths | Global | Pertussis | Both | 2016 | 0.38 | 4589 |
| Deaths | Global | Pertussis | Both | 2017 | 0.37 | 4496 |
| Deaths | Global | Pertussis | Both | 2018 | 0.36 | 4408 |
| Deaths | Global | Pertussis | Both | 2019 | 0.34 | 4242 |
| Deaths | Global | Pertussis | Both | 2020 | 0.16 | 2095 |
| Deaths | Global | Pertussis | Both | 2021 | 0.12 | 1547 |
| Deaths | Global | Pertussis | Both | 2022 | 0.24 | 3045 |
| Deaths | Global | Pertussis | Both | 2023 | 0.23 | 2939 |
| Deaths | Global | Pertussis | Both | 2024 | 0.22 | 2825 |
| Deaths | Global | Pertussis | Both | 2025 | 0.21 | 2776 |
| Deaths | Global | Pertussis | Both | 2026 | 0.21 | 2723 |
| Deaths | Global | Pertussis | Both | 2027 | 0.20 | 2664 |
| Deaths | Global | Pertussis | Both | 2028 | 0.20 | 2597 |
| Deaths | Global | Pertussis | Both | 2029 | 0.19 | 2522 |
| Deaths | Global | Pertussis | Both | 2030 | 0.19 | 2465 |
| Deaths | Global | Pertussis | Both | 2031 | 0.19 | 2403 |
| Deaths | Global | Pertussis | Both | 2032 | 0.18 | 2338 |
| Deaths | Global | Pertussis | Both | 2033 | 0.18 | 2279 |
| Deaths | Global | Pertussis | Both | 2034 | 0.18 | 2228 |
| Deaths | Global | Pertussis | Both | 2035 | 0.18 | 2203 |
| Deaths | Global | Pertussis | Both | 2036 | 0.17 | 2184 |
| Deaths | Global | Pertussis | Both | 2037 | 0.17 | 2168 |
| Deaths | Global | Pertussis | Both | 2038 | 0.17 | 2152 |
| Deaths | Global | Pertussis | Both | 2039 | 0.17 | 2138 |
| Deaths | Global | Pertussis | Both | 2040 | 0.17 | 2124 |
| Deaths | Global | Pertussis | Both | 2041 | 0.17 | 2112 |
| Deaths | Global | Pertussis | Both | 2042 | 0.17 | 2100 |
| Deaths | Global | Pertussis | Both | 2043 | 0.16 | 2089 |
| Deaths | Global | Pertussis | Both | 2044 | 0.16 | 2077 |
| Deaths | Global | Pertussis | Both | 2045 | 0.16 | 2065 |
| Deaths | Global | Pertussis | Both | 2046 | 0.16 | 2053 |

Sup_Table 19 Global Adolescent Pertussis Forecasts: DALYs, 1992 to 2046

| measure | location | cause | sex | year | ASR | case |
| --- | --- | --- | --- | --- | --- | --- |
| DALYs (Disability-Adjusted Life Years) | Global | Pertussis | Both | 1992 | 51.32 | 543864 |
| DALYs (Disability-Adjusted Life Years) | Global | Pertussis | Both | 1993 | 49.34 | 529937 |
| DALYs (Disability-Adjusted Life Years) | Global | Pertussis | Both | 1994 | 47.46 | 517603 |
| DALYs (Disability-Adjusted Life Years) | Global | Pertussis | Both | 1995 | 45.16 | 500874 |
| DALYs (Disability-Adjusted Life Years) | Global | Pertussis | Both | 1996 | 44.58 | 503633 |
| DALYs (Disability-Adjusted Life Years) | Global | Pertussis | Both | 1997 | 43.01 | 494889 |
| DALYs (Disability-Adjusted Life Years) | Global | Pertussis | Both | 1998 | 42.30 | 494719 |
| DALYs (Disability-Adjusted Life Years) | Global | Pertussis | Both | 1999 | 41.98 | 497533 |
| DALYs (Disability-Adjusted Life Years) | Global | Pertussis | Both | 2000 | 42.76 | 511575 |
| DALYs (Disability-Adjusted Life Years) | Global | Pertussis | Both | 2001 | 42.40 | 509614 |
| DALYs (Disability-Adjusted Life Years) | Global | Pertussis | Both | 2002 | 41.10 | 494432 |
| DALYs (Disability-Adjusted Life Years) | Global | Pertussis | Both | 2003 | 40.55 | 486950 |
| DALYs (Disability-Adjusted Life Years) | Global | Pertussis | Both | 2004 | 39.56 | 473309 |
| DALYs (Disability-Adjusted Life Years) | Global | Pertussis | Both | 2005 | 39.22 | 466984 |
| DALYs (Disability-Adjusted Life Years) | Global | Pertussis | Both | 2006 | 37.97 | 450275 |
| DALYs (Disability-Adjusted Life Years) | Global | Pertussis | Both | 2007 | 36.59 | 432694 |
| DALYs (Disability-Adjusted Life Years) | Global | Pertussis | Both | 2008 | 36.29 | 428285 |
| DALYs (Disability-Adjusted Life Years) | Global | Pertussis | Both | 2009 | 34.42 | 405563 |
| DALYs (Disability-Adjusted Life Years) | Global | Pertussis | Both | 2010 | 33.00 | 388522 |
| DALYs (Disability-Adjusted Life Years) | Global | Pertussis | Both | 2011 | 32.22 | 379502 |
| DALYs (Disability-Adjusted Life Years) | Global | Pertussis | Both | 2012 | 32.05 | 378339 |
| DALYs (Disability-Adjusted Life Years) | Global | Pertussis | Both | 2013 | 31.35 | 371467 |
| DALYs (Disability-Adjusted Life Years) | Global | Pertussis | Both | 2014 | 31.17 | 371359 |
| DALYs (Disability-Adjusted Life Years) | Global | Pertussis | Both | 2015 | 30.72 | 368484 |
| DALYs (Disability-Adjusted Life Years) | Global | Pertussis | Both | 2016 | 29.43 | 356306 |
| DALYs (Disability-Adjusted Life Years) | Global | Pertussis | Both | 2017 | 28.50 | 349135 |
| DALYs (Disability-Adjusted Life Years) | Global | Pertussis | Both | 2018 | 27.59 | 342358 |
| DALYs (Disability-Adjusted Life Years) | Global | Pertussis | Both | 2019 | 26.22 | 329590 |
| DALYs (Disability-Adjusted Life Years) | Global | Pertussis | Both | 2020 | 12.81 | 162962 |
| DALYs (Disability-Adjusted Life Years) | Global | Pertussis | Both | 2021 | 9.35 | 120134 |
| DALYs (Disability-Adjusted Life Years) | Global | Pertussis | Both | 2022 | 18.48 | 237412 |
| DALYs (Disability-Adjusted Life Years) | Global | Pertussis | Both | 2023 | 17.72 | 229479 |
| DALYs (Disability-Adjusted Life Years) | Global | Pertussis | Both | 2024 | 16.95 | 220935 |
| DALYs (Disability-Adjusted Life Years) | Global | Pertussis | Both | 2025 | 16.58 | 217193 |
| DALYs (Disability-Adjusted Life Years) | Global | Pertussis | Both | 2026 | 16.22 | 213185 |
| DALYs (Disability-Adjusted Life Years) | Global | Pertussis | Both | 2027 | 15.85 | 208763 |
| DALYs (Disability-Adjusted Life Years) | Global | Pertussis | Both | 2028 | 15.48 | 203620 |
| DALYs (Disability-Adjusted Life Years) | Global | Pertussis | Both | 2029 | 15.11 | 197888 |
| DALYs (Disability-Adjusted Life Years) | Global | Pertussis | Both | 2030 | 14.88 | 193516 |
| DALYs (Disability-Adjusted Life Years) | Global | Pertussis | Both | 2031 | 14.65 | 188668 |
| DALYs (Disability-Adjusted Life Years) | Global | Pertussis | Both | 2032 | 14.43 | 183669 |
| DALYs (Disability-Adjusted Life Years) | Global | Pertussis | Both | 2033 | 14.20 | 179137 |
| DALYs (Disability-Adjusted Life Years) | Global | Pertussis | Both | 2034 | 13.97 | 175201 |
| DALYs (Disability-Adjusted Life Years) | Global | Pertussis | Both | 2035 | 13.86 | 173307 |
| DALYs (Disability-Adjusted Life Years) | Global | Pertussis | Both | 2036 | 13.76 | 171894 |
| DALYs (Disability-Adjusted Life Years) | Global | Pertussis | Both | 2037 | 13.65 | 170657 |
| DALYs (Disability-Adjusted Life Years) | Global | Pertussis | Both | 2038 | 13.54 | 169493 |
| DALYs (Disability-Adjusted Life Years) | Global | Pertussis | Both | 2039 | 13.43 | 168396 |
| DALYs (Disability-Adjusted Life Years) | Global | Pertussis | Both | 2040 | 13.33 | 167419 |
| DALYs (Disability-Adjusted Life Years) | Global | Pertussis | Both | 2041 | 13.22 | 166504 |
| DALYs (Disability-Adjusted Life Years) | Global | Pertussis | Both | 2042 | 13.12 | 165620 |
| DALYs (Disability-Adjusted Life Years) | Global | Pertussis | Both | 2043 | 13.01 | 164743 |
| DALYs (Disability-Adjusted Life Years) | Global | Pertussis | Both | 2044 | 12.91 | 163859 |
| DALYs (Disability-Adjusted Life Years) | Global | Pertussis | Both | 2045 | 12.80 | 162969 |
| DALYs (Disability-Adjusted Life Years) | Global | Pertussis | Both | 2046 | 12.70 | 162069 |
